# Supplementary material for: GTPase Rab11b and effector Rab11-FIP2 promote NLRP3 stability during inflammasome priming
Source: EMBO J. 2026 Mar 25;45(9):2991–3021. doi: 10.1038/s44318-026-00755-7 (PMC13144346; doi:10.1038/s44318-026-00755-7)
Supplement: Supplementary file 2 — Appendix [file 44318_2026_755_MOESM2_ESM.pdf]

Appendix for:

GTPase Rab11b and effector Rab11-FIP2 promote NLRP3 stability during inflammasome priming

Table of contents

**Appendix Figures**	Page
Appendix Figure S1	2
Appendix Figure S2	3
Appendix Figure S3	4
Appendix Figure S4	5
Appendix Figure S5	7

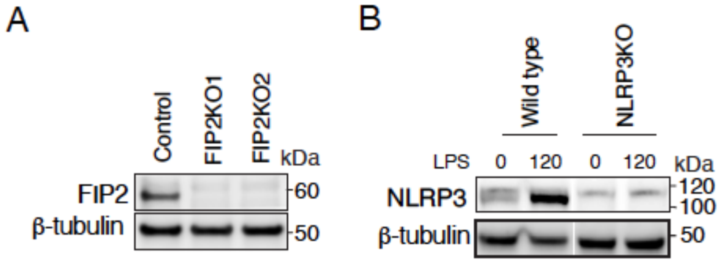

Appendix Figure S1: Verification of antibody specificity in FIP2-, or NLRP3 knockout cells by Western blotting

(**A**) Immunoblots of FIP2 in FIP2 knockout (KO) THP-1-derived-macrophage cell lines left untreated or primed with 100 ng/mL LPS for 2 h. (**B**) Immunoblots of NLRP3 in unstimulated NLRP3KO THP-1-derived-macrophages.

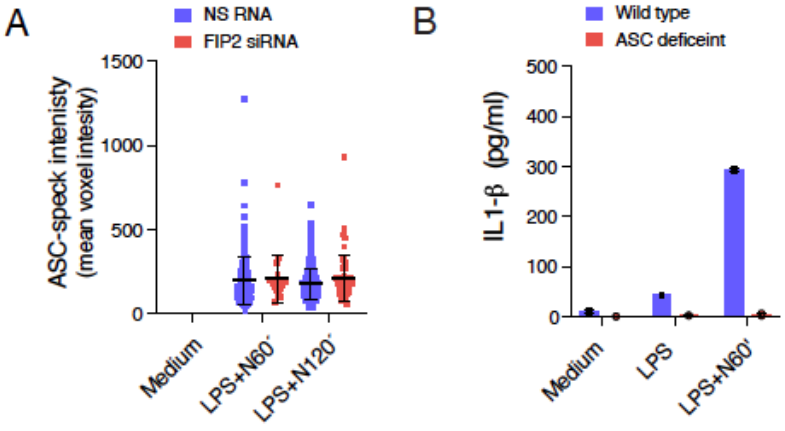

Appendix Figure S2: ASC-speck intensity and IL-1b

(**A**) ASC-speck intensity of the total amount of ASC specks shown in Figure 4C. (**B**) IL-1 β ELISA on two technical replicates from ASCKO THP-1-derived macrophage cell line primed with 100 ng/mL LPS and treated with 10 μM nigericin as indicated.

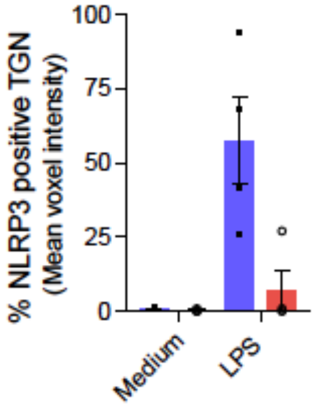

Appendix Figure S3: Quantification of NLRP3 positive TGN structures
The percentage of NLRP3 positive TGN was calculated in the NS RNA and FIP2 siRNA treated THP-1-derived macrophages form Figure 6E. To calculate the % positive TGN we used the highest value of NLRP3 in the TGN of unstimulated cells from the same experiments and subtracted this from all NLRP3 values in LPS primed cells. p =0.0100 (NS RNA unstimulatedvs LPS stimulated cells) and p = 0.0048 (NS RNA vs FIP2 siRNA) LPS primed cells. Data are presented as mean +/- s.e.m. and shown as black bars. (Two-way ANOVA Tukey's multiple comparisons test with adj. p values).

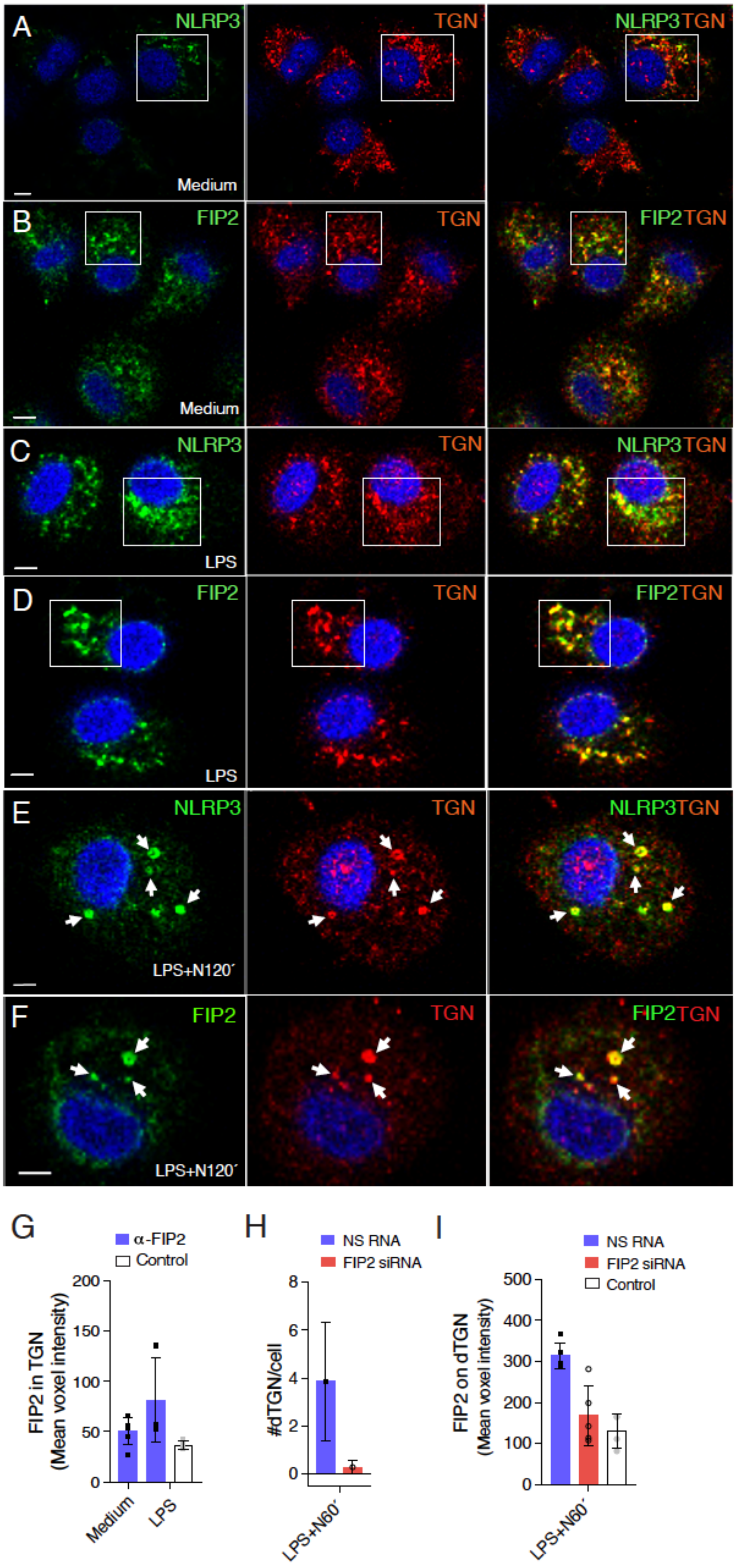
A
NLRP3
TGN
NLRP3TGN
Medium
B
FIP2
TGN
FIP2TGN
Medium
C
NLRP3
TGN
NLRP3TGN
LPS
D
FIP2
TGN
FIP2TGN
LPS
E
NLRP3
TGN
NLRP3TGN
LPS+N120´
F
FIP2
TGN
FIP2TGN
LPS+N120´
G
α-FIP2
Control
FIP2 in TGN
(Mean voxel intensity)
200
150
100
50
0
Medium
LPS
H
NS RNA
FIP2 siRNA
#dTGN/cell
8
6
4
2
0
LPS+N60´
I
NS RNA
FIP2 siRNA
Control
FIP2 on dTGN
(Mean voxel intensity)
500
400
300
200
100
0
LPS+N60´

Appendix Figure S4: FIP2 and NLRP3 both localize to the TGN in primary human Macrophages

(**A**) Confocal image showing NLRP3 (green) and TGN46 (red) in unstimulated human macrophages. (**B**) Confocal image showing FIP2 (green) and TGN46 (red) in unstimulated human macrophages. (**C**) Confocal image showing NLRP3 (green) and TGN46 (red) in LPS stimulated human macrophages.(**D**) Confocal image showing FIP2 (green) and TGN46 (red) in LPS stimulated human macrophages. (**E**) Confocal image showing NLRP3 (green) on TGN46 (red) in dTGN structures of LPS stimulated and nigericin stimulated human macrophages. (**F**) Confocal image showing FIP2 (green) and TGN46 structures (red) in dTGN structures of LPS stimulated and nigericin stimulated human macrophages. (**G**) Quantification of NLRP3 in the TGN-ring in unstimulated and LPS-primed THP-1-derived macrophages. (**H**) Number of dTGN structures in LPS and nigericin treated THP-1-derived macrophages. Quantification of NLRP3 in the dTGN structures in the cells of H. (**I**) Number of dTGN structures in LPS and nigericin treated THP-1-derived macrophages. The cells were left untreated, primed with 100 ng/mL LPS for 2 h or primed with 100 ng/mL LPS for 2 h and treated with 5 mM nigericin for 2 h.

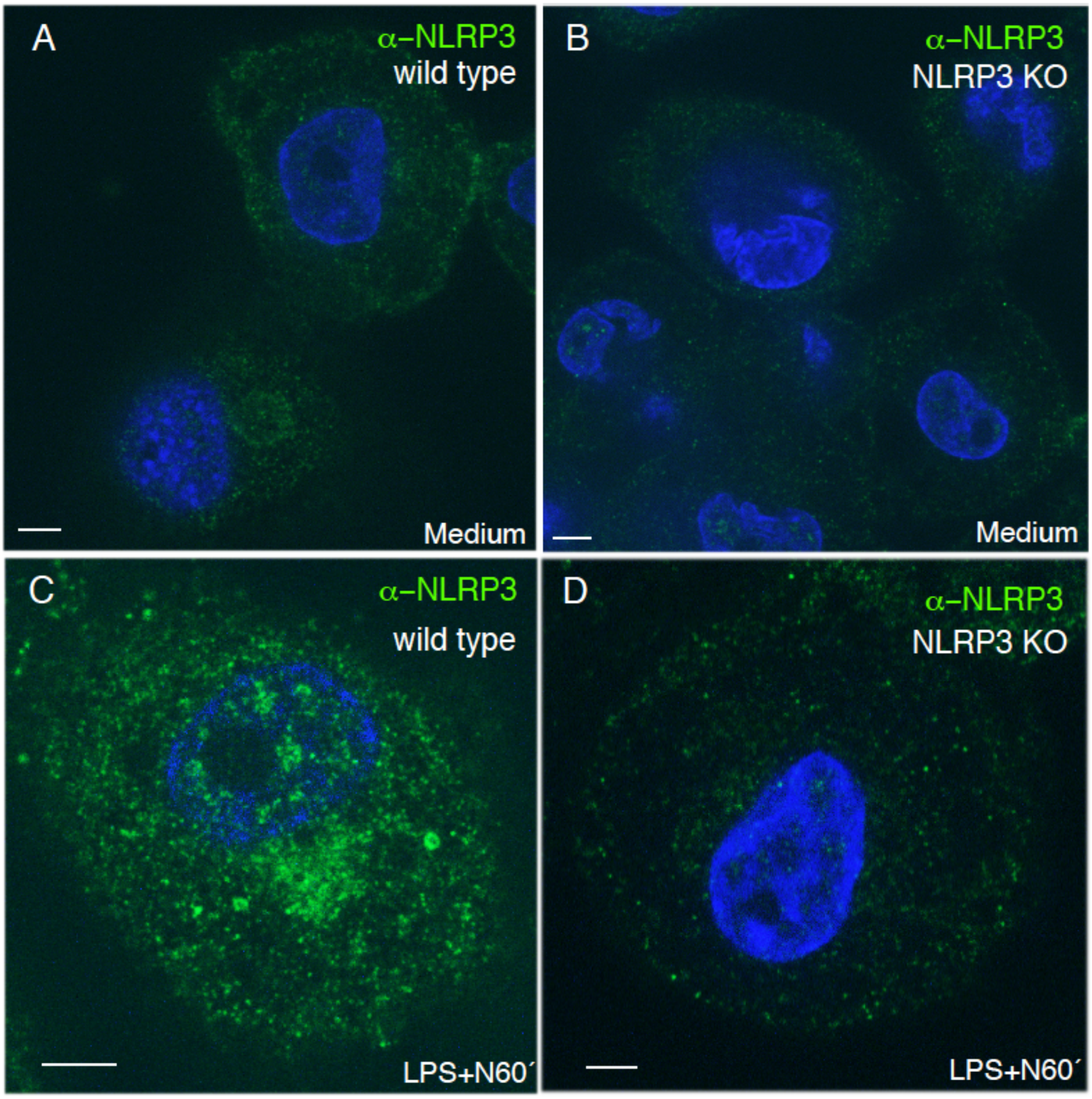

Appendix Figure S5: Verification of NLRP3 antibody specificity by immuno-staining
(**A**) Confocal image of NLRP3 (green) in unstimulated wild type THP-1-derived macrophages (**B**) Confocal image of unstimulated NLRP3KO THP-1-derived macrophages. (**C**) Confocal image of NLRP3 (green) in nigericin treated LPS primed THP-1-derived macrophages. (**D**) Confocal image of NLRP3 (green) in nigericin treated LPS primed THP-1 NLRP3KO cells. The cells were primed with 100 ng/mL LPS for 2 h and treated with 10 μM nigericin for 1 h. Scale bar = 5 μm.
