# Supplementary figures and images for: GTPase Rab11b and effector Rab11-FIP2 promote NLRP3 stability during inflammasome priming (part 1 of 2)
Source: EMBO J. 2026 Mar 25;45(9):2991–3021. doi: 10.1038/s44318-026-00755-7 (PMC13144346; doi:10.1038/s44318-026-00755-7)

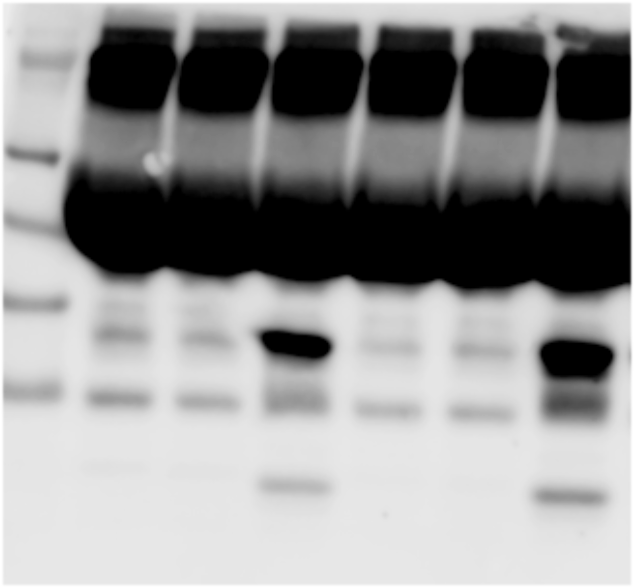

Supplement: Supplementary file 3 — Source data Fig. 1 [file 44318_2026_755_MOESM3_ESM.zip › EMBOJ-2025-121050 Figure 1/Fig. 1 western TIF/1F/1F sup_IL1b_Flg_FIP2.tif]

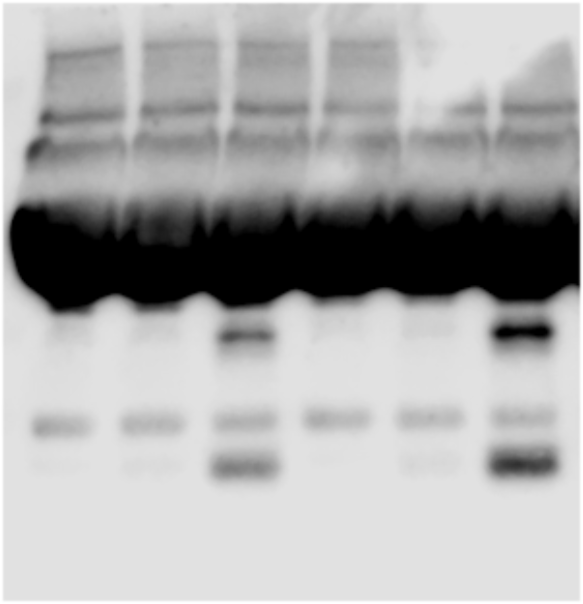

Supplement: Supplementary file 3 — Source data Fig. 1 [file 44318_2026_755_MOESM3_ESM.zip › EMBOJ-2025-121050 Figure 1/Fig. 1 western TIF/1F/1F sup_casp1_Flg_FIP2.tif]

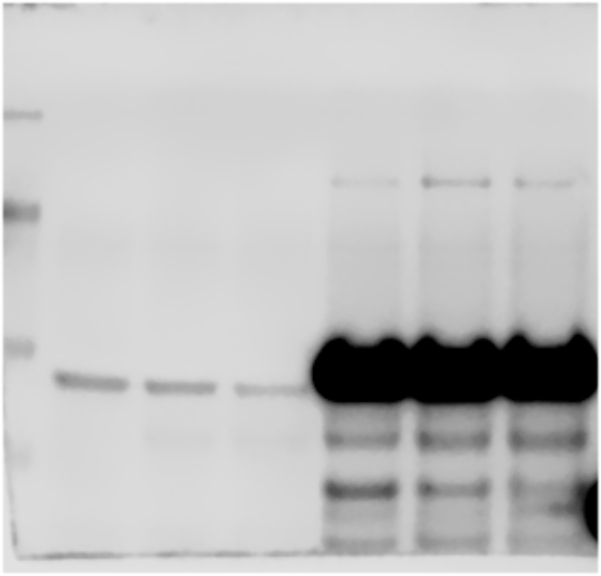

Supplement: Supplementary file 3 — Source data Fig. 1 [file 44318_2026_755_MOESM3_ESM.zip › EMBOJ-2025-121050 Figure 1/Fig. 1 western TIF/1F/1F lys_FIP2_Flg_FIP2.tif]

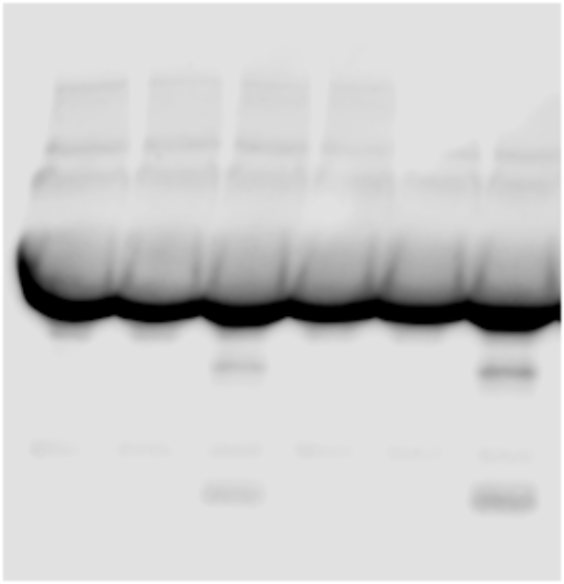

Supplement: Supplementary file 3 — Source data Fig. 1 [file 44318_2026_755_MOESM3_ESM.zip › EMBOJ-2025-121050 Figure 1/Fig. 1 western TIF/1F/1F sup_casp1_Flg_FIP2 ligth exposure.tif]

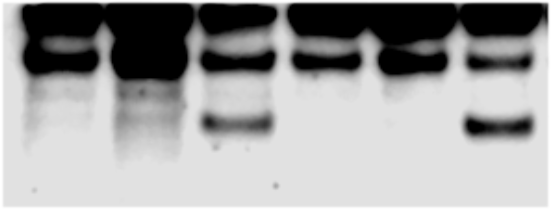

Supplement: Supplementary file 3 — Source data Fig. 1 [file 44318_2026_755_MOESM3_ESM.zip › EMBOJ-2025-121050 Figure 1/Fig. 1 western TIF/1F/1F lys_GSDMD_Flg_FIP2.tif]

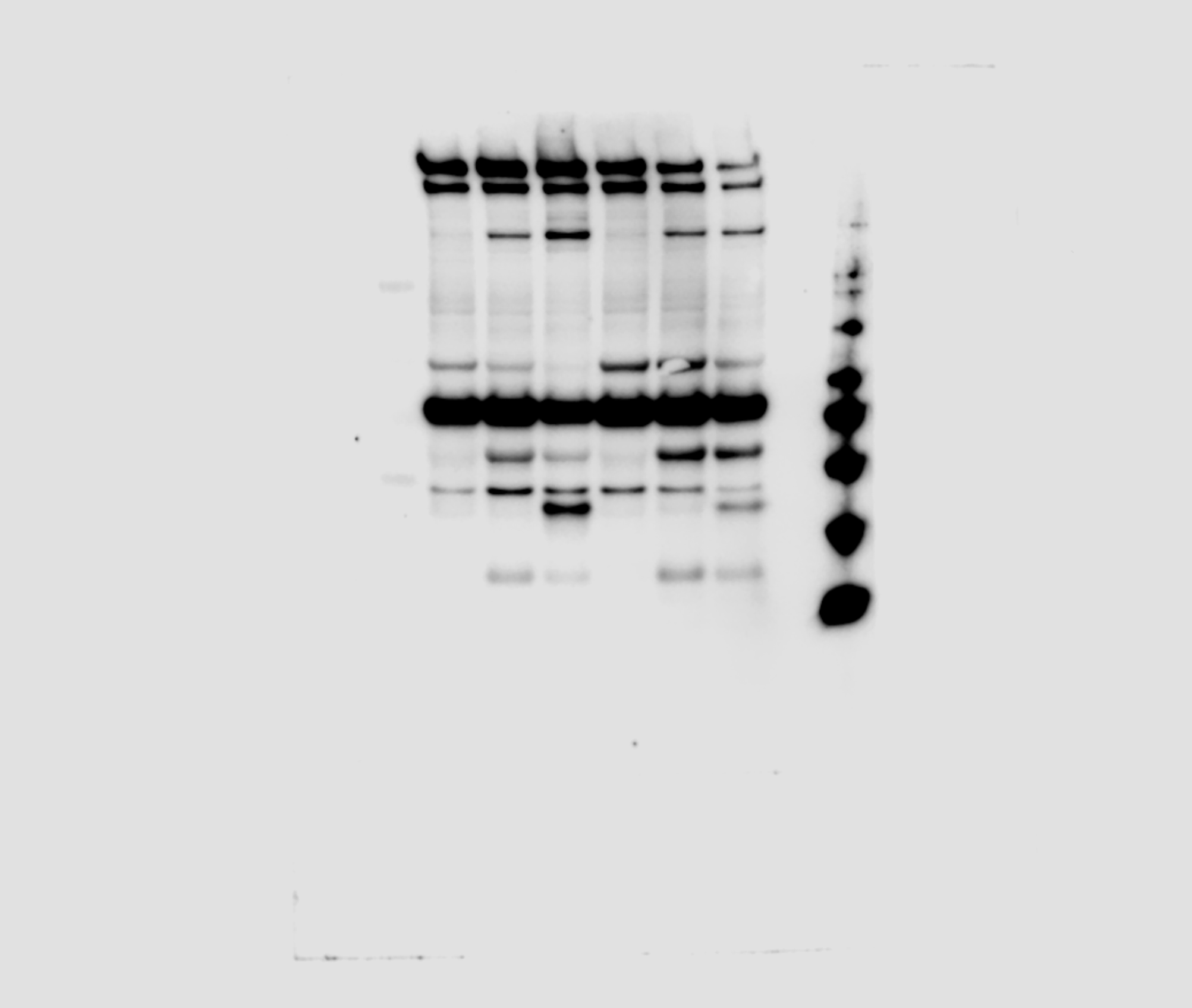

Supplement: Supplementary file 3 — Source data Fig. 1 [file 44318_2026_755_MOESM3_ESM.zip › EMBOJ-2025-121050 Figure 1/Fig. 1 western TIF/1I/1I_lys_GSDMD.tif]

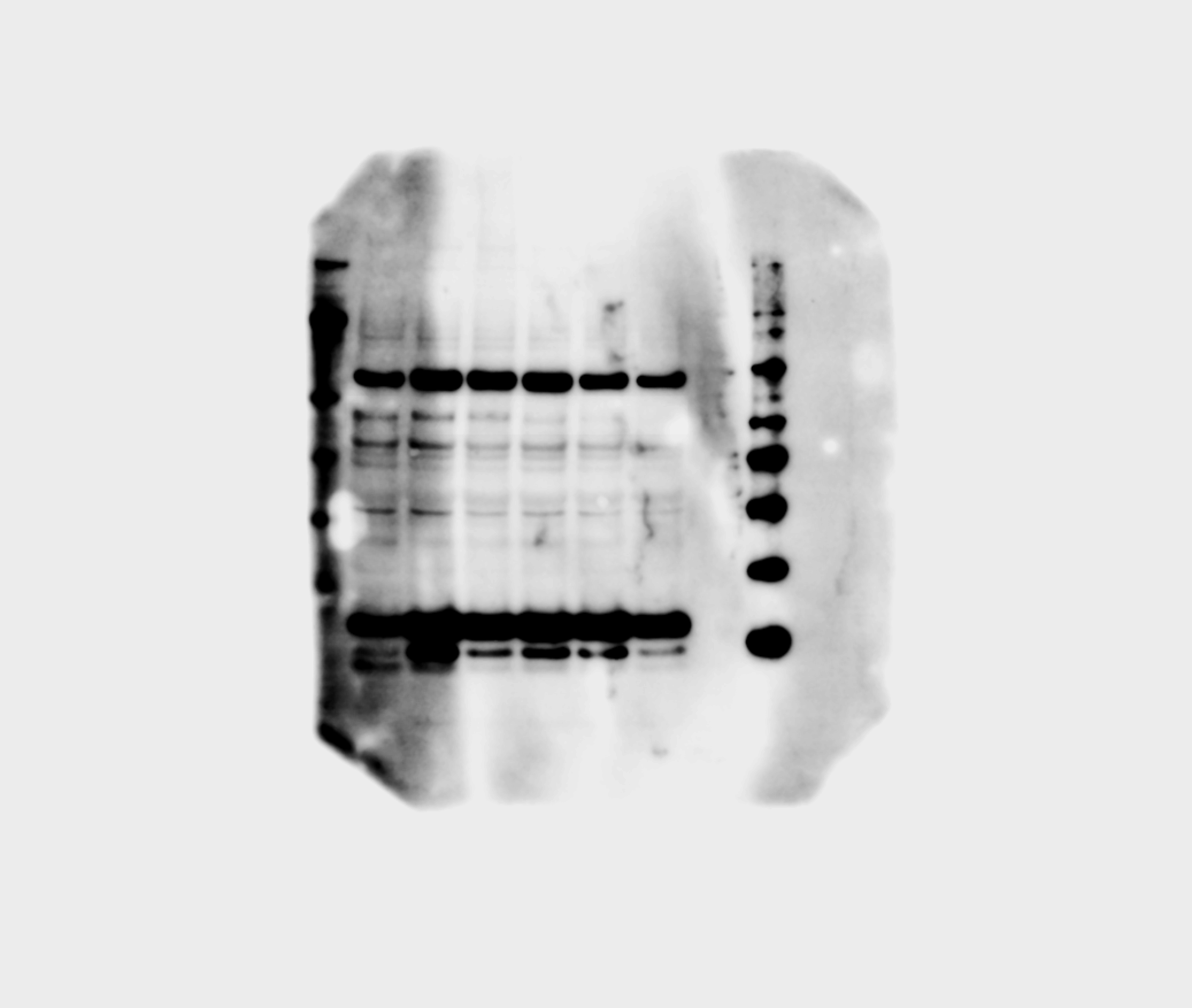

Supplement: Supplementary file 3 — Source data Fig. 1 [file 44318_2026_755_MOESM3_ESM.zip › EMBOJ-2025-121050 Figure 1/Fig. 1 western TIF/1I/1I_lys_FIP2.tif]

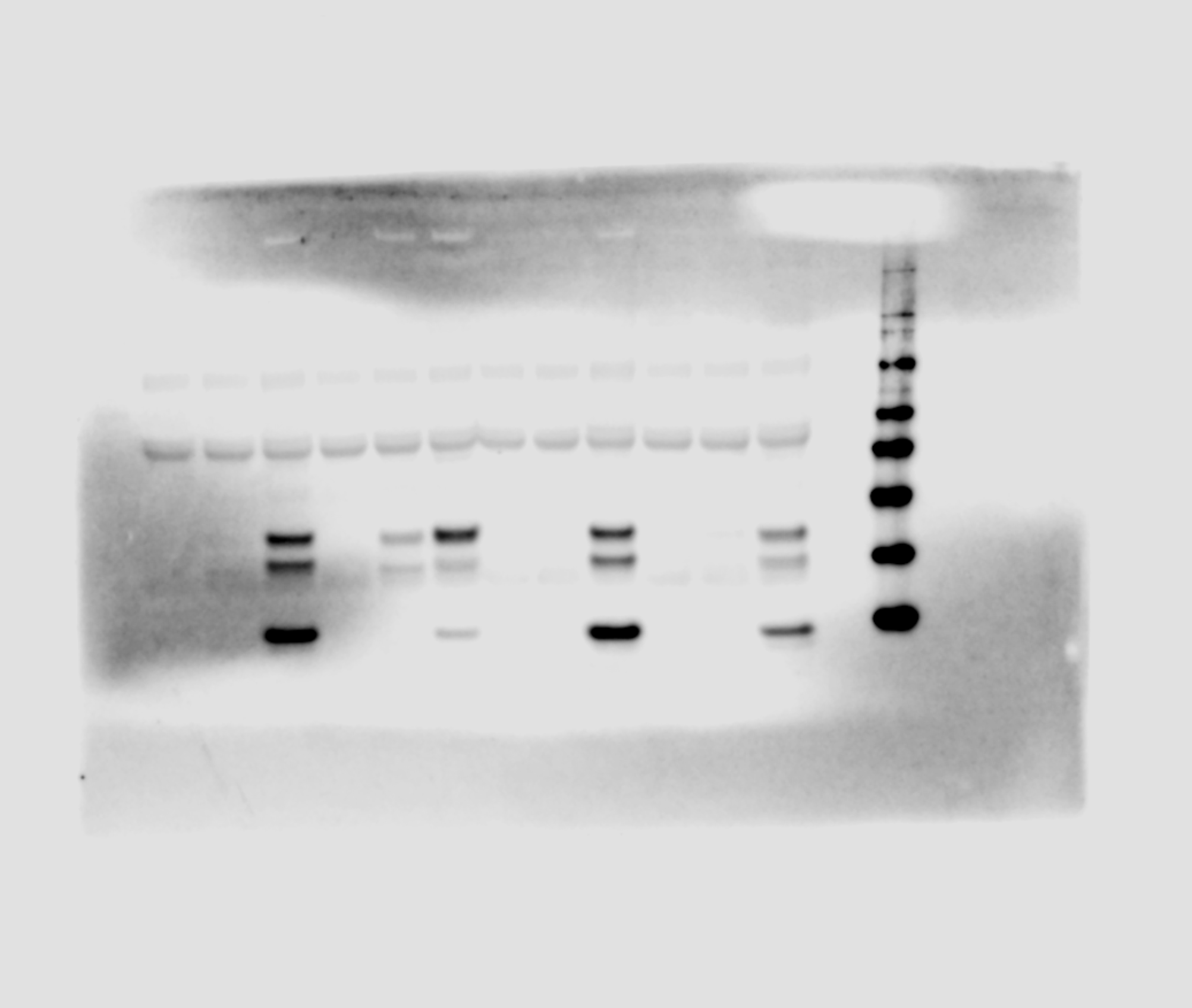

Supplement: Supplementary file 3 — Source data Fig. 1 [file 44318_2026_755_MOESM3_ESM.zip › EMBOJ-2025-121050 Figure 1/Fig. 1 western TIF/1I/1I sup_IL1B.tif]

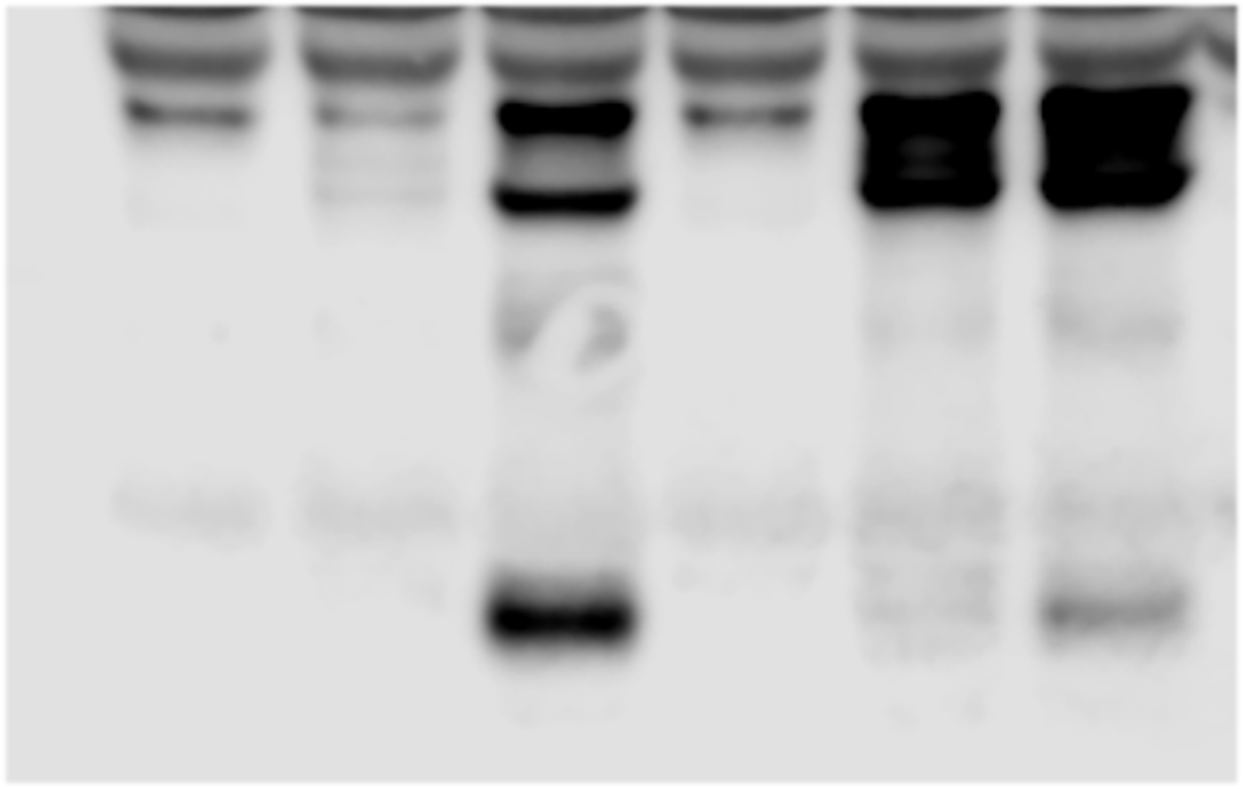

Supplement: Supplementary file 3 — Source data Fig. 1 [file 44318_2026_755_MOESM3_ESM.zip › EMBOJ-2025-121050 Figure 1/Fig. 1 western TIF/1I/1I sup_casp1.tif]

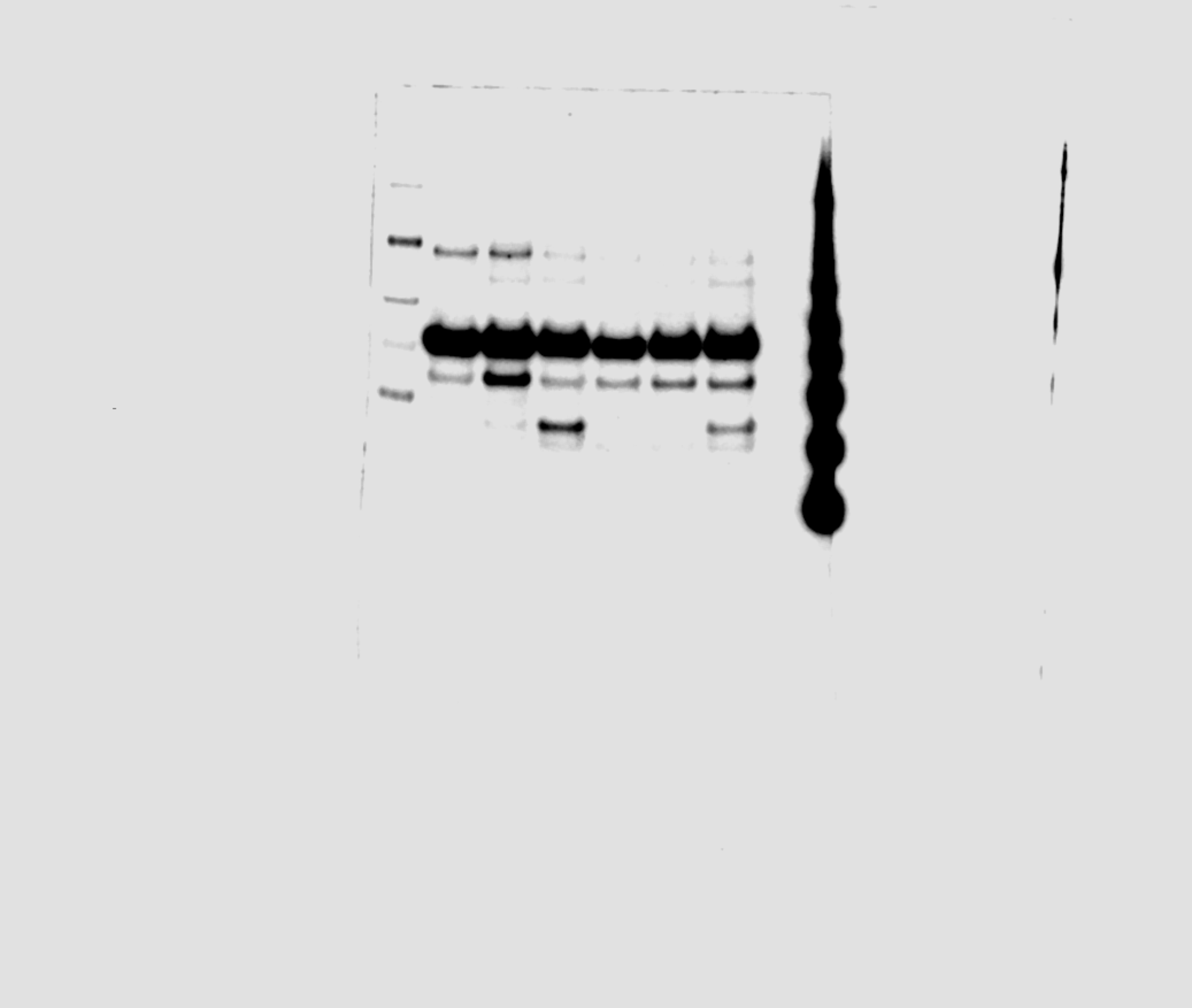

Supplement: Supplementary file 3 — Source data Fig. 1 [file 44318_2026_755_MOESM3_ESM.zip › EMBOJ-2025-121050 Figure 1/Fig. 1 western TIF/1C/1C gsdmd.tif]

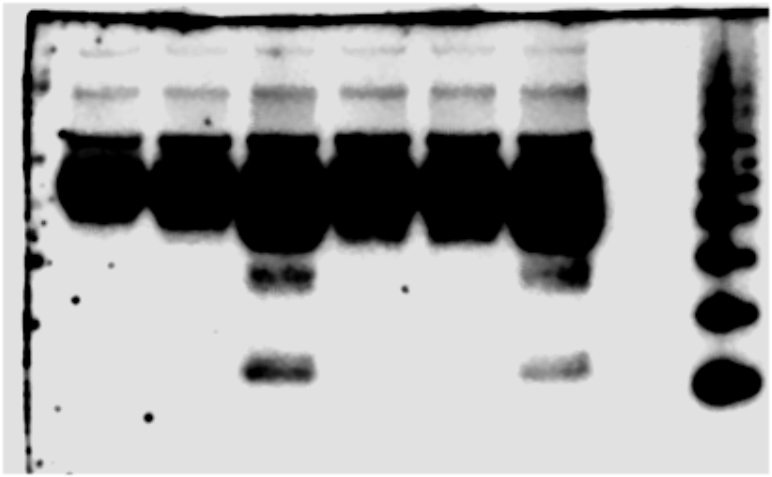

Supplement: Supplementary file 3 — Source data Fig. 1 [file 44318_2026_755_MOESM3_ESM.zip › EMBOJ-2025-121050 Figure 1/Fig. 1 western TIF/1C/1C casp1-sup.tif]

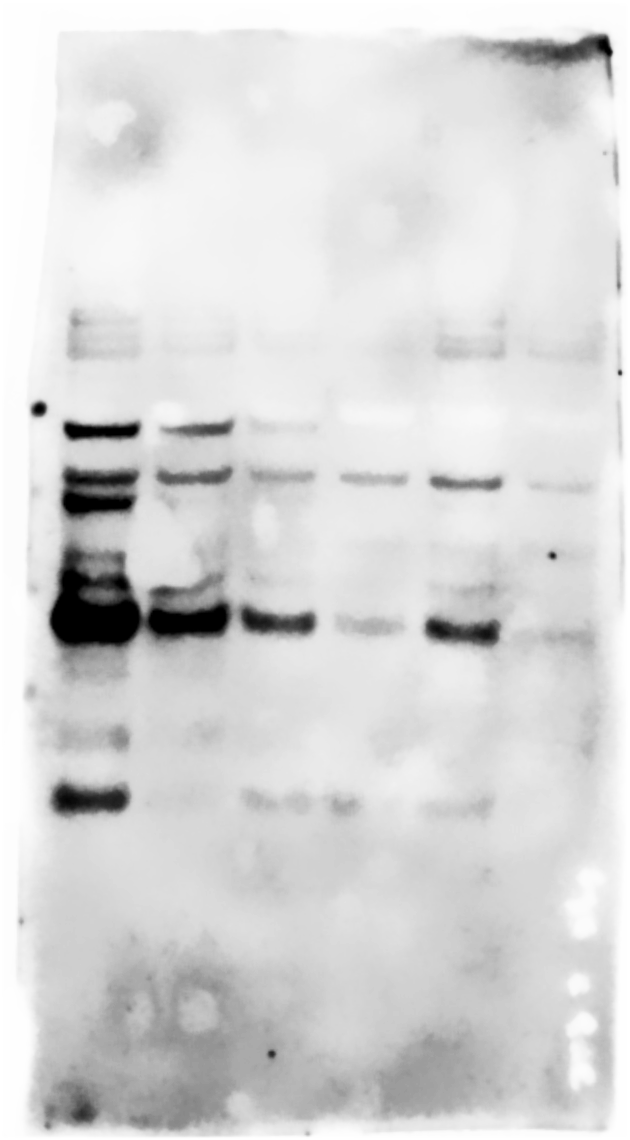

Supplement: Supplementary file 3 — Source data Fig. 1 [file 44318_2026_755_MOESM3_ESM.zip › EMBOJ-2025-121050 Figure 1/Fig. 1 western TIF/1C/1C_FIP2.tif]

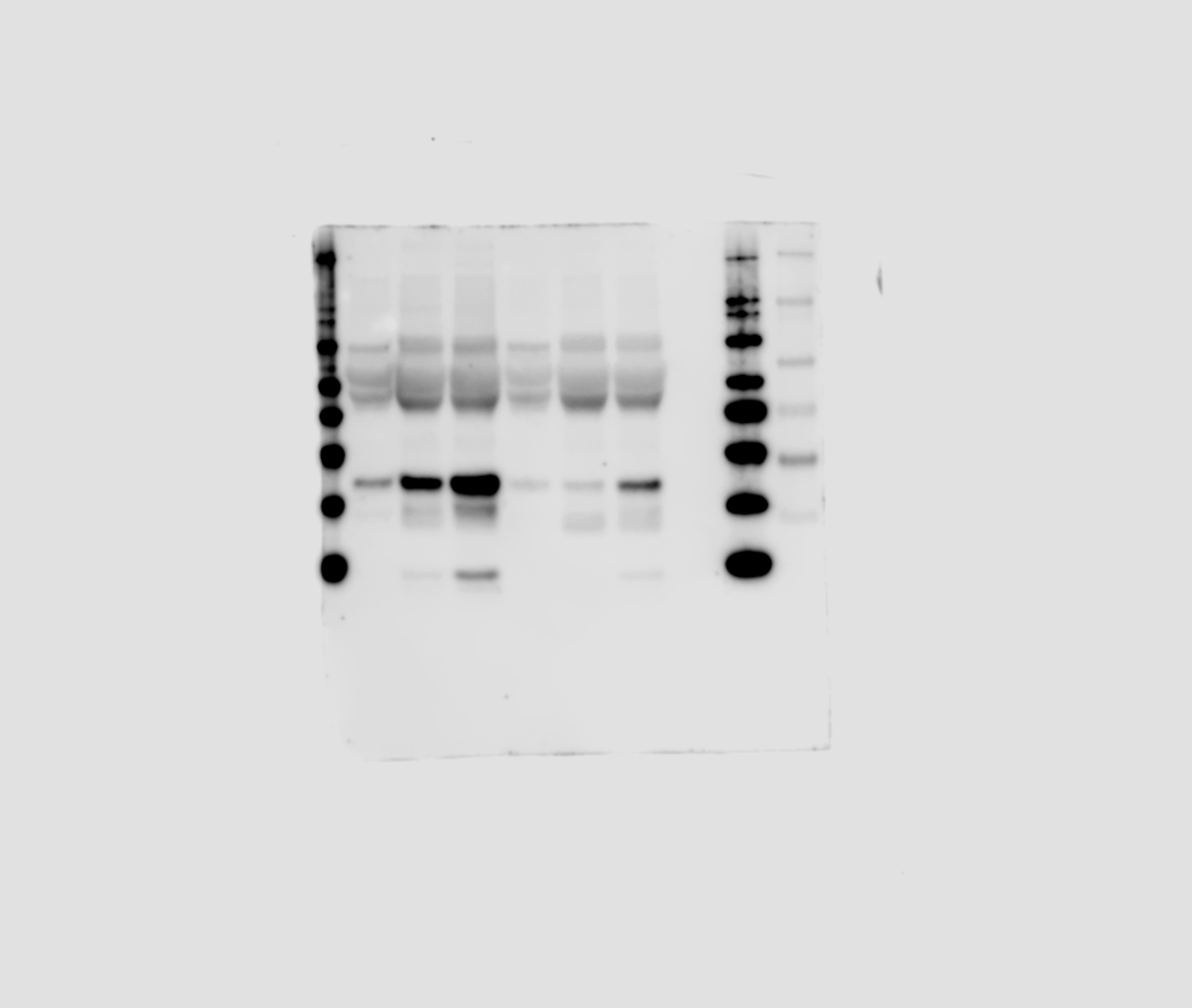

Supplement: Supplementary file 3 — Source data Fig. 1 [file 44318_2026_755_MOESM3_ESM.zip › EMBOJ-2025-121050 Figure 1/Fig. 1 western TIF/1C/1C_sup_IL1b.tif]

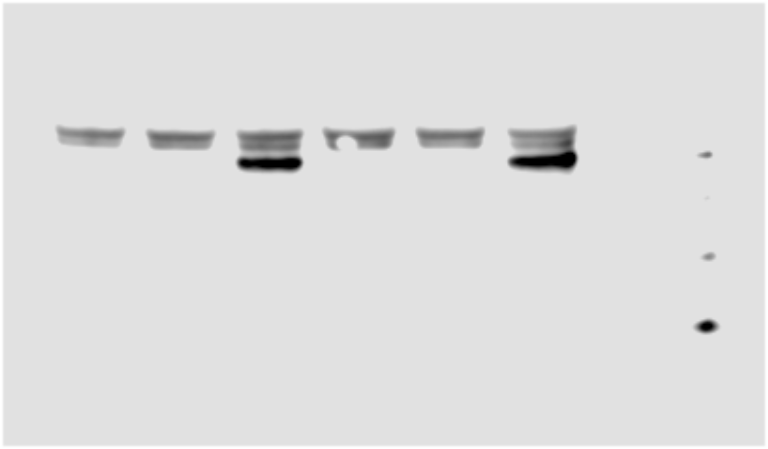

Supplement: Supplementary file 3 — Source data Fig. 1 [file 44318_2026_755_MOESM3_ESM.zip › EMBOJ-2025-121050 Figure 1/Fig. 1 western TIF/1C/1C casp1-sup ligth exp.tif]

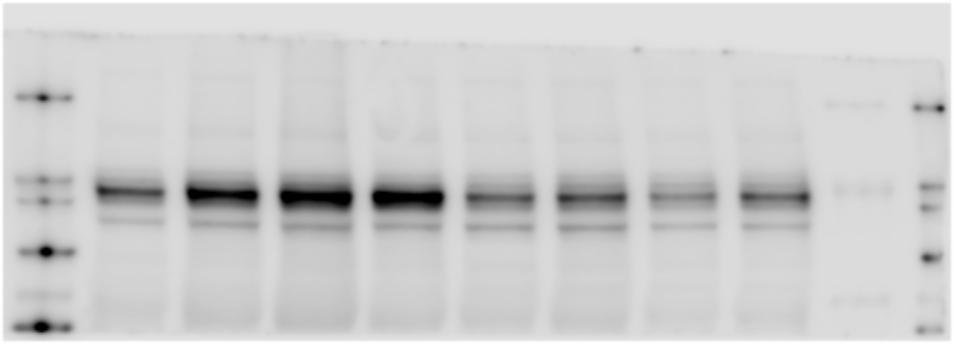

Supplement: Supplementary file 4 — Source data Fig. 2 [file 44318_2026_755_MOESM4_ESM.zip › EMBOJ-2025-121050 Figure 2/Fig. 2 western TIF/1A NLRP3 NS RNA_FIP2siRNA.tif]

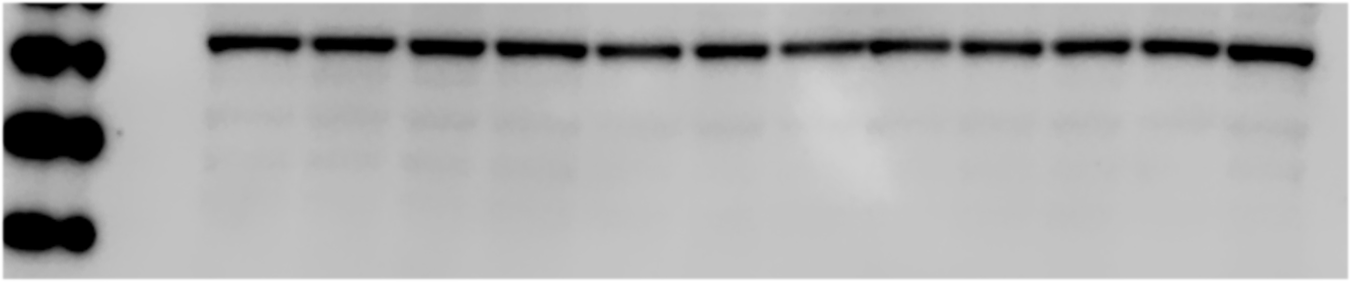

Supplement: Supplementary file 4 — Source data Fig. 2 [file 44318_2026_755_MOESM4_ESM.zip › EMBOJ-2025-121050 Figure 2/Fig. 2 western TIF/1G beta-tubulin NS RNA_FIP2siRNA.tif]

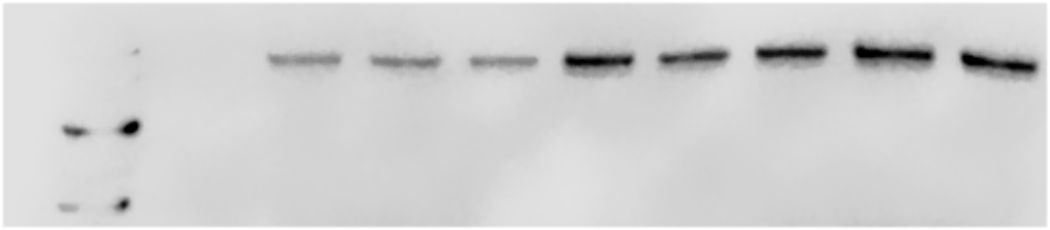

Supplement: Supplementary file 4 — Source data Fig. 2 [file 44318_2026_755_MOESM4_ESM.zip › EMBOJ-2025-121050 Figure 2/Fig. 2 western TIF/1C NLRP3_Flag-FIP2.tif]

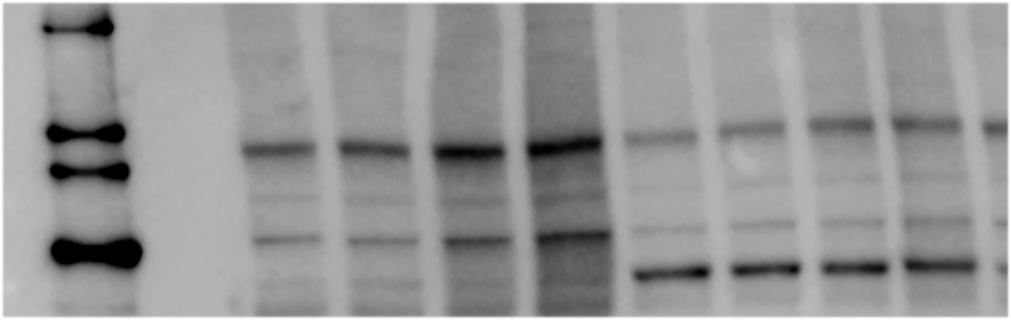

Supplement: Supplementary file 4 — Source data Fig. 2 [file 44318_2026_755_MOESM4_ESM.zip › EMBOJ-2025-121050 Figure 2/Fig. 2 western TIF/1G NLRP3_NS RNA_FIP2siRNA.tif]

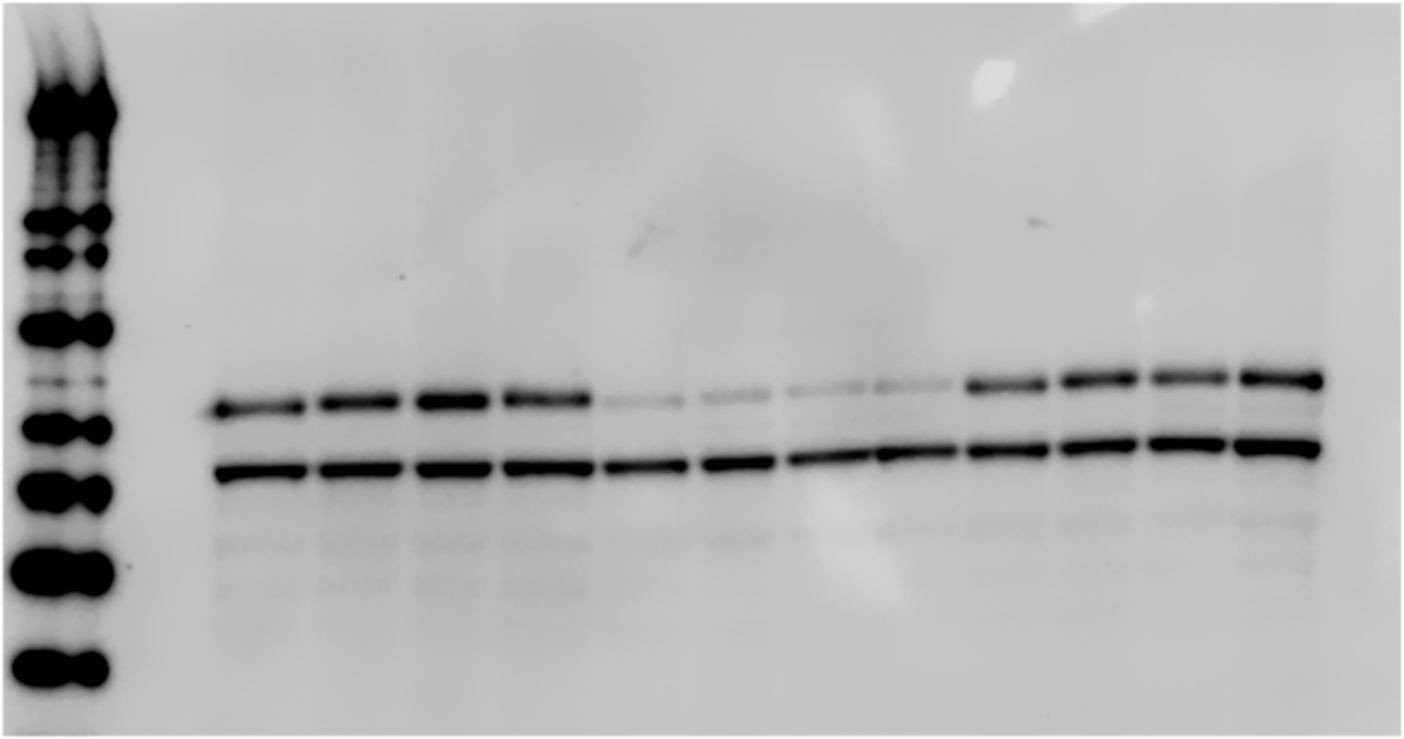

Supplement: Supplementary file 4 — Source data Fig. 2 [file 44318_2026_755_MOESM4_ESM.zip › EMBOJ-2025-121050 Figure 2/Fig. 2 western TIF/1G FIP2_NS RNA_FIP2siRNA.tif]

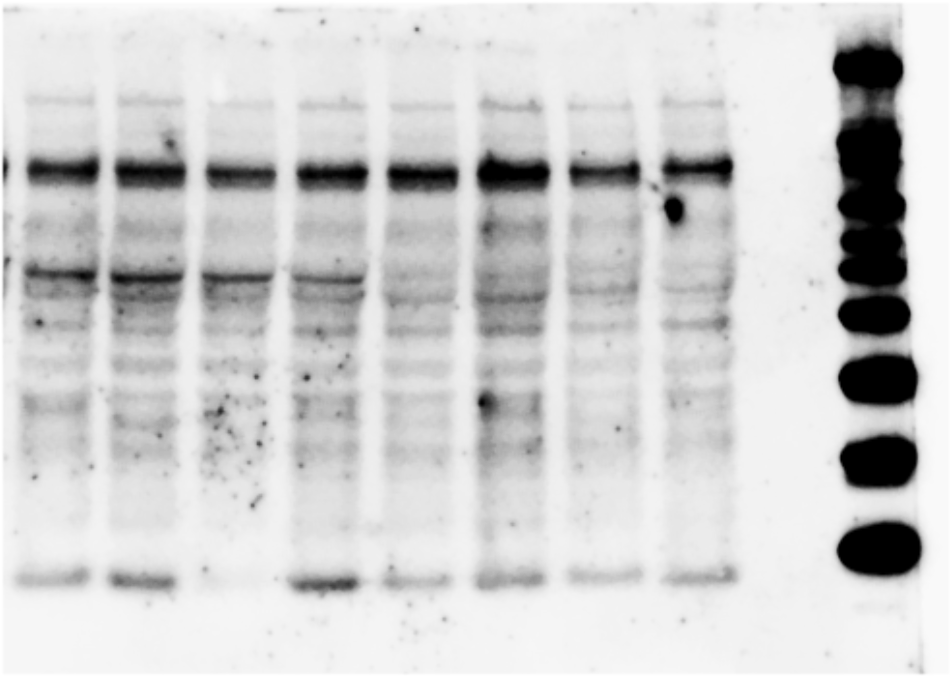

Supplement: Supplementary file 4 — Source data Fig. 2 [file 44318_2026_755_MOESM4_ESM.zip › EMBOJ-2025-121050 Figure 2/Fig. 2 western TIF/1A FIP2 NS RNA_FIP2siRNA.tif]

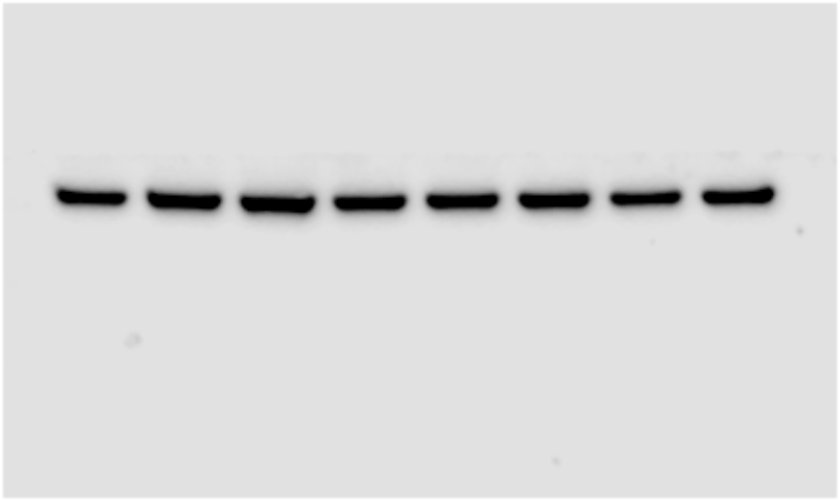

Supplement: Supplementary file 4 — Source data Fig. 2 [file 44318_2026_755_MOESM4_ESM.zip › EMBOJ-2025-121050 Figure 2/Fig. 2 western TIF/1A beta-tubulin NS RNA_FIP2siRNA.tif]

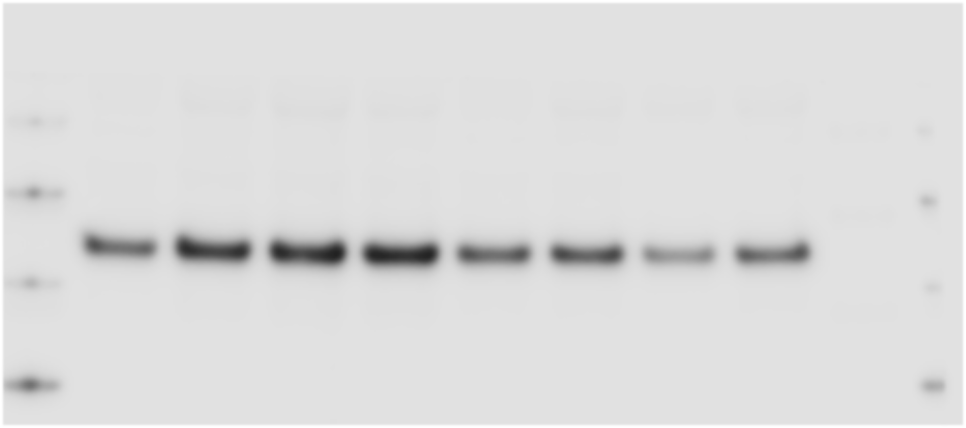

Supplement: Supplementary file 4 — Source data Fig. 2 [file 44318_2026_755_MOESM4_ESM.zip › EMBOJ-2025-121050 Figure 2/Fig. 2 western TIF/1A pro-IL1b NS RNA_FIP2siRNA.tif]

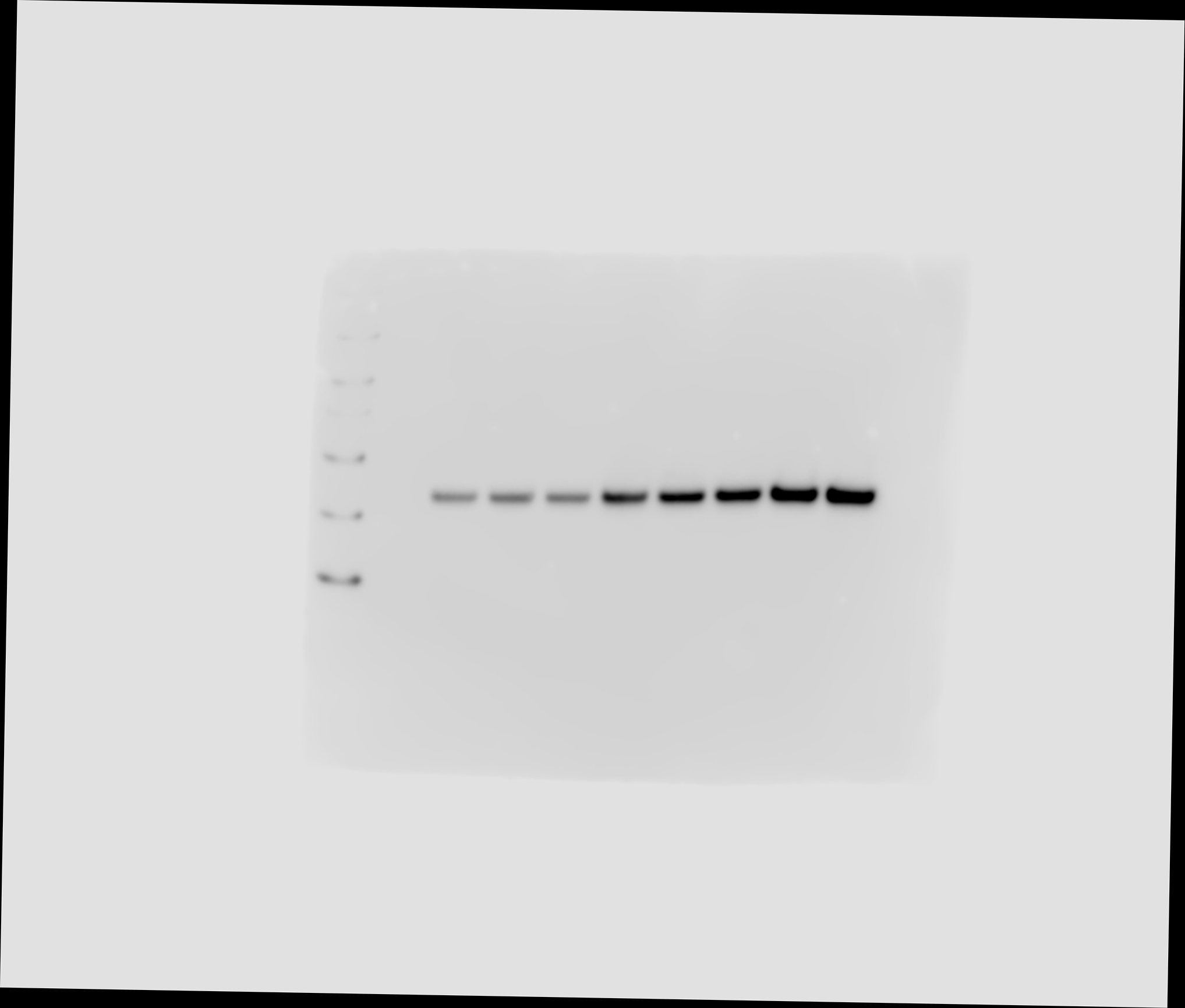

Supplement: Supplementary file 4 — Source data Fig. 2 [file 44318_2026_755_MOESM4_ESM.zip › EMBOJ-2025-121050 Figure 2/Fig. 2 western TIF/1C pro-IL1b_Flag-FIP2.tif]

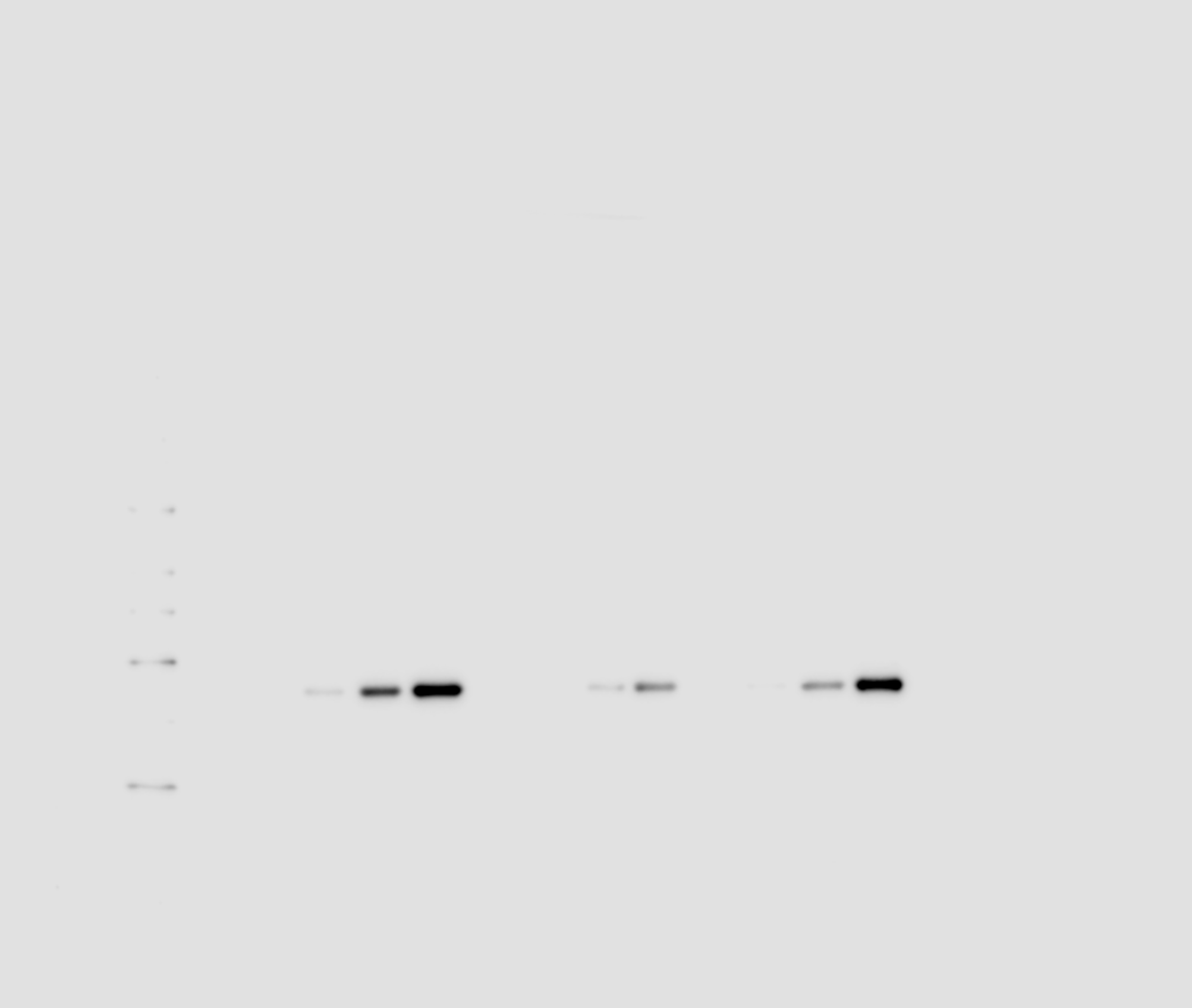

Supplement: Supplementary file 4 — Source data Fig. 2 [file 44318_2026_755_MOESM4_ESM.zip › EMBOJ-2025-121050 Figure 2/Fig. 2 western TIF/1G pro-IL1b_NS RNA_FIP2siRNA.tif]

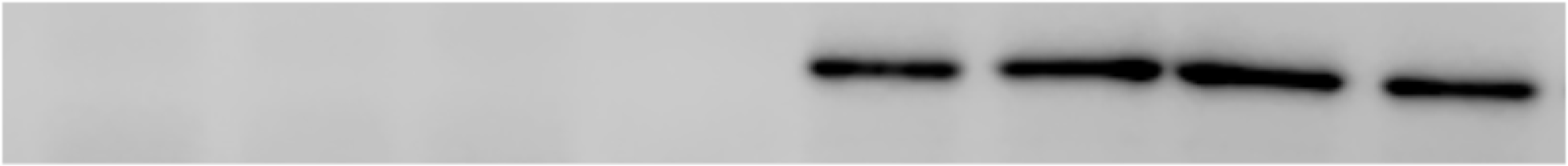

Supplement: Supplementary file 4 — Source data Fig. 2 [file 44318_2026_755_MOESM4_ESM.zip › EMBOJ-2025-121050 Figure 2/Fig. 2 western TIF/1C Flag Flag-FIP2.tif]

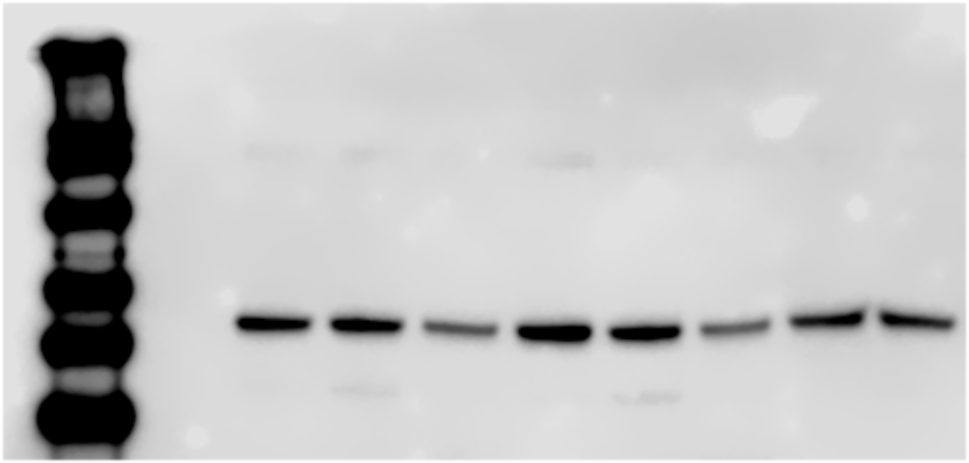

Supplement: Supplementary file 4 — Source data Fig. 2 [file 44318_2026_755_MOESM4_ESM.zip › EMBOJ-2025-121050 Figure 2/Fig. 2 western TIF/1C beta-tubulin_Flag-FIP2.tif]

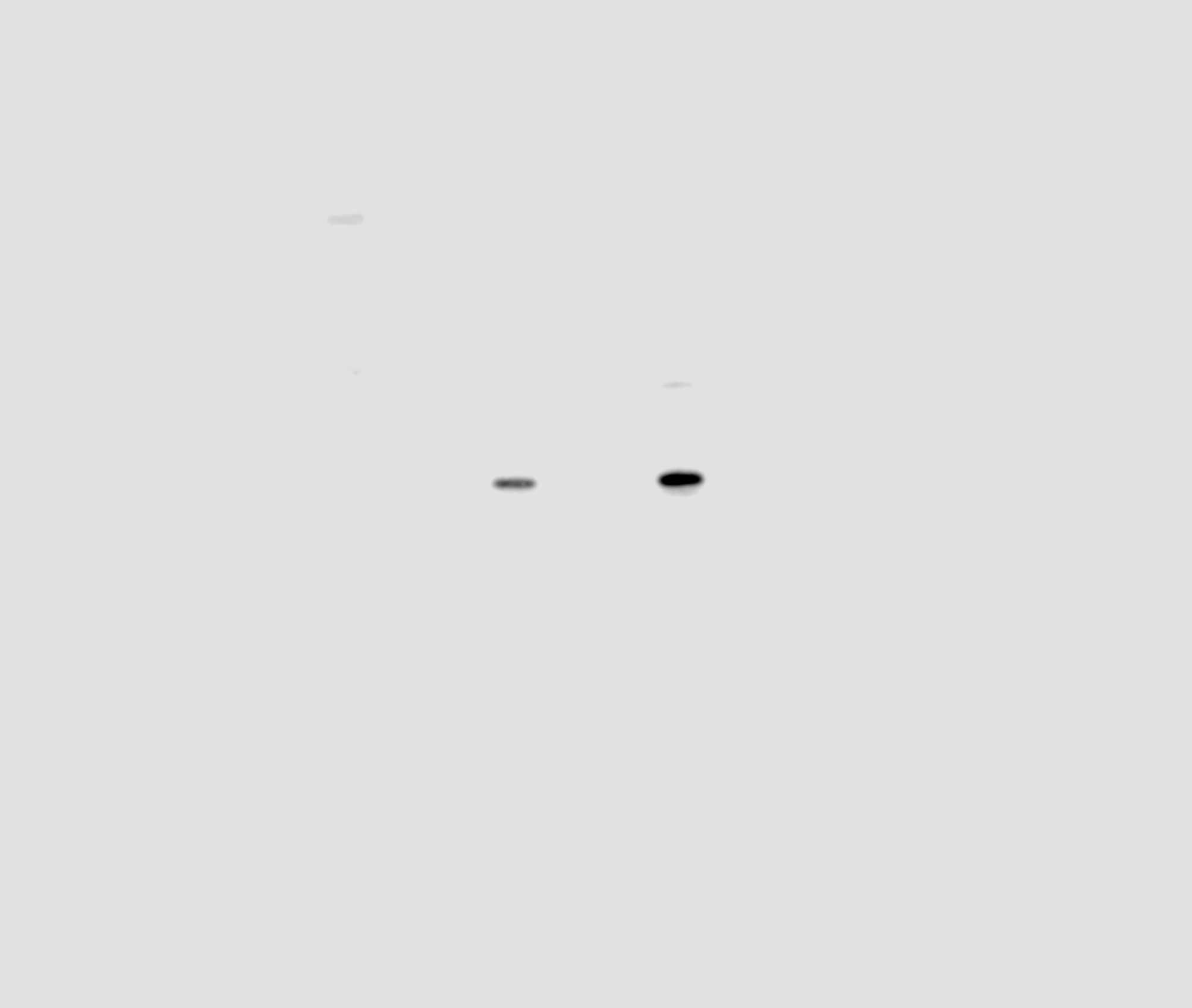

Supplement: Supplementary file 5 — Source data Fig. 3 [file 44318_2026_755_MOESM5_ESM.zip › EMBOJ-2025-121050 Figure 3/Fig. 3 western TIF/3C sup_IL1b p17 spesific.tif]

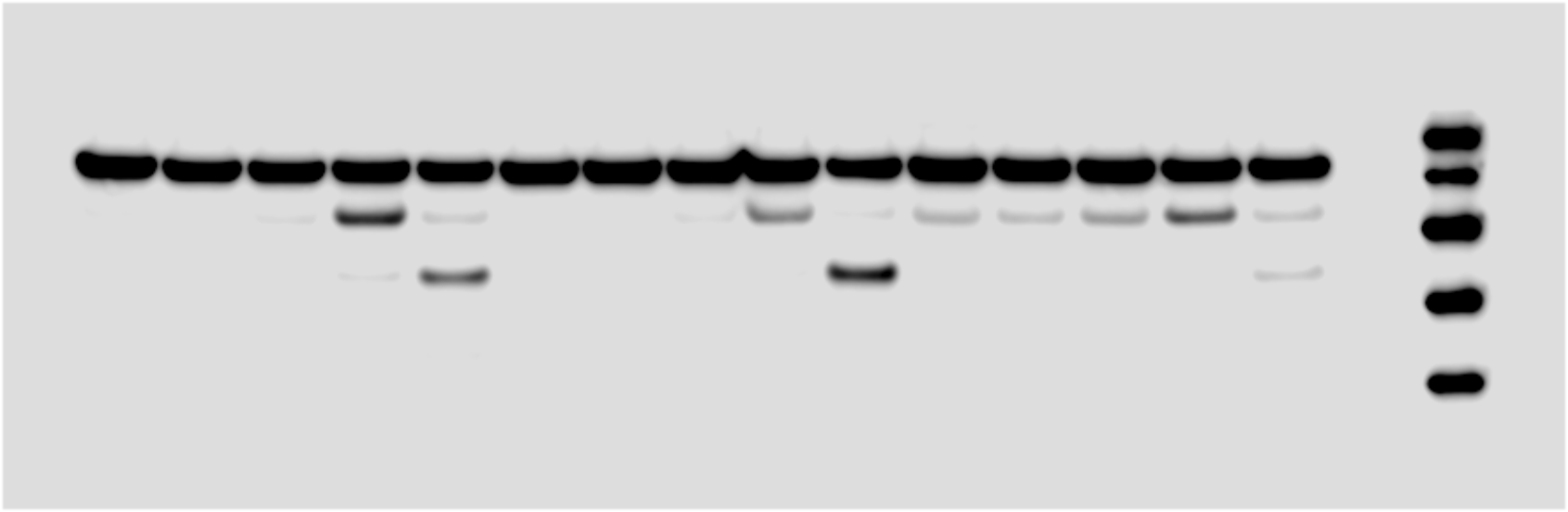

Supplement: Supplementary file 5 — Source data Fig. 3 [file 44318_2026_755_MOESM5_ESM.zip › EMBOJ-2025-121050 Figure 3/Fig. 3 western TIF/3C lys_GSDMD.tif]

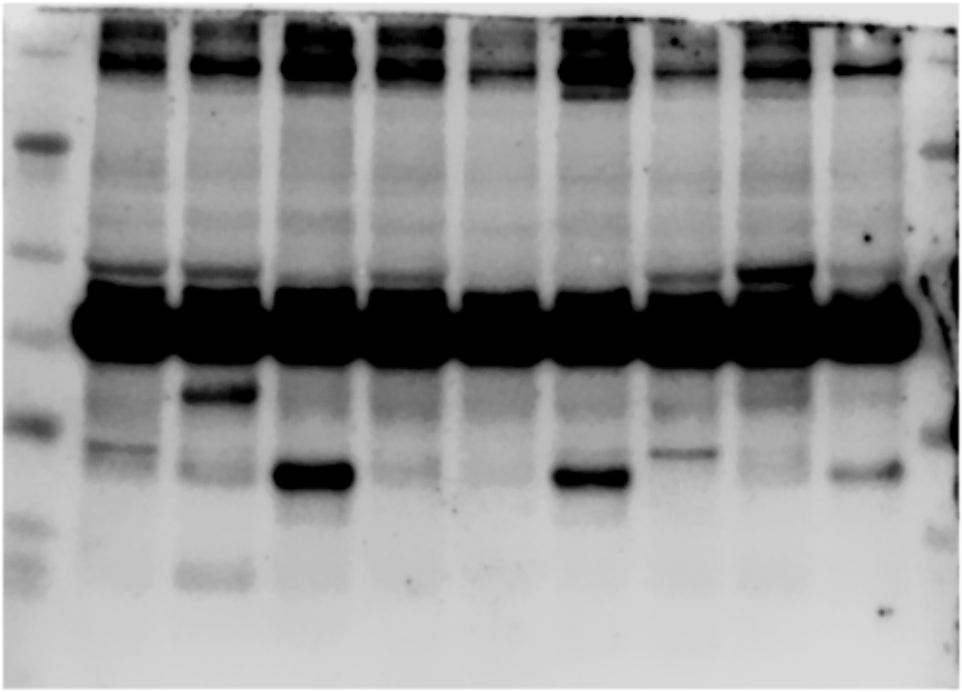

Supplement: Supplementary file 5 — Source data Fig. 3 [file 44318_2026_755_MOESM5_ESM.zip › EMBOJ-2025-121050 Figure 3/Fig. 3 western TIF/3D lys_GSDMD D1.tif]

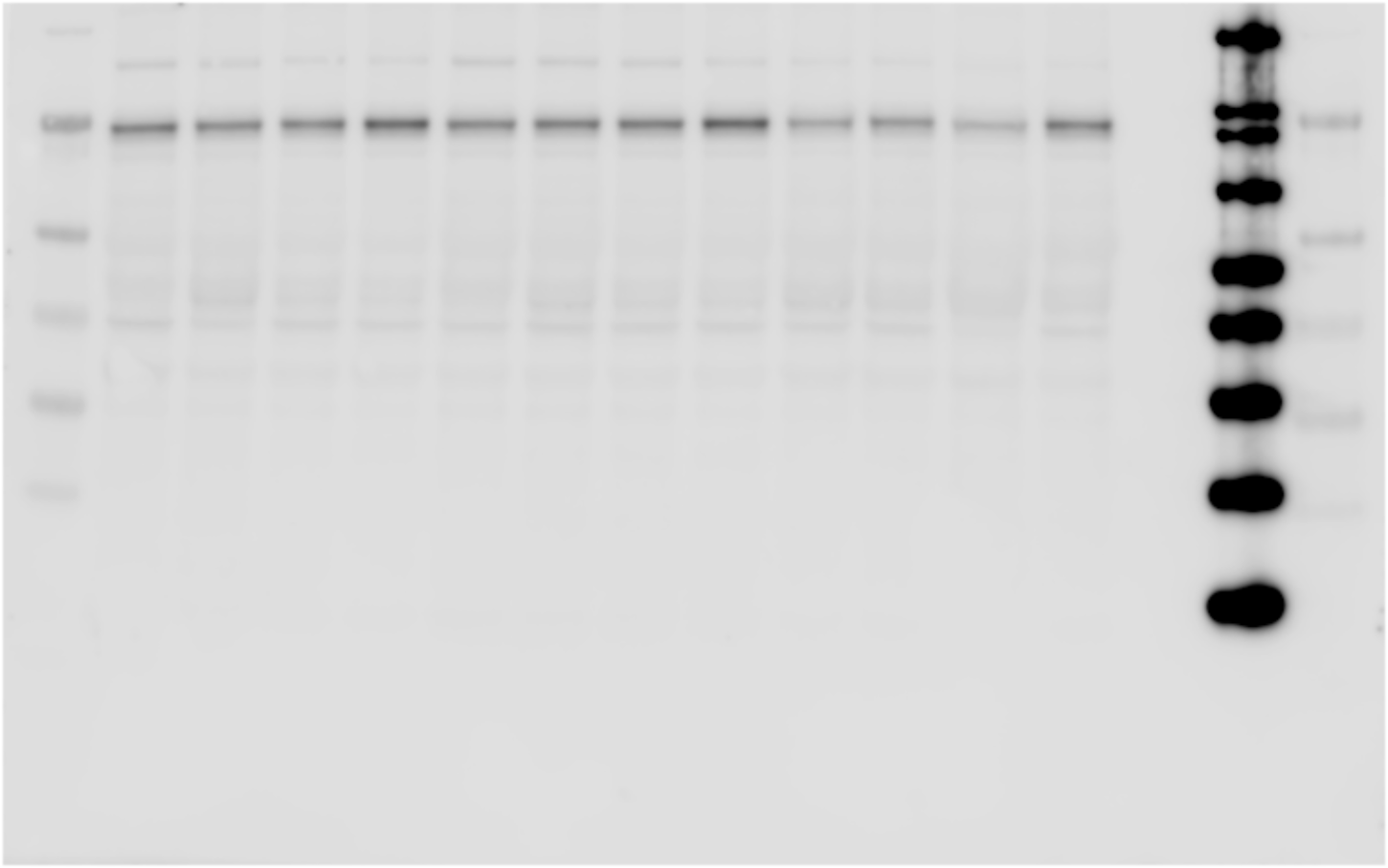

Supplement: Supplementary file 5 — Source data Fig. 3 [file 44318_2026_755_MOESM5_ESM.zip › EMBOJ-2025-121050 Figure 3/Fig. 3 western TIF/3E lys_NLRP3.tif]

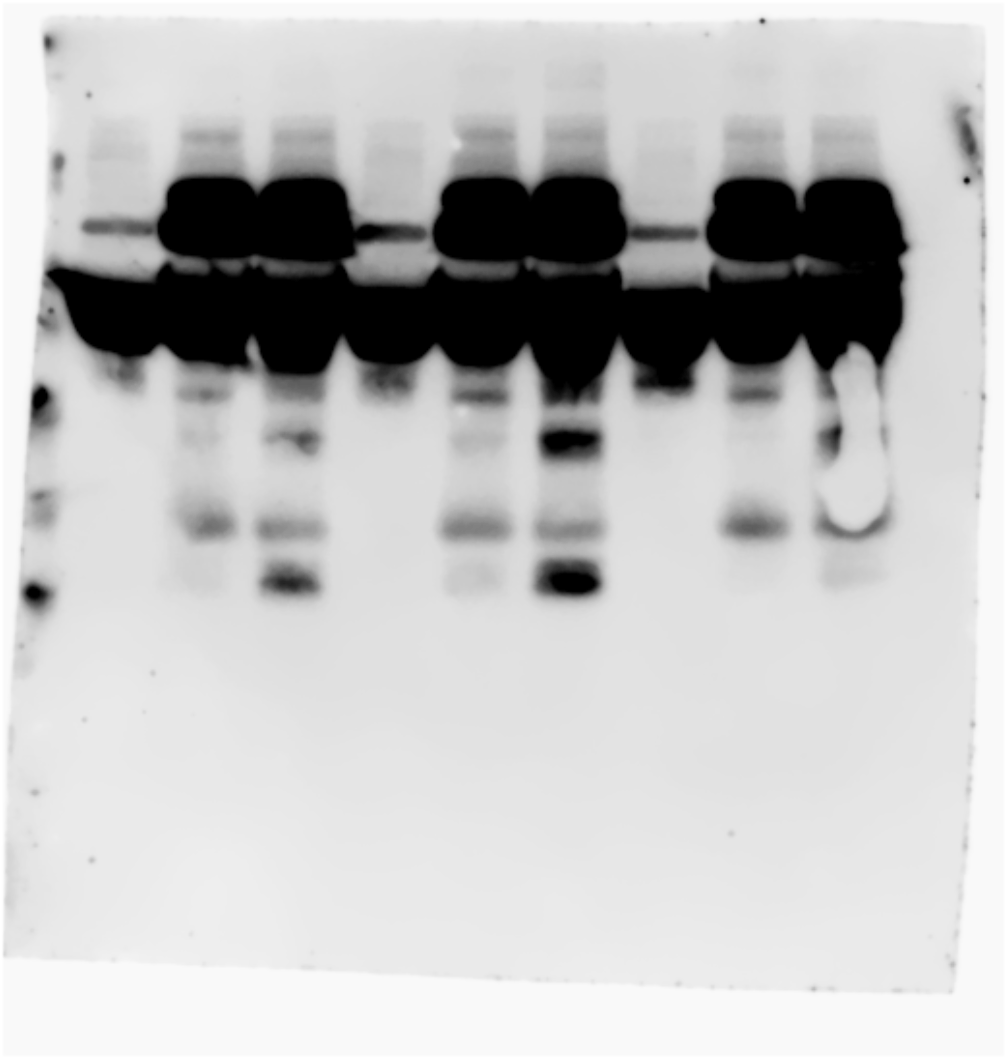

Supplement: Supplementary file 5 — Source data Fig. 3 [file 44318_2026_755_MOESM5_ESM.zip › EMBOJ-2025-121050 Figure 3/Fig. 3 western TIF/3C sup_casp1.tif]

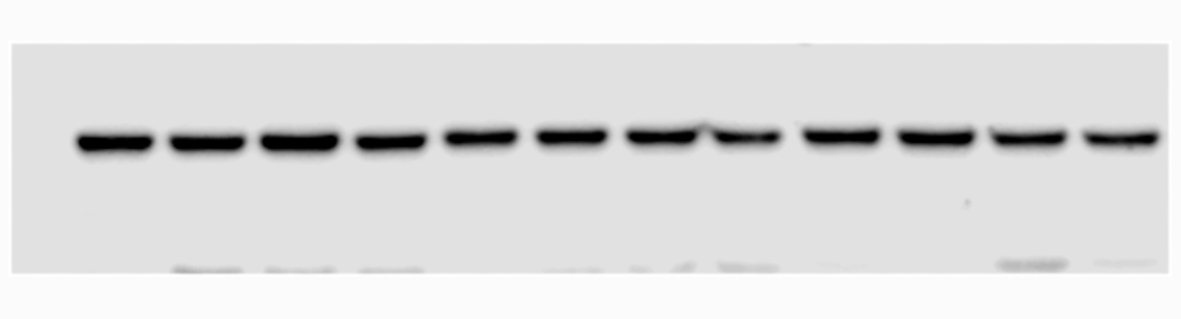

Supplement: Supplementary file 5 — Source data Fig. 3 [file 44318_2026_755_MOESM5_ESM.zip › EMBOJ-2025-121050 Figure 3/Fig. 3 western TIF/3E lys_beta_tubulin.tif]

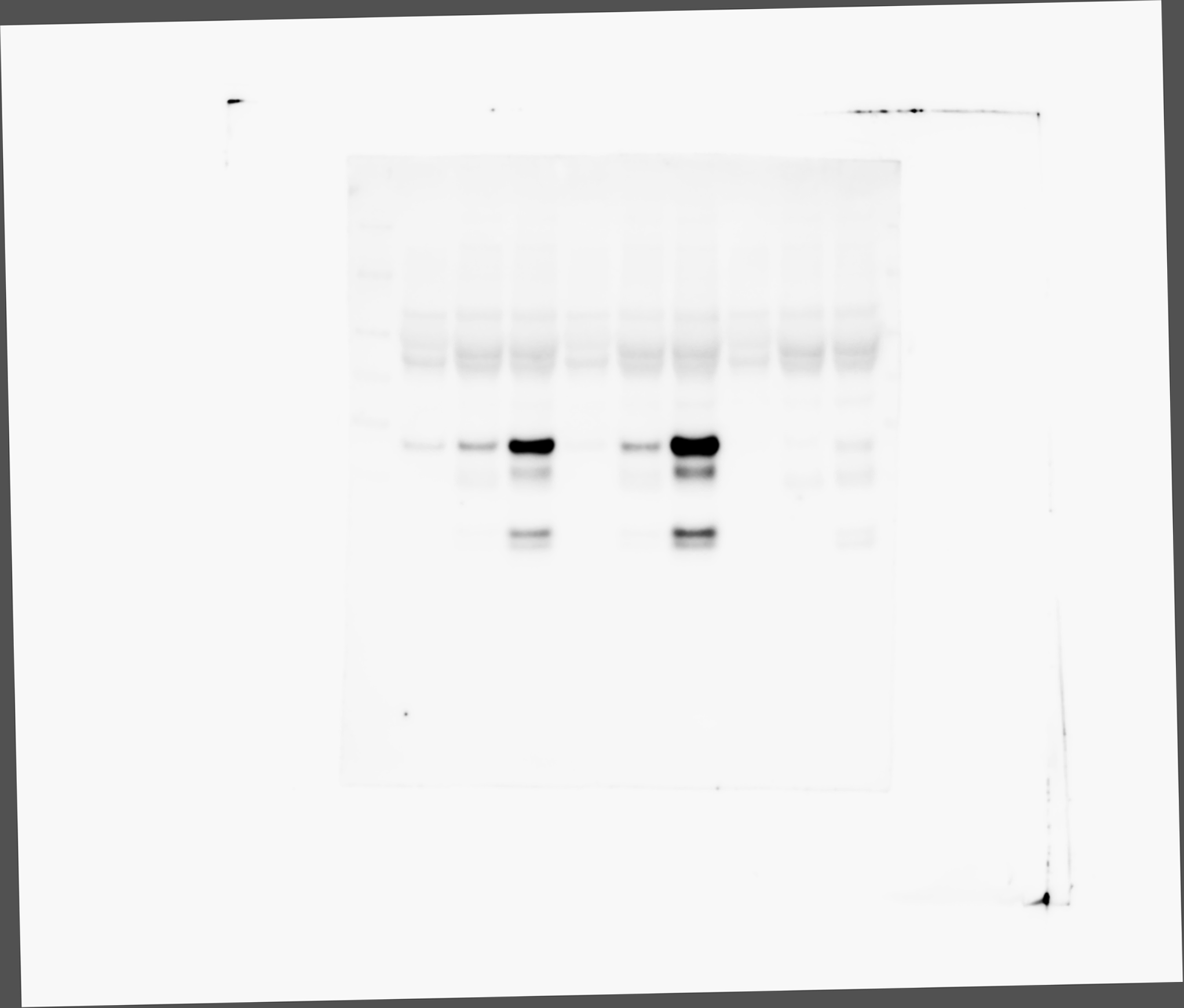

Supplement: Supplementary file 5 — Source data Fig. 3 [file 44318_2026_755_MOESM5_ESM.zip › EMBOJ-2025-121050 Figure 3/Fig. 3 western TIF/3C sup_IL1b.tif]

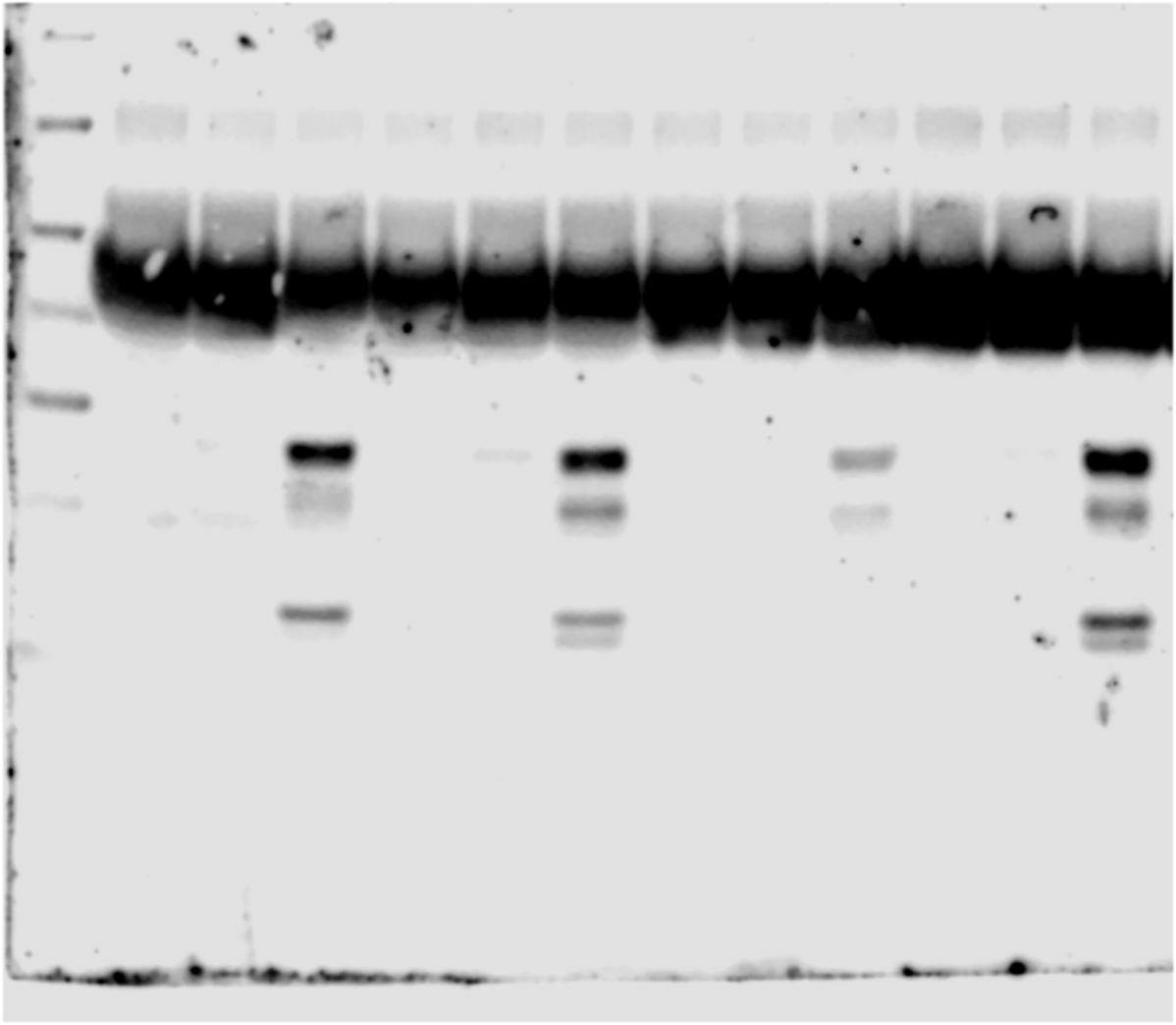

Supplement: Supplementary file 5 — Source data Fig. 3 [file 44318_2026_755_MOESM5_ESM.zip › EMBOJ-2025-121050 Figure 3/Fig. 3 western TIF/3D sup_IL1b.tif]

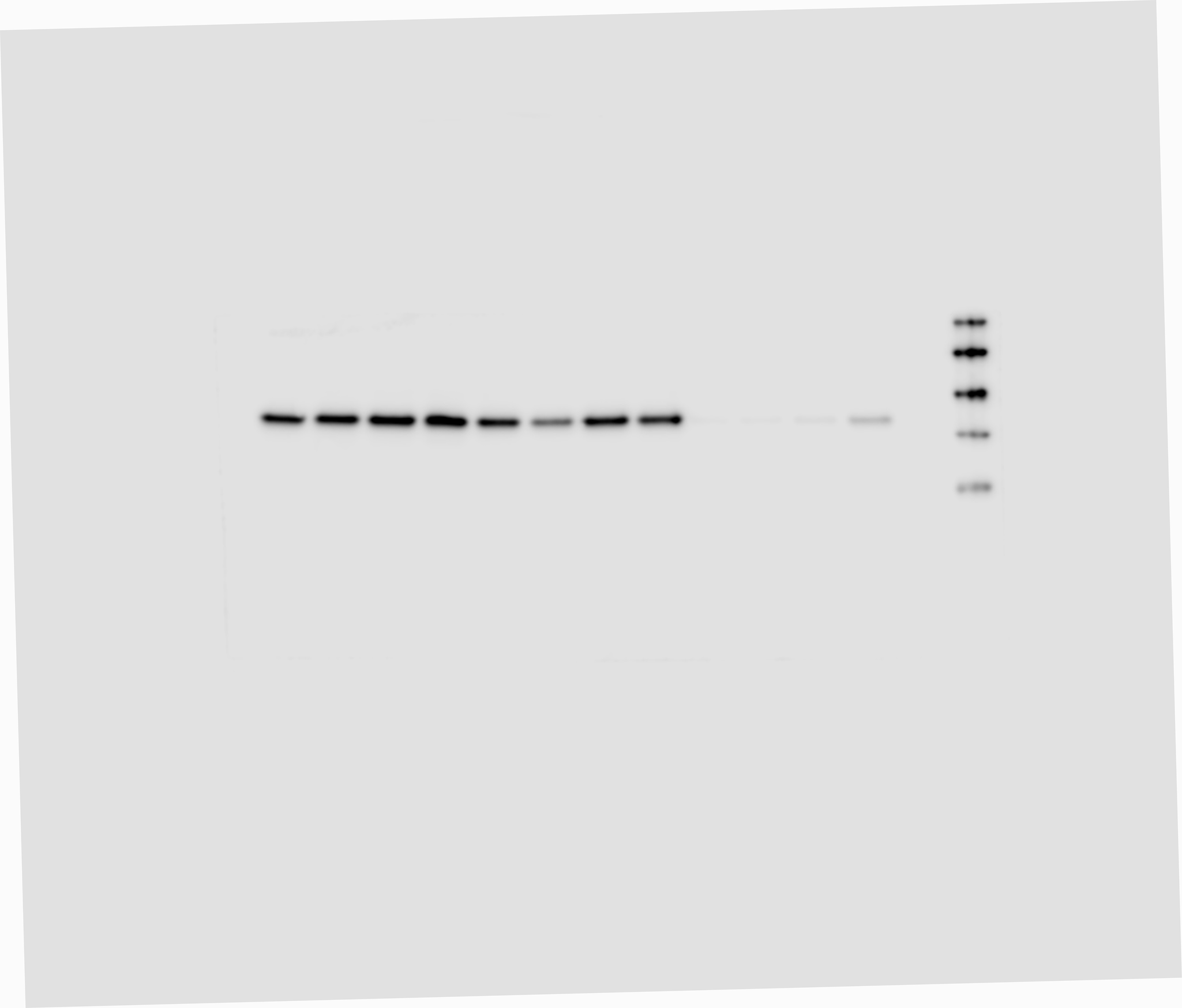

Supplement: Supplementary file 5 — Source data Fig. 3 [file 44318_2026_755_MOESM5_ESM.zip › EMBOJ-2025-121050 Figure 3/Fig. 3 western TIF/3E lys_proIL1.tif]

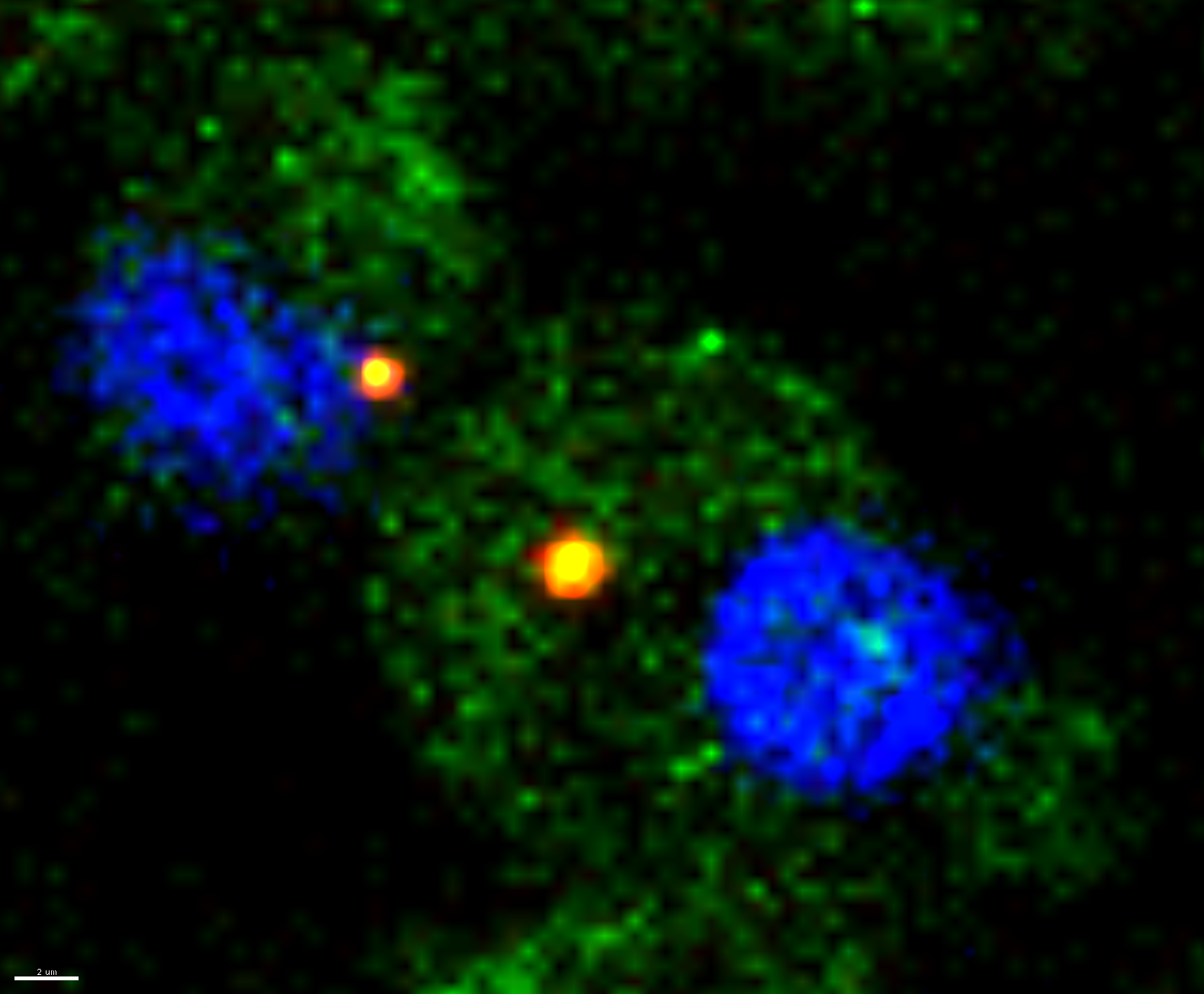

Supplement: Supplementary file 6 — Source data Fig. 4 [file 44318_2026_755_MOESM6_ESM.zip › EMBOJ-2025-121050 Figure 4/4B/Microscopy ASC-NLRP3.tif]

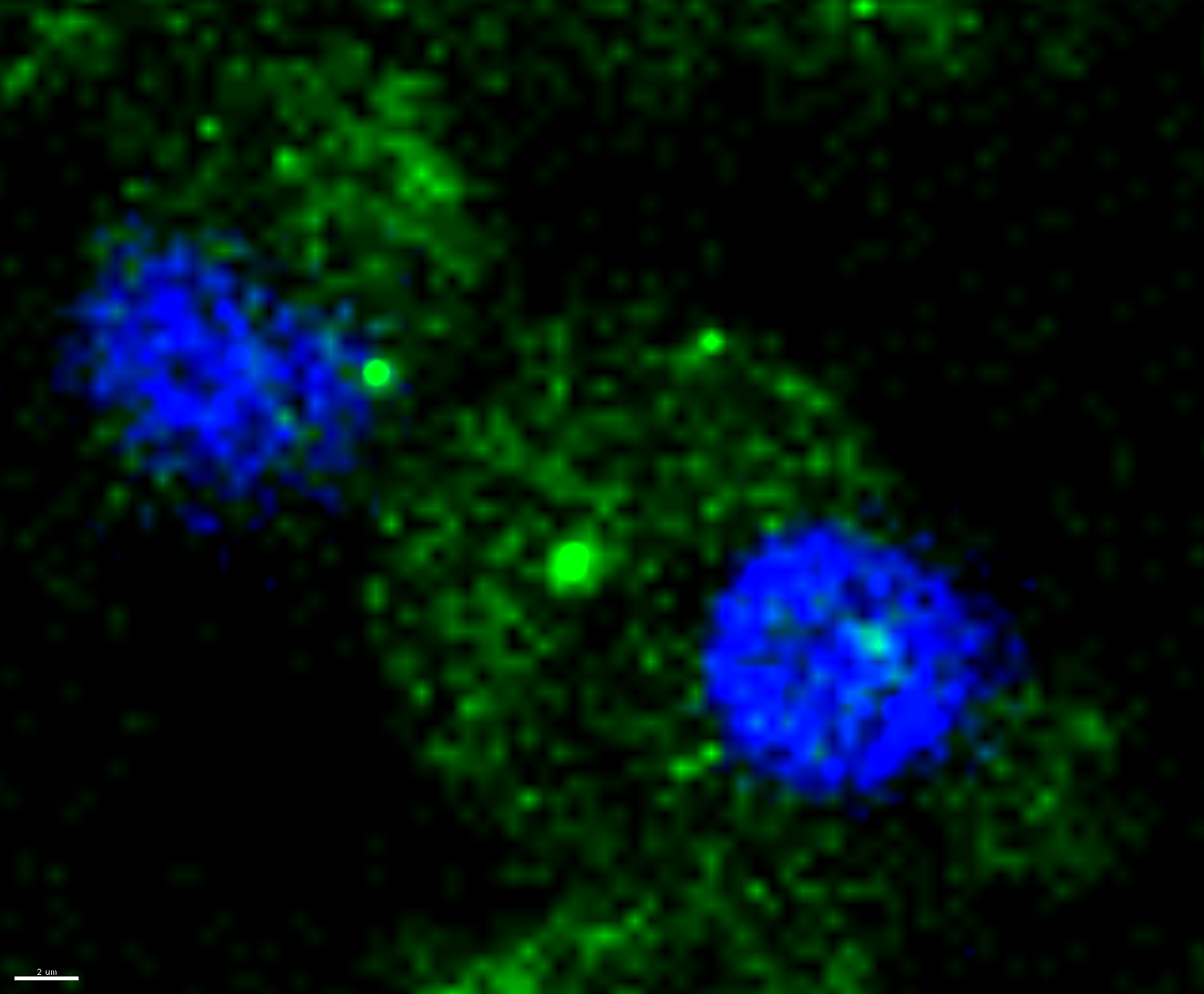

Supplement: Supplementary file 6 — Source data Fig. 4 [file 44318_2026_755_MOESM6_ESM.zip › EMBOJ-2025-121050 Figure 4/4B/Microscopy NLRP3.tif]

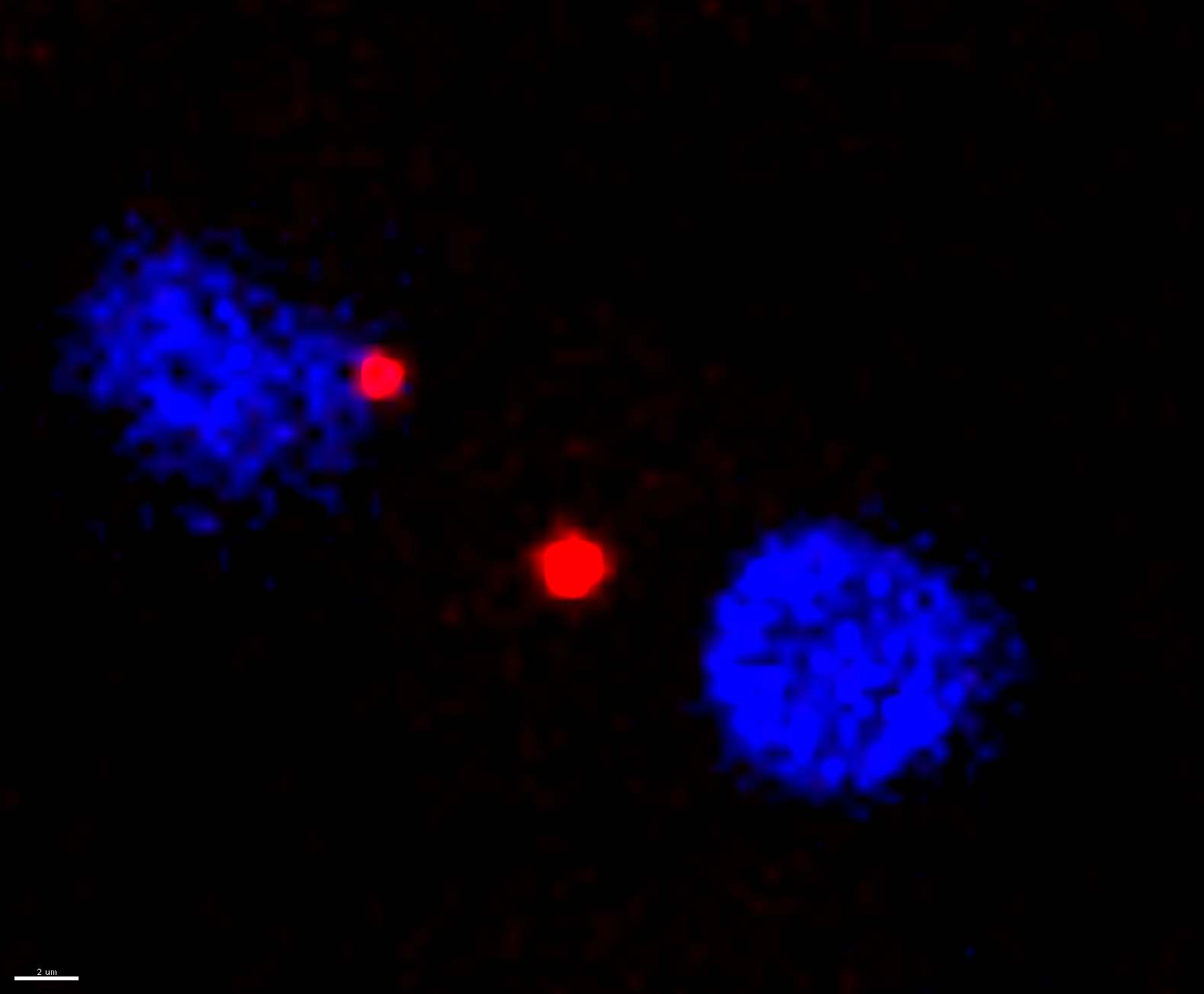

Supplement: Supplementary file 6 — Source data Fig. 4 [file 44318_2026_755_MOESM6_ESM.zip › EMBOJ-2025-121050 Figure 4/4B/Microscopy ASC.tif]

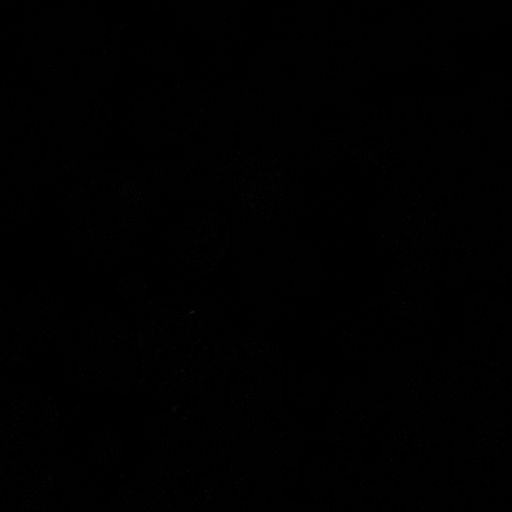

Supplement: Supplementary file 6 — Source data Fig. 4 [file 44318_2026_755_MOESM6_ESM.zip › EMBOJ-2025-121050 Figure 4/4A/Microscopy Z-stack.tif]

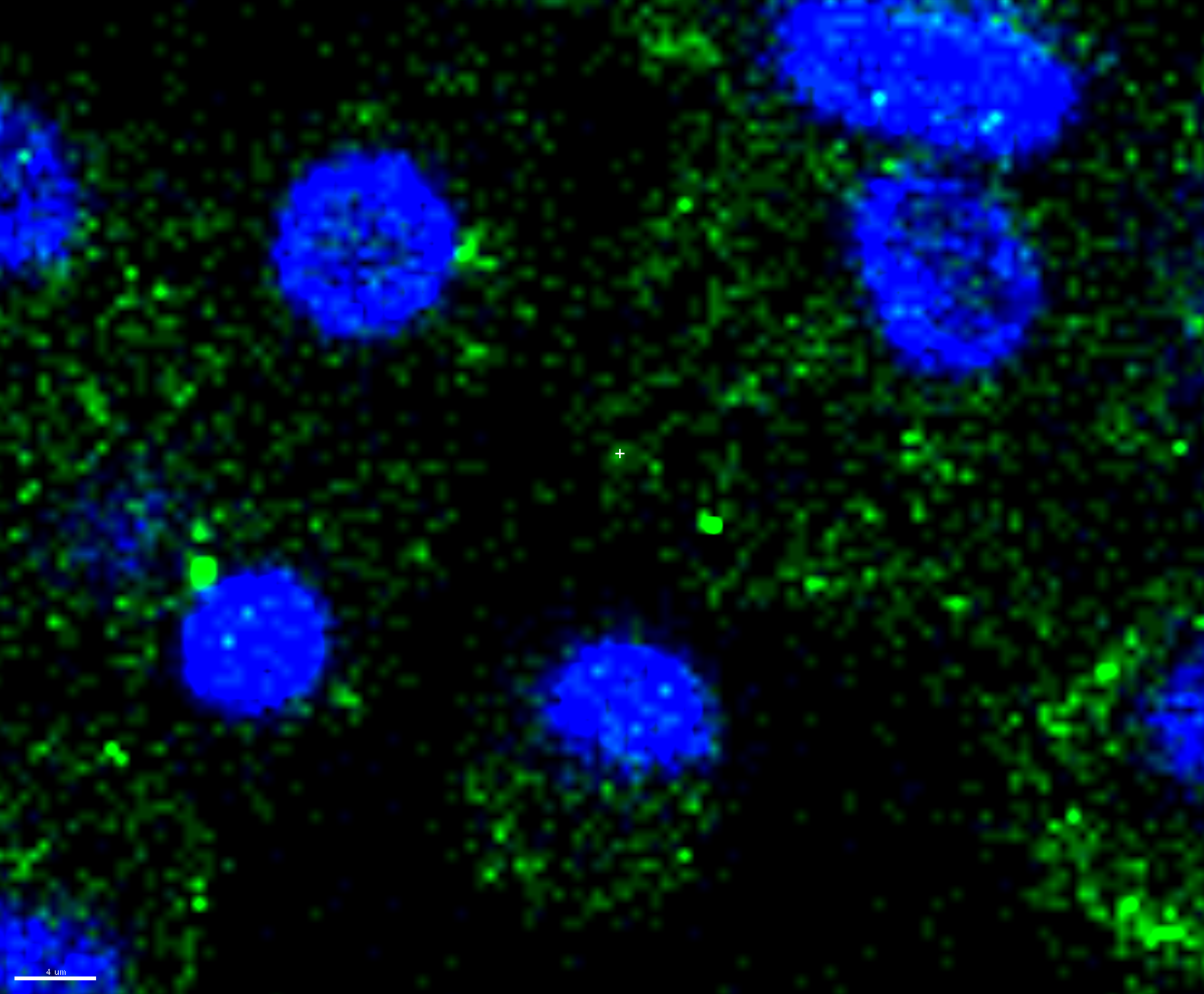

Supplement: Supplementary file 6 — Source data Fig. 4 [file 44318_2026_755_MOESM6_ESM.zip › EMBOJ-2025-121050 Figure 4/4A/Microscopy FIP2.tif]

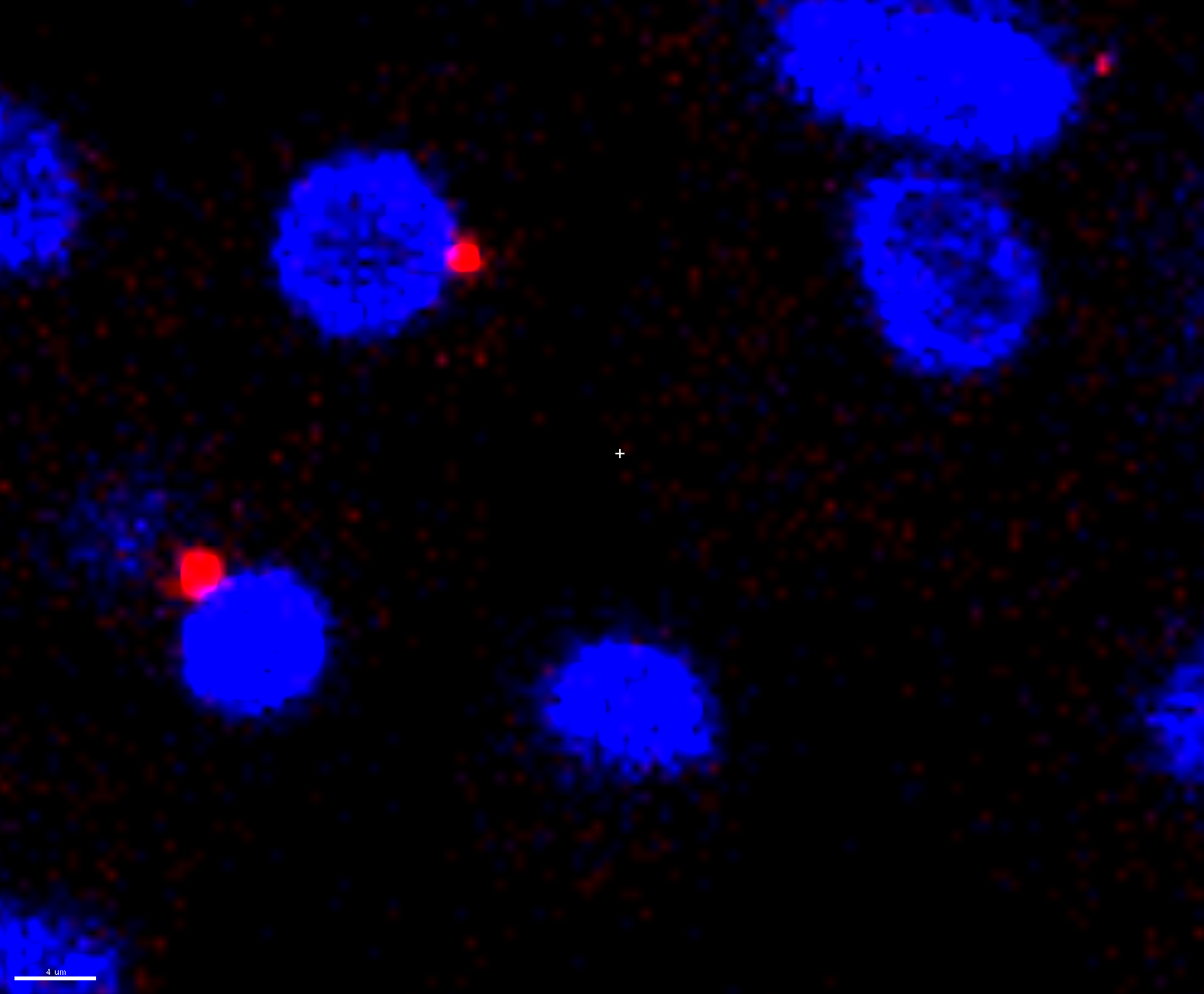

Supplement: Supplementary file 6 — Source data Fig. 4 [file 44318_2026_755_MOESM6_ESM.zip › EMBOJ-2025-121050 Figure 4/4A/Microscopy ASC.tif]

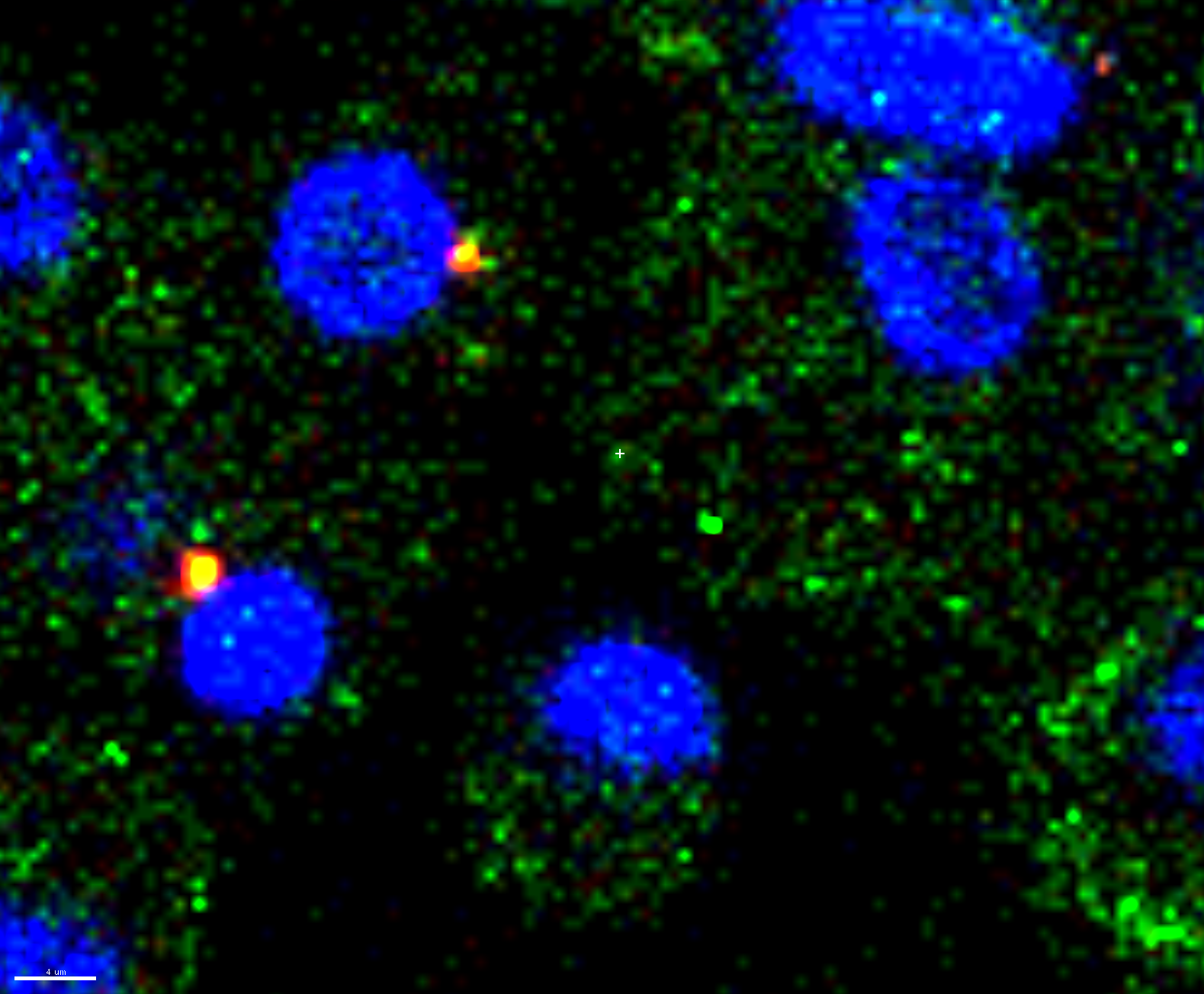

Supplement: Supplementary file 6 — Source data Fig. 4 [file 44318_2026_755_MOESM6_ESM.zip › EMBOJ-2025-121050 Figure 4/4A/Microscopy ASC-FIP2.tif]

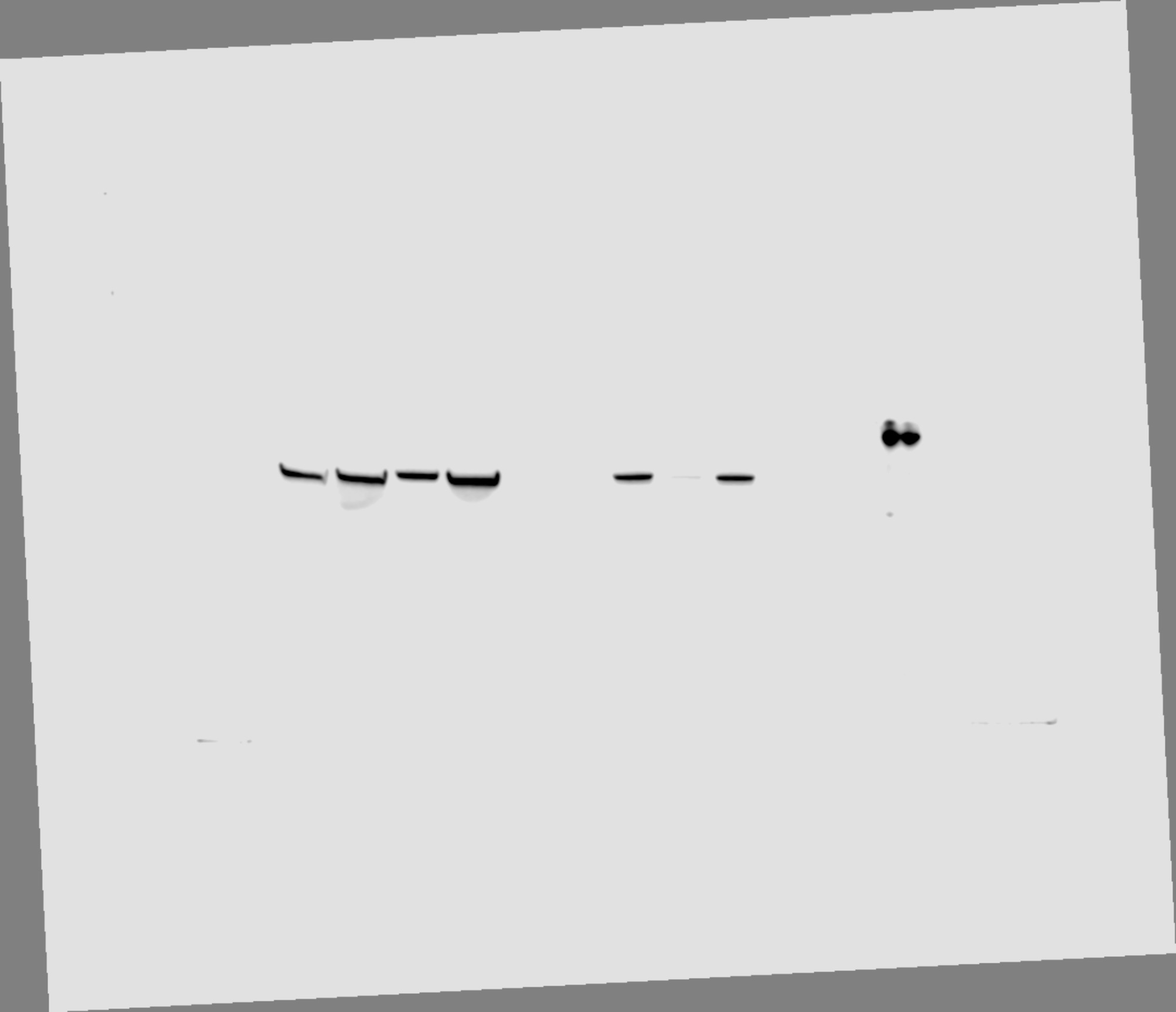

Supplement: Supplementary file 7 — Source data Fig. 5 [file 44318_2026_755_MOESM7_ESM.zip › EMBOJ-2025-121050 Figure 5/Western TIF/5G/5G IP EGFP_NLRP3 new.tif]

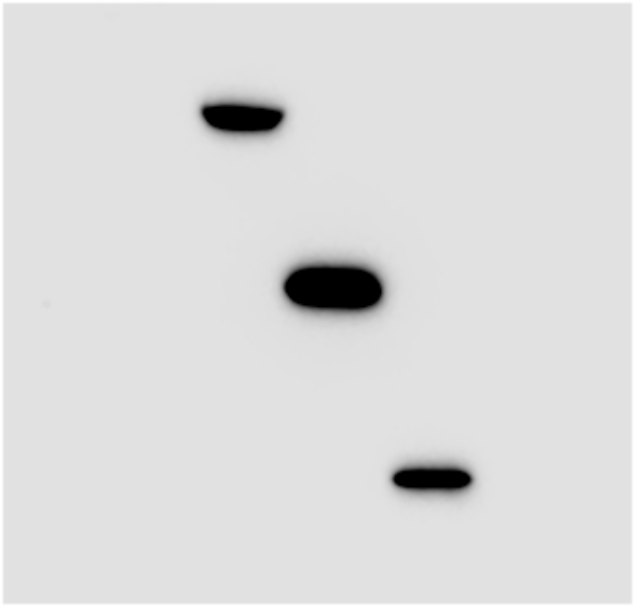

Supplement: Supplementary file 7 — Source data Fig. 5 [file 44318_2026_755_MOESM7_ESM.zip › EMBOJ-2025-121050 Figure 5/Western TIF/5G/5G WCL Flag_FIP2.tif]

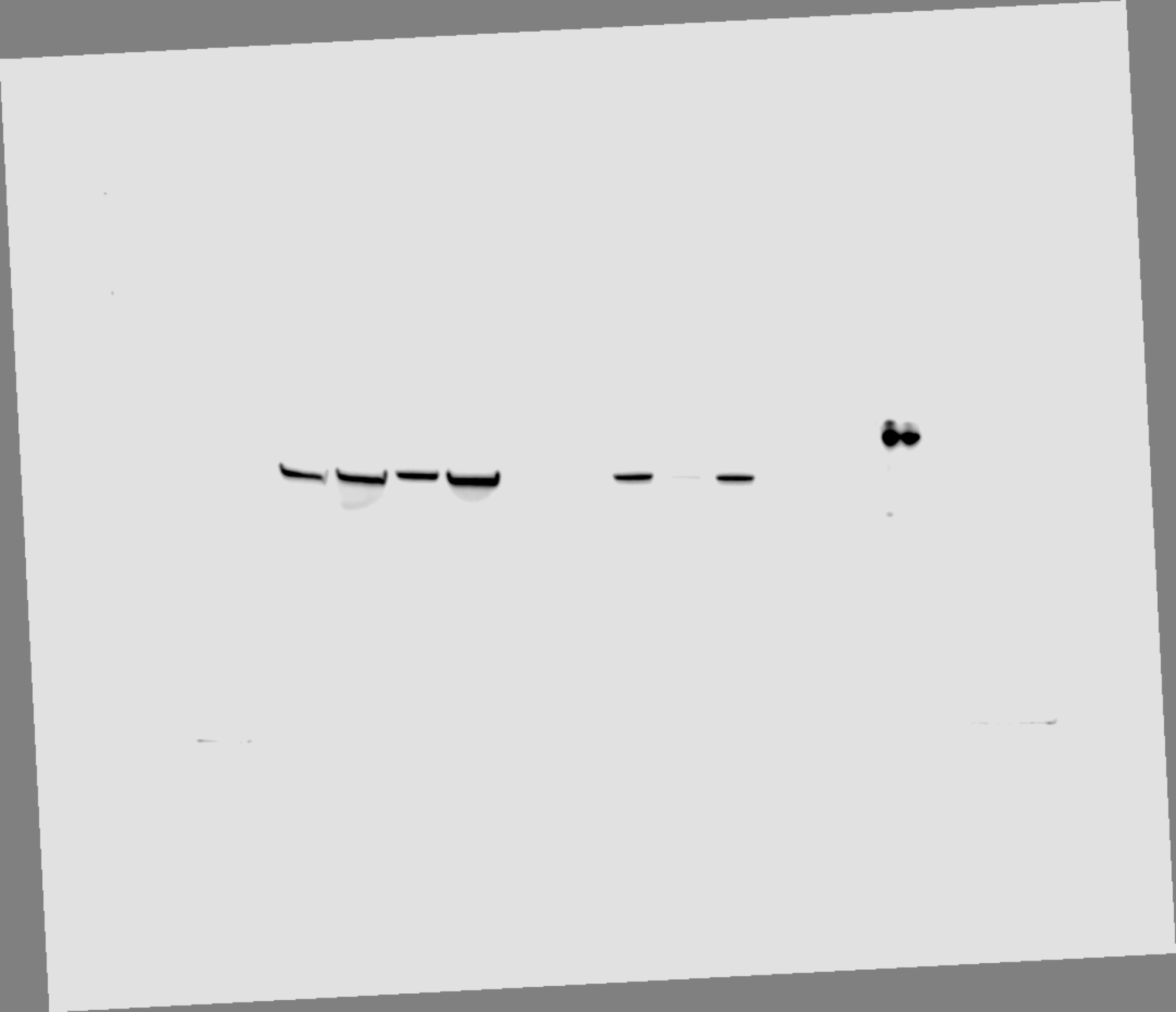

Supplement: Supplementary file 7 — Source data Fig. 5 [file 44318_2026_755_MOESM7_ESM.zip › EMBOJ-2025-121050 Figure 5/Western TIF/5G/5G IP EGFP-NLRP3.tif]

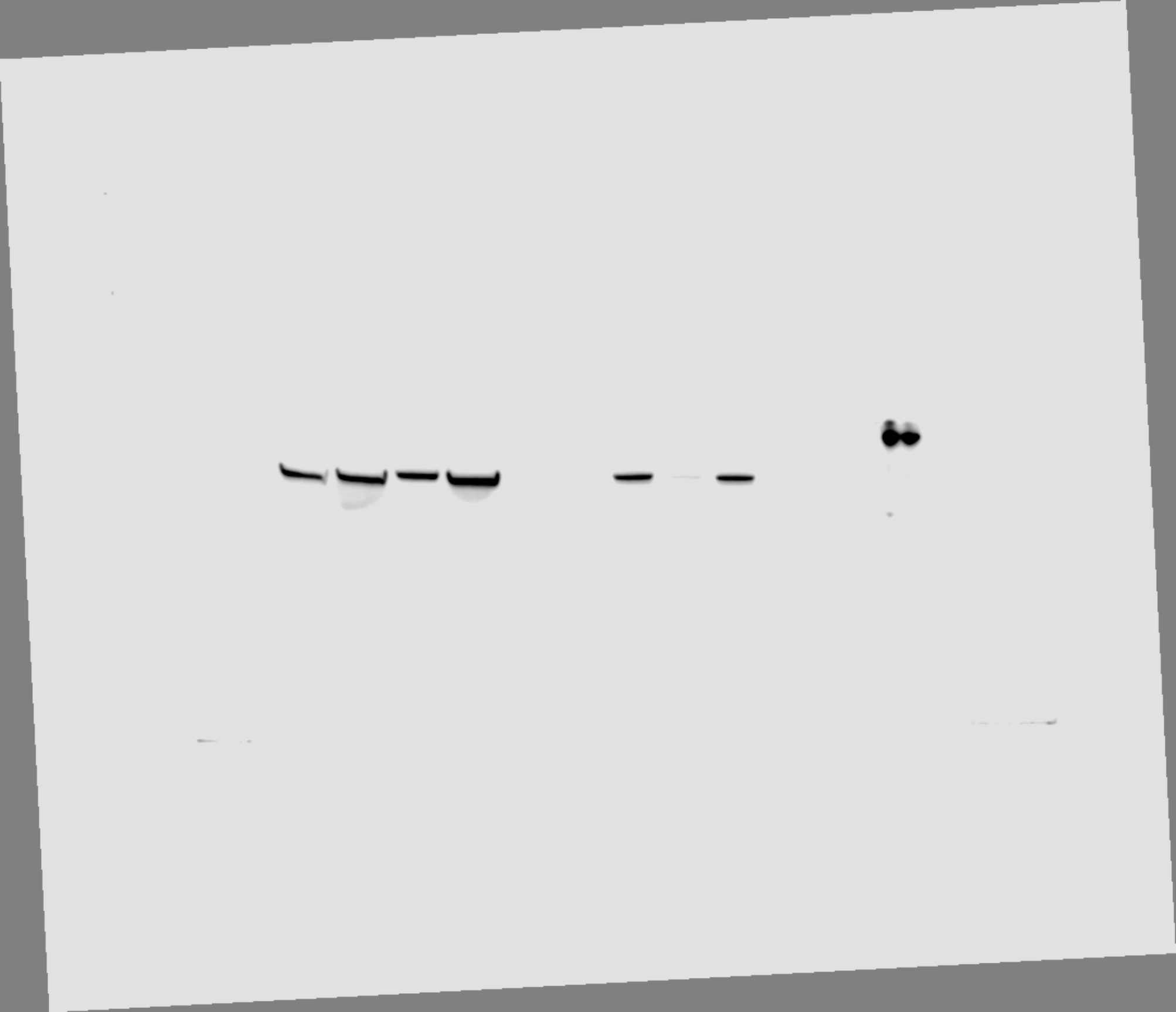

Supplement: Supplementary file 7 — Source data Fig. 5 [file 44318_2026_755_MOESM7_ESM.zip › EMBOJ-2025-121050 Figure 5/Western TIF/5G/5G WCL EGFP-NLRP3.tif]

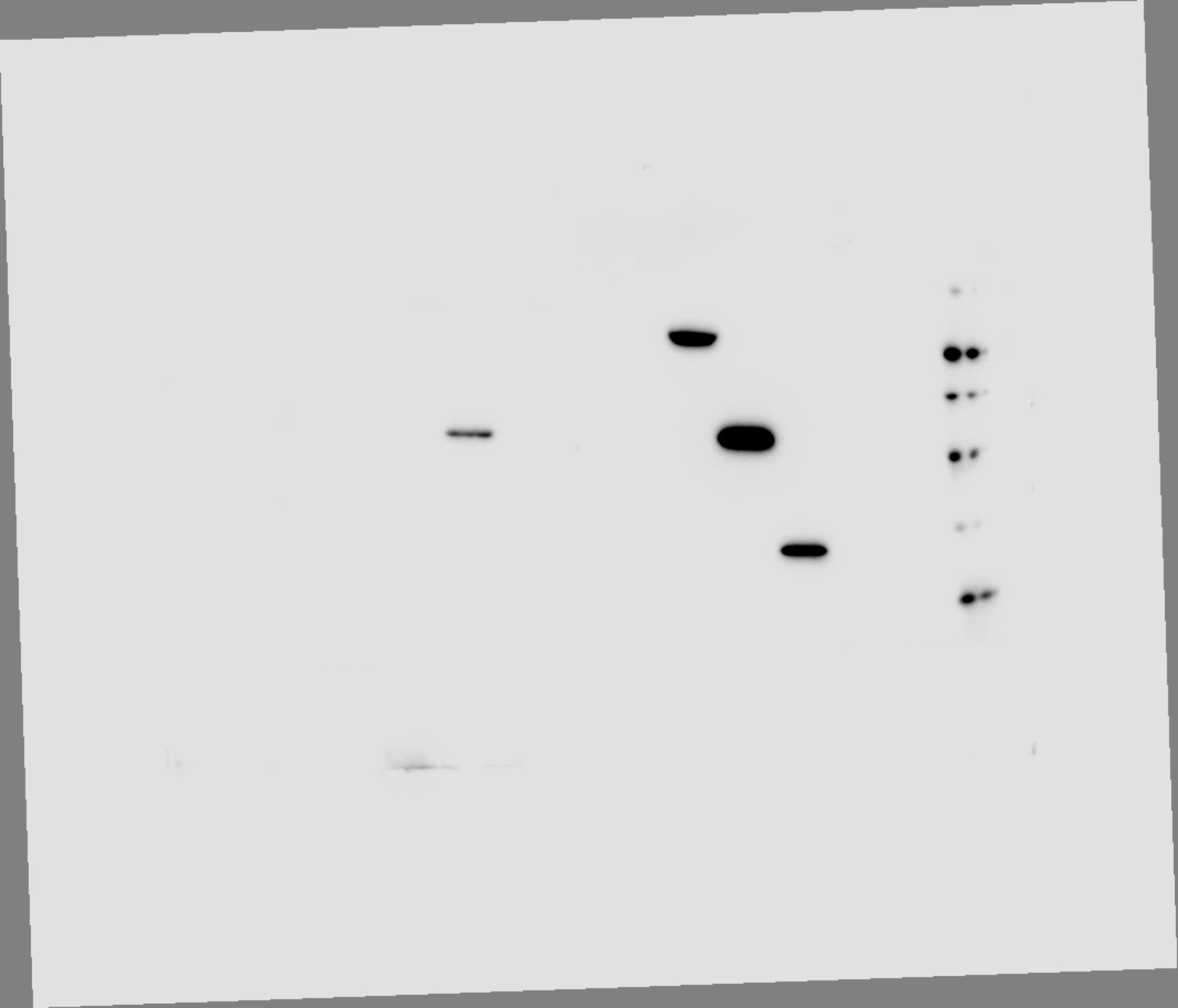

Supplement: Supplementary file 7 — Source data Fig. 5 [file 44318_2026_755_MOESM7_ESM.zip › EMBOJ-2025-121050 Figure 5/Western TIF/5G/5G IP Flag-FIP2.tif]

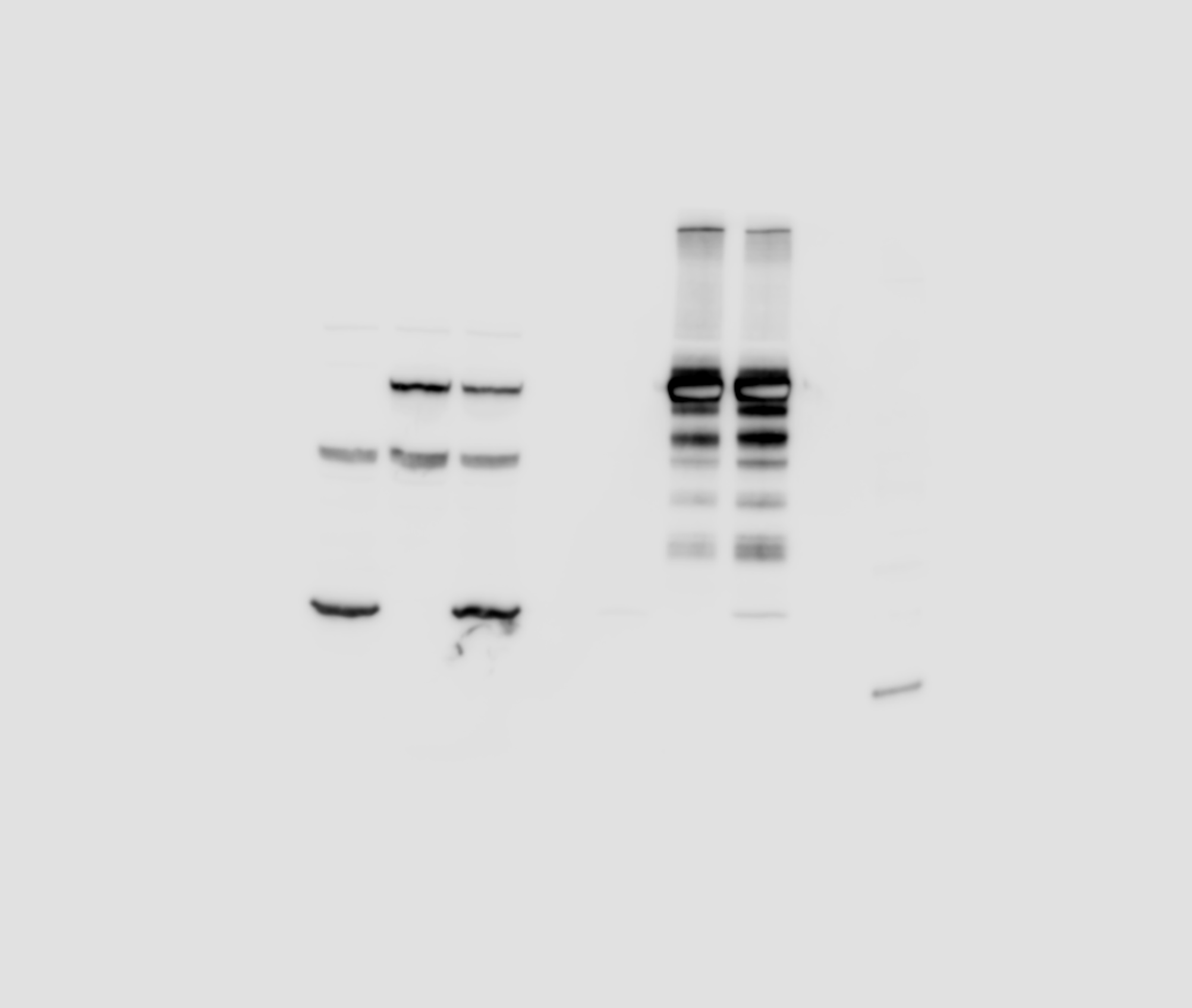

Supplement: Supplementary file 7 — Source data Fig. 5 [file 44318_2026_755_MOESM7_ESM.zip › EMBOJ-2025-121050 Figure 5/Western TIF/5A/5A IP Flag-NLRP3.tif]

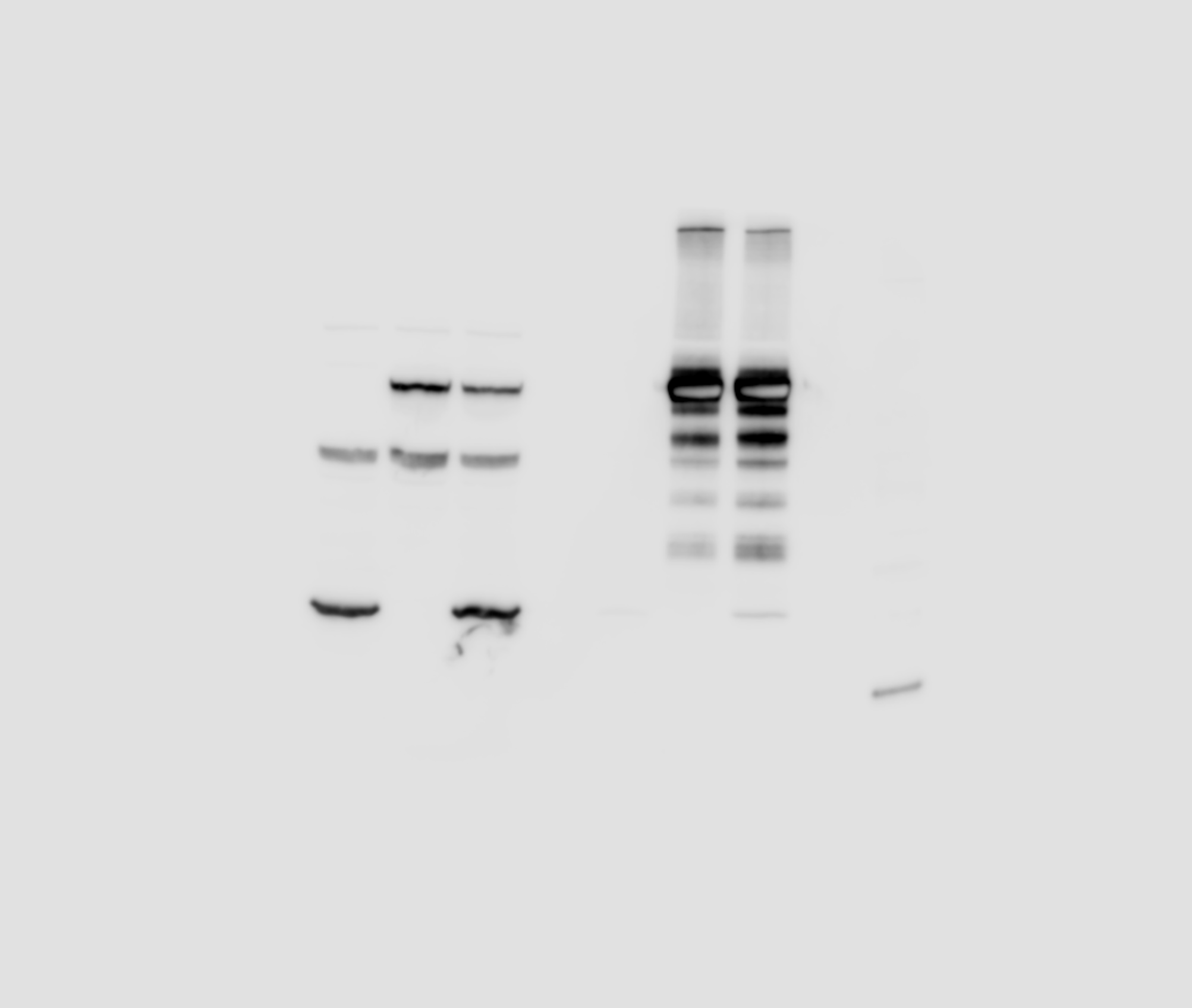

Supplement: Supplementary file 7 — Source data Fig. 5 [file 44318_2026_755_MOESM7_ESM.zip › EMBOJ-2025-121050 Figure 5/Western TIF/5A/5A WCL Flag-NLRP3.tif]

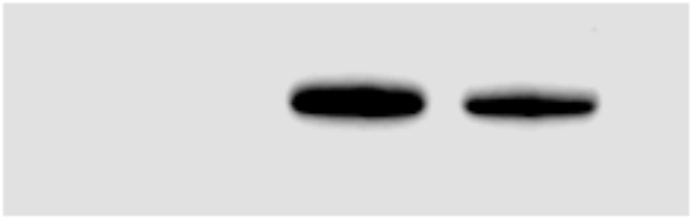

Supplement: Supplementary file 7 — Source data Fig. 5 [file 44318_2026_755_MOESM7_ESM.zip › EMBOJ-2025-121050 Figure 5/Western TIF/5A/5A IP FIP2-GFP.tif]

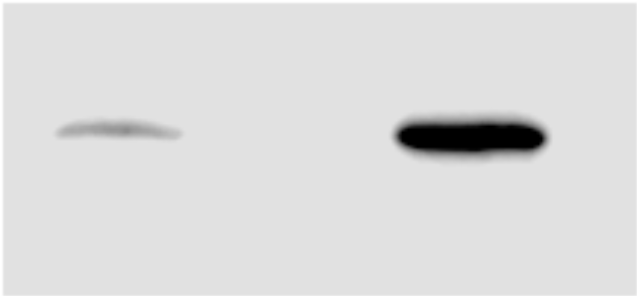

Supplement: Supplementary file 7 — Source data Fig. 5 [file 44318_2026_755_MOESM7_ESM.zip › EMBOJ-2025-121050 Figure 5/Western TIF/5A/5A IP ECFP-Rab11.tif]

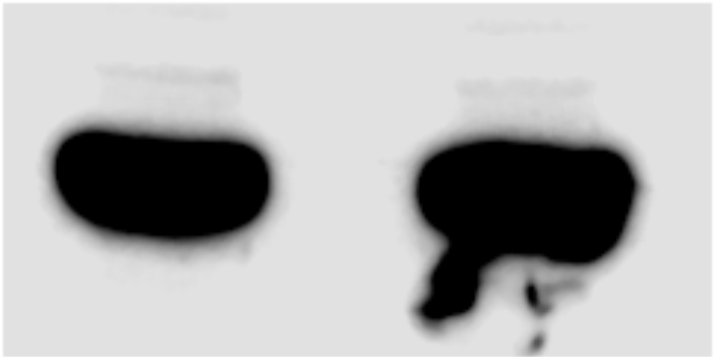

Supplement: Supplementary file 7 — Source data Fig. 5 [file 44318_2026_755_MOESM7_ESM.zip › EMBOJ-2025-121050 Figure 5/Western TIF/5A/5A WCL ECFP-Rab11.tif]

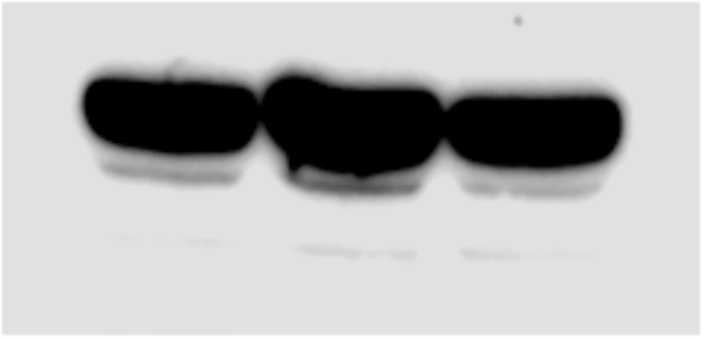

Supplement: Supplementary file 7 — Source data Fig. 5 [file 44318_2026_755_MOESM7_ESM.zip › EMBOJ-2025-121050 Figure 5/Western TIF/5A/5A WCL FIP2-GFP.tif]

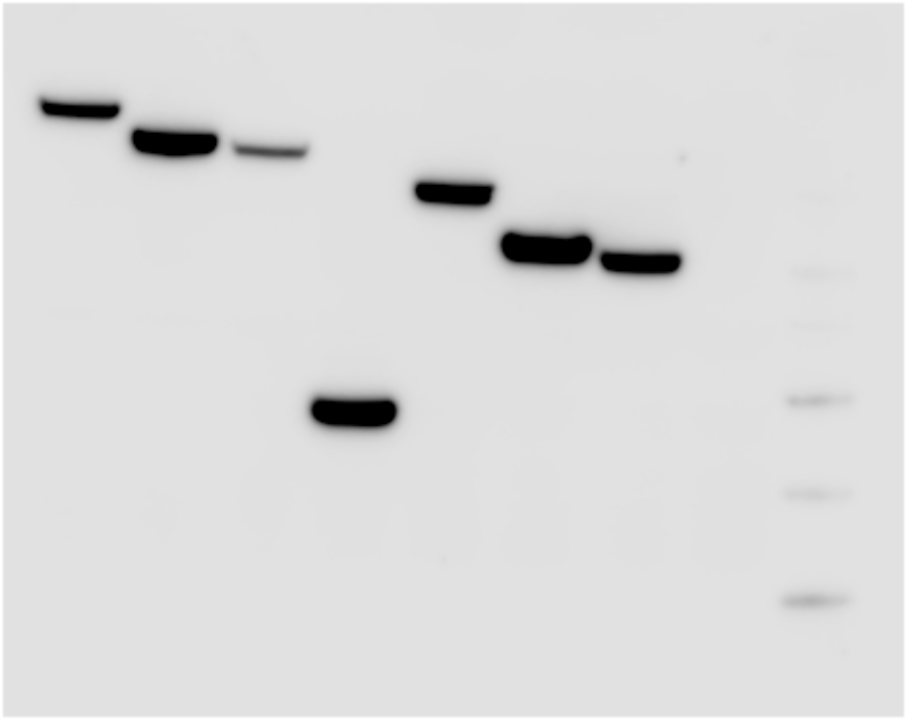

Supplement: Supplementary file 7 — Source data Fig. 5 [file 44318_2026_755_MOESM7_ESM.zip › EMBOJ-2025-121050 Figure 5/Western TIF/5C/5C WCL Flag_NLRP3 empty vector no 8.tif]

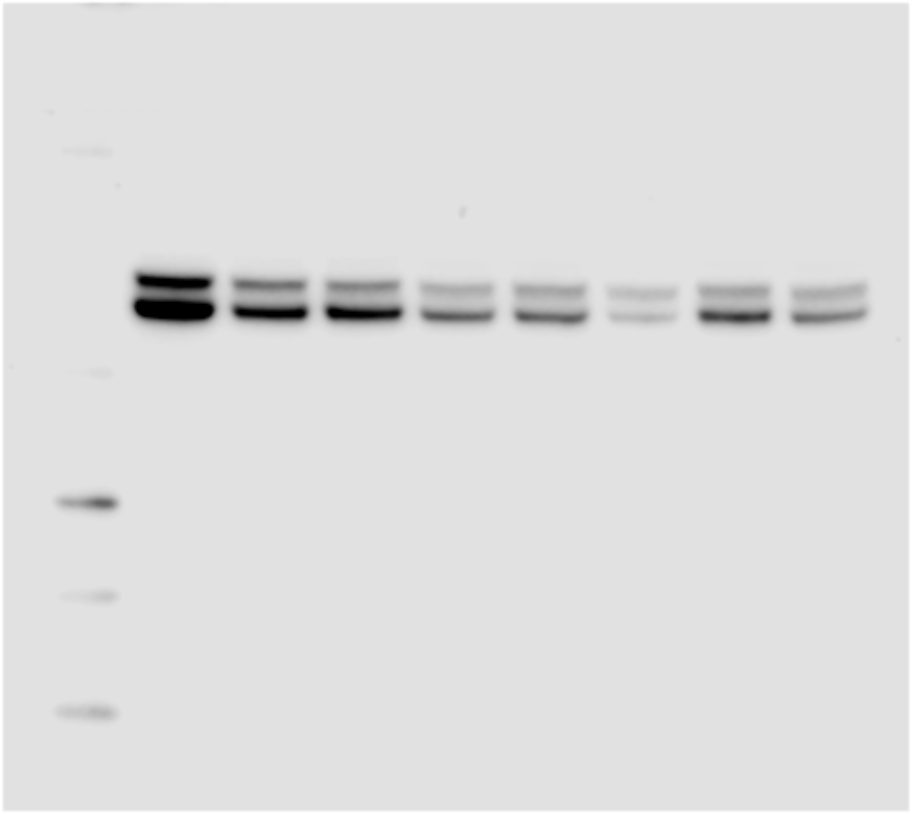

Supplement: Supplementary file 7 — Source data Fig. 5 [file 44318_2026_755_MOESM7_ESM.zip › EMBOJ-2025-121050 Figure 5/Western TIF/5C/5C WCL EGFP_FIP2 empty vector no 8.tif]

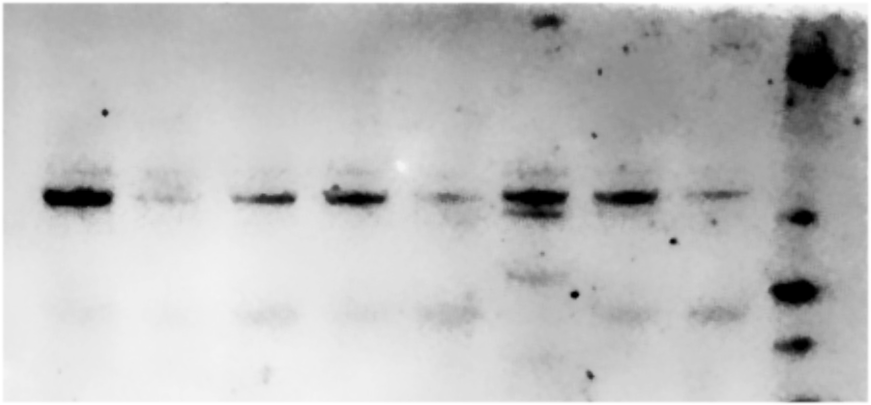

Supplement: Supplementary file 7 — Source data Fig. 5 [file 44318_2026_755_MOESM7_ESM.zip › EMBOJ-2025-121050 Figure 5/Western TIF/5C/5C IP EGFP_FIP2 empty vector number 8.tif]

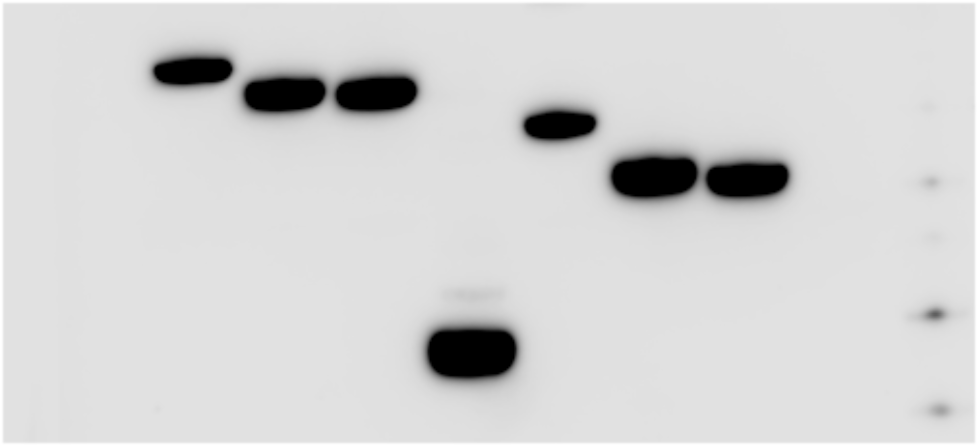

Supplement: Supplementary file 7 — Source data Fig. 5 [file 44318_2026_755_MOESM7_ESM.zip › EMBOJ-2025-121050 Figure 5/Western TIF/5C/5C IP Flag_NLRP3 empty vector no 8.tif]

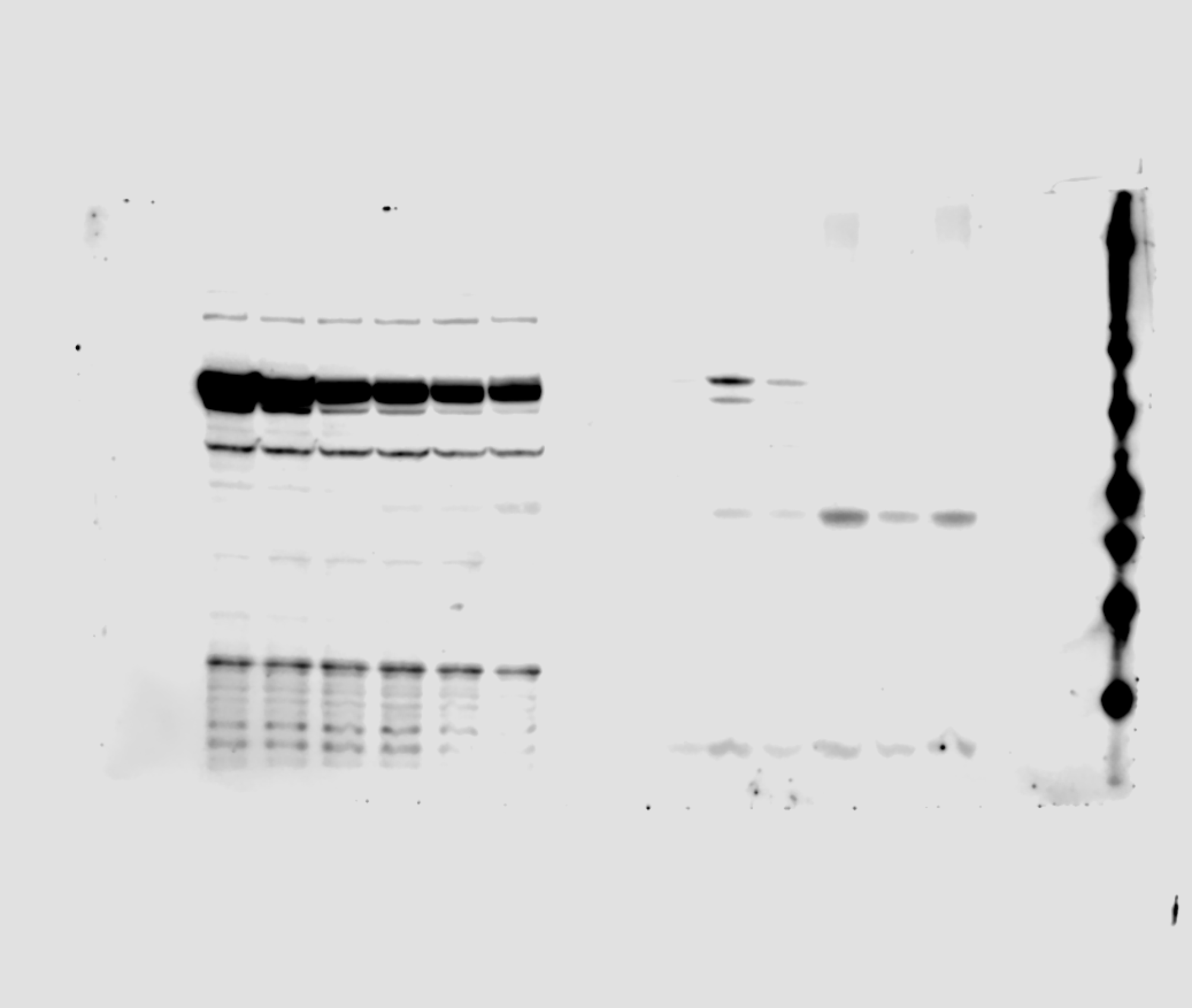

Supplement: Supplementary file 7 — Source data Fig. 5 [file 44318_2026_755_MOESM7_ESM.zip › EMBOJ-2025-121050 Figure 5/Western TIF/5E/5E IP EGFP-FIP2.tif]

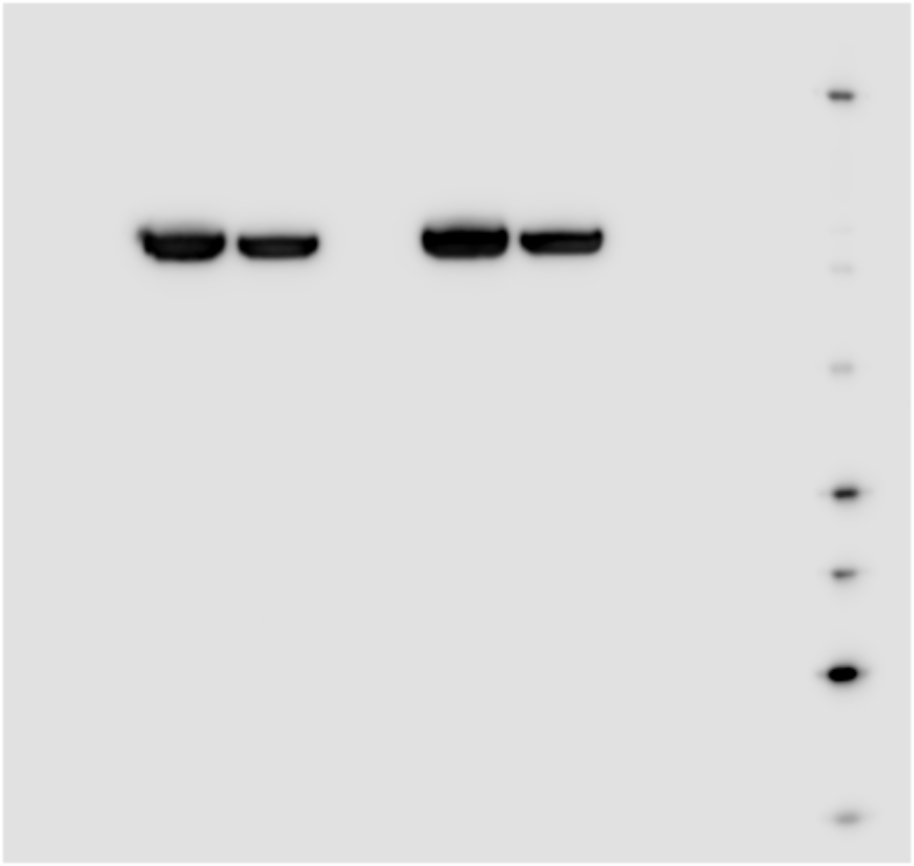

Supplement: Supplementary file 7 — Source data Fig. 5 [file 44318_2026_755_MOESM7_ESM.zip › EMBOJ-2025-121050 Figure 5/Western TIF/5E/5E IP Flag-NLRP3.tif]

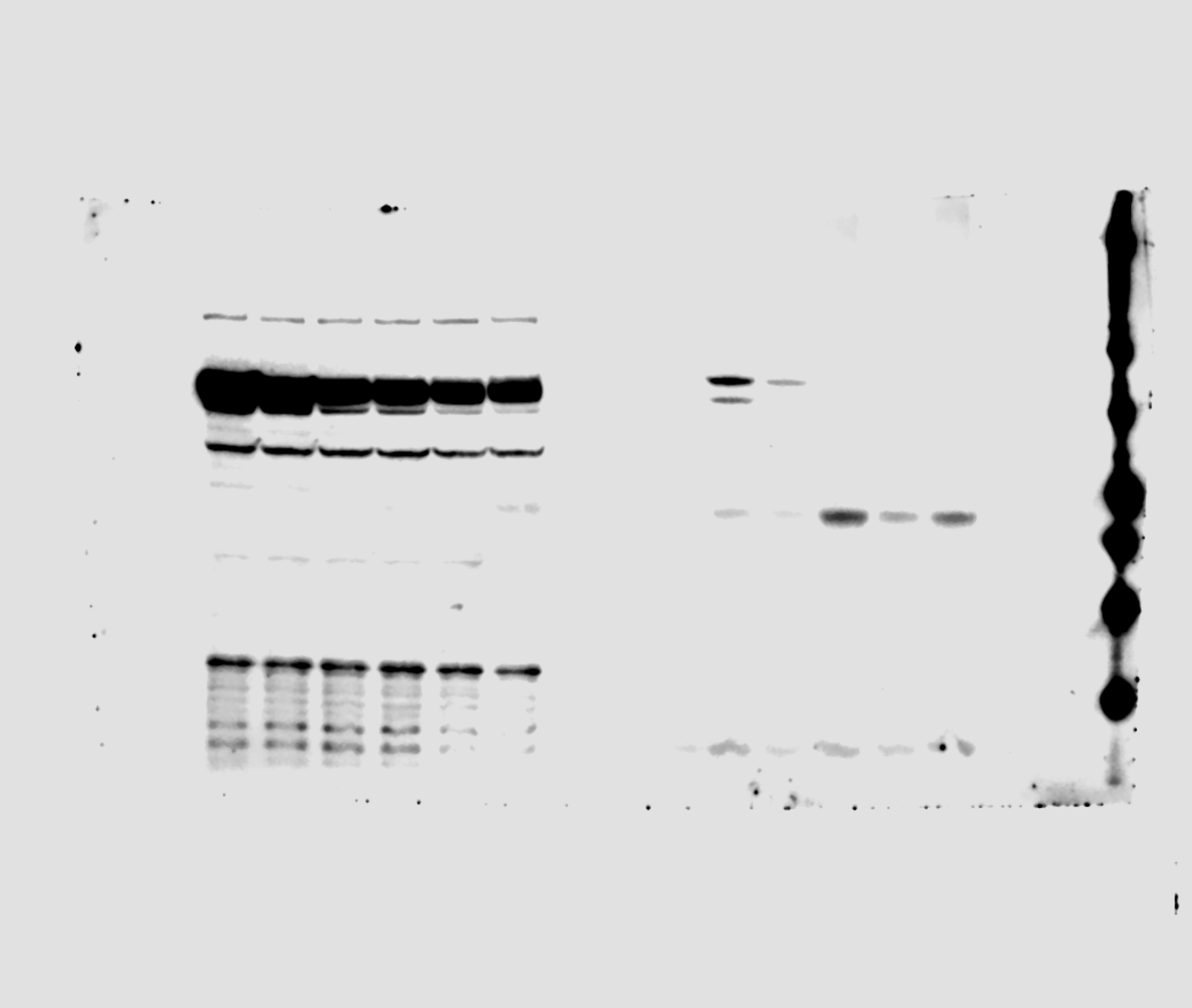

Supplement: Supplementary file 7 — Source data Fig. 5 [file 44318_2026_755_MOESM7_ESM.zip › EMBOJ-2025-121050 Figure 5/Western TIF/5E/5E WCL FIP2-GFP II.tif]

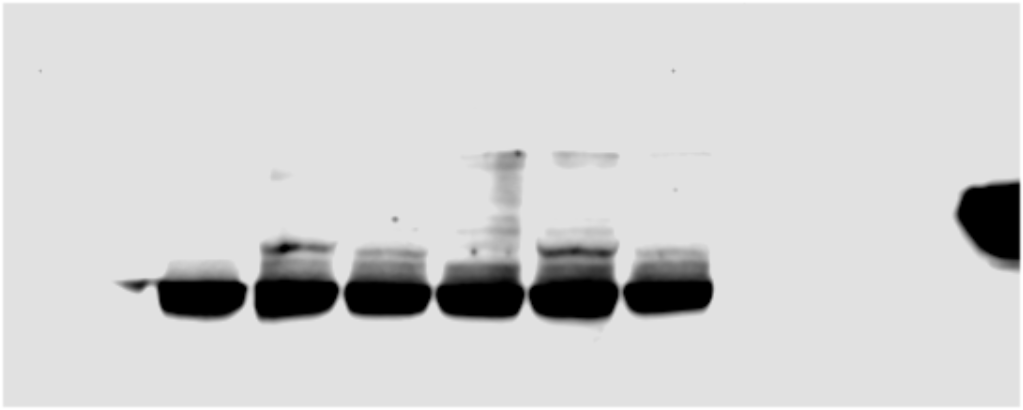

Supplement: Supplementary file 7 — Source data Fig. 5 [file 44318_2026_755_MOESM7_ESM.zip › EMBOJ-2025-121050 Figure 5/Western TIF/5E/5E WCL Flag-NLRP3.tif]

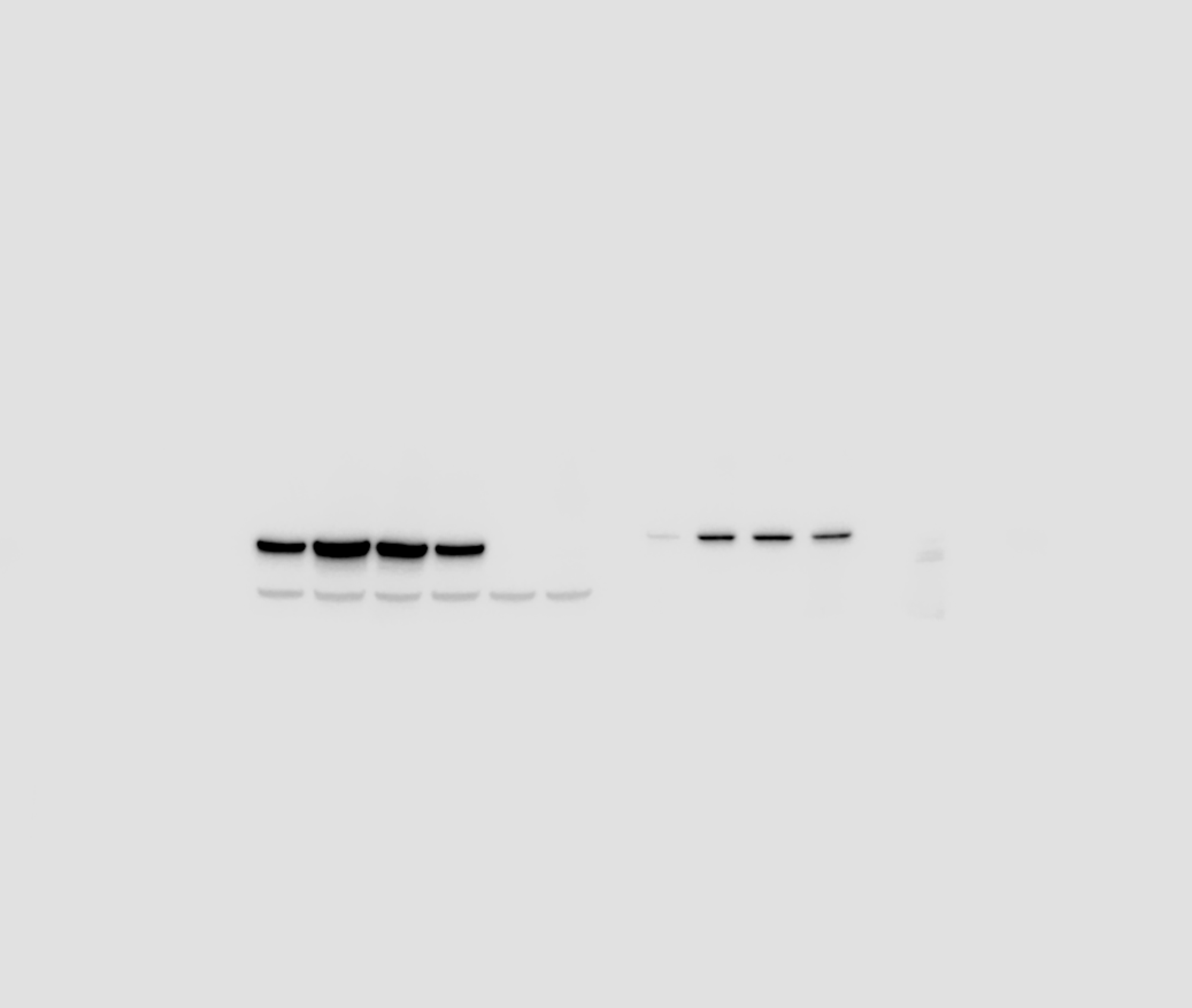

Supplement: Supplementary file 7 — Source data Fig. 5 [file 44318_2026_755_MOESM7_ESM.zip › EMBOJ-2025-121050 Figure 5/Western TIF/5B/5B IP EGFP-NLRP3.tif]

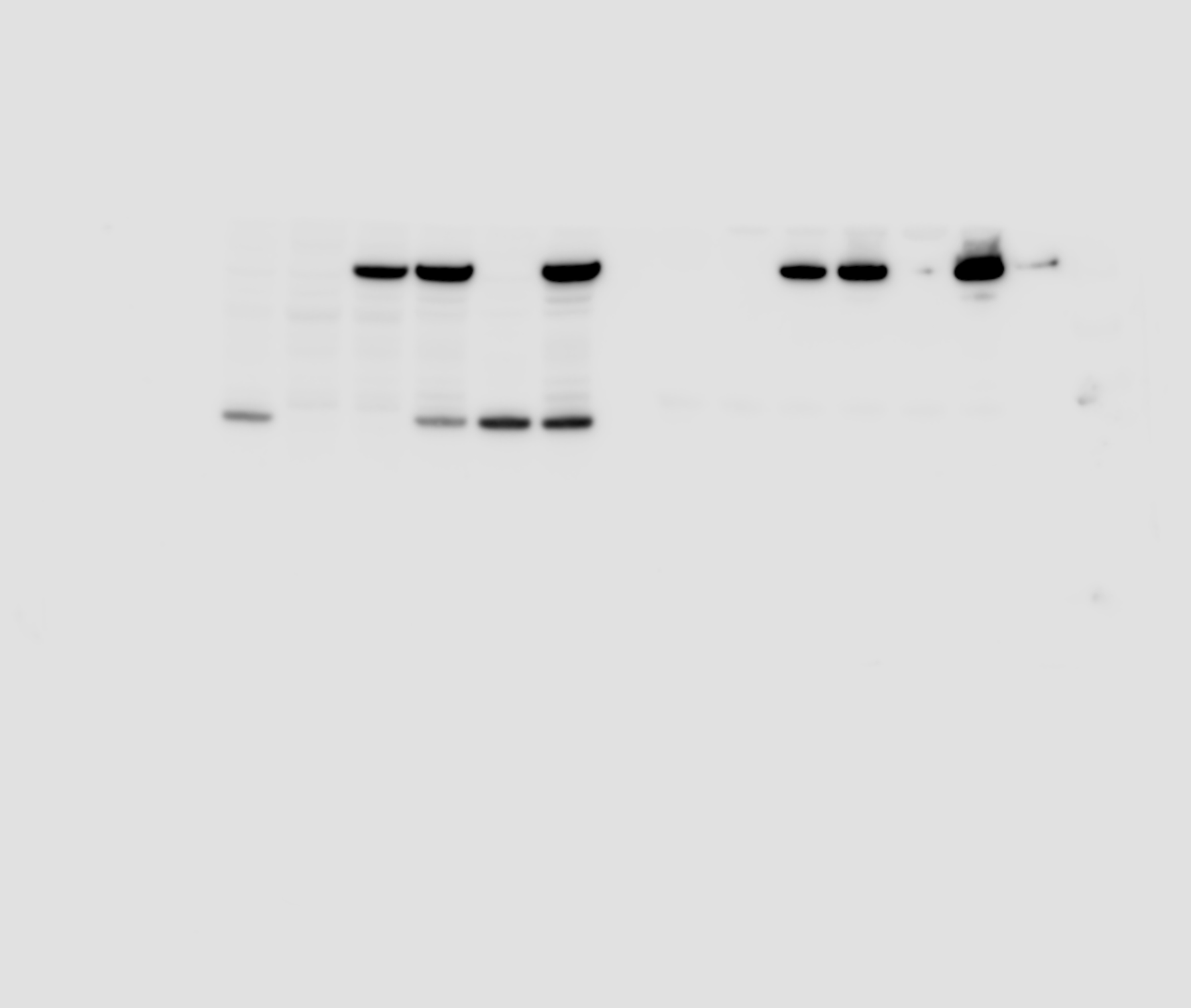

Supplement: Supplementary file 7 — Source data Fig. 5 [file 44318_2026_755_MOESM7_ESM.zip › EMBOJ-2025-121050 Figure 5/Western TIF/5B/5B WCL ECFP-Rab11.tif]

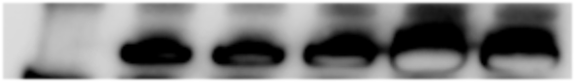

Supplement: Supplementary file 7 — Source data Fig. 5 [file 44318_2026_755_MOESM7_ESM.zip › EMBOJ-2025-121050 Figure 5/Western TIF/5B/5B IP Flag-FIP2.tif]

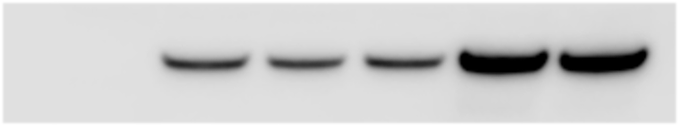

Supplement: Supplementary file 7 — Source data Fig. 5 [file 44318_2026_755_MOESM7_ESM.zip › EMBOJ-2025-121050 Figure 5/Western TIF/5B/5B WCL Flag-FIP2.tif]

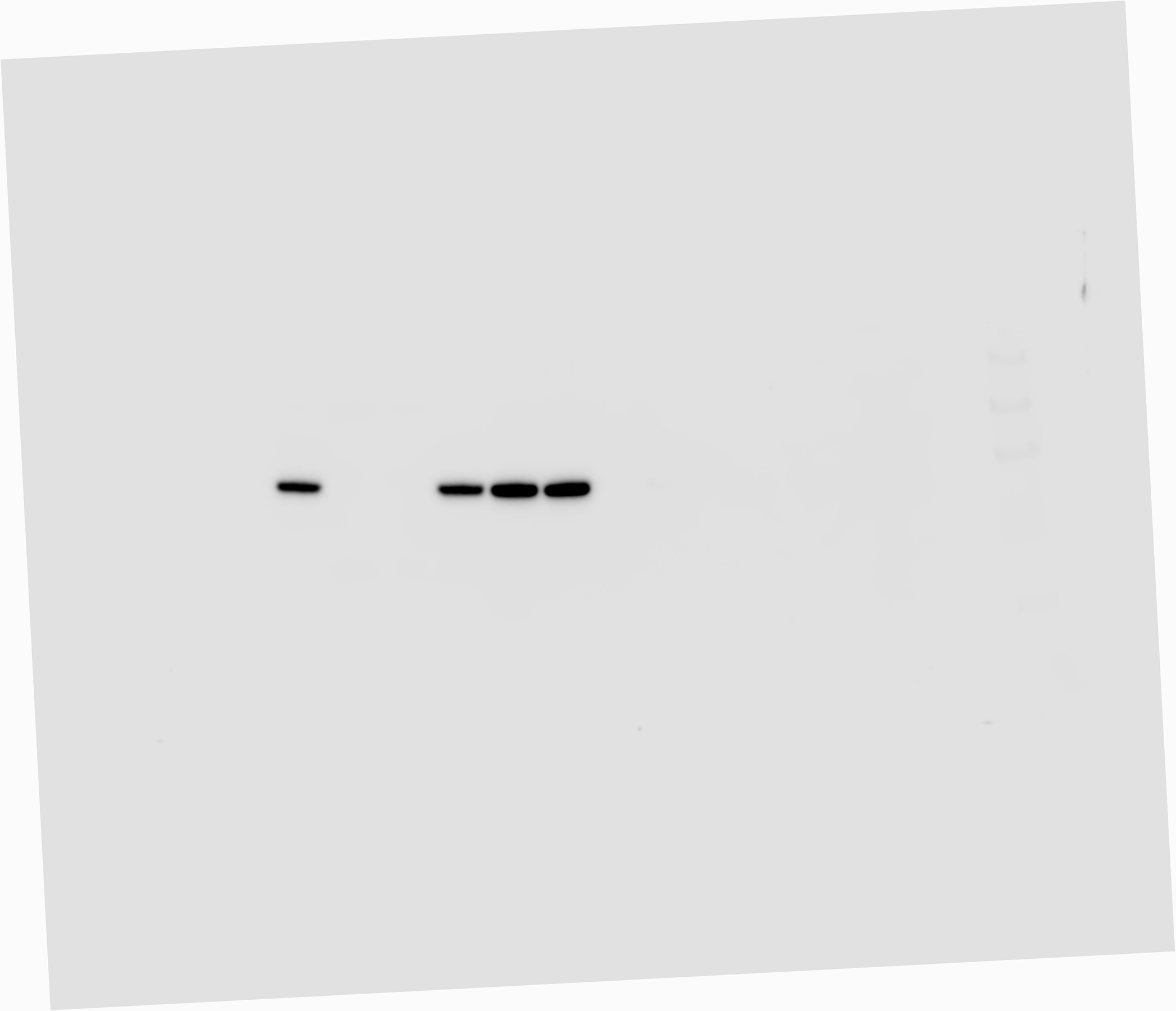

Supplement: Supplementary file 7 — Source data Fig. 5 [file 44318_2026_755_MOESM7_ESM.zip › EMBOJ-2025-121050 Figure 5/Western TIF/5B/5B WCL HA-ASC.tif]

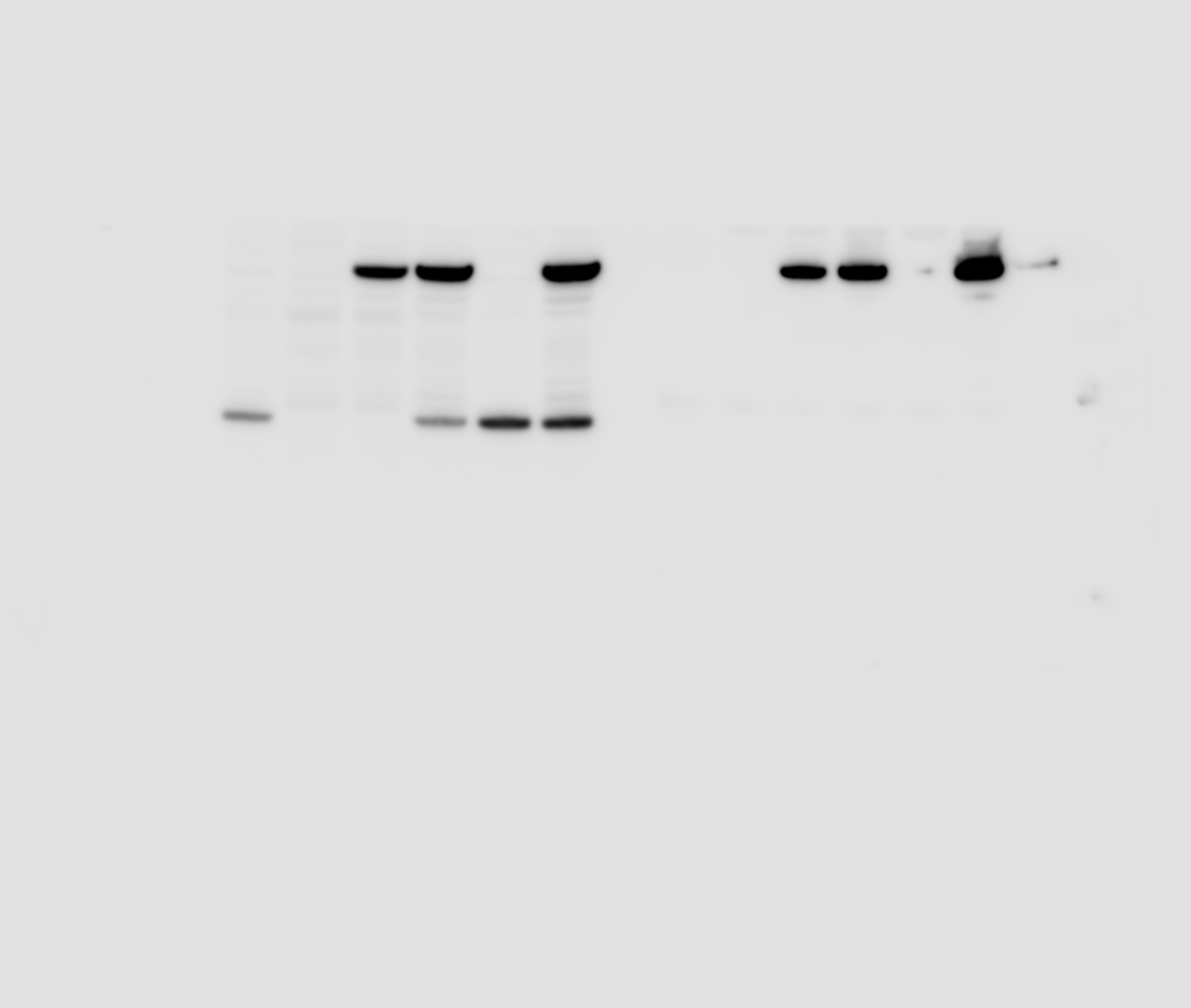

Supplement: Supplementary file 7 — Source data Fig. 5 [file 44318_2026_755_MOESM7_ESM.zip › EMBOJ-2025-121050 Figure 5/Western TIF/5B/5B IP ECFP-Rab11.tif]

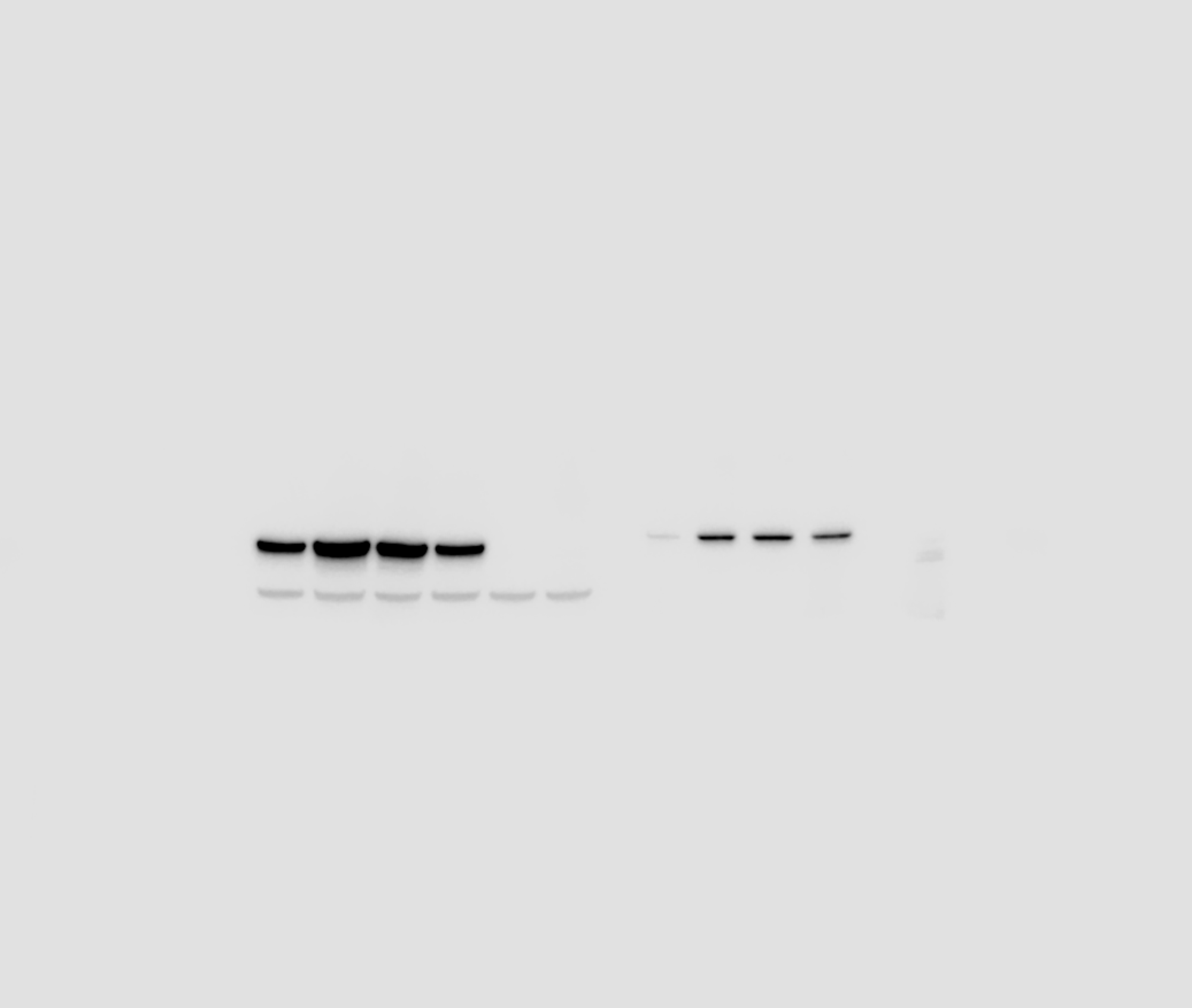

Supplement: Supplementary file 7 — Source data Fig. 5 [file 44318_2026_755_MOESM7_ESM.zip › EMBOJ-2025-121050 Figure 5/Western TIF/5B/5B WCL EGFP-NLRP3.tif]

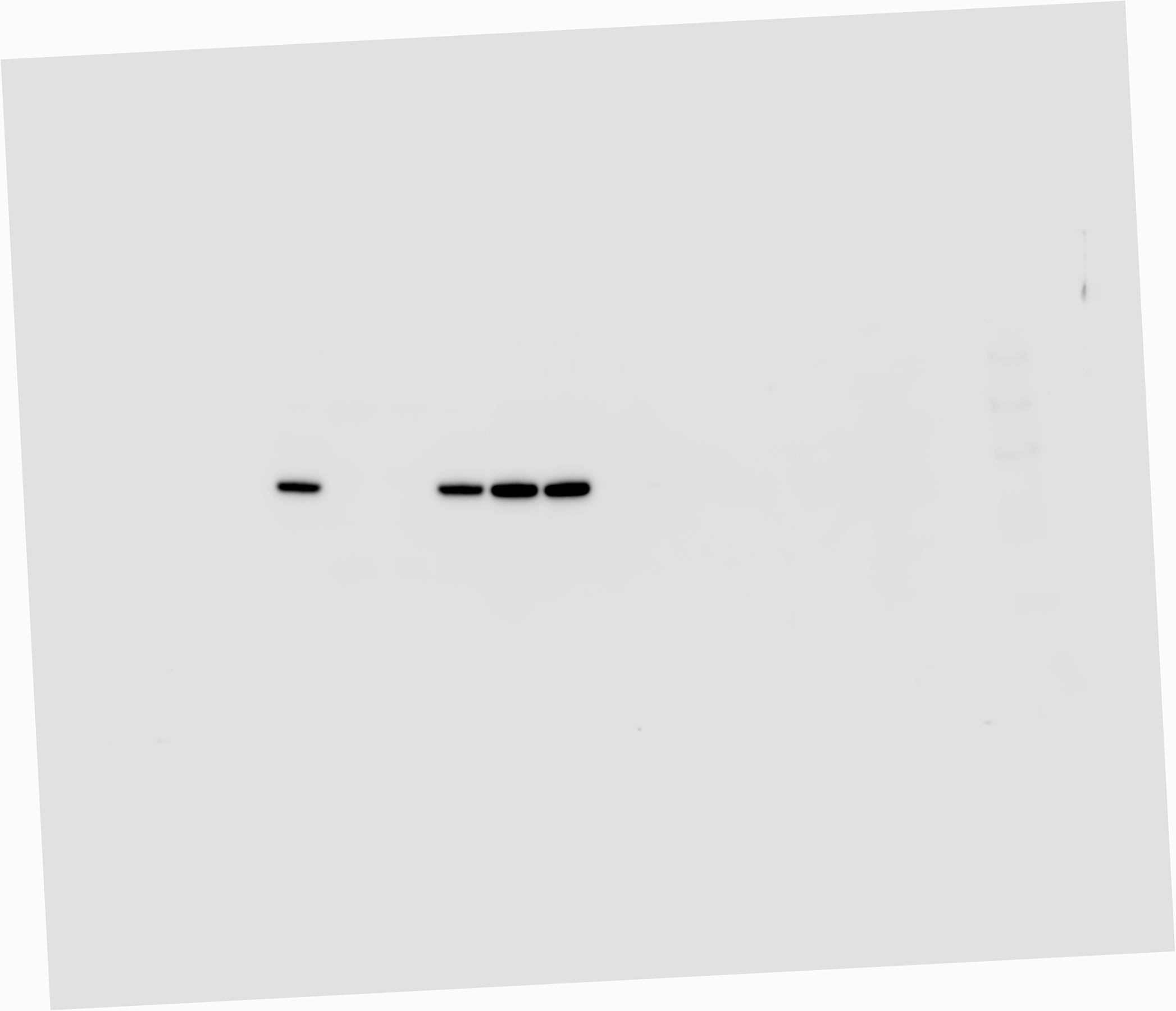

Supplement: Supplementary file 7 — Source data Fig. 5 [file 44318_2026_755_MOESM7_ESM.zip › EMBOJ-2025-121050 Figure 5/Western TIF/5B/5B IP HA-ASC.tif]

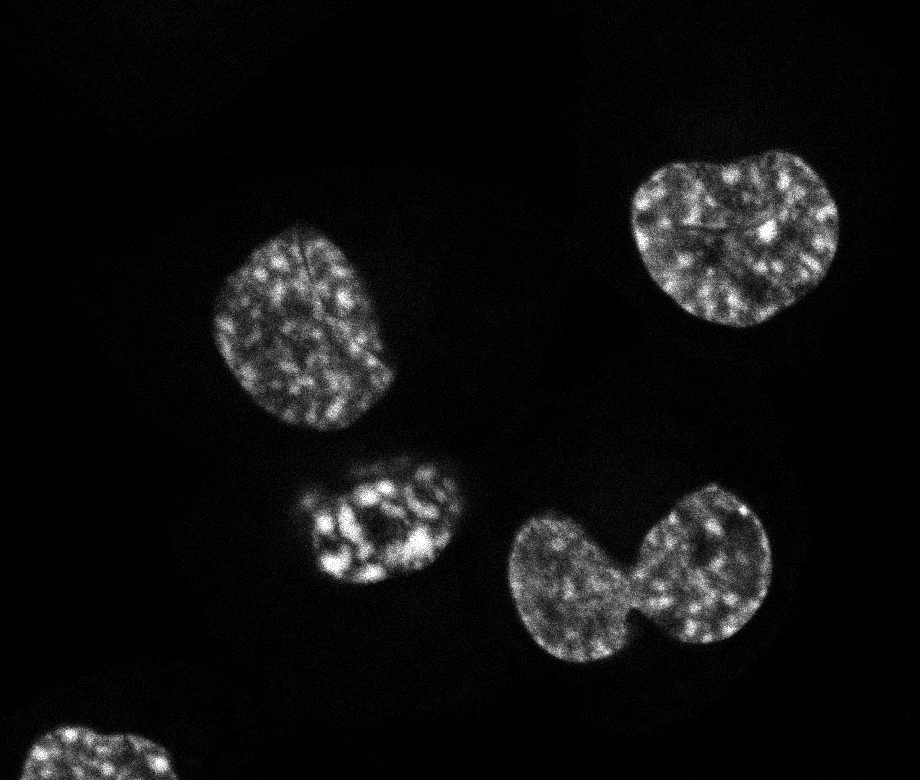

Supplement: Supplementary file 8 — Source data Fig. 6 [file 44318_2026_755_MOESM8_ESM.zip › EMBOJ-2025-121050 Figure 6/6A/6A Microscopy Z-stack.tif]

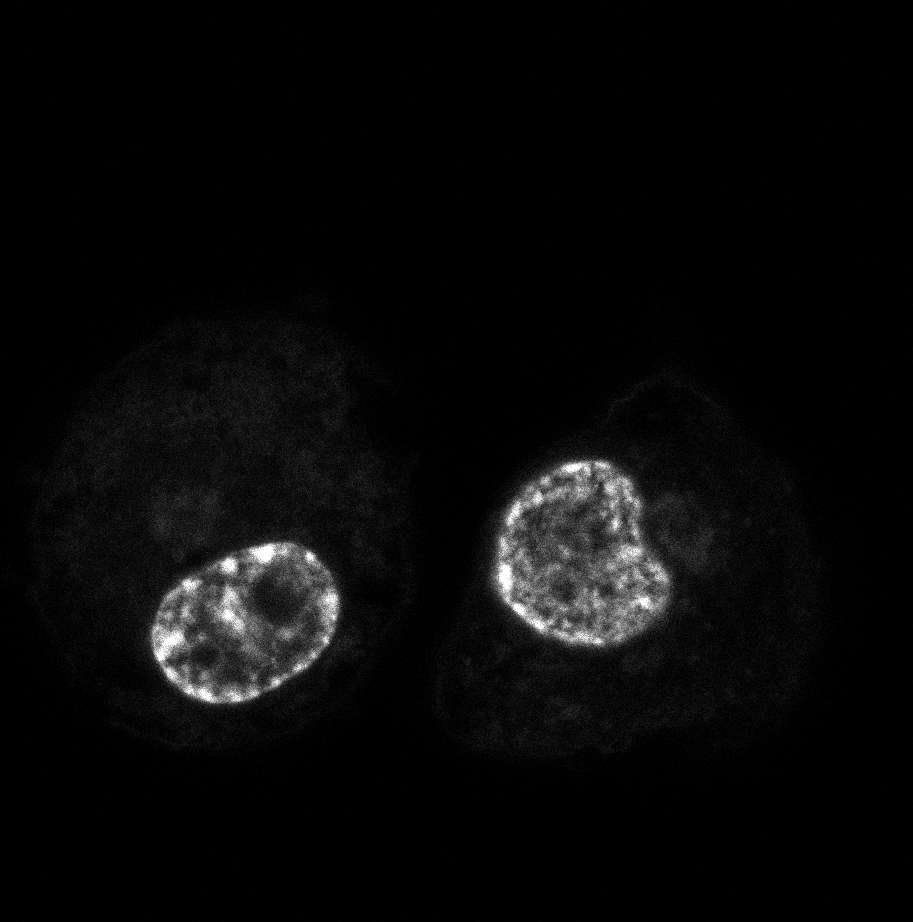

Supplement: Supplementary file 8 — Source data Fig. 6 [file 44318_2026_755_MOESM8_ESM.zip › EMBOJ-2025-121050 Figure 6/6B/6B microscopy Z-stack.tif]

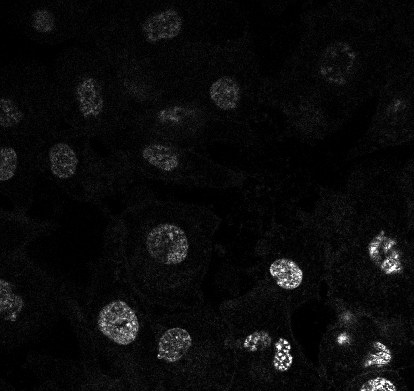

Supplement: Supplementary file 8 — Source data Fig. 6 [file 44318_2026_755_MOESM8_ESM.zip › EMBOJ-2025-121050 Figure 6/6C/6C microscopy Z-stack.tif]

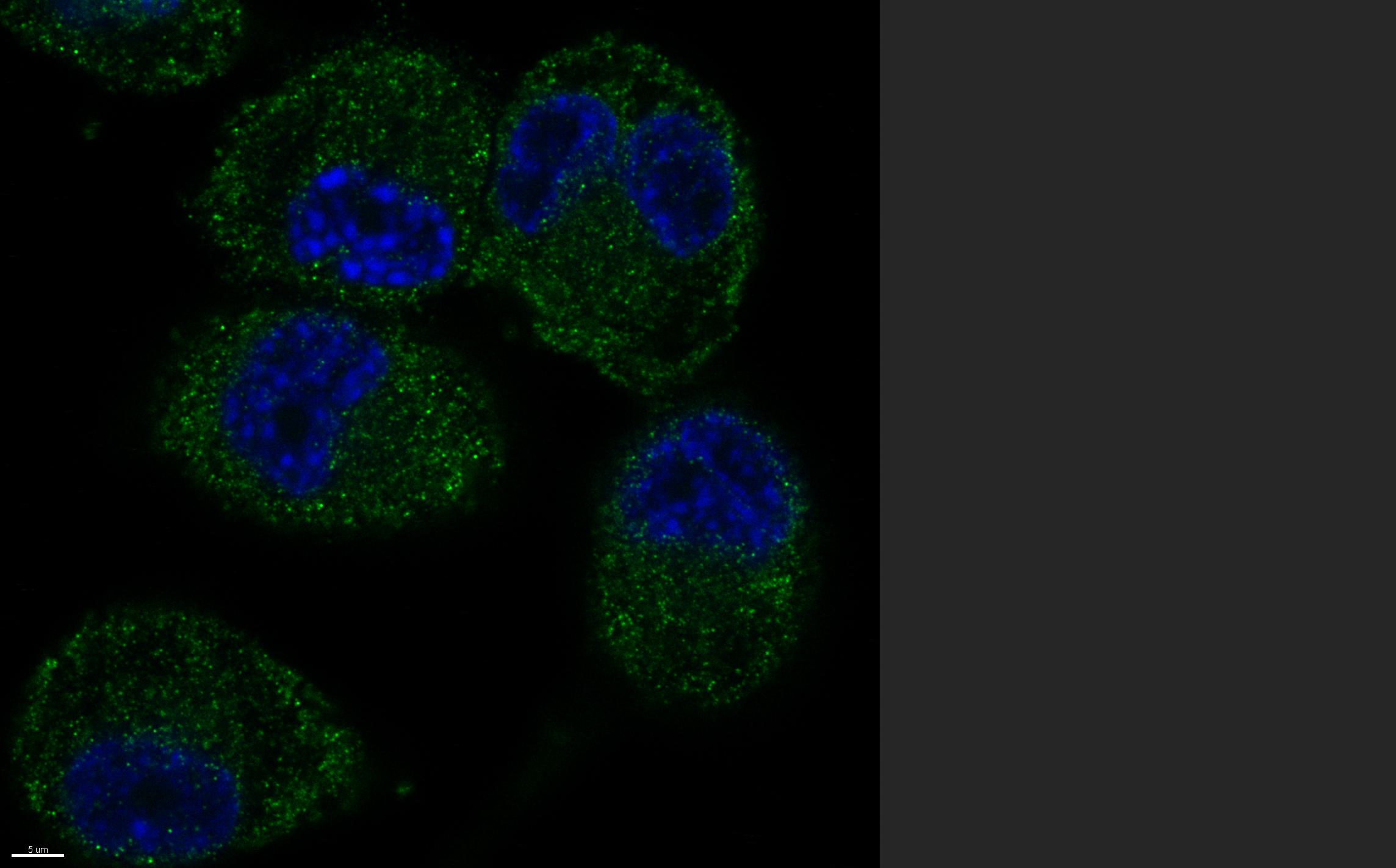

Supplement: Supplementary file 8 — Source data Fig. 6 [file 44318_2026_755_MOESM8_ESM.zip › EMBOJ-2025-121050 Figure 6/Figure 6 Microscopy TIF/6A/Microscopy NLRP3.tif]

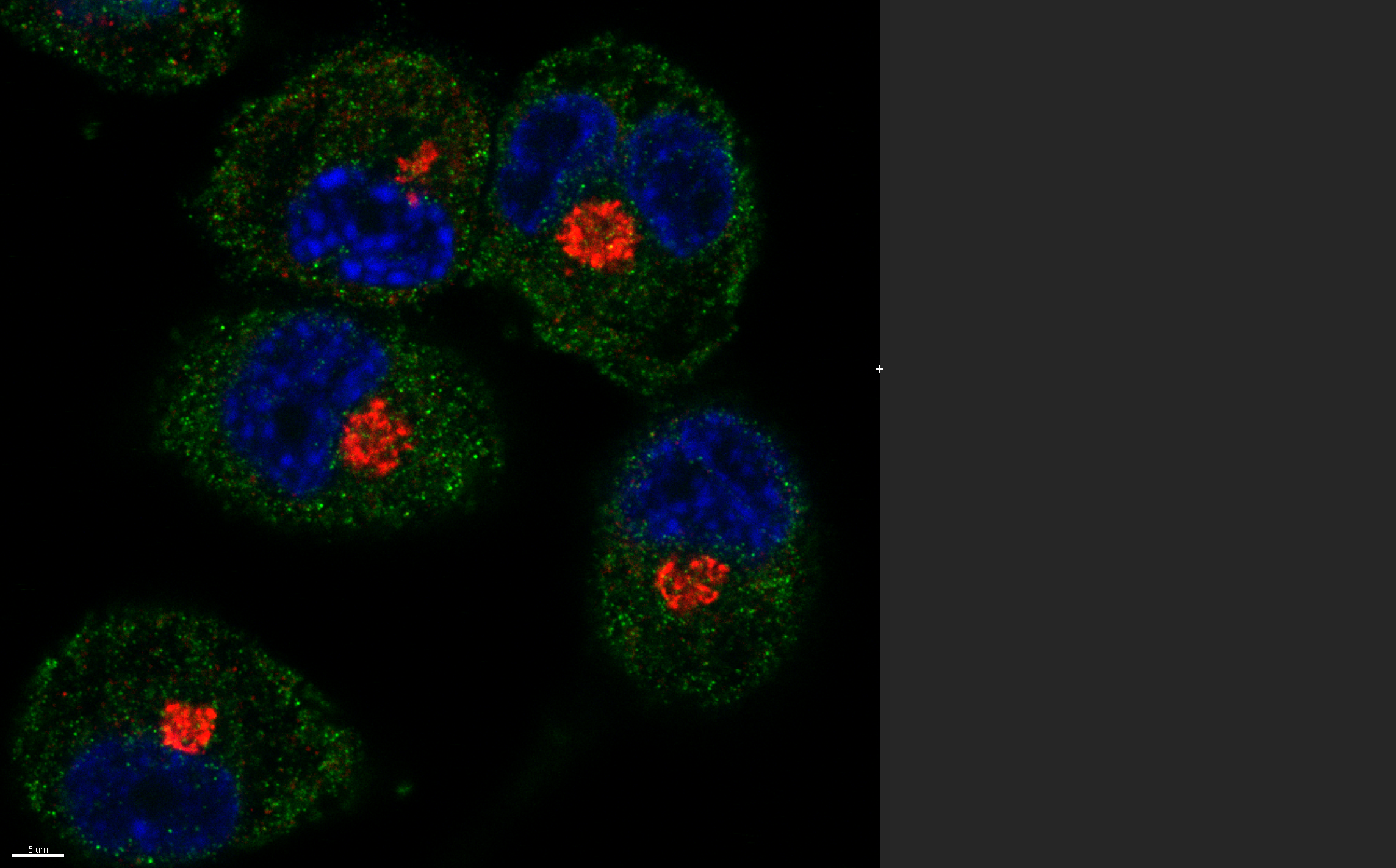

Supplement: Supplementary file 8 — Source data Fig. 6 [file 44318_2026_755_MOESM8_ESM.zip › EMBOJ-2025-121050 Figure 6/Figure 6 Microscopy TIF/6A/Microscopy NLRP3-TGN46.tif]

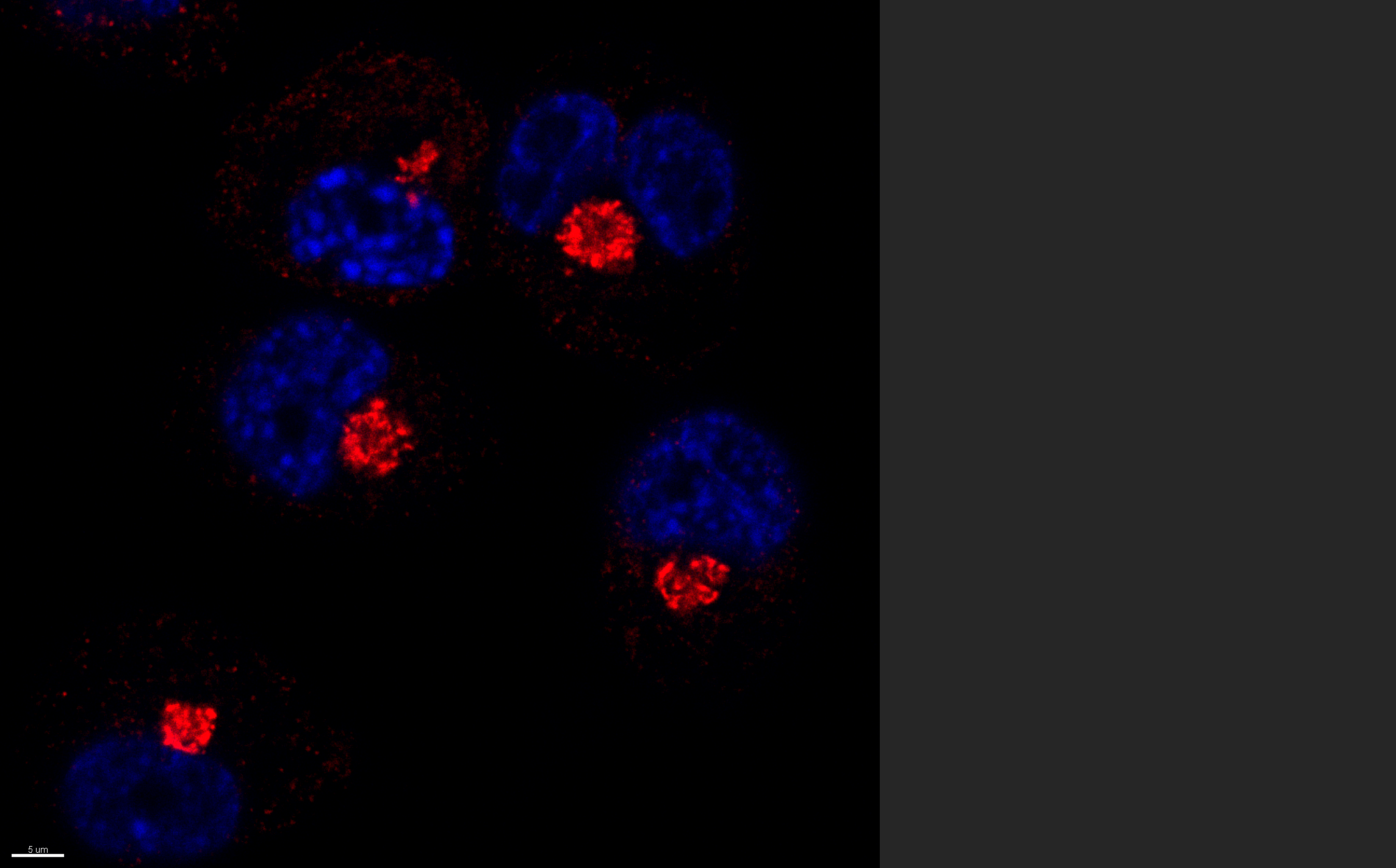

Supplement: Supplementary file 8 — Source data Fig. 6 [file 44318_2026_755_MOESM8_ESM.zip › EMBOJ-2025-121050 Figure 6/Figure 6 Microscopy TIF/6A/Microscopy TGN46.tif]

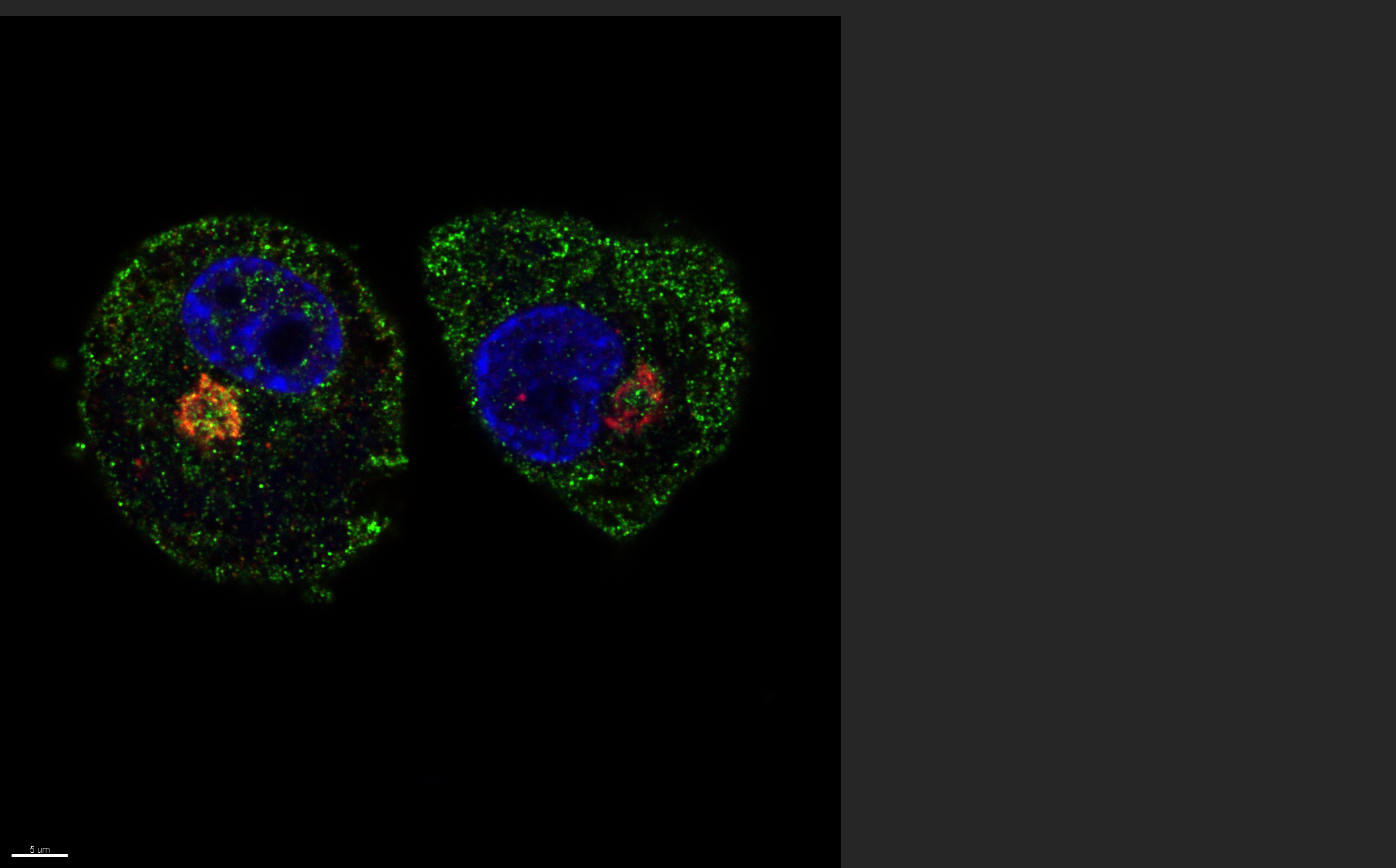

Supplement: Supplementary file 8 — Source data Fig. 6 [file 44318_2026_755_MOESM8_ESM.zip › EMBOJ-2025-121050 Figure 6/Figure 6 Microscopy TIF/6B/Microscopy NLRP3-TGN46.tif]

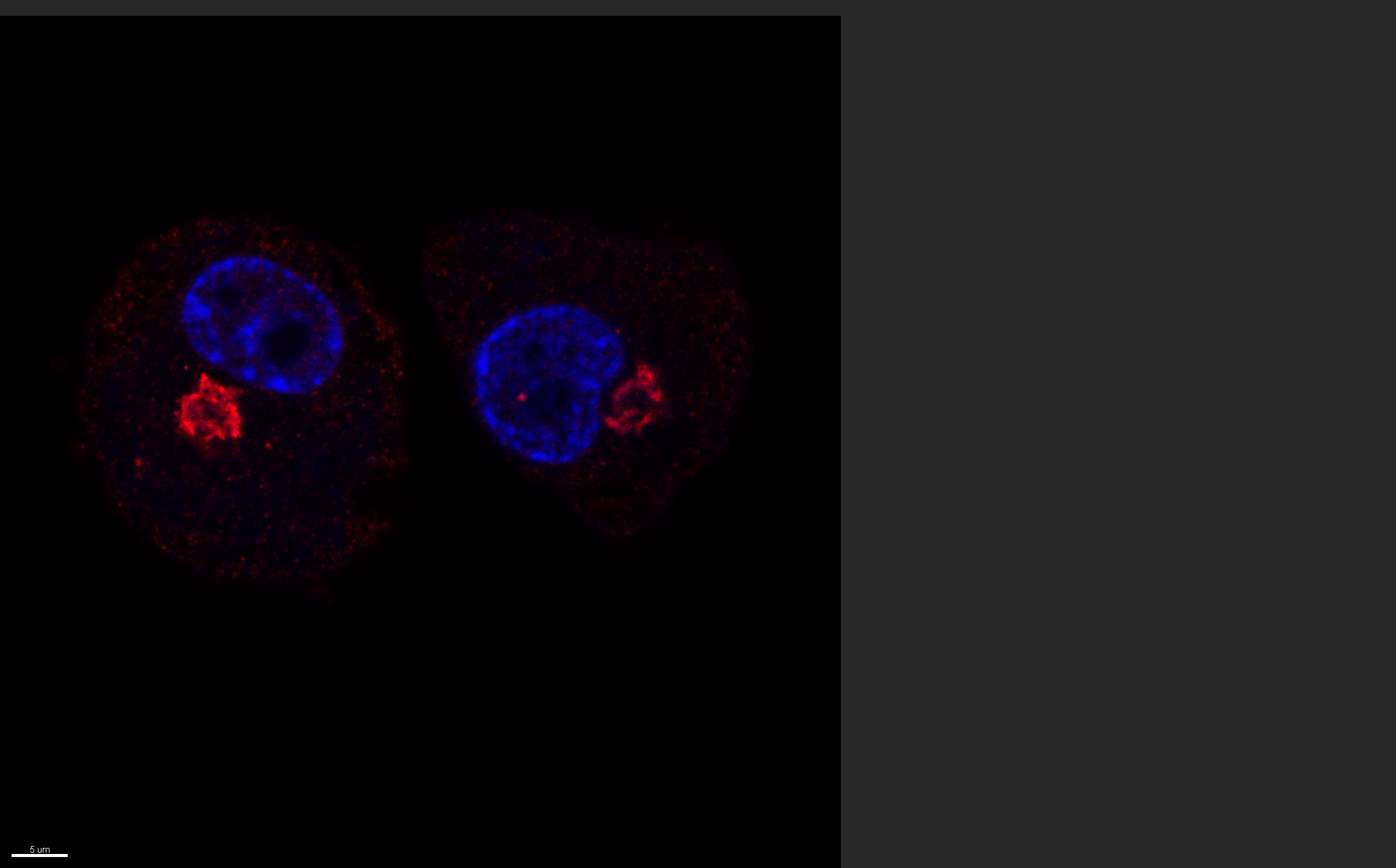

Supplement: Supplementary file 8 — Source data Fig. 6 [file 44318_2026_755_MOESM8_ESM.zip › EMBOJ-2025-121050 Figure 6/Figure 6 Microscopy TIF/6B/Microscopy TGN46.tif]

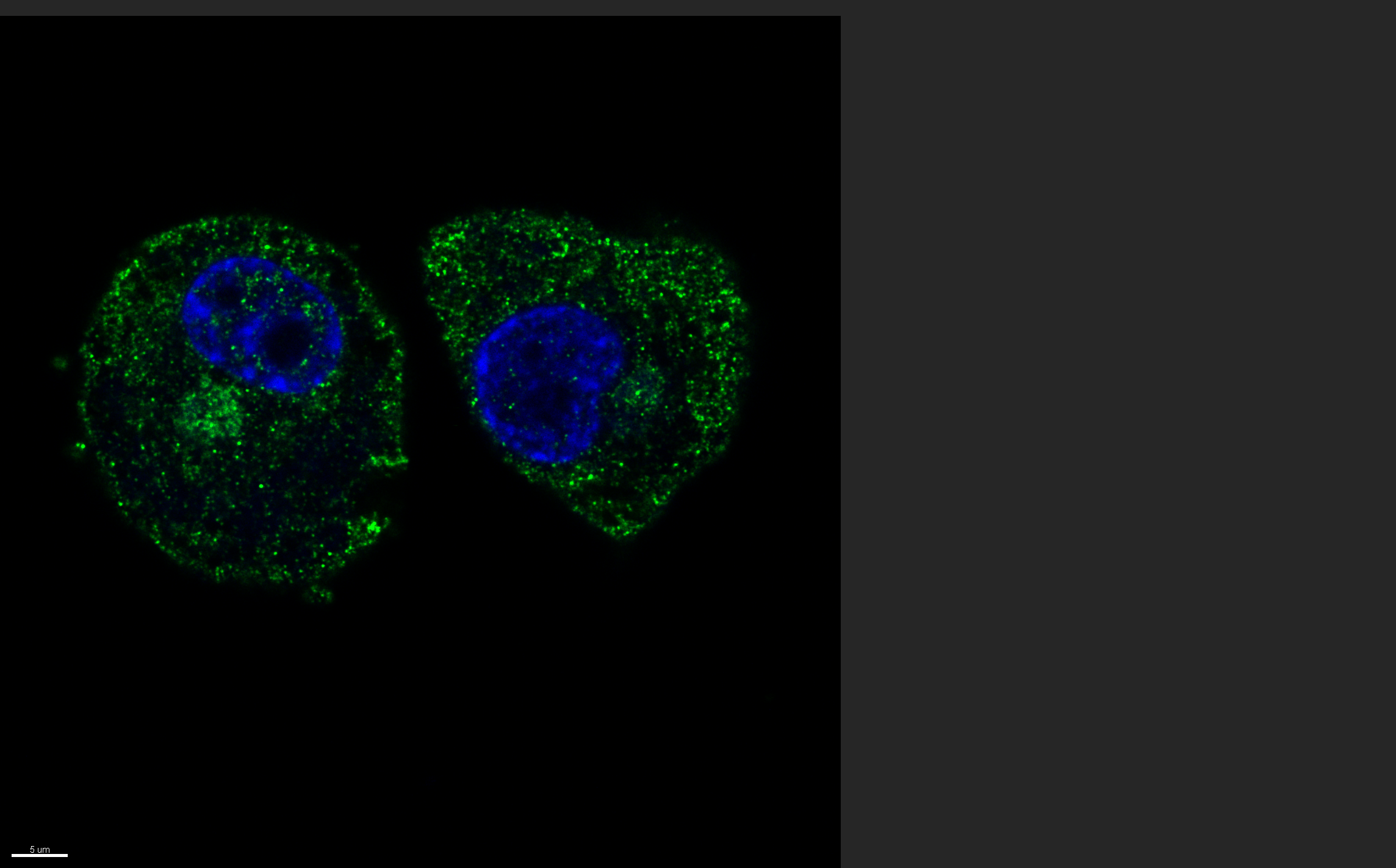

Supplement: Supplementary file 8 — Source data Fig. 6 [file 44318_2026_755_MOESM8_ESM.zip › EMBOJ-2025-121050 Figure 6/Figure 6 Microscopy TIF/6B/Microscopy NLRP3.tif]

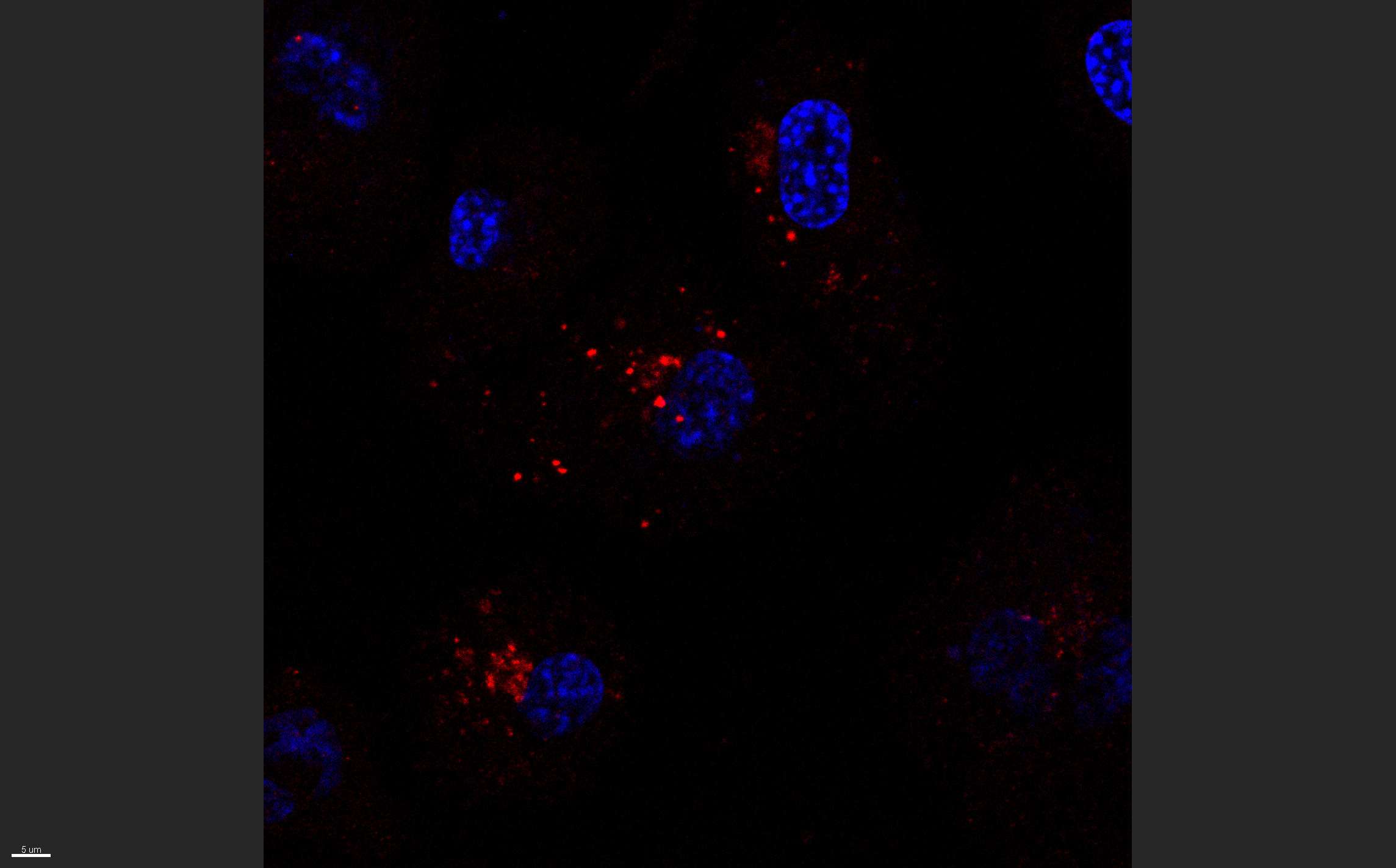

Supplement: Supplementary file 8 — Source data Fig. 6 [file 44318_2026_755_MOESM8_ESM.zip › EMBOJ-2025-121050 Figure 6/Figure 6 Microscopy TIF/6D/Microscopy TGN46.tif]

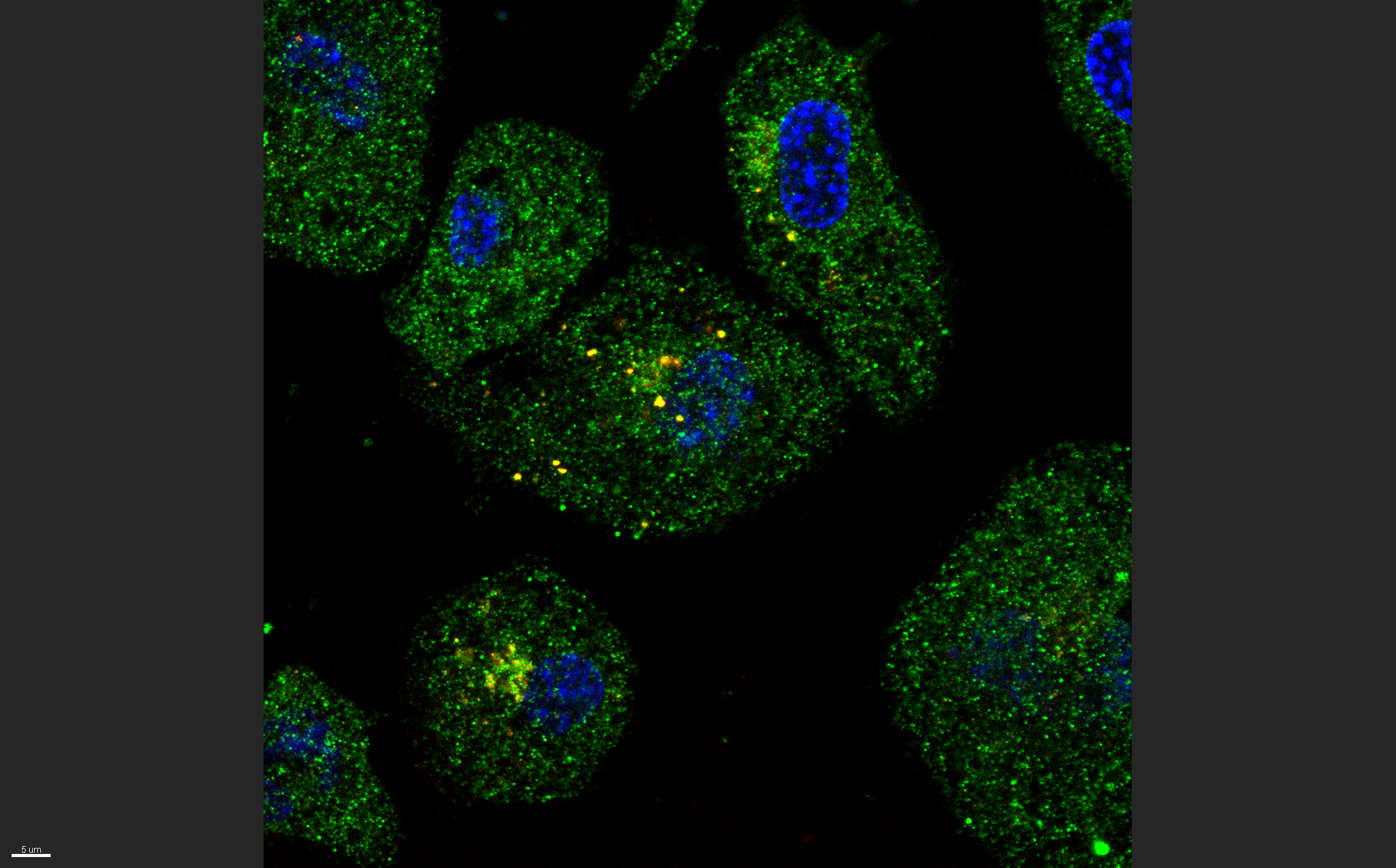

Supplement: Supplementary file 8 — Source data Fig. 6 [file 44318_2026_755_MOESM8_ESM.zip › EMBOJ-2025-121050 Figure 6/Figure 6 Microscopy TIF/6D/Microscopy FIP2-TGN46.tif]

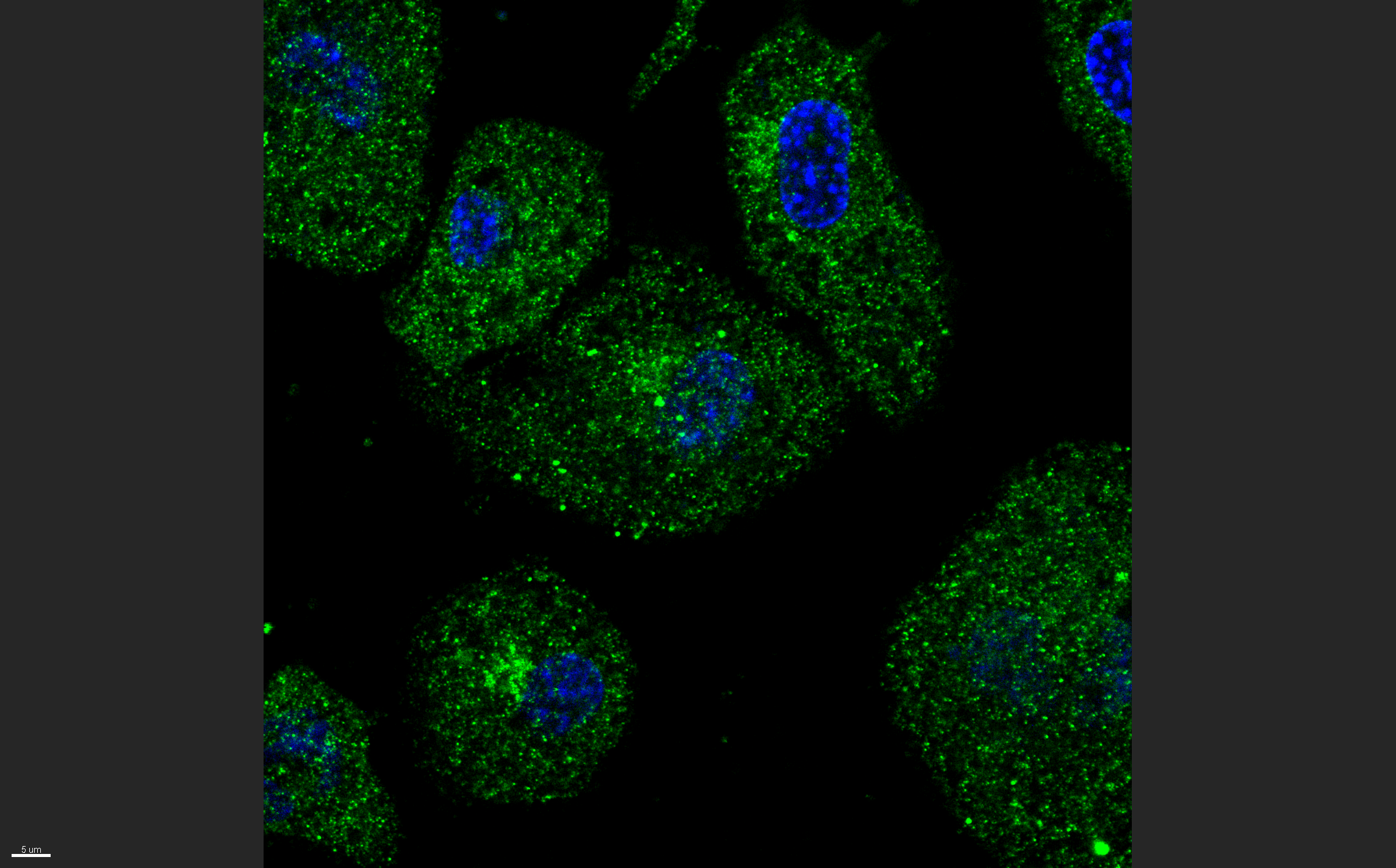

Supplement: Supplementary file 8 — Source data Fig. 6 [file 44318_2026_755_MOESM8_ESM.zip › EMBOJ-2025-121050 Figure 6/Figure 6 Microscopy TIF/6D/Microscopy FIP2.tif]

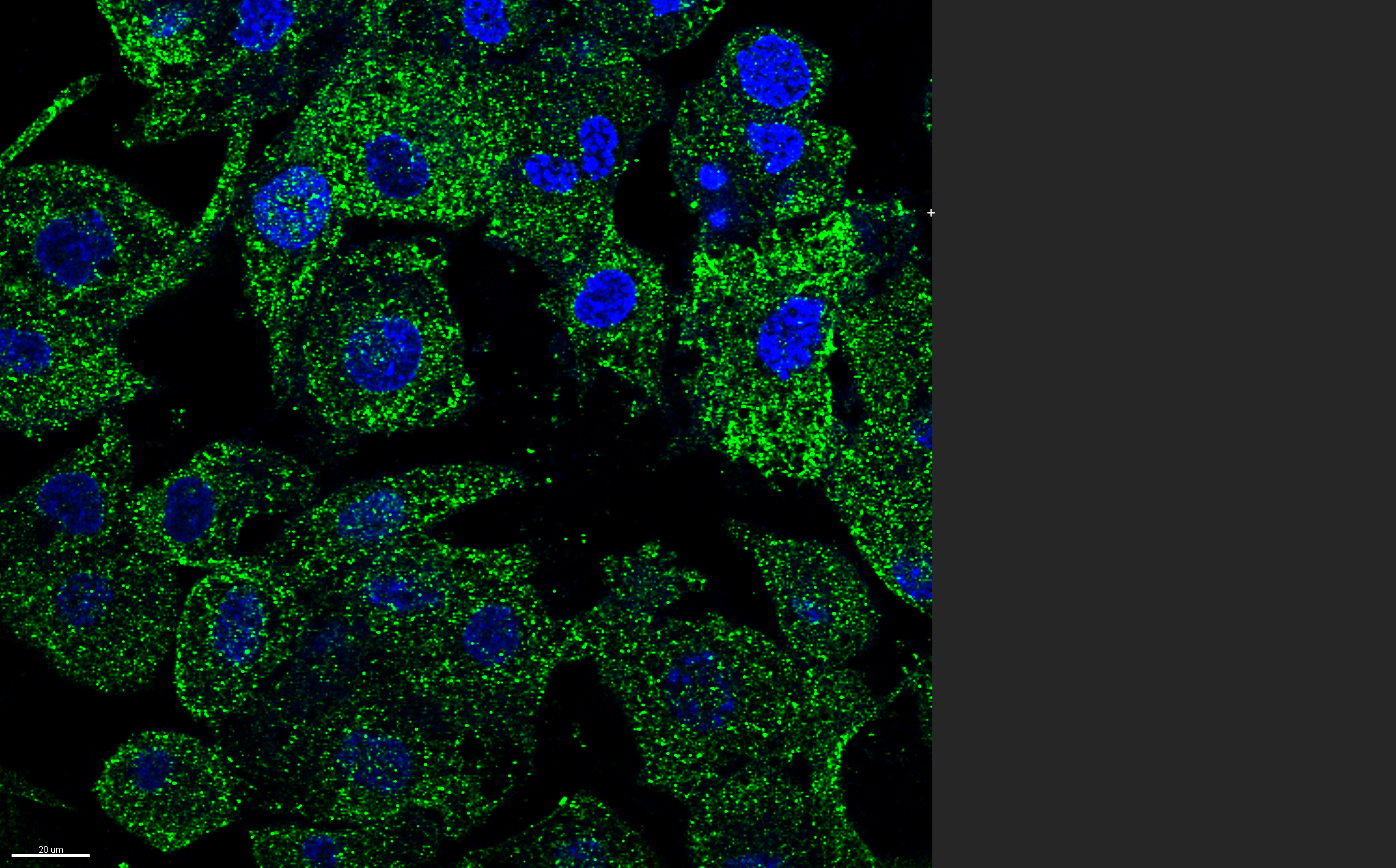

Supplement: Supplementary file 8 — Source data Fig. 6 [file 44318_2026_755_MOESM8_ESM.zip › EMBOJ-2025-121050 Figure 6/Figure 6 Microscopy TIF/6C/Microscopy NLRP3.tif]

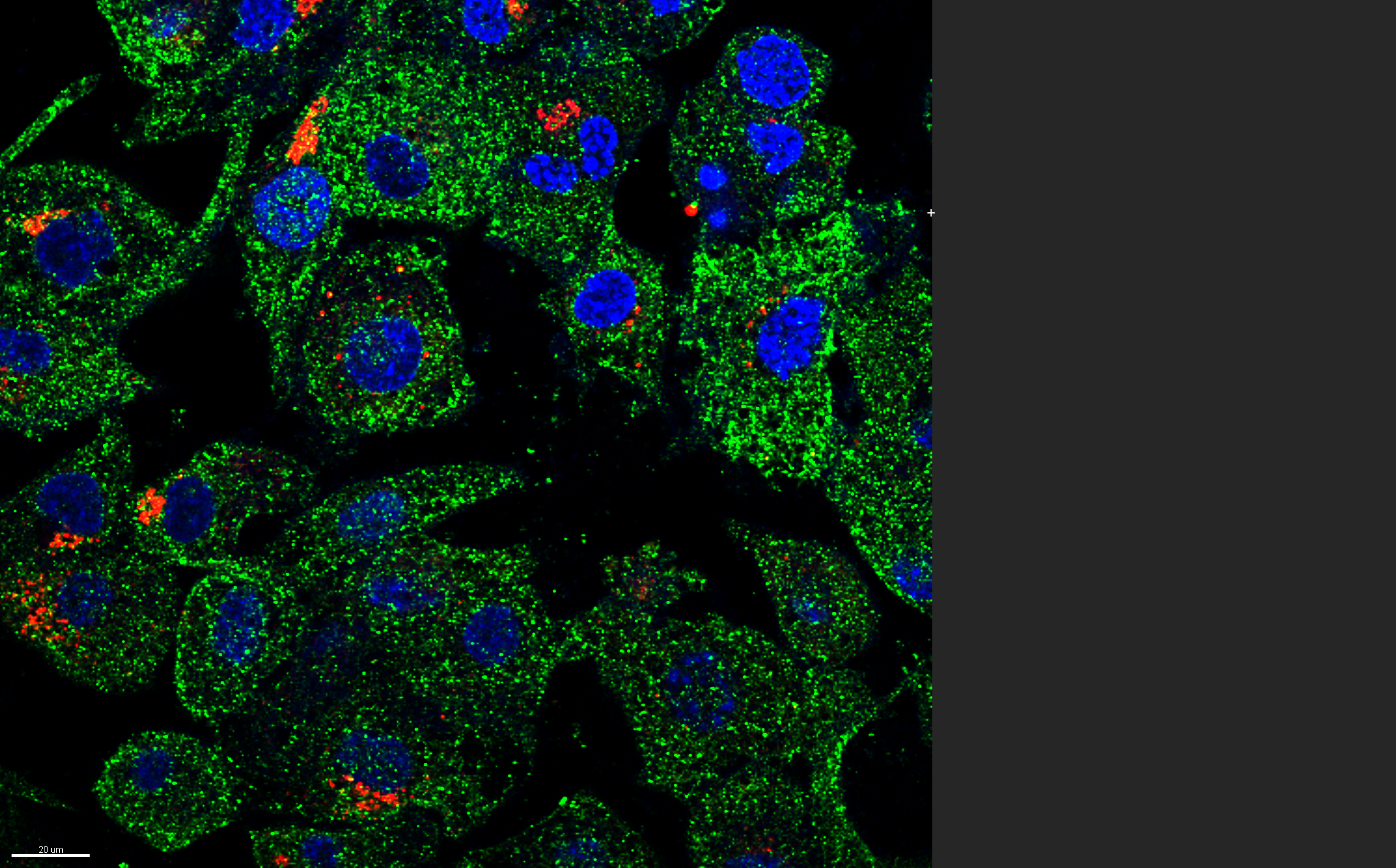

Supplement: Supplementary file 8 — Source data Fig. 6 [file 44318_2026_755_MOESM8_ESM.zip › EMBOJ-2025-121050 Figure 6/Figure 6 Microscopy TIF/6C/Microscopy NLRP3-TGN46.tif]

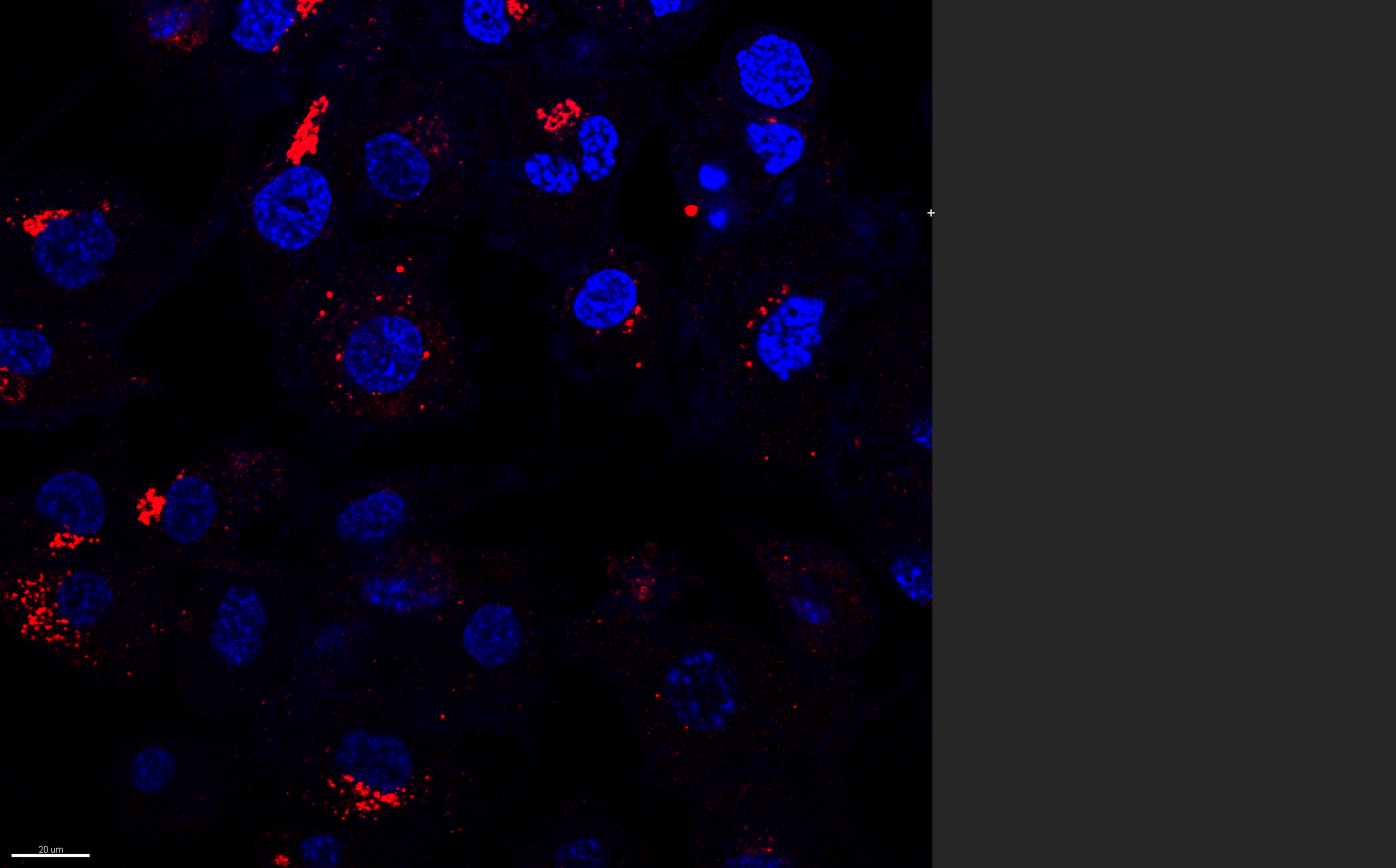

Supplement: Supplementary file 8 — Source data Fig. 6 [file 44318_2026_755_MOESM8_ESM.zip › EMBOJ-2025-121050 Figure 6/Figure 6 Microscopy TIF/6C/Microscopy TGN46.tif]

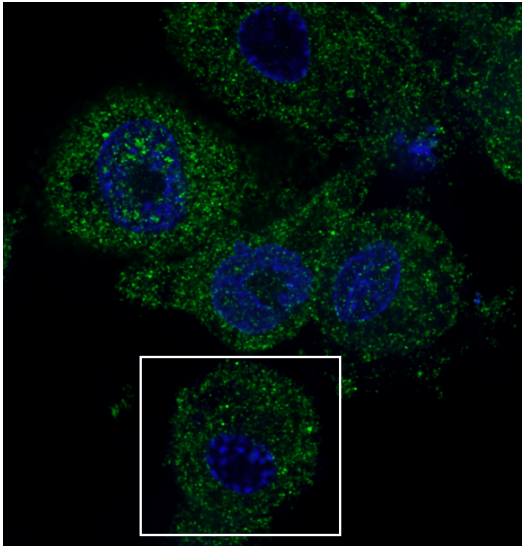

NLRP3 Crop

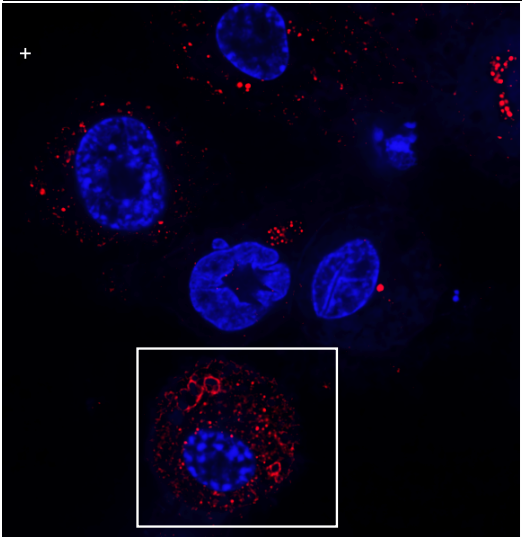

FIP2 Crop

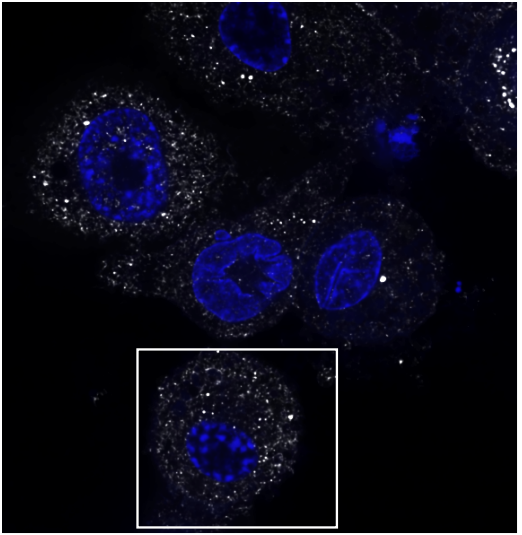

co-localization Crop

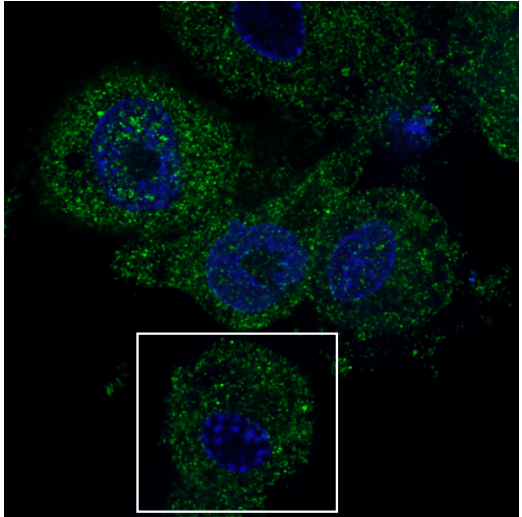

NLRP3\_FIP2 Crop

Supplement: Supplementary file 9 — Source data Fig. 7 [file 44318_2026_755_MOESM9_ESM.zip › EMBOJ-2025-121050 Figure 7/7D/~ai-c88b2f41-44b9-4c39-9cfc-ecb717f76449_.tmp]

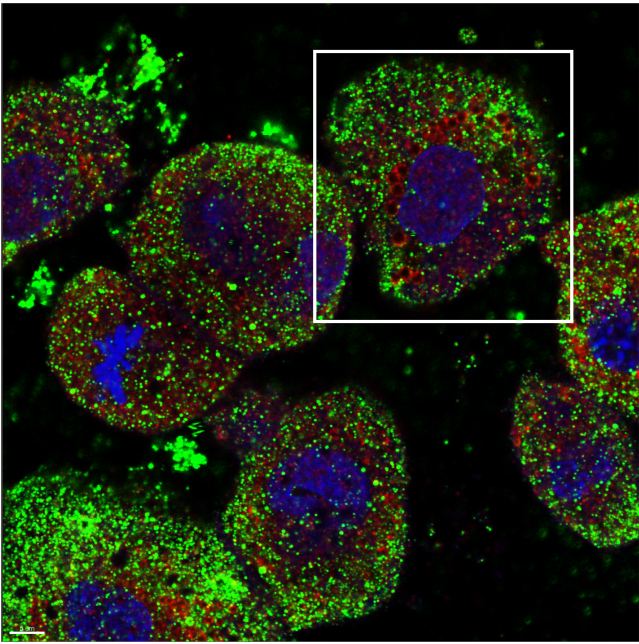

NLRP3\_Rab5 Crop

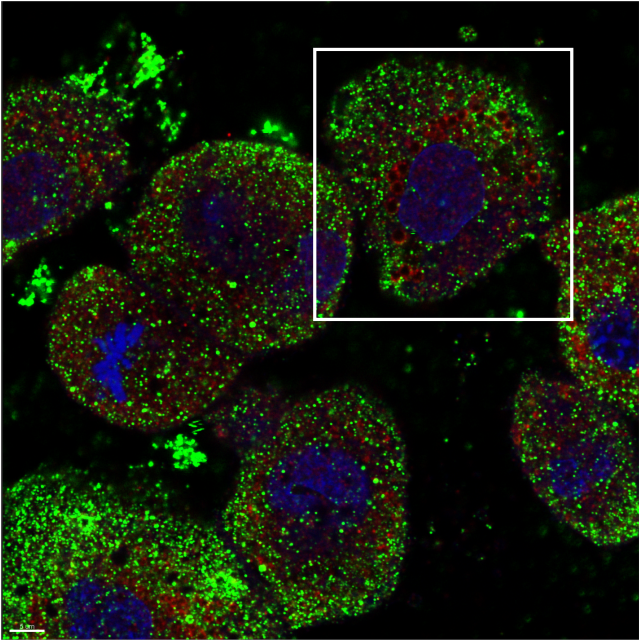

PI4P Crop

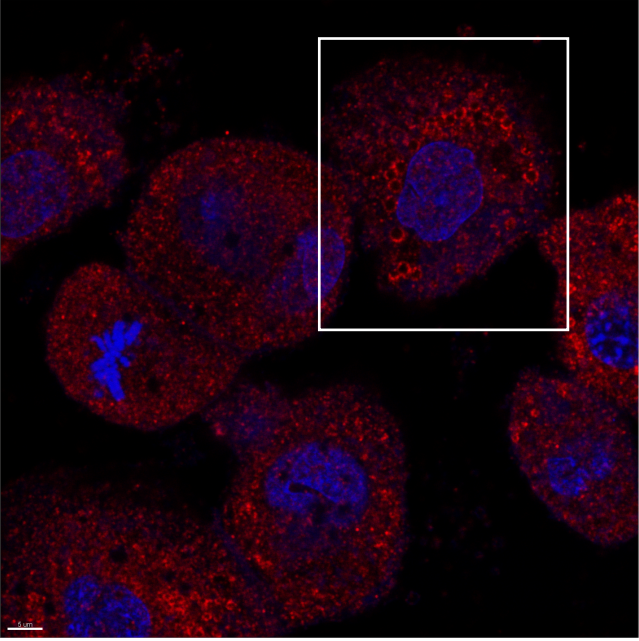

Rab5 Crop

Supplement: Supplementary file 9 — Source data Fig. 7 [file 44318_2026_755_MOESM9_ESM.zip › EMBOJ-2025-121050 Figure 7/7C/~ai-072bbd9d-3655-4cd8-8857-0559dfcdeee3_.tmp]

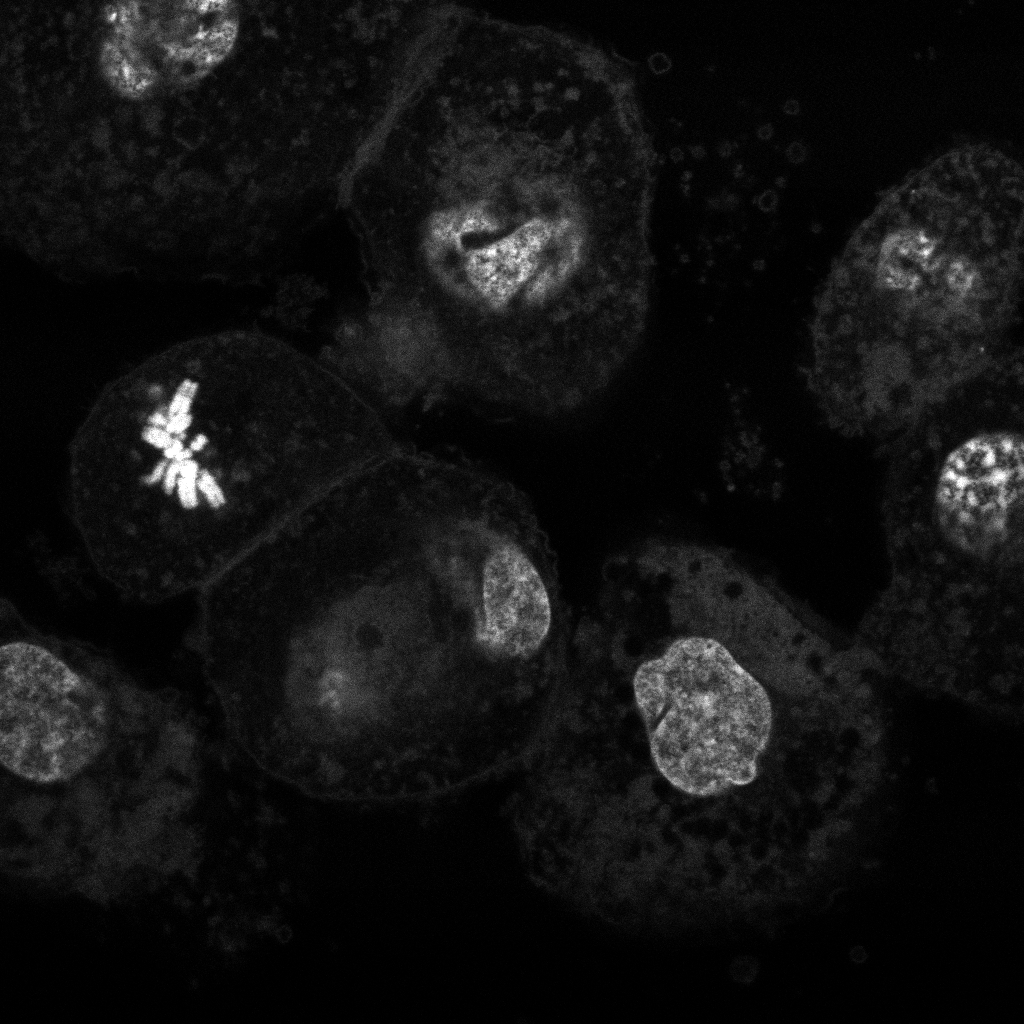

Supplement: Supplementary file 9 — Source data Fig. 7 [file 44318_2026_755_MOESM9_ESM.zip › EMBOJ-2025-121050 Figure 7/7C/7C Microscopy Z-stack.tif]

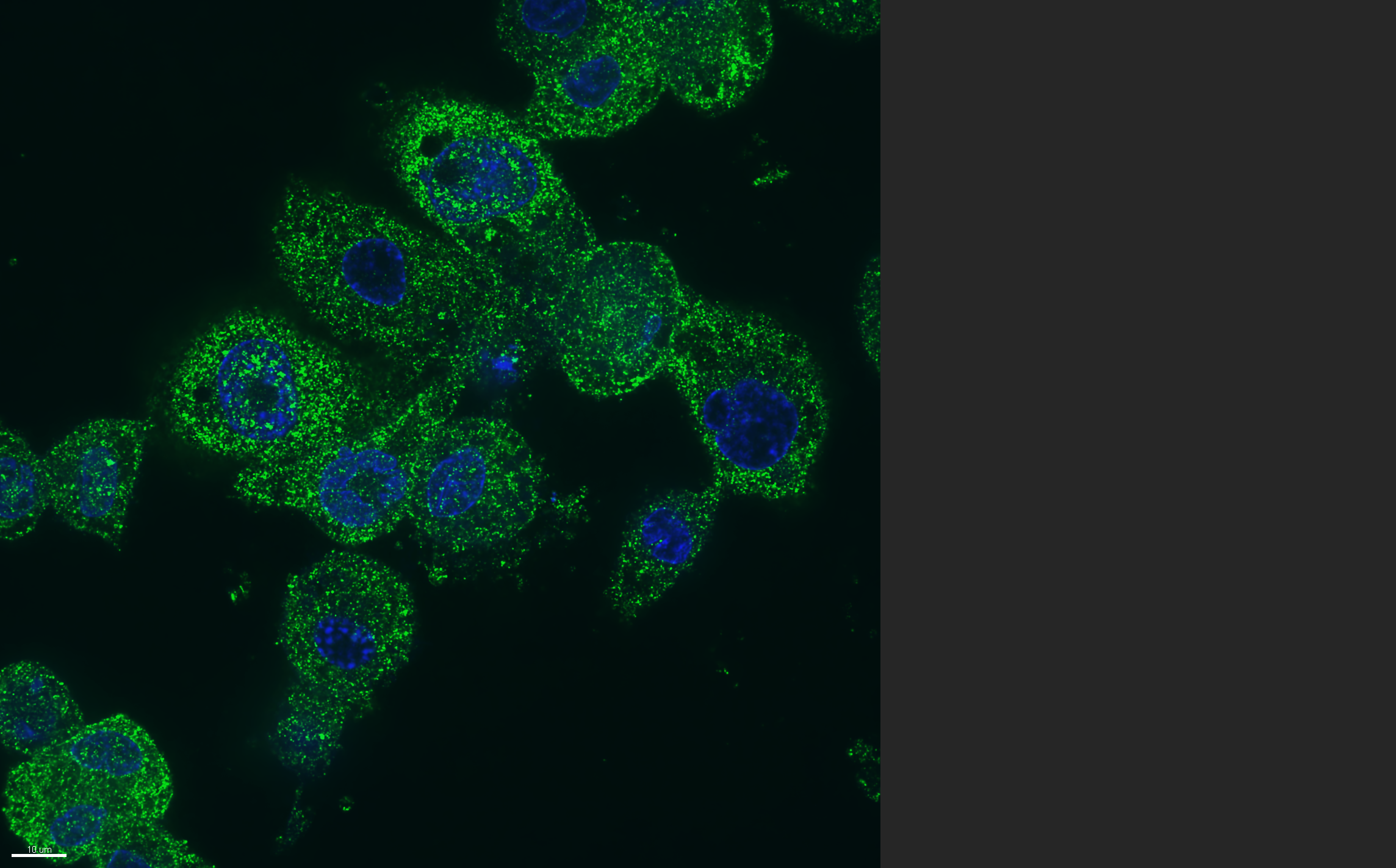

Supplement: Supplementary file 9 — Source data Fig. 7 [file 44318_2026_755_MOESM9_ESM.zip › EMBOJ-2025-121050 Figure 7/Microscopy TIF/7D/7D NLRP3 full view.tif]

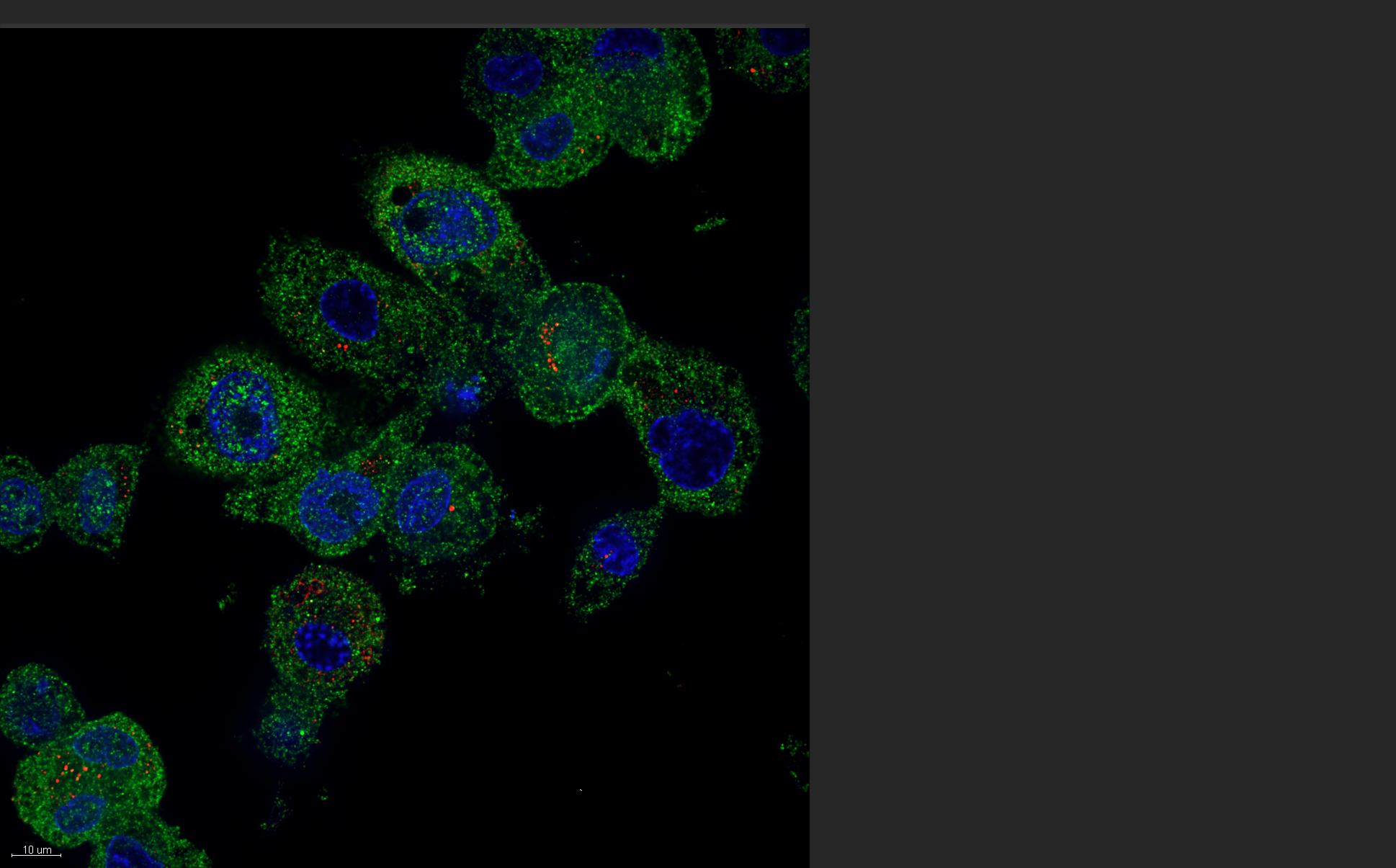

Supplement: Supplementary file 9 — Source data Fig. 7 [file 44318_2026_755_MOESM9_ESM.zip › EMBOJ-2025-121050 Figure 7/Microscopy TIF/7D/7D FIP2_NLRP3 Full view.tif]

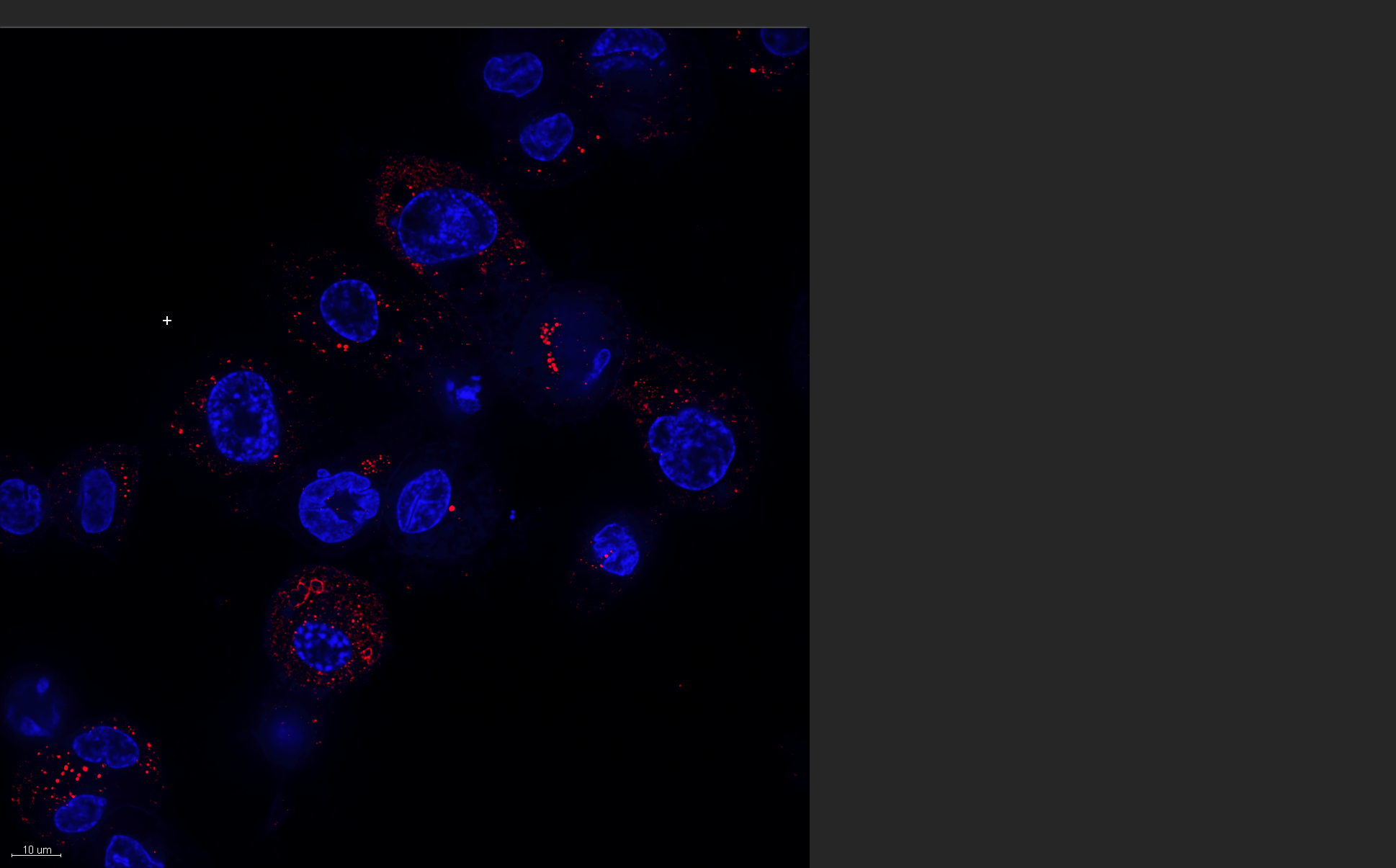

Supplement: Supplementary file 9 — Source data Fig. 7 [file 44318_2026_755_MOESM9_ESM.zip › EMBOJ-2025-121050 Figure 7/Microscopy TIF/7D/7D FIP2 Full view.tif]

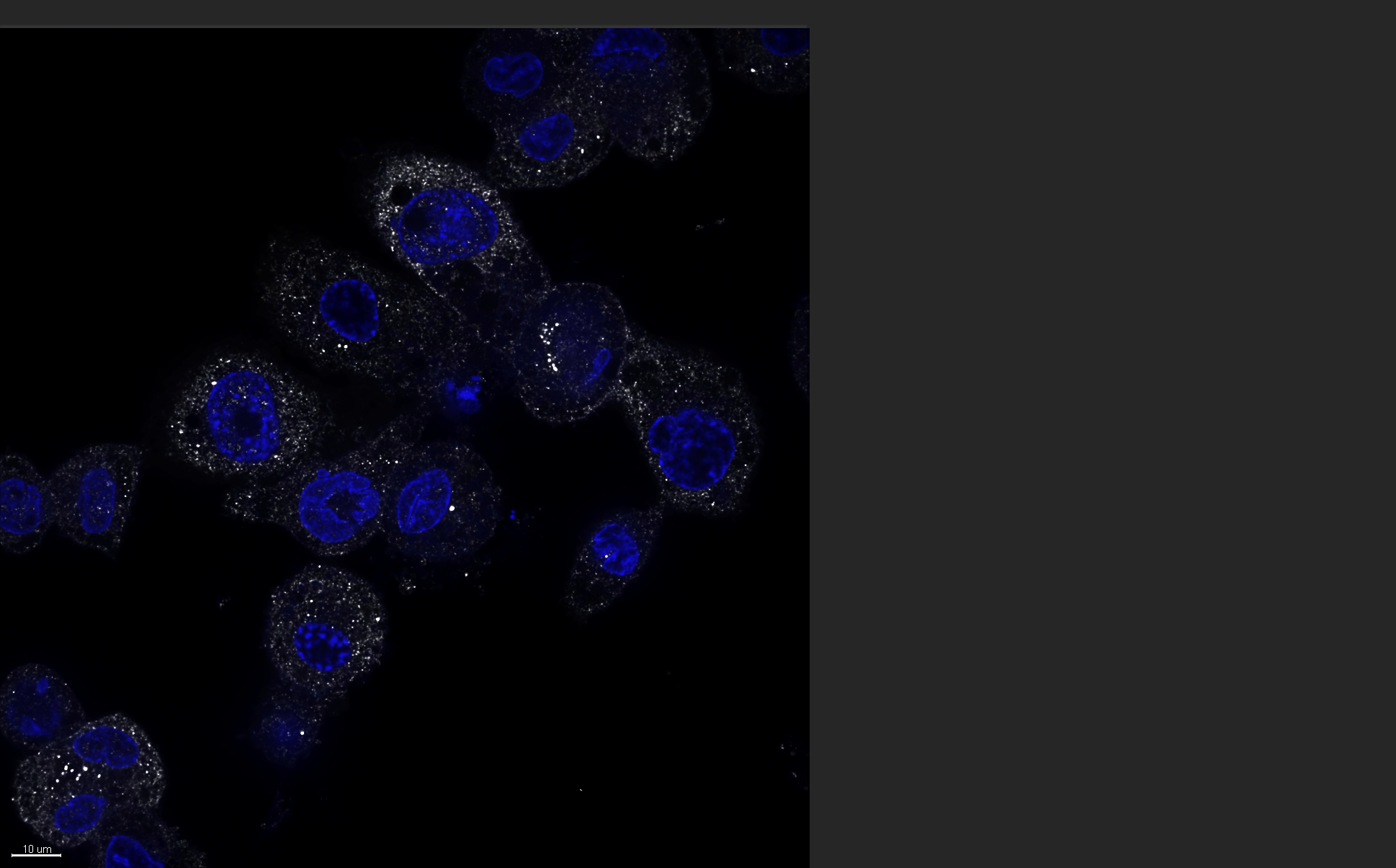

Supplement: Supplementary file 9 — Source data Fig. 7 [file 44318_2026_755_MOESM9_ESM.zip › EMBOJ-2025-121050 Figure 7/Microscopy TIF/7D/7D Calculated OL full view.tif]

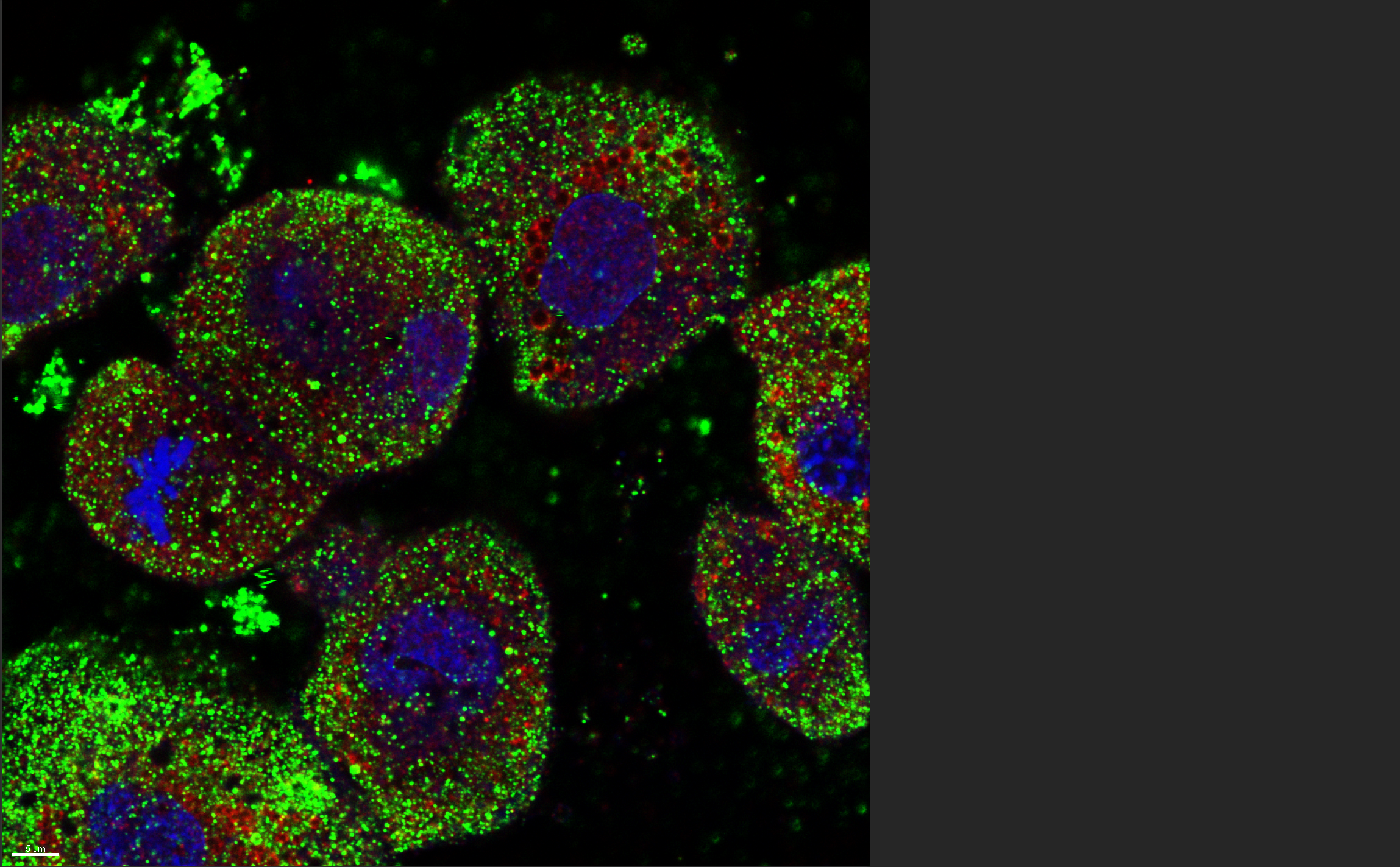

Supplement: Supplementary file 9 — Source data Fig. 7 [file 44318_2026_755_MOESM9_ESM.zip › EMBOJ-2025-121050 Figure 7/Microscopy TIF/7C/7C OL Full view.tif]

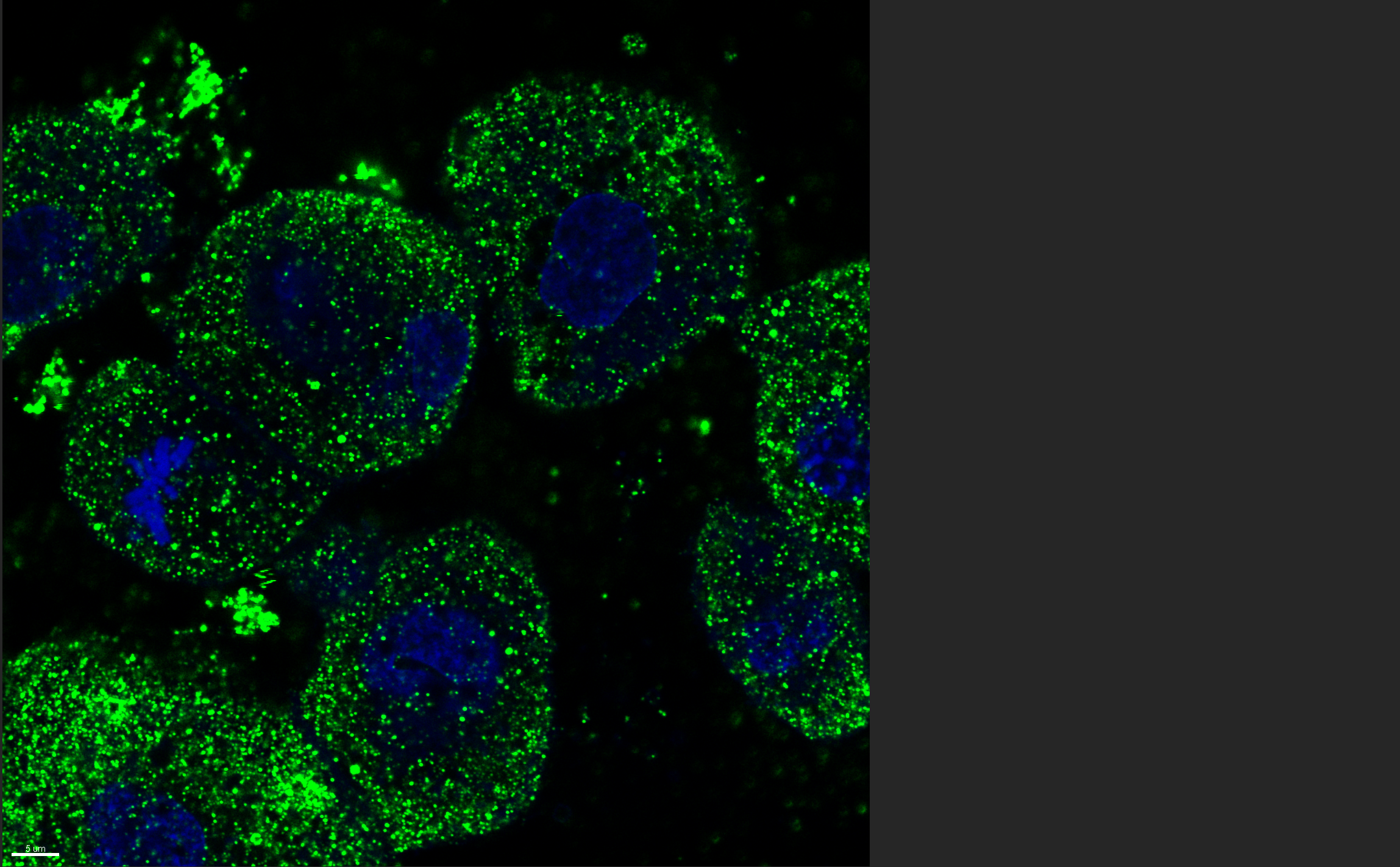

Supplement: Supplementary file 9 — Source data Fig. 7 [file 44318_2026_755_MOESM9_ESM.zip › EMBOJ-2025-121050 Figure 7/Microscopy TIF/7C/7C OL PI4P full view.tif]

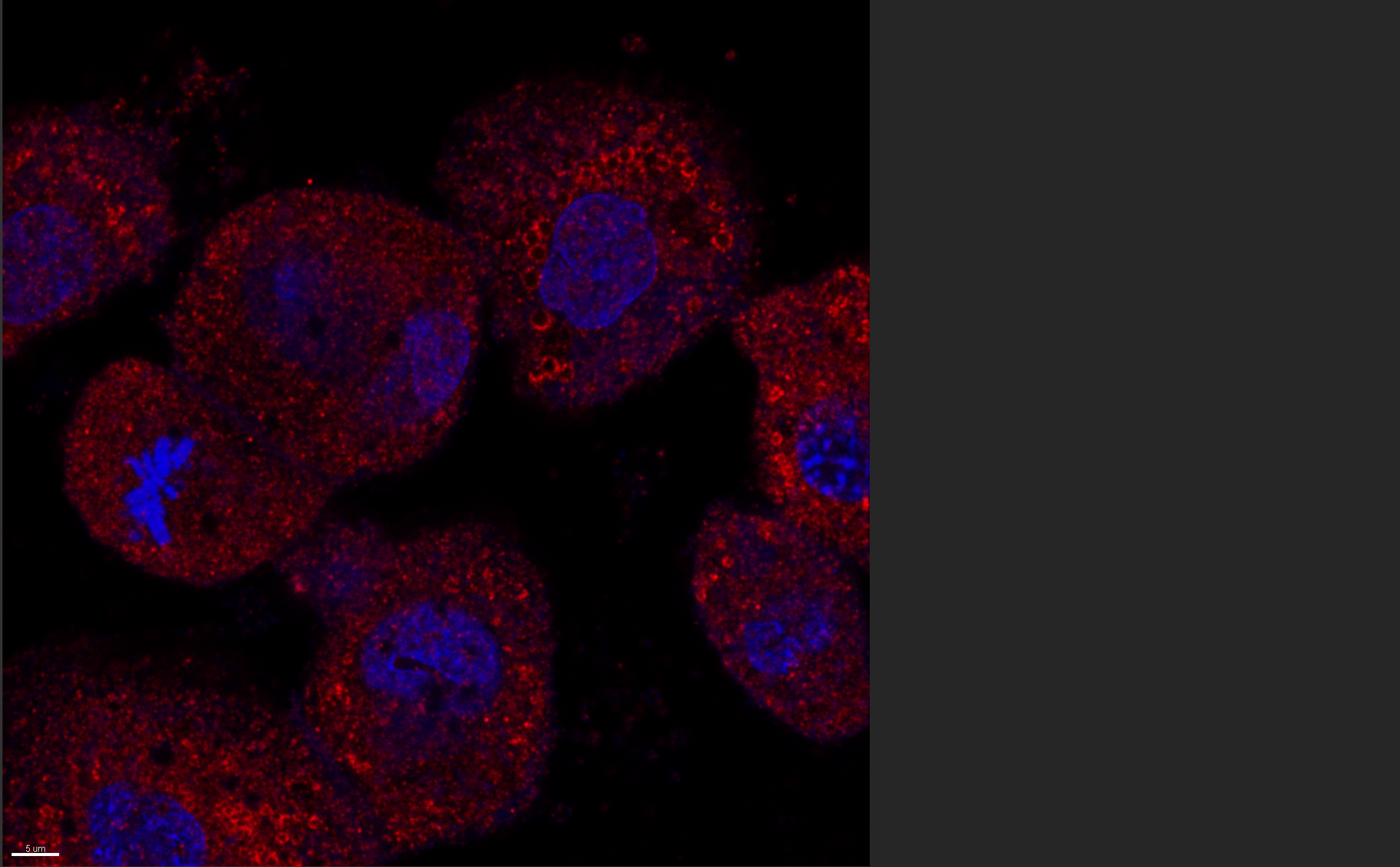

Supplement: Supplementary file 9 — Source data Fig. 7 [file 44318_2026_755_MOESM9_ESM.zip › EMBOJ-2025-121050 Figure 7/Microscopy TIF/7C/7C OL Rab5 full view.tif]

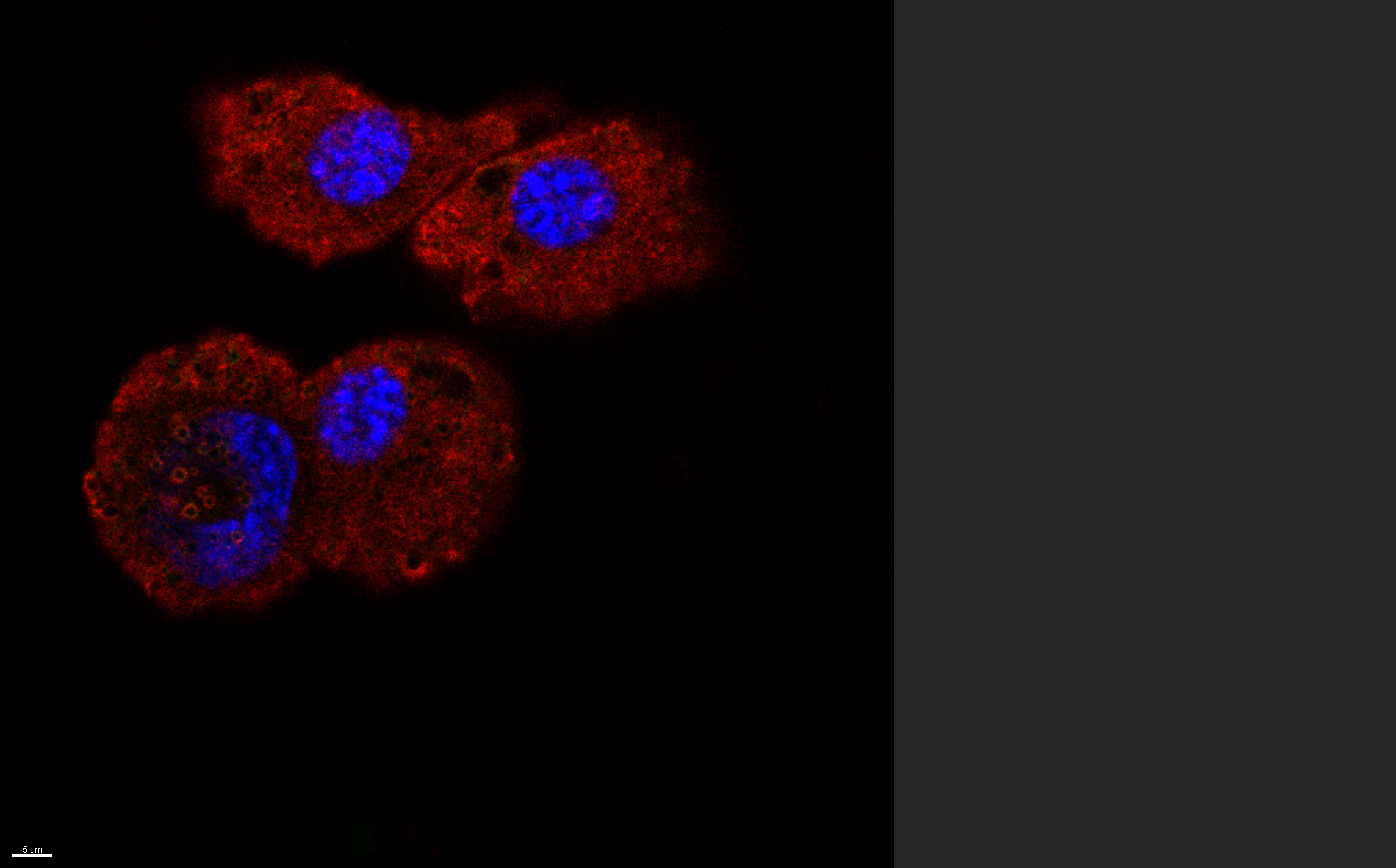

Supplement: Supplementary file 9 — Source data Fig. 7 [file 44318_2026_755_MOESM9_ESM.zip › EMBOJ-2025-121050 Figure 7/Microscopy TIF/7B/7B NLRP3 Full view.tif]

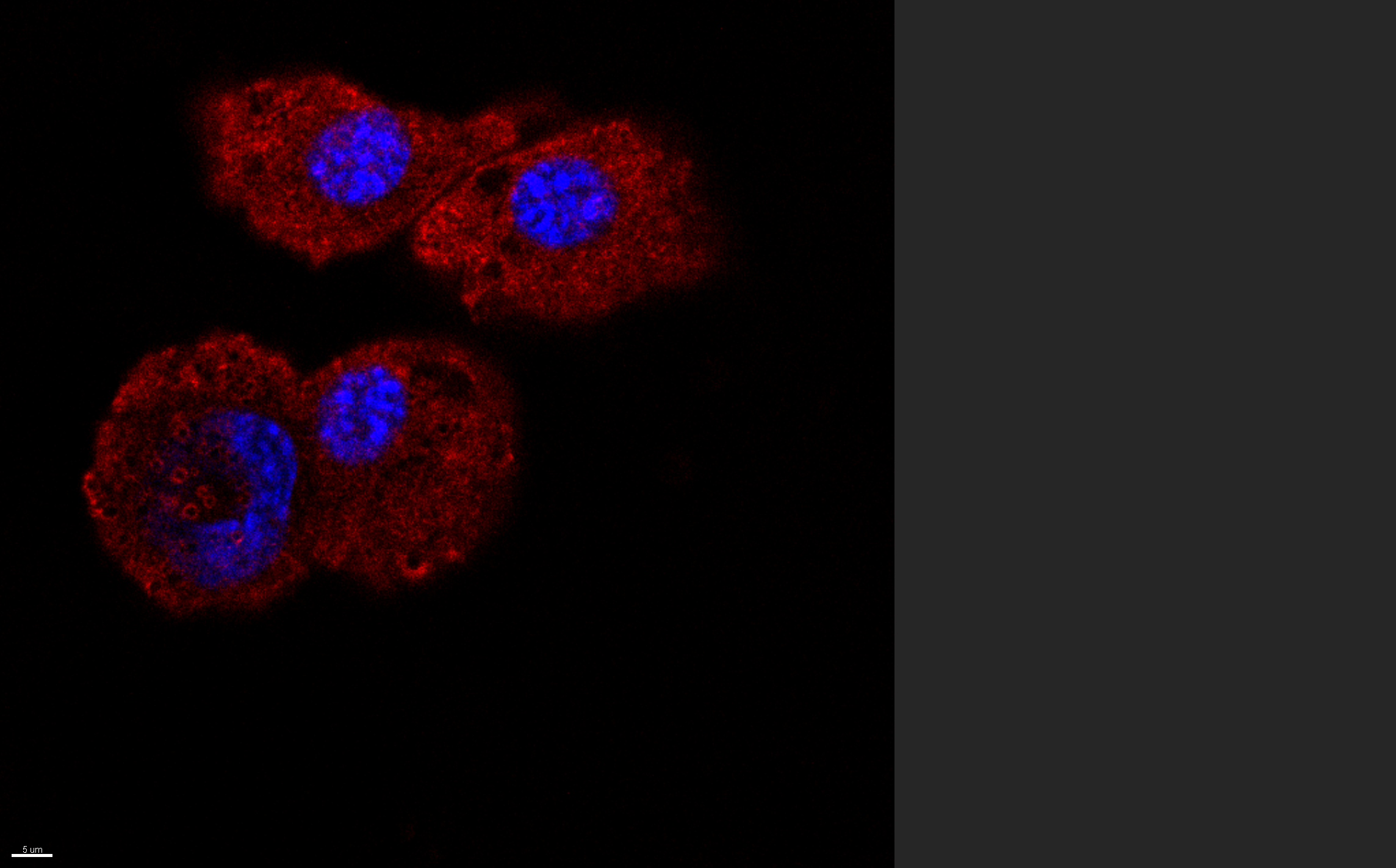

Supplement: Supplementary file 9 — Source data Fig. 7 [file 44318_2026_755_MOESM9_ESM.zip › EMBOJ-2025-121050 Figure 7/Microscopy TIF/7B/7B Rab5 Full view.tif]

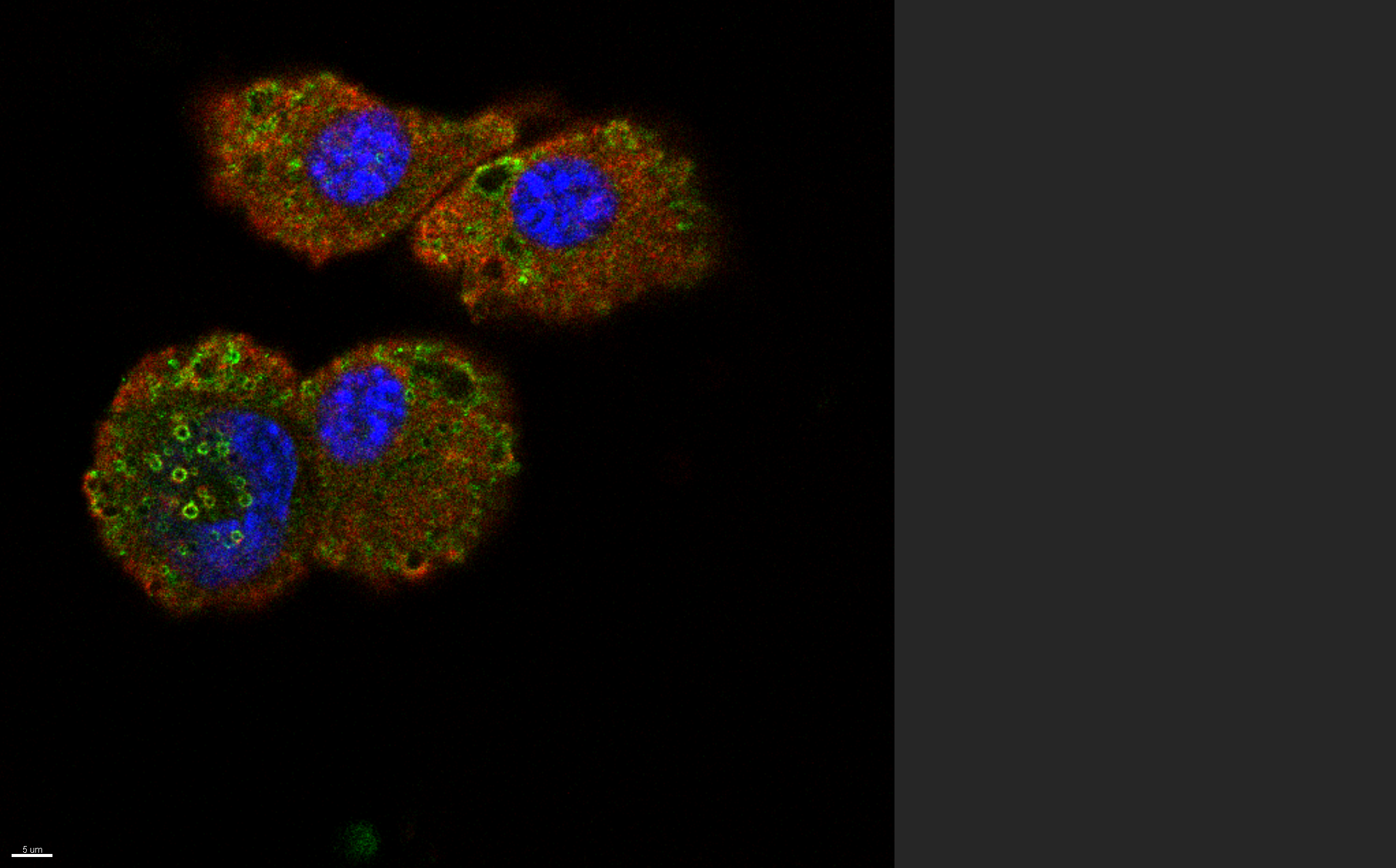

Supplement: Supplementary file 9 — Source data Fig. 7 [file 44318_2026_755_MOESM9_ESM.zip › EMBOJ-2025-121050 Figure 7/Microscopy TIF/7B/7B OL Full view.tif]

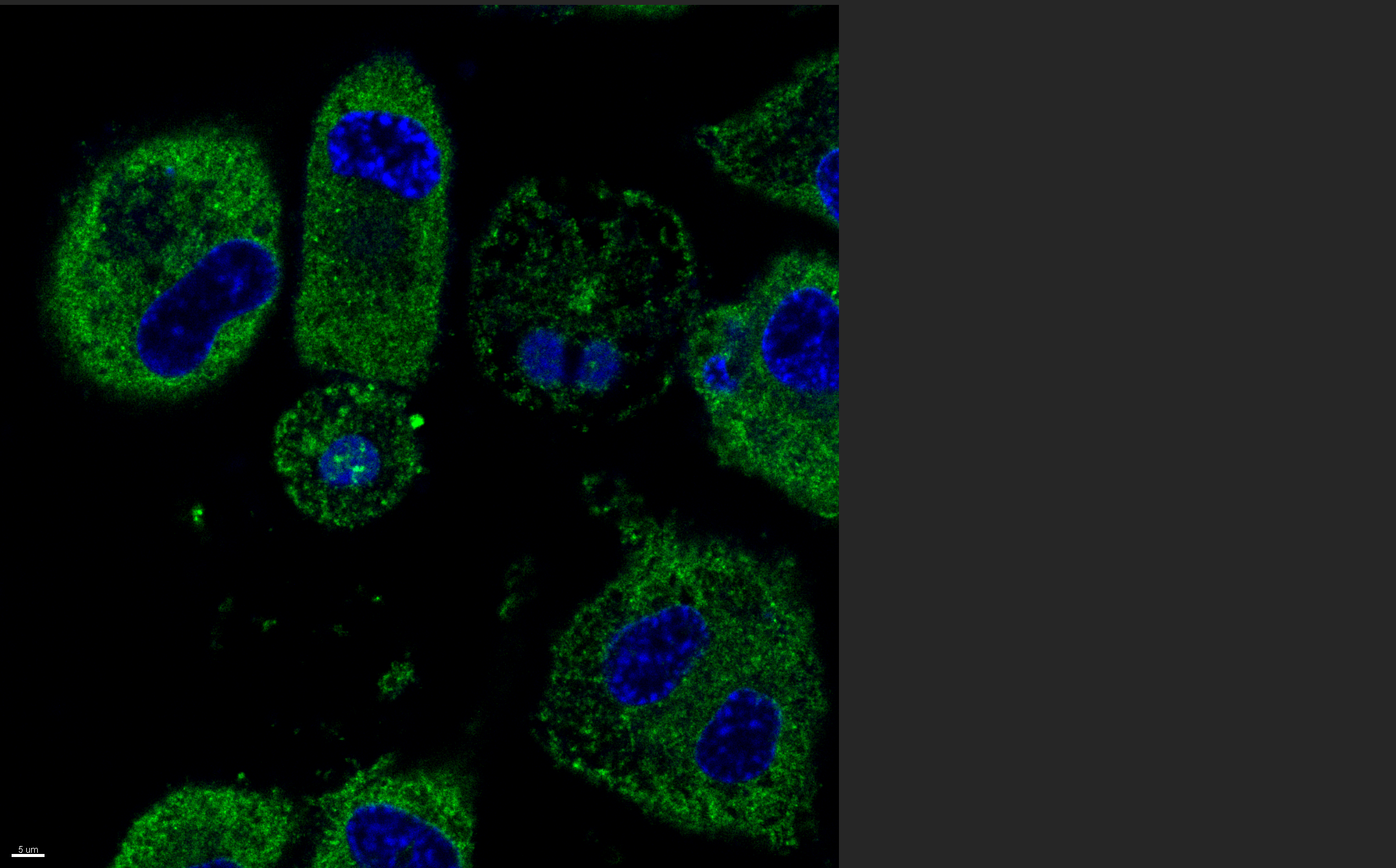

Supplement: Supplementary file 9 — Source data Fig. 7 [file 44318_2026_755_MOESM9_ESM.zip › EMBOJ-2025-121050 Figure 7/Microscopy TIF/7A/7A NLRP3 Full view.tif]

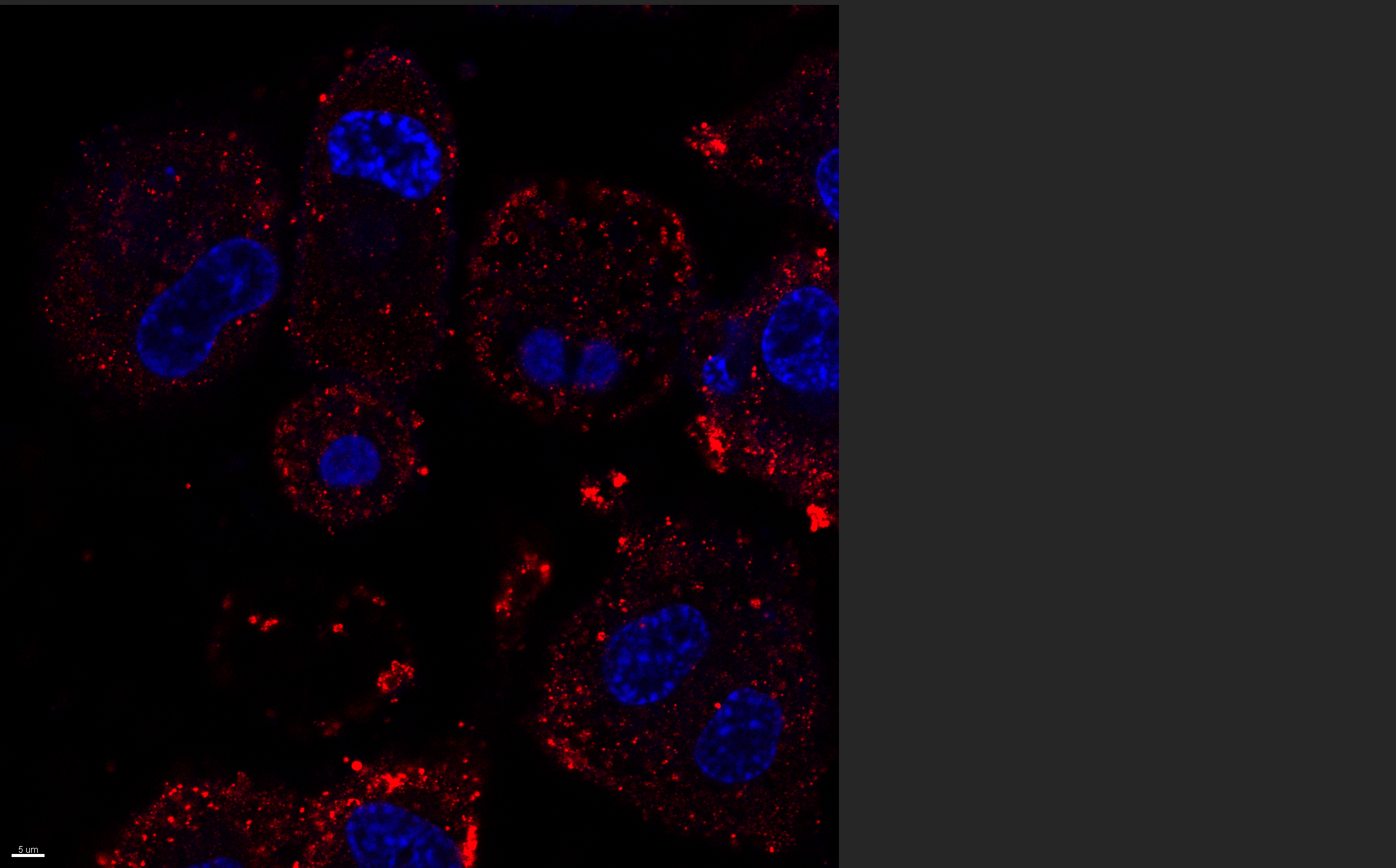

Supplement: Supplementary file 9 — Source data Fig. 7 [file 44318_2026_755_MOESM9_ESM.zip › EMBOJ-2025-121050 Figure 7/Microscopy TIF/7A/7A PI4P full view.tif]

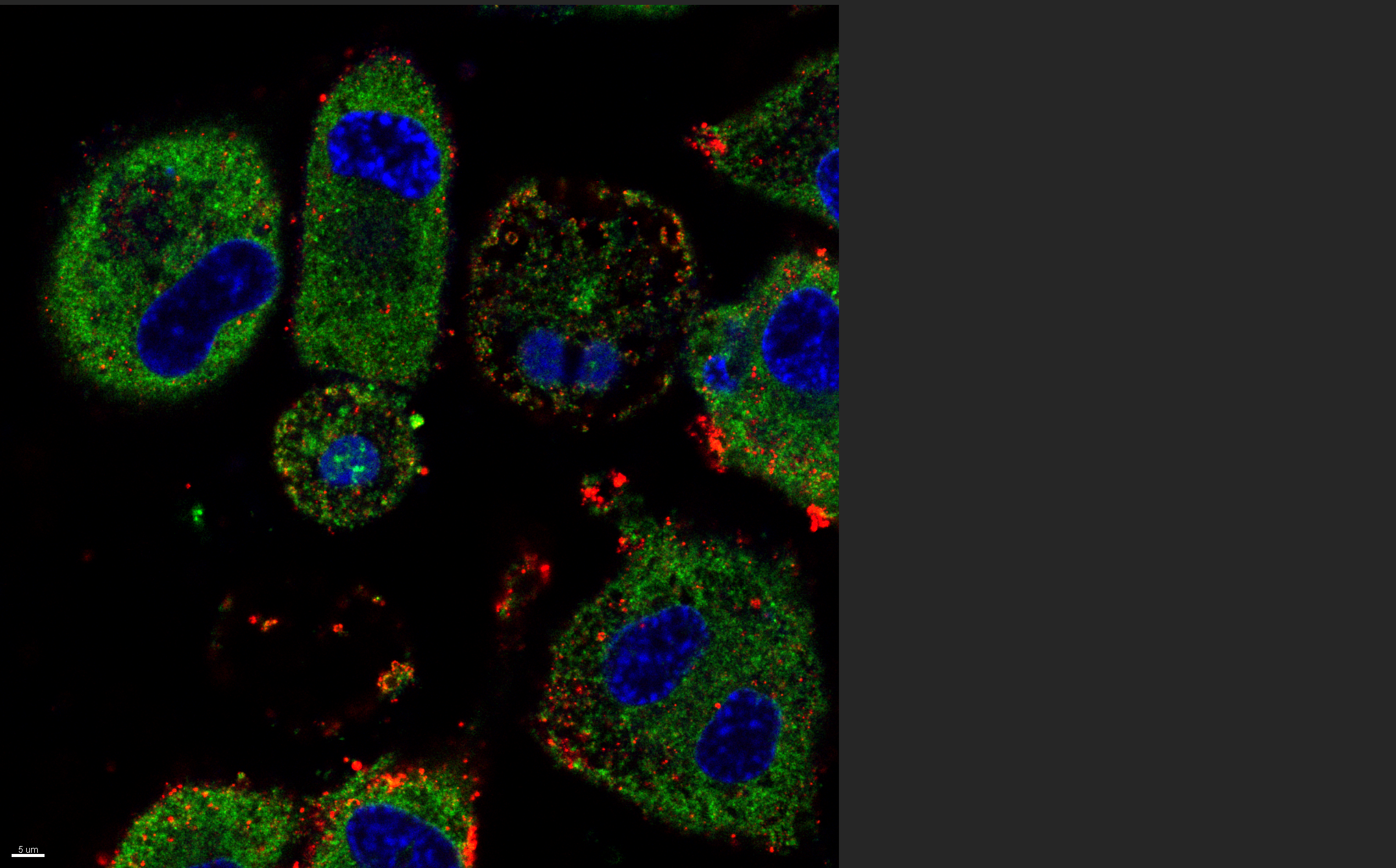

Supplement: Supplementary file 9 — Source data Fig. 7 [file 44318_2026_755_MOESM9_ESM.zip › EMBOJ-2025-121050 Figure 7/Microscopy TIF/7A/7A OL Full view.tif]
