# Supplementary figures and images for: GTPase Rab11b and effector Rab11-FIP2 promote NLRP3 stability during inflammasome priming (part 2 of 2)
Source: EMBO J. 2026 Mar 25;45(9):2991–3021. doi: 10.1038/s44318-026-00755-7 (PMC13144346; doi:10.1038/s44318-026-00755-7)

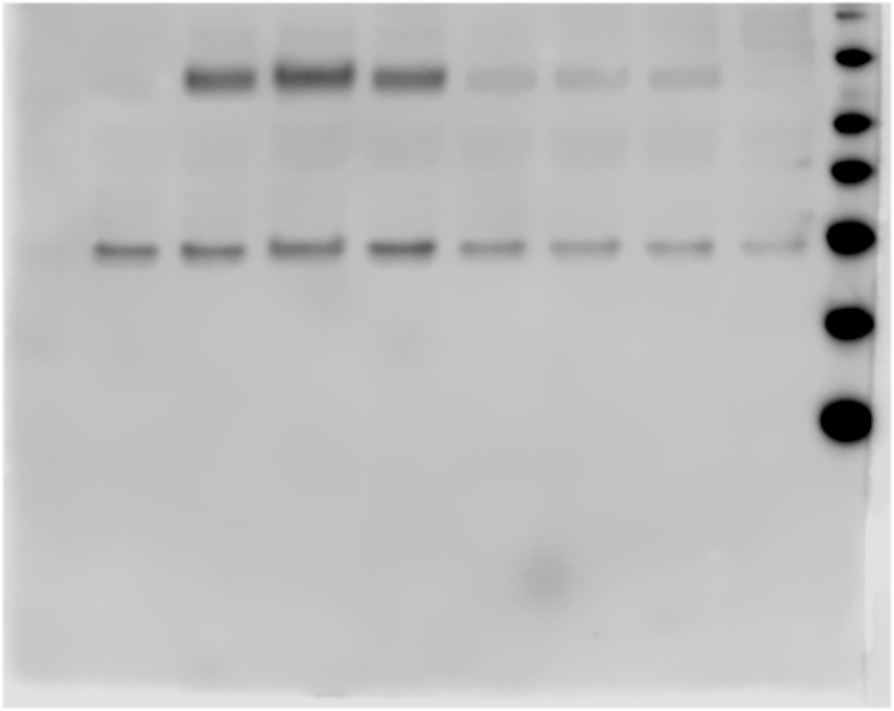

Supplement: Supplementary file 10 — Source data Fig. 8 [file 44318_2026_755_MOESM10_ESM.zip › EMBOJ-2025-121050 Figure 8/Western TIF/8A/Figure 8A p_TAK1 II.tif]

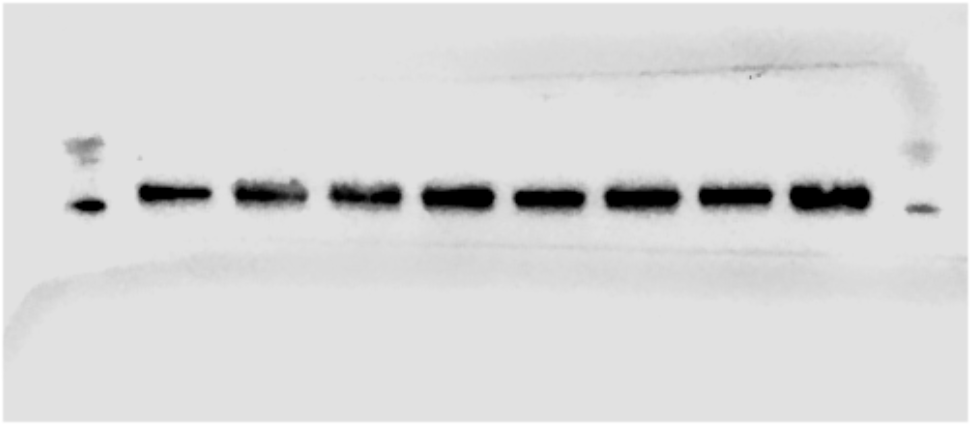

Supplement: Supplementary file 10 — Source data Fig. 8 [file 44318_2026_755_MOESM10_ESM.zip › EMBOJ-2025-121050 Figure 8/Western TIF/8A/Figure 8A tot-IKKbeta.tif]

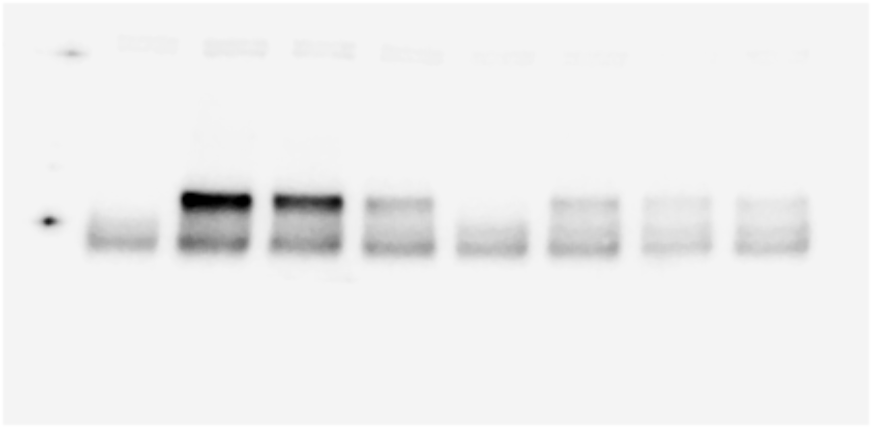

Supplement: Supplementary file 10 — Source data Fig. 8 [file 44318_2026_755_MOESM10_ESM.zip › EMBOJ-2025-121050 Figure 8/Western TIF/8A/Figure 8A p-IKKab.tif]

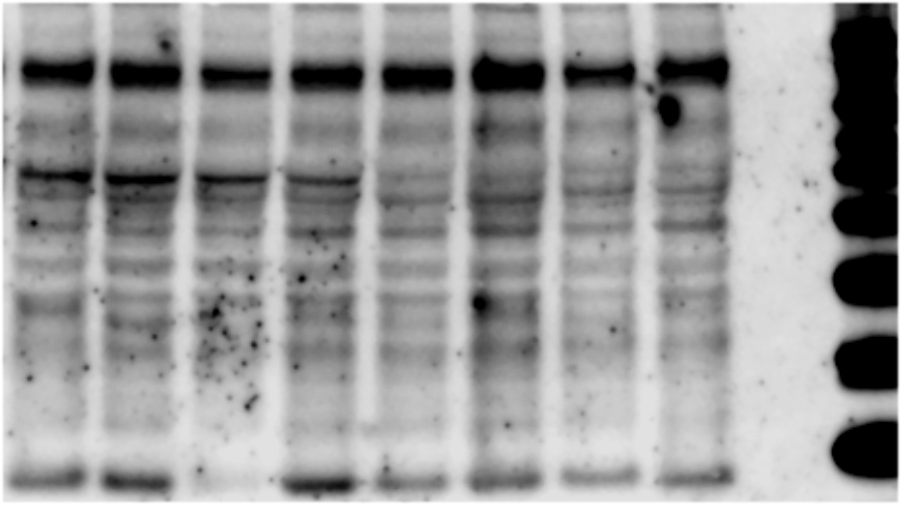

Supplement: Supplementary file 10 — Source data Fig. 8 [file 44318_2026_755_MOESM10_ESM.zip › EMBOJ-2025-121050 Figure 8/Western TIF/8A/Figure 8A FIP2.tif]

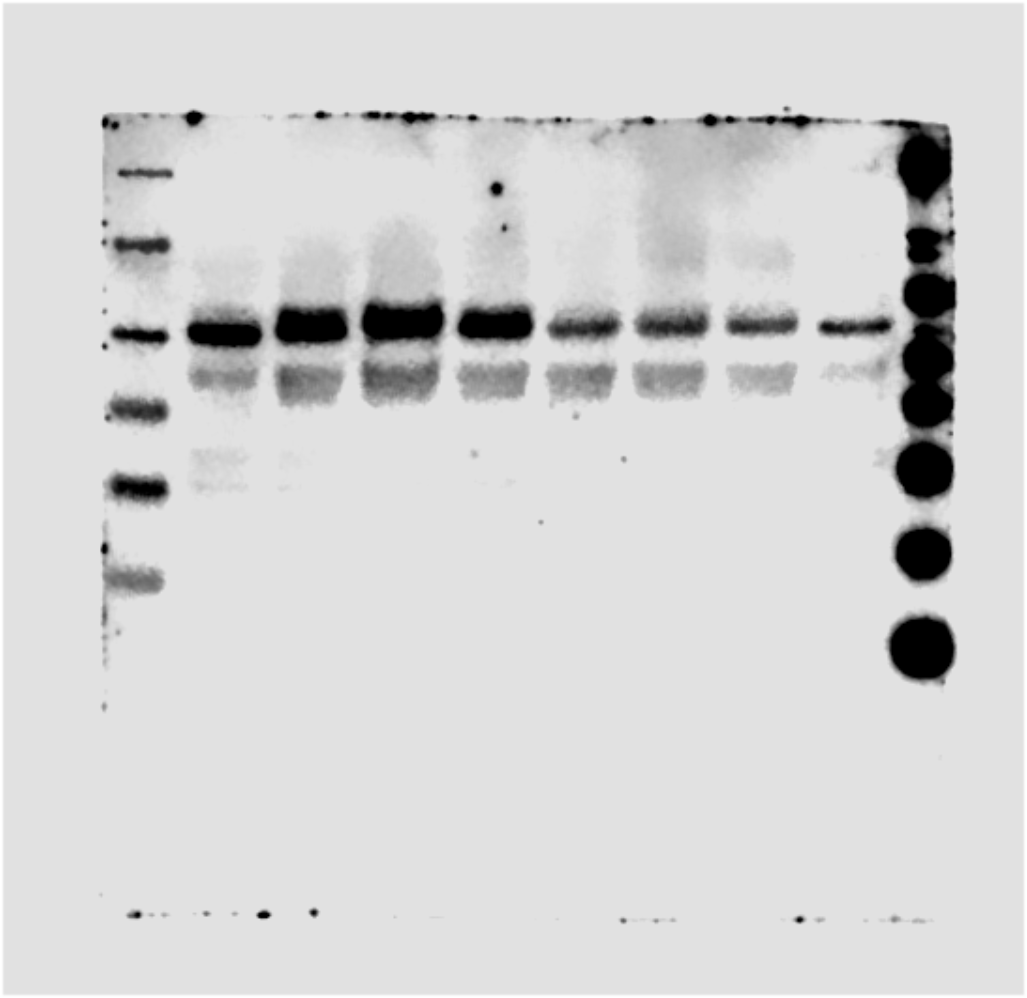

Supplement: Supplementary file 10 — Source data Fig. 8 [file 44318_2026_755_MOESM10_ESM.zip › EMBOJ-2025-121050 Figure 8/Western TIF/8A/Figure 8A tot_TAK1.tif]

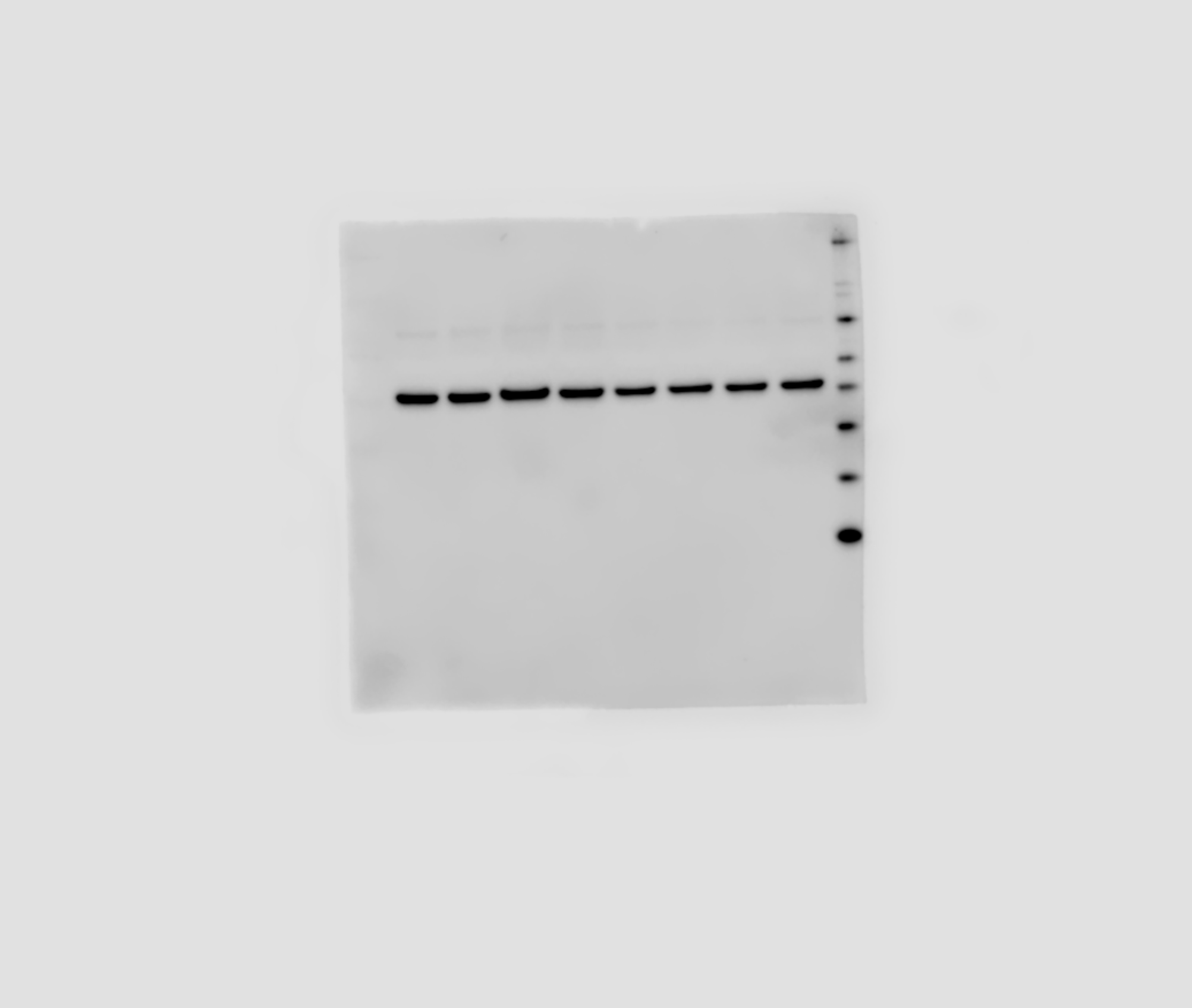

Supplement: Supplementary file 10 — Source data Fig. 8 [file 44318_2026_755_MOESM10_ESM.zip › EMBOJ-2025-121050 Figure 8/Western TIF/8A/Figure 8A beta_tubulin_IKK.tif]

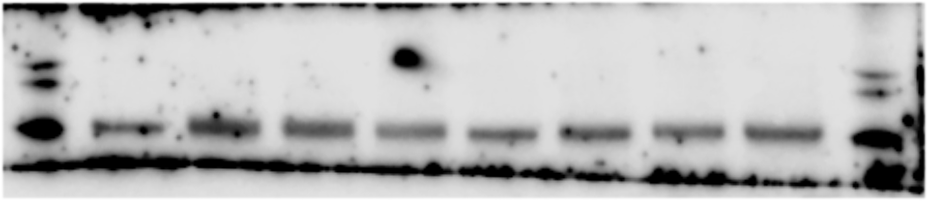

Supplement: Supplementary file 10 — Source data Fig. 8 [file 44318_2026_755_MOESM10_ESM.zip › EMBOJ-2025-121050 Figure 8/Western TIF/8A/Figure 8A tot_IKKalfa.tif]

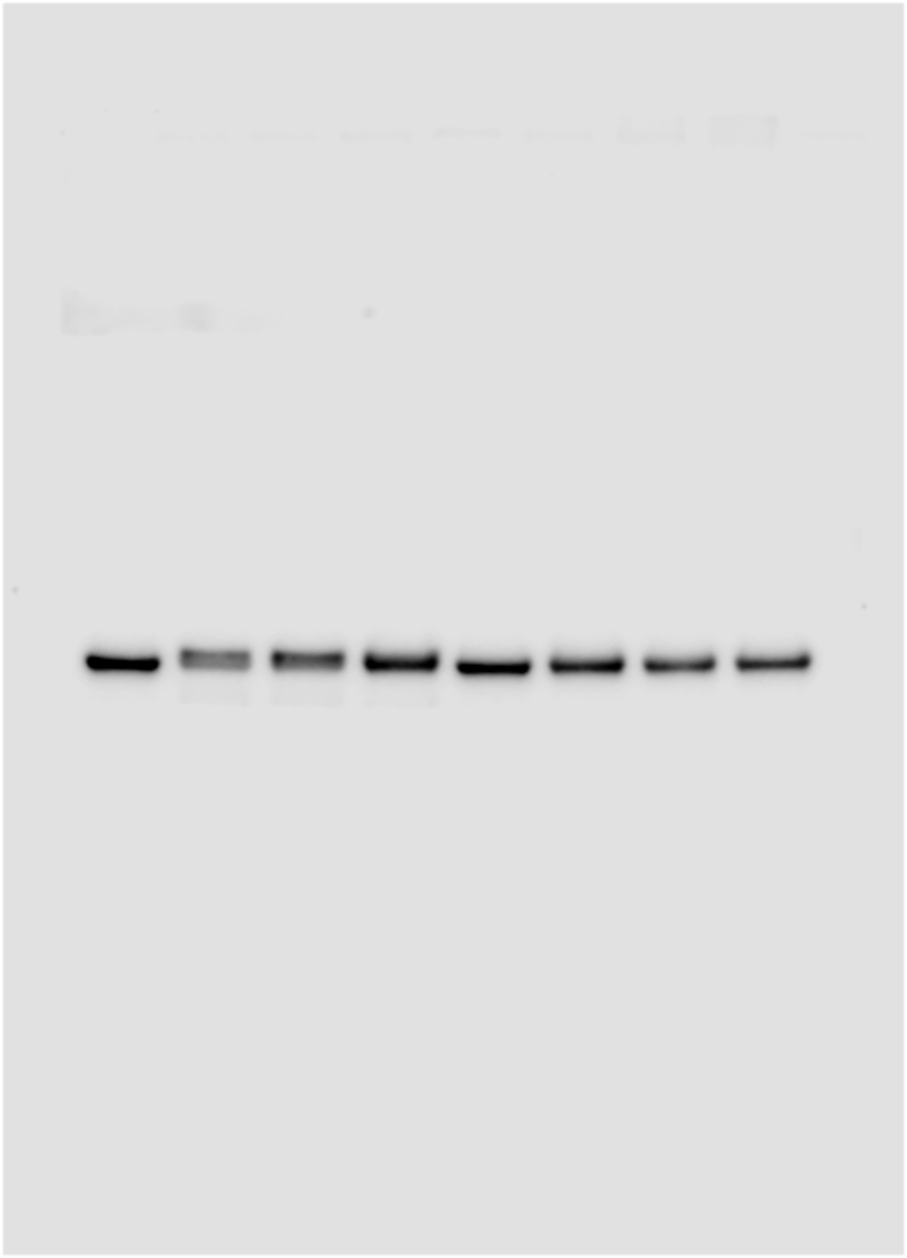

Supplement: Supplementary file 10 — Source data Fig. 8 [file 44318_2026_755_MOESM10_ESM.zip › EMBOJ-2025-121050 Figure 8/Western TIF/8A/Figure 8A tot_IKKbeta.tif]

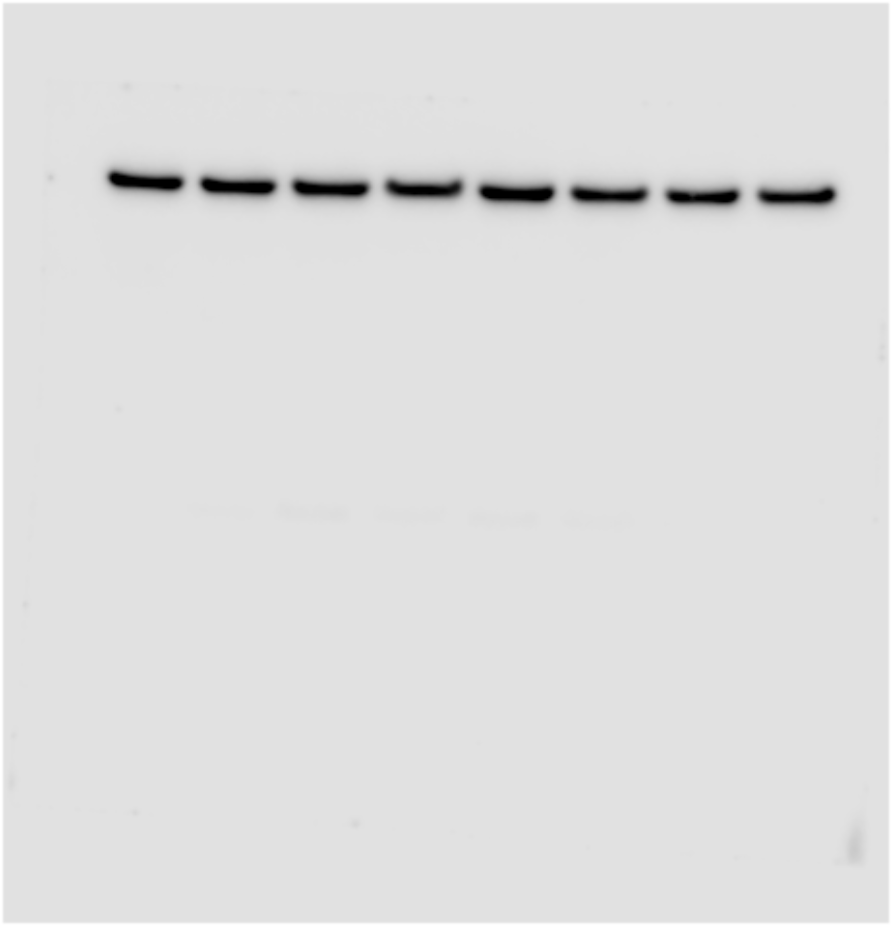

Supplement: Supplementary file 10 — Source data Fig. 8 [file 44318_2026_755_MOESM10_ESM.zip › EMBOJ-2025-121050 Figure 8/Western TIF/8C/Figure 8C beta_tubulin.tif]

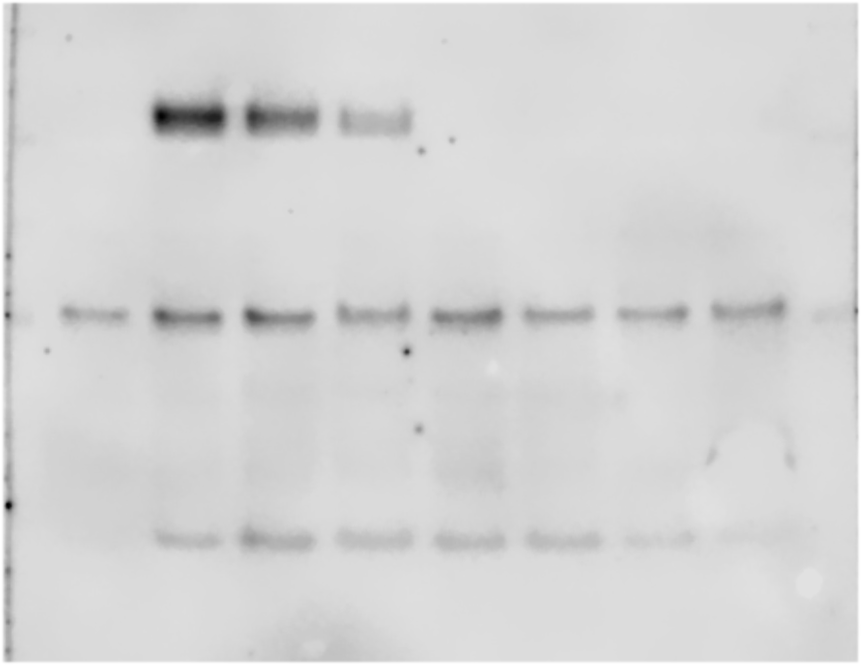

Supplement: Supplementary file 10 — Source data Fig. 8 [file 44318_2026_755_MOESM10_ESM.zip › EMBOJ-2025-121050 Figure 8/Western TIF/8C/Figure 8C p_TAK1.tif]

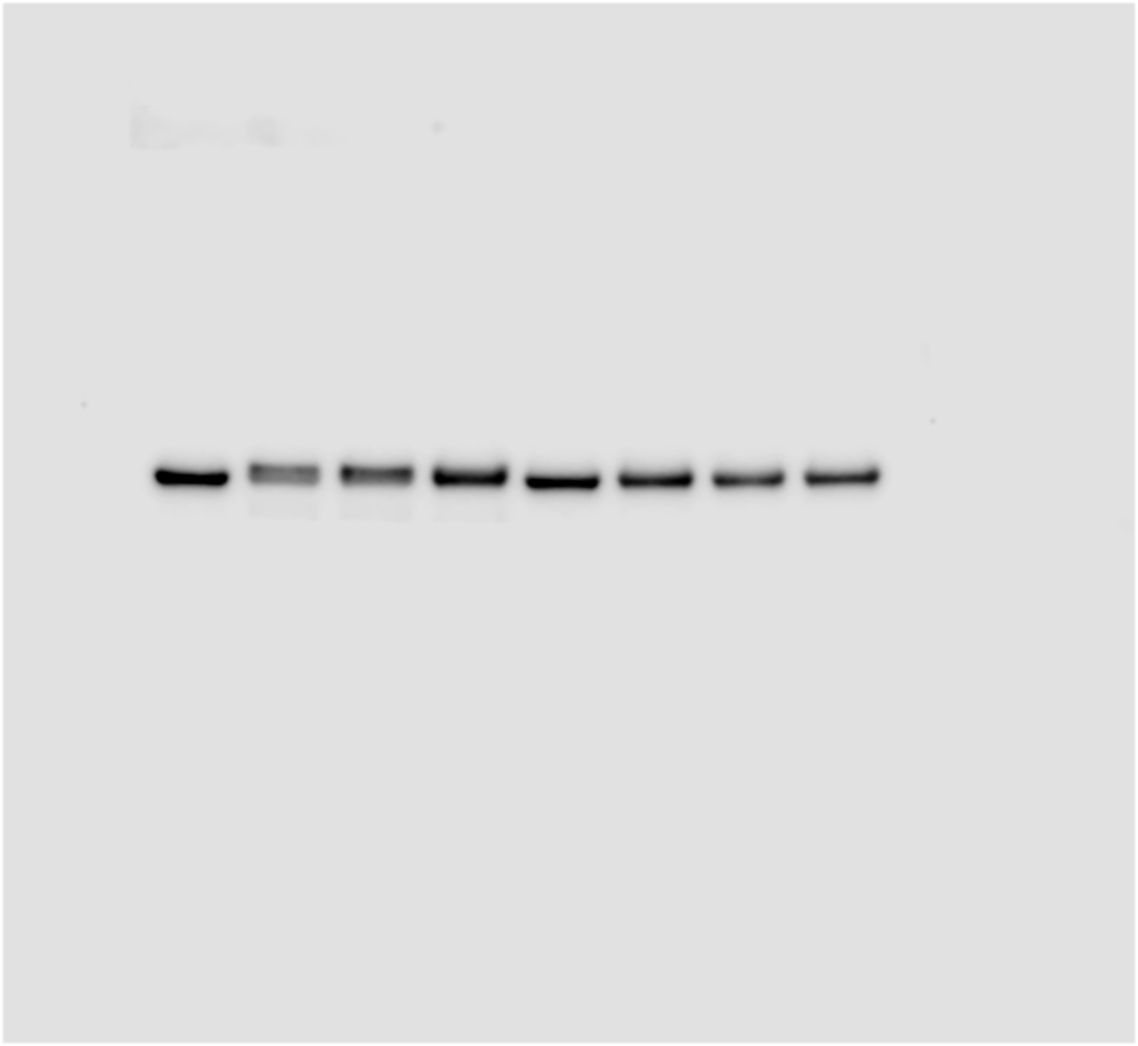

Supplement: Supplementary file 10 — Source data Fig. 8 [file 44318_2026_755_MOESM10_ESM.zip › EMBOJ-2025-121050 Figure 8/Western TIF/8C/Figure 8C anti_IKKbeta.tif]

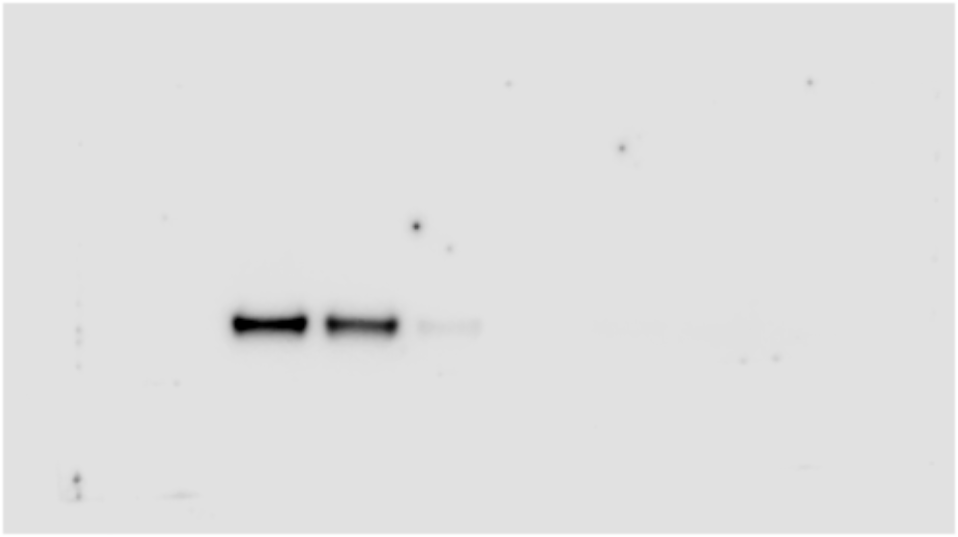

Supplement: Supplementary file 10 — Source data Fig. 8 [file 44318_2026_755_MOESM10_ESM.zip › EMBOJ-2025-121050 Figure 8/Western TIF/8C/Figure 8C p_IKKab.tif]

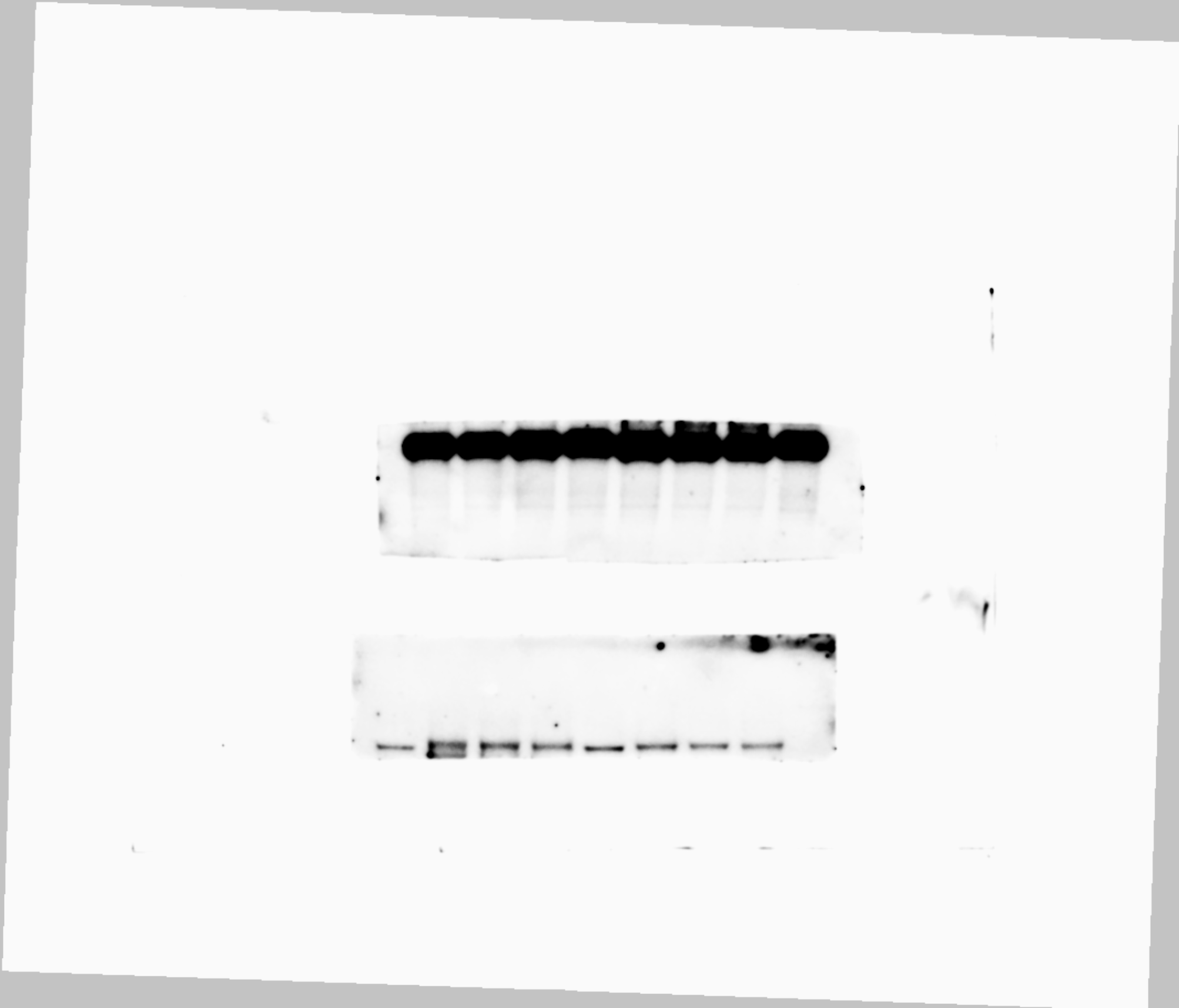

Supplement: Supplementary file 10 — Source data Fig. 8 [file 44318_2026_755_MOESM10_ESM.zip › EMBOJ-2025-121050 Figure 8/Western TIF/8C/Figure 8C anti_IKKalfa.tif]

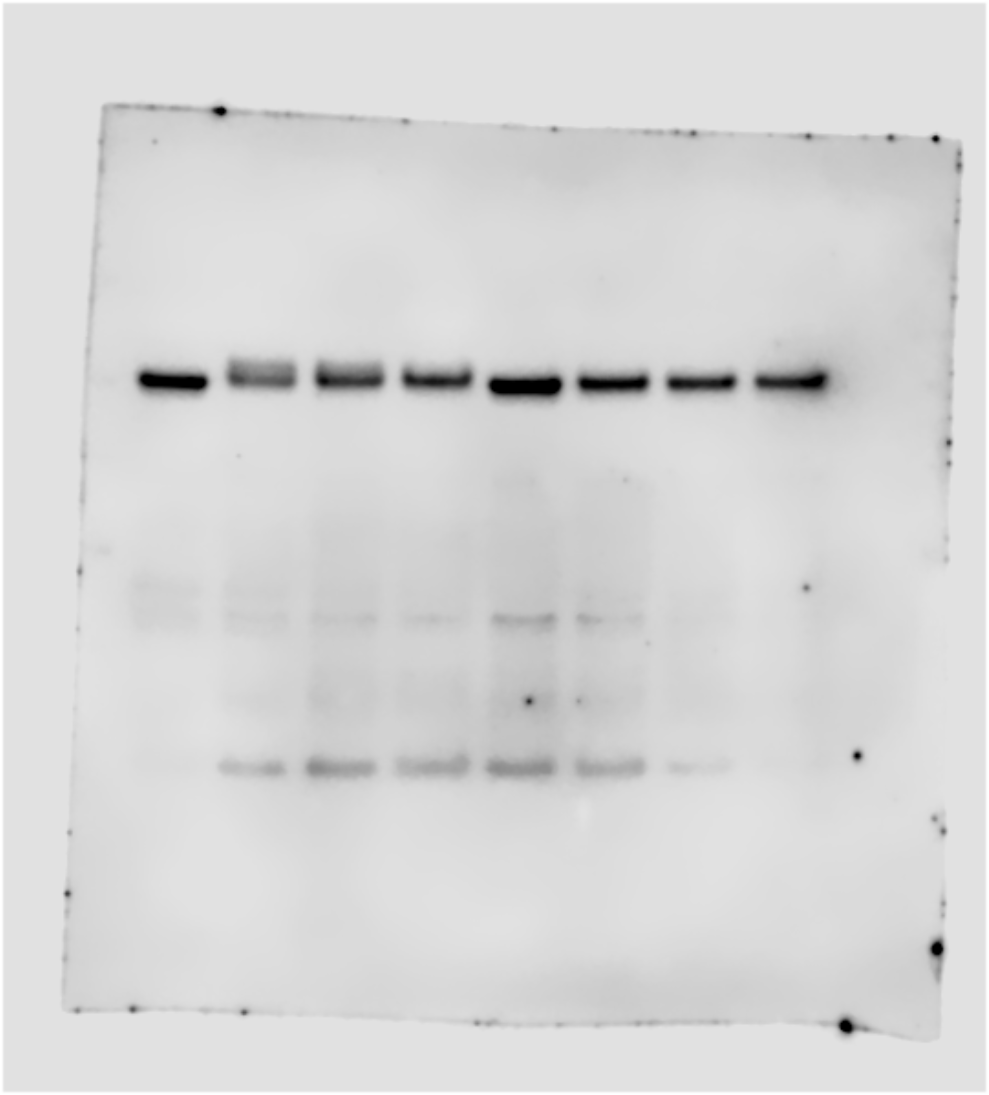

Supplement: Supplementary file 10 — Source data Fig. 8 [file 44318_2026_755_MOESM10_ESM.zip › EMBOJ-2025-121050 Figure 8/Western TIF/8C/Figure 8C Tot_TAK1.tif]

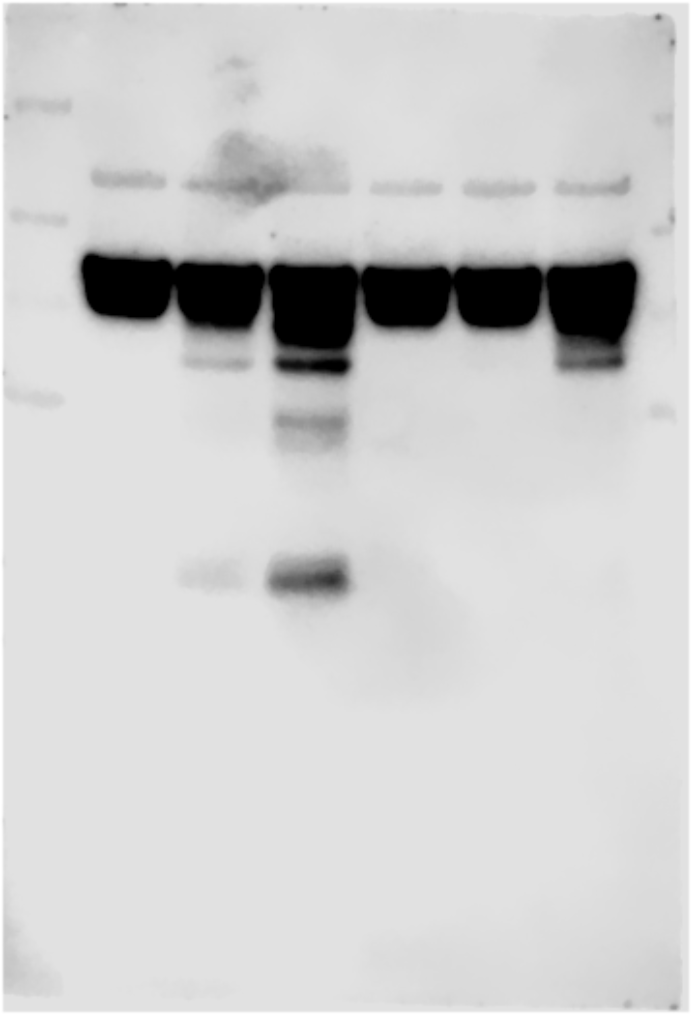

Supplement: Supplementary file 10 — Source data Fig. 8 [file 44318_2026_755_MOESM10_ESM.zip › EMBOJ-2025-121050 Figure 8/Western TIF/8E/Figure 8E casp1.tif]

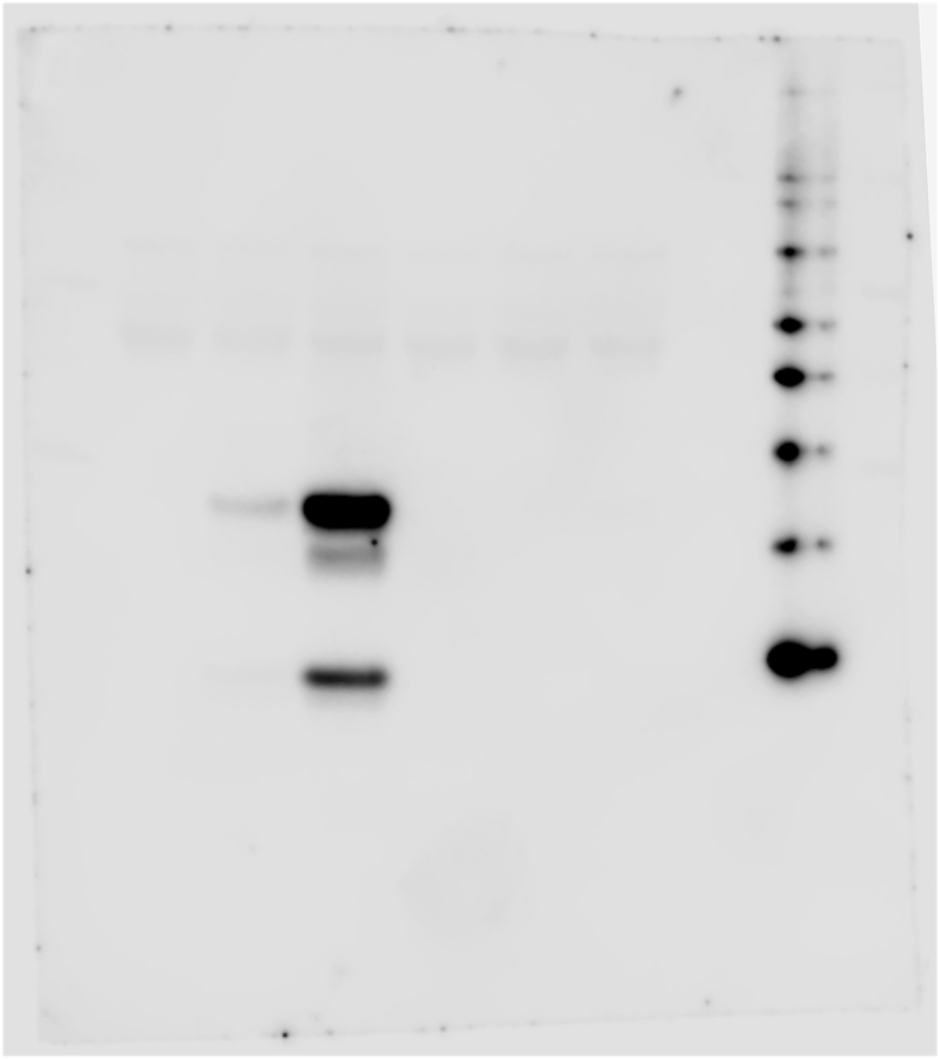

Supplement: Supplementary file 10 — Source data Fig. 8 [file 44318_2026_755_MOESM10_ESM.zip › EMBOJ-2025-121050 Figure 8/Western TIF/8E/Figure 8E IL1beta.tif]

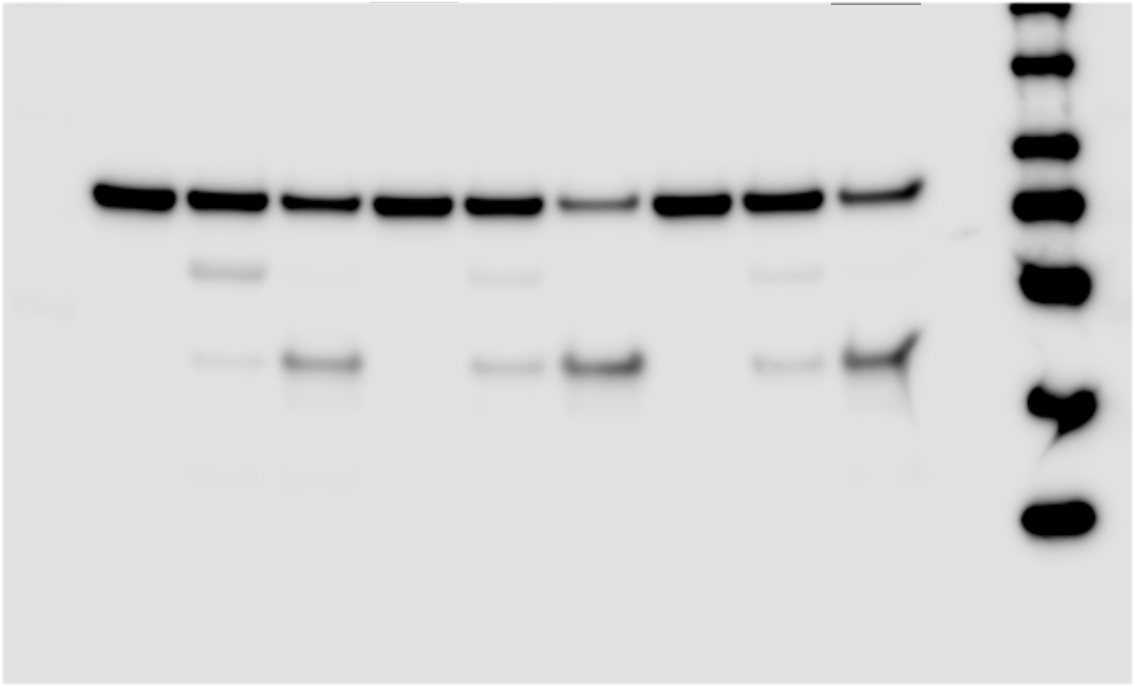

Supplement: Supplementary file 11 — Figure EV1-EV5 Source Data [file 44318_2026_755_MOESM11_ESM.zip › Extended version Figures EV1-EV5/EMBOJ-2025-121050 Figure EV5/Figure EV5 western TIF/Western GSDMD.tif]

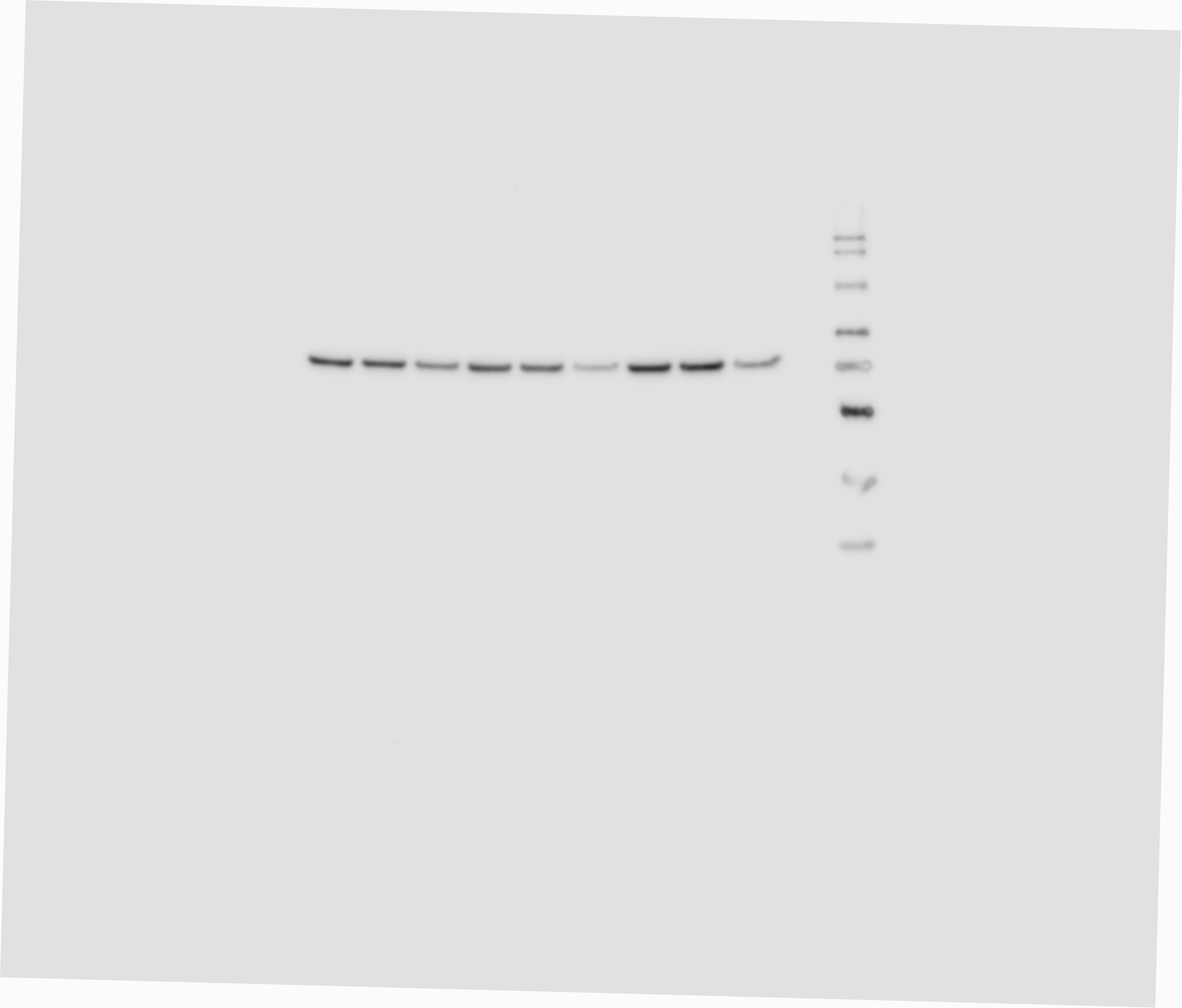

Supplement: Supplementary file 11 — Figure EV1-EV5 Source Data [file 44318_2026_755_MOESM11_ESM.zip › Extended version Figures EV1-EV5/EMBOJ-2025-121050 Figure EV5/Figure EV5 western TIF/Western beta-tubilin.tif]

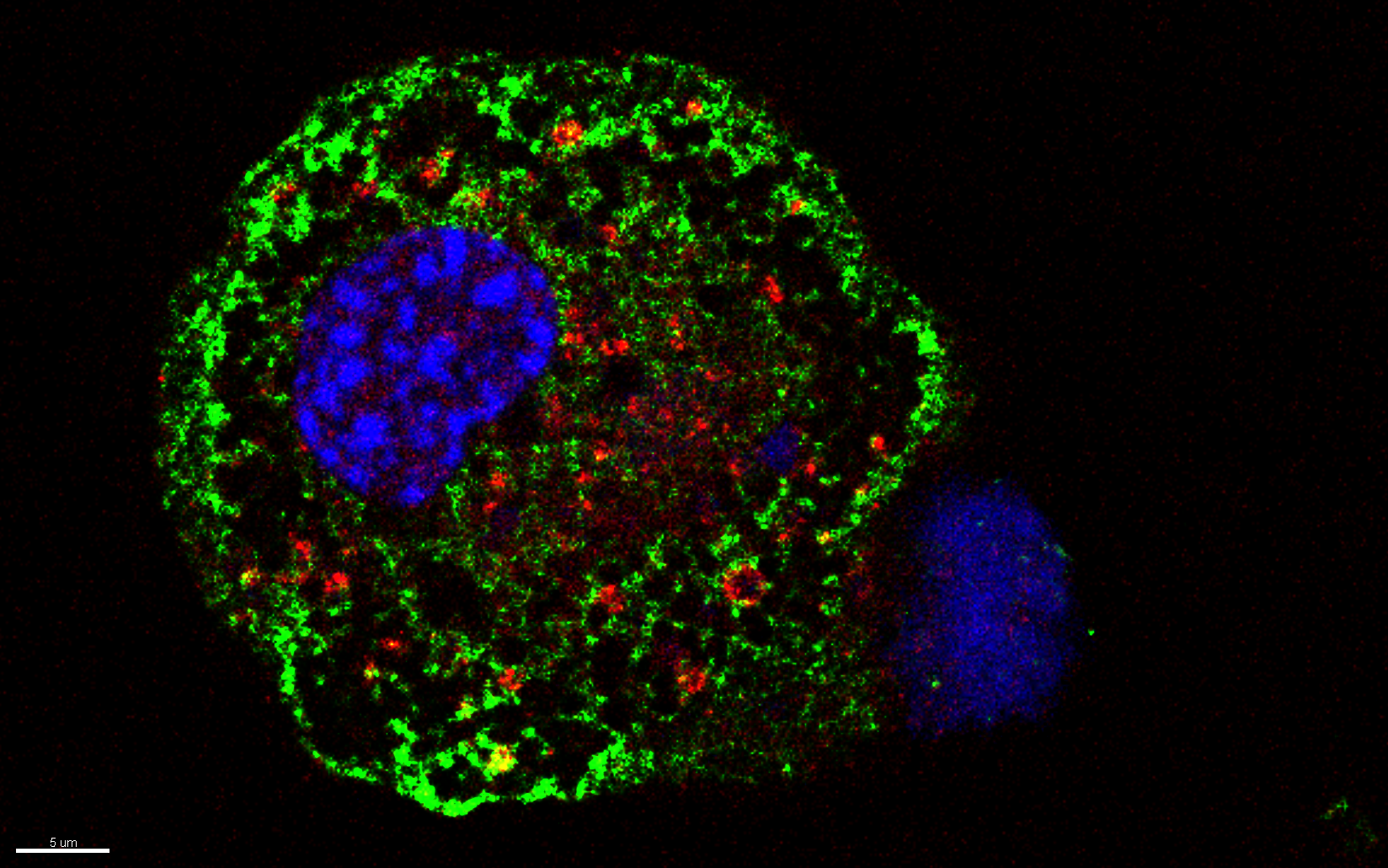

Supplement: Supplementary file 11 — Figure EV1-EV5 Source Data [file 44318_2026_755_MOESM11_ESM.zip › Extended version Figures EV1-EV5/EMBOJ-2025-121050 Figure EV5/EV5B/Microscopy NLRP3_EEA1 .tif]

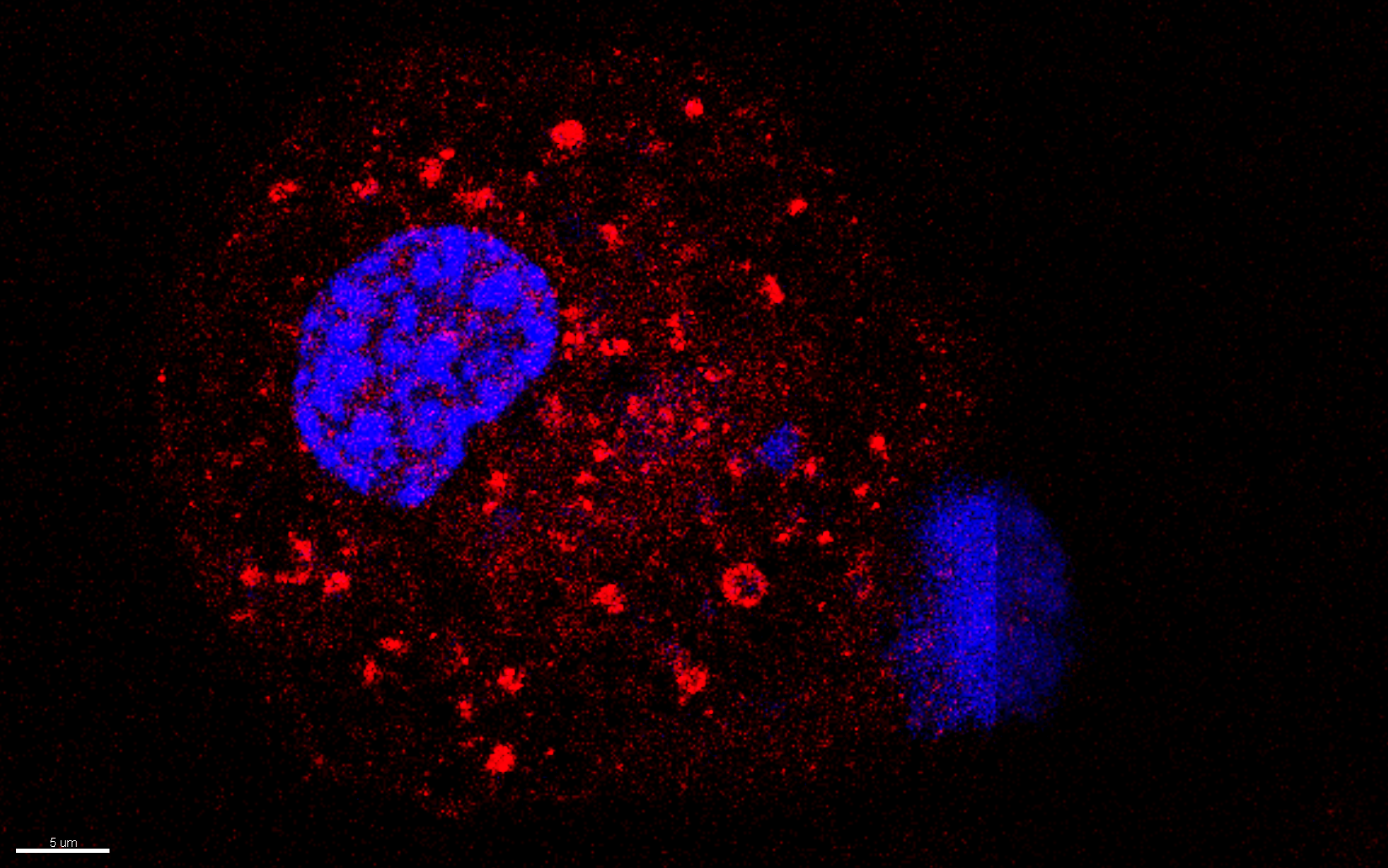

Supplement: Supplementary file 11 — Figure EV1-EV5 Source Data [file 44318_2026_755_MOESM11_ESM.zip › Extended version Figures EV1-EV5/EMBOJ-2025-121050 Figure EV5/EV5B/Microscopy EEA1.tif]

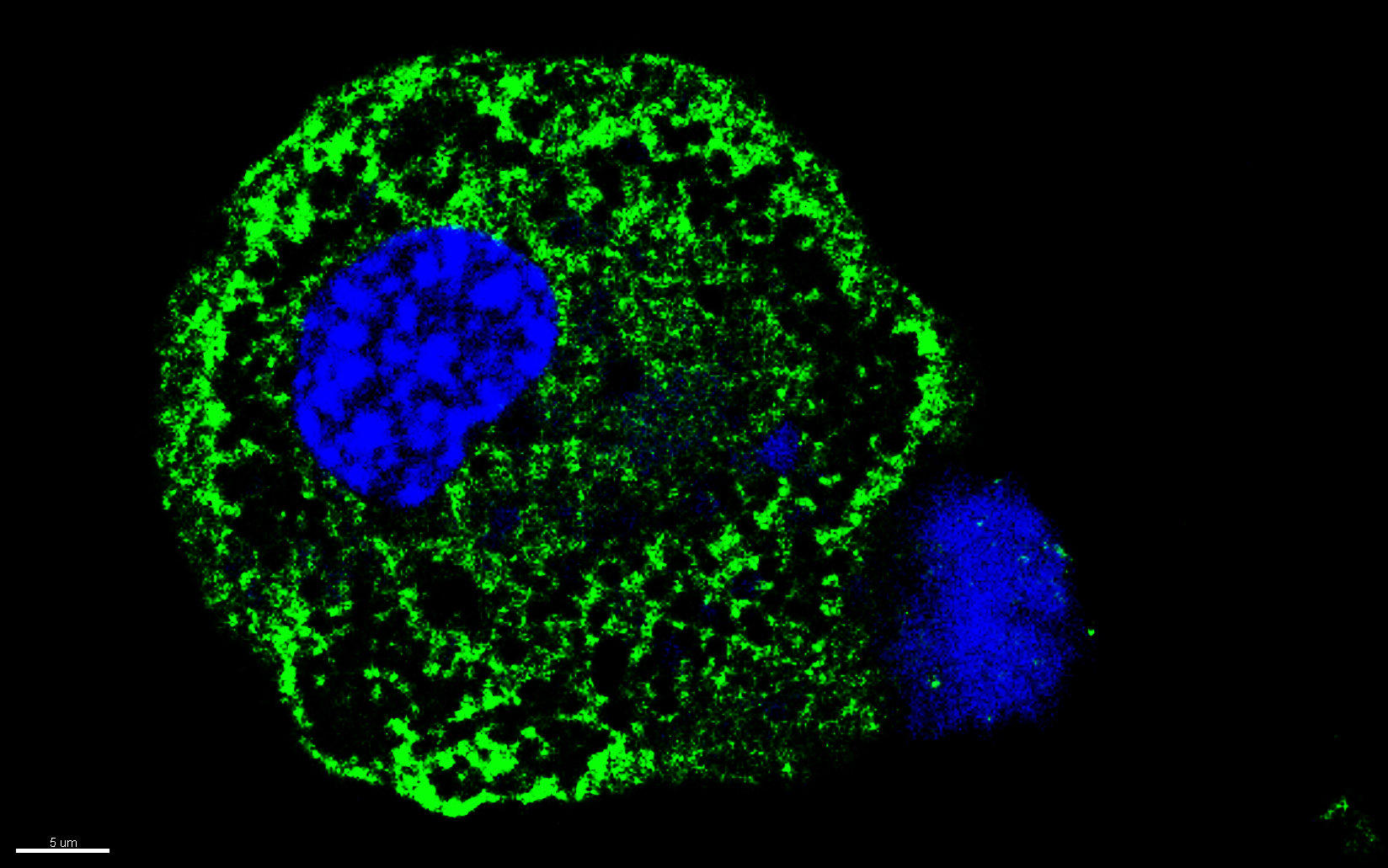

Supplement: Supplementary file 11 — Figure EV1-EV5 Source Data [file 44318_2026_755_MOESM11_ESM.zip › Extended version Figures EV1-EV5/EMBOJ-2025-121050 Figure EV5/EV5B/Microscopy NLRP3.tif]

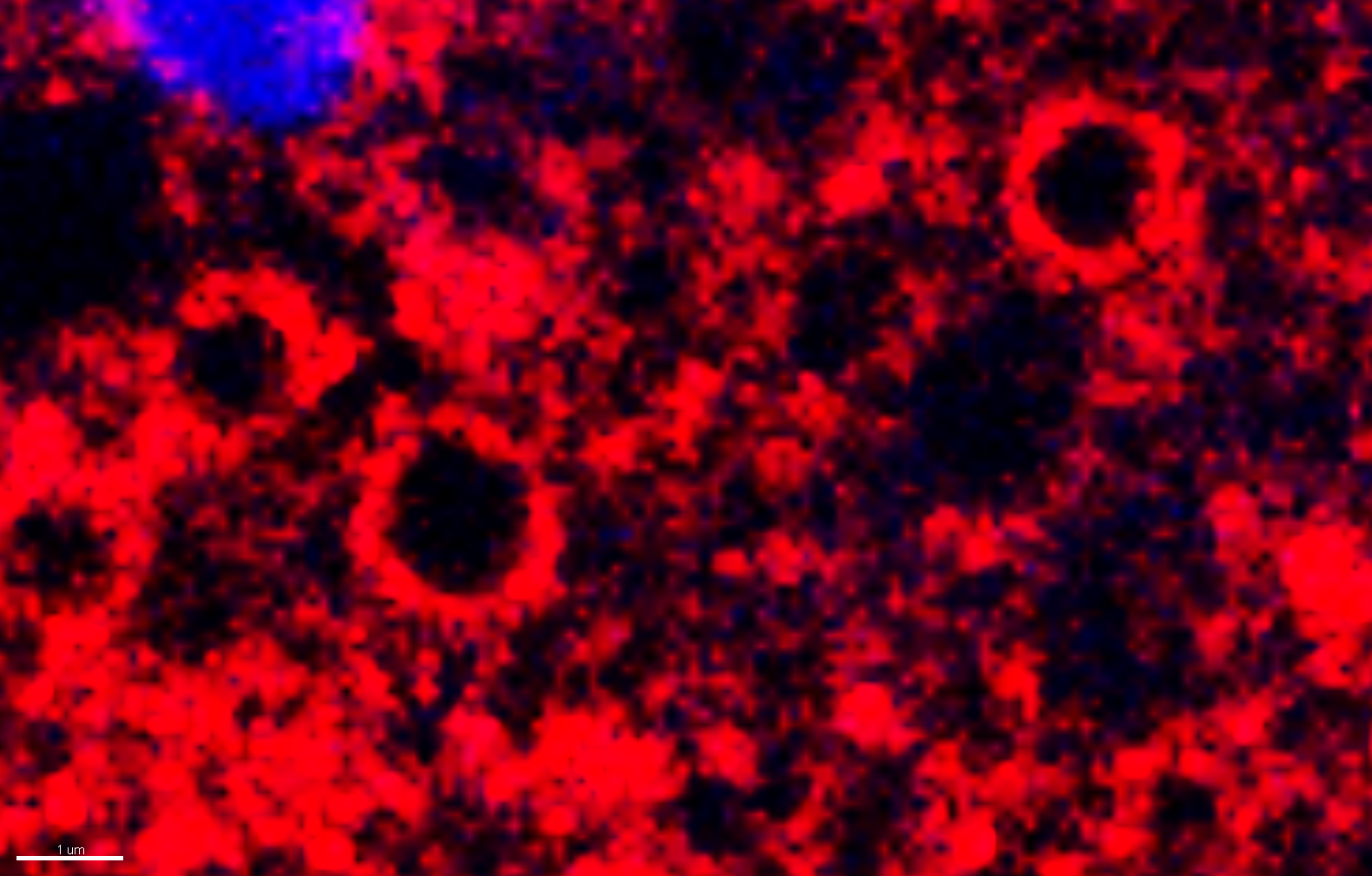

Supplement: Supplementary file 11 — Figure EV1-EV5 Source Data [file 44318_2026_755_MOESM11_ESM.zip › Extended version Figures EV1-EV5/EMBOJ-2025-121050 Figure EV5/EV5E/Microscopy Flag-Rab11b_Zoom.tif]

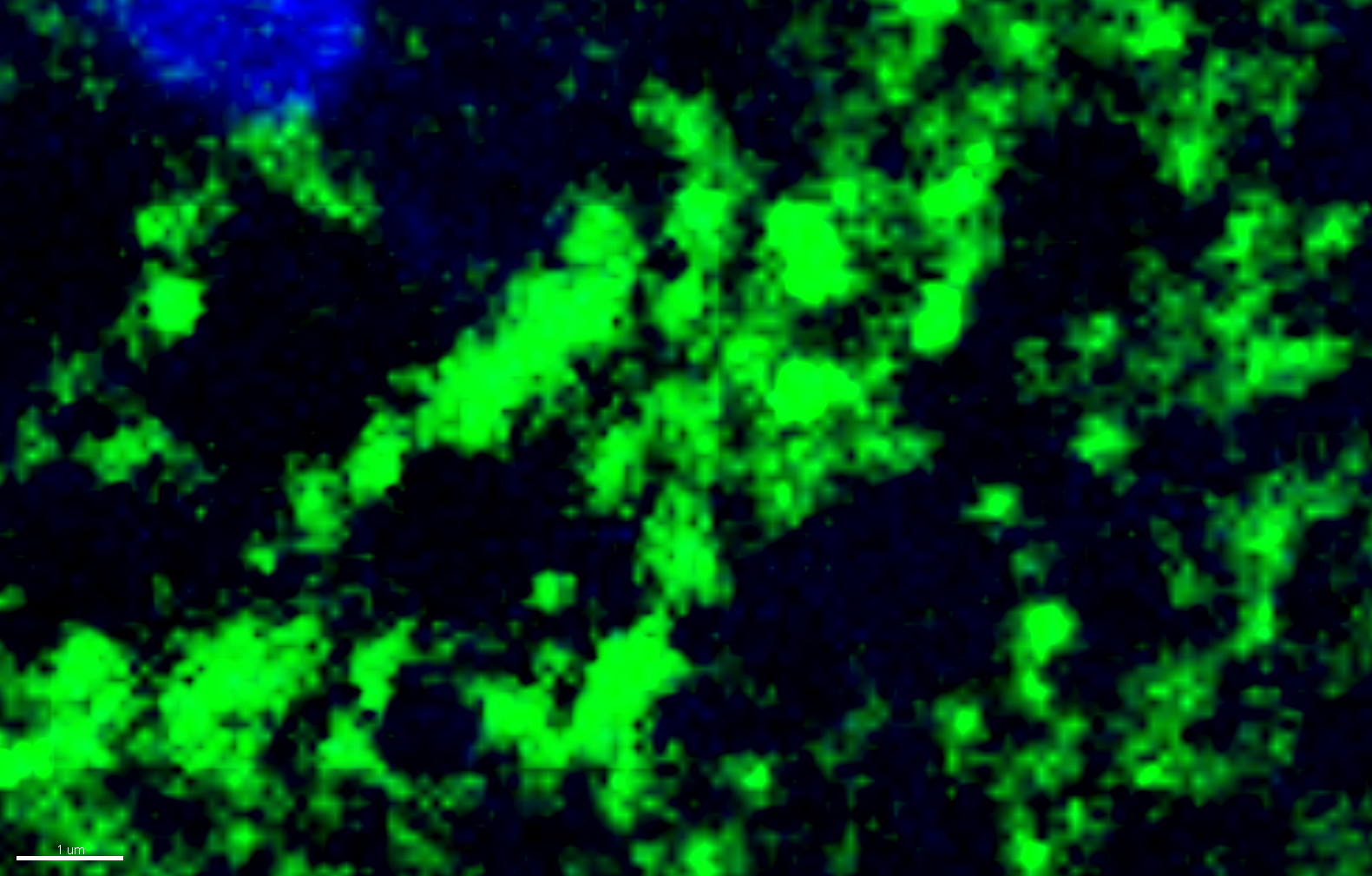

Supplement: Supplementary file 11 — Figure EV1-EV5 Source Data [file 44318_2026_755_MOESM11_ESM.zip › Extended version Figures EV1-EV5/EMBOJ-2025-121050 Figure EV5/EV5E/Microscopy NLRP3_ Zoom.tif]

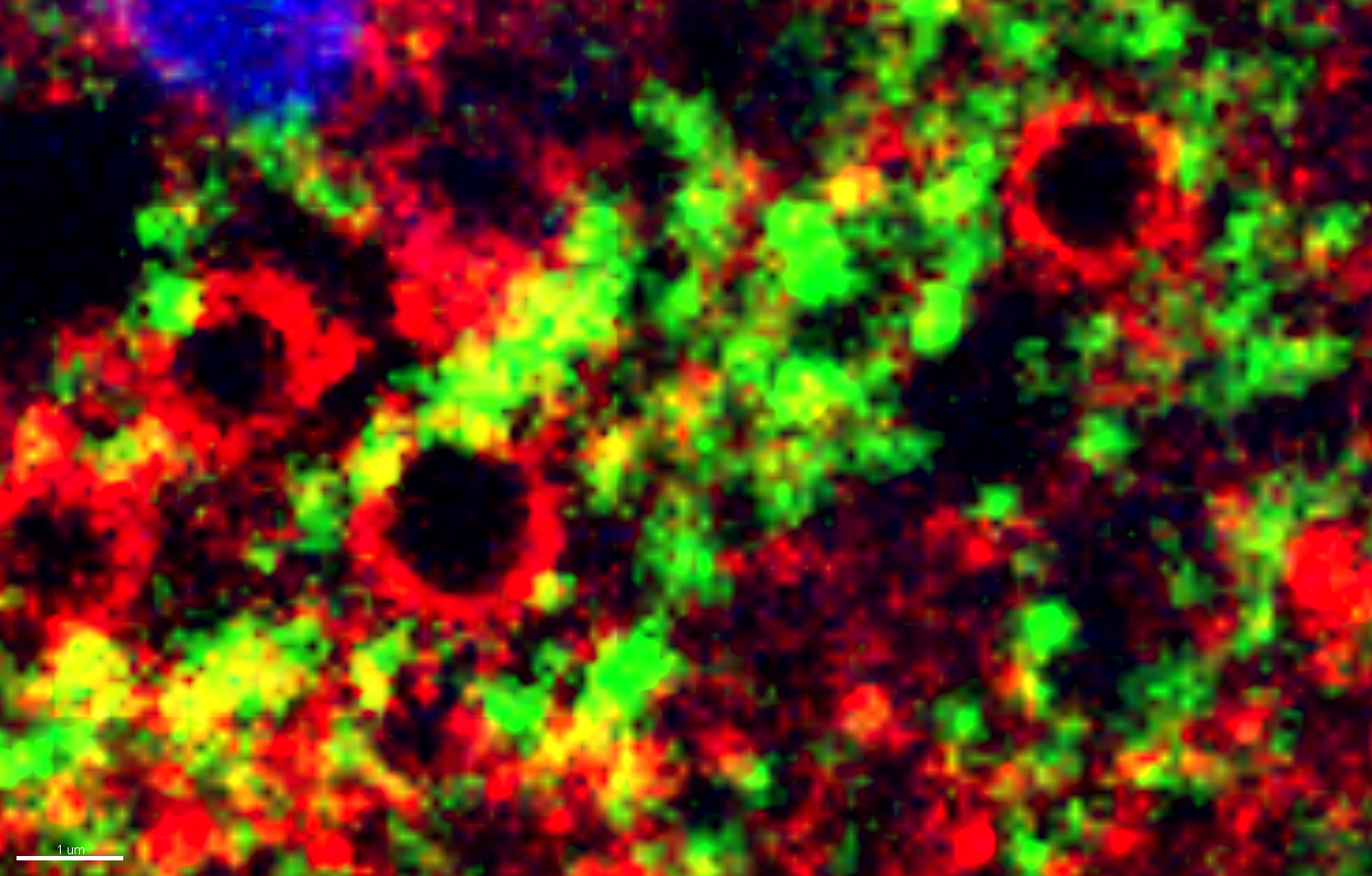

Supplement: Supplementary file 11 — Figure EV1-EV5 Source Data [file 44318_2026_755_MOESM11_ESM.zip › Extended version Figures EV1-EV5/EMBOJ-2025-121050 Figure EV5/EV5E/Microscopy NLRP3_Flag-Rab11b Zoom.tif]

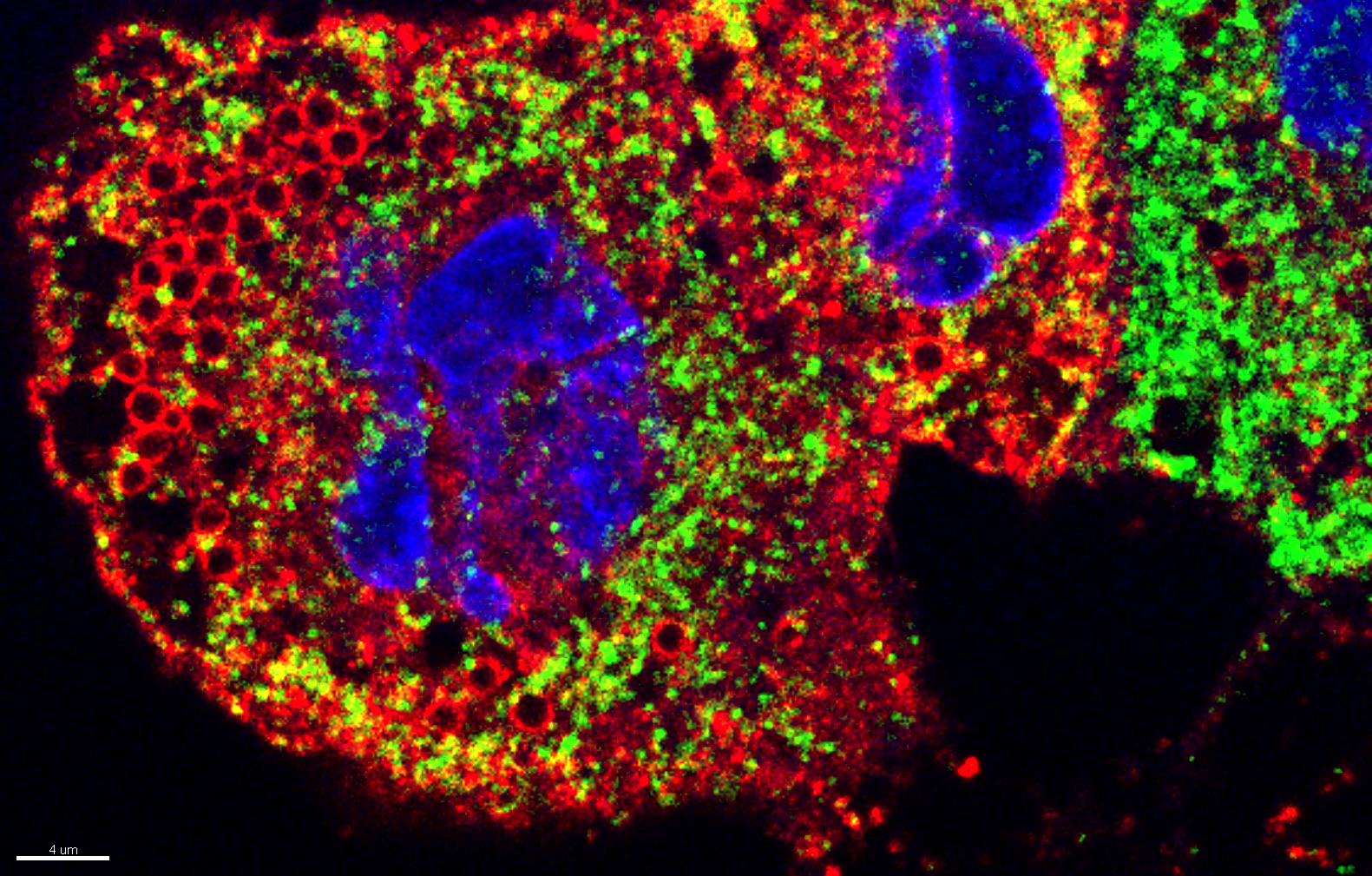

Supplement: Supplementary file 11 — Figure EV1-EV5 Source Data [file 44318_2026_755_MOESM11_ESM.zip › Extended version Figures EV1-EV5/EMBOJ-2025-121050 Figure EV5/EV5E/Microscopy Flag-Rab11b.tif]

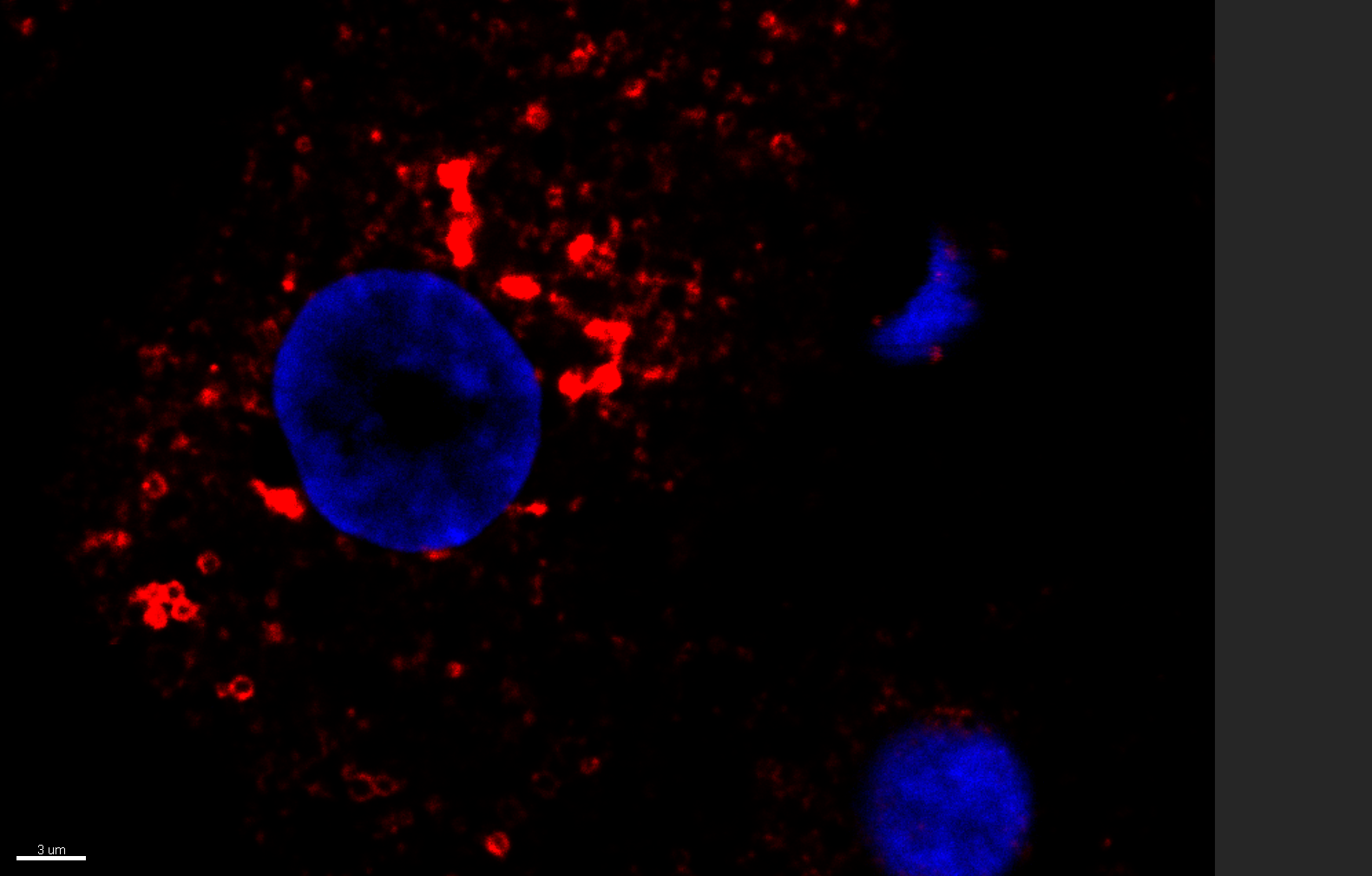

Supplement: Supplementary file 11 — Figure EV1-EV5 Source Data [file 44318_2026_755_MOESM11_ESM.zip › Extended version Figures EV1-EV5/EMBOJ-2025-121050 Figure EV5/EV5D/Microscopy Rab11b.tif]

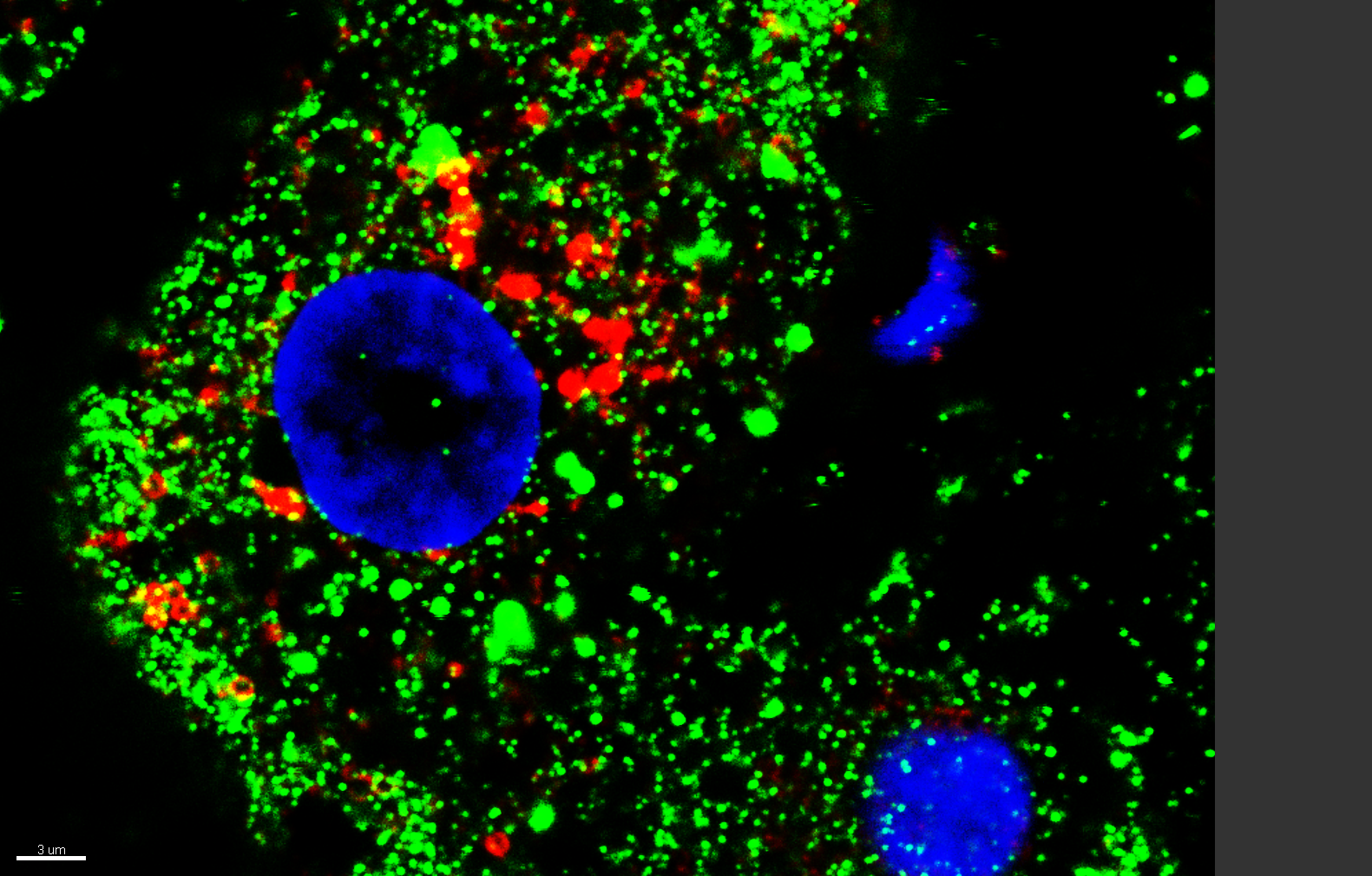

Supplement: Supplementary file 11 — Figure EV1-EV5 Source Data [file 44318_2026_755_MOESM11_ESM.zip › Extended version Figures EV1-EV5/EMBOJ-2025-121050 Figure EV5/EV5D/Microscopy PI4P_Rab11b.tif]

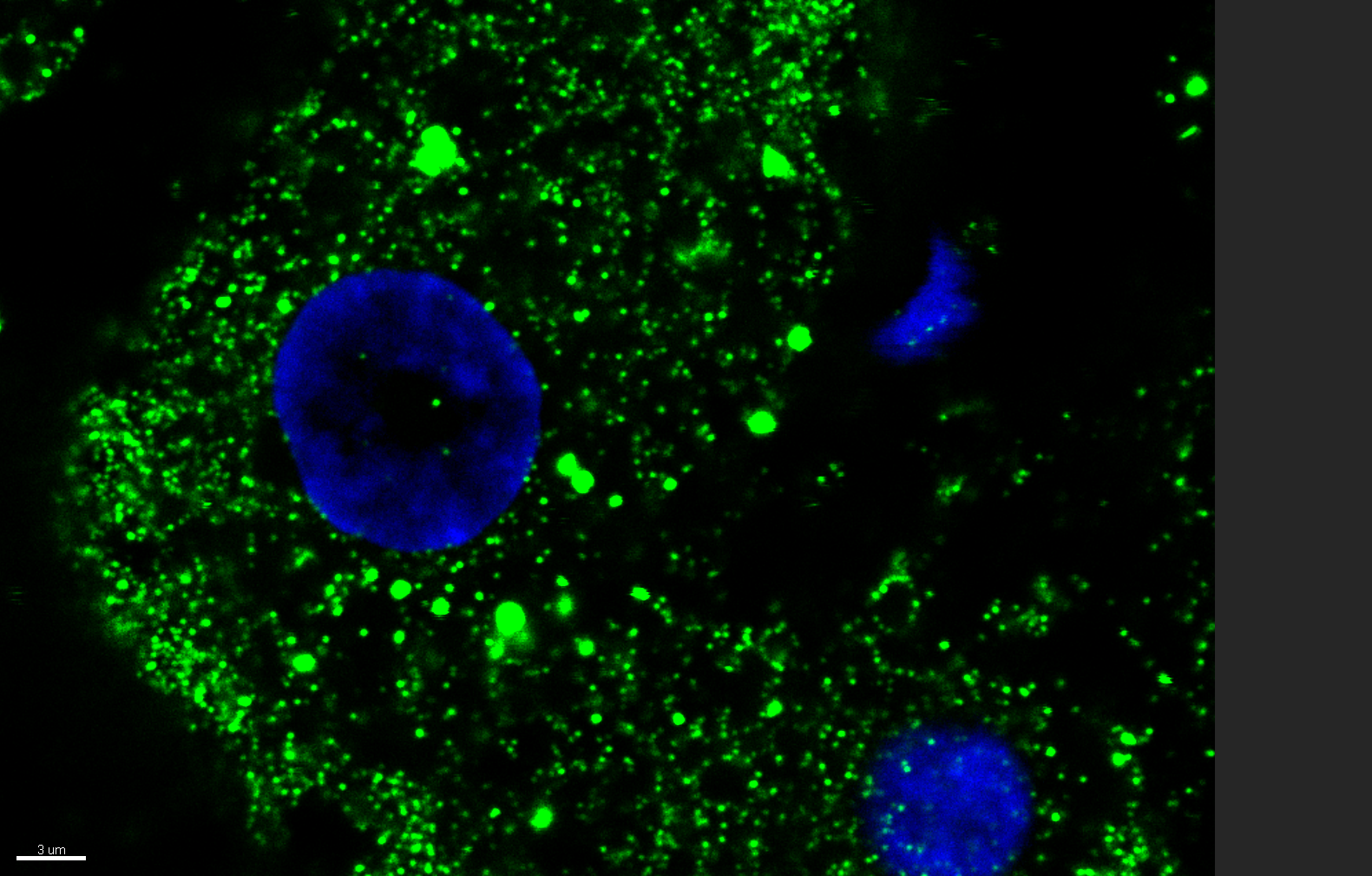

Supplement: Supplementary file 11 — Figure EV1-EV5 Source Data [file 44318_2026_755_MOESM11_ESM.zip › Extended version Figures EV1-EV5/EMBOJ-2025-121050 Figure EV5/EV5D/MicroscopyPI4P.tif]

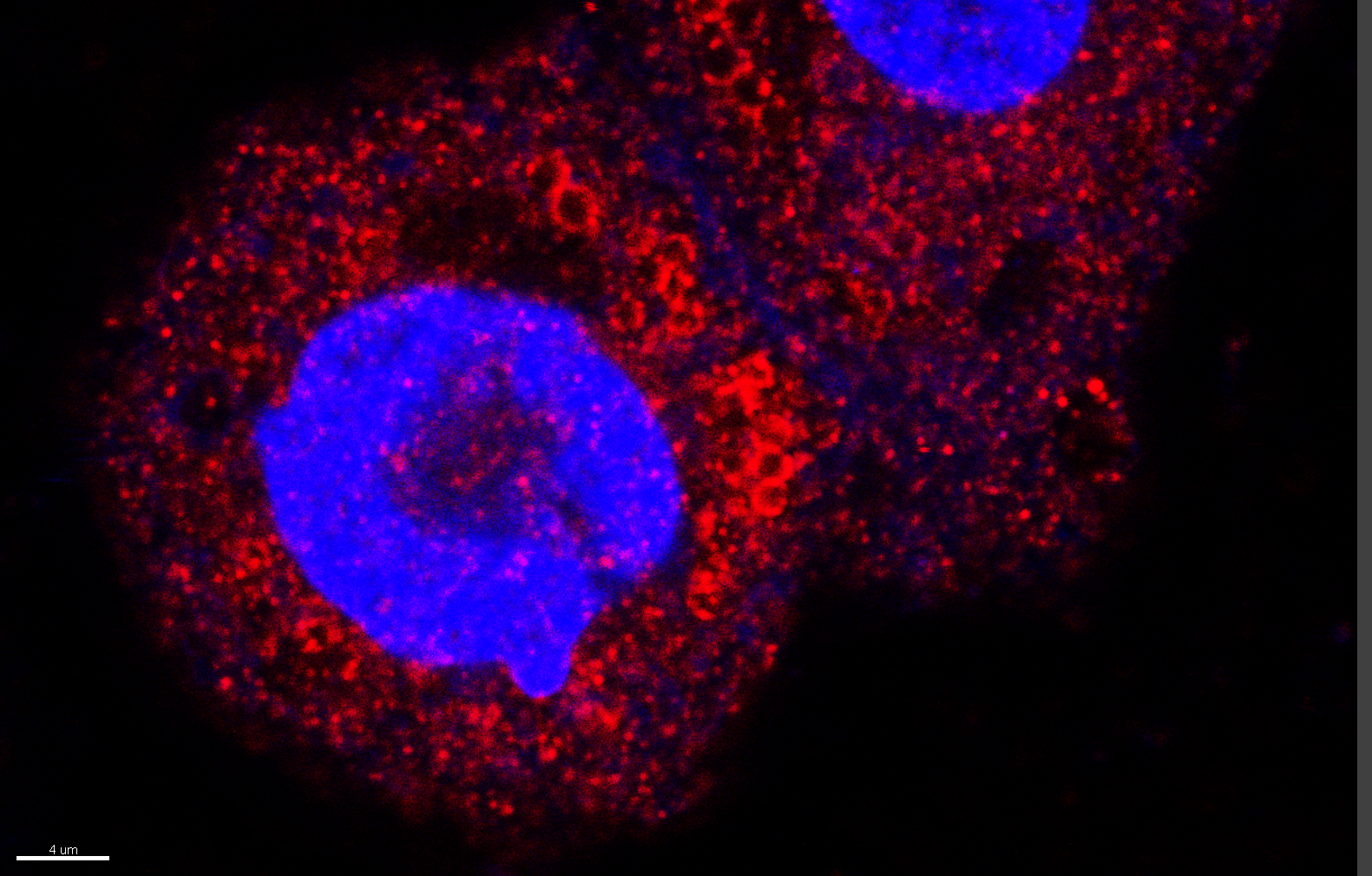

Supplement: Supplementary file 11 — Figure EV1-EV5 Source Data [file 44318_2026_755_MOESM11_ESM.zip › Extended version Figures EV1-EV5/EMBOJ-2025-121050 Figure EV5/EV5C/Microscopy Rab5.tif]

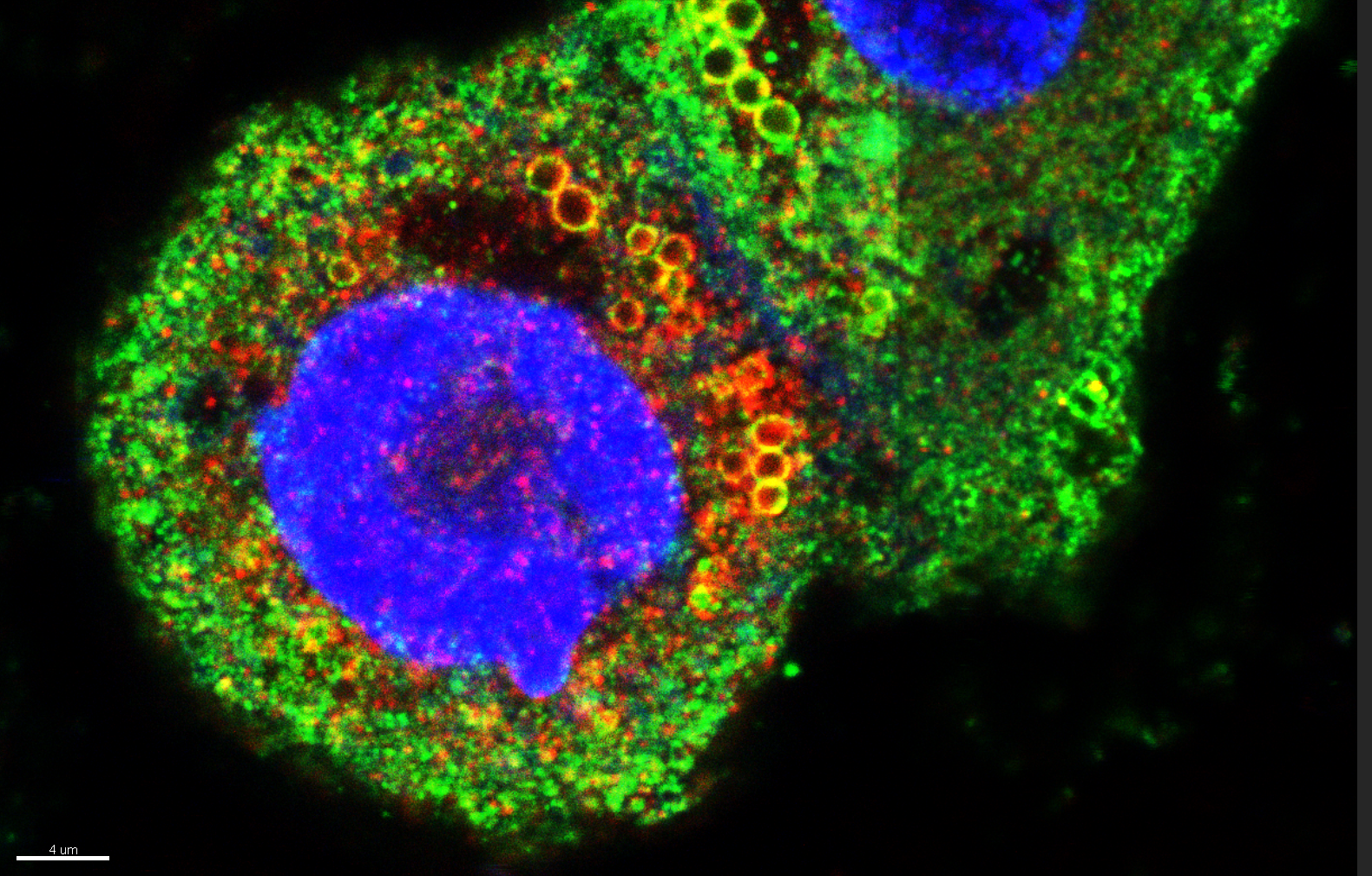

Supplement: Supplementary file 11 — Figure EV1-EV5 Source Data [file 44318_2026_755_MOESM11_ESM.zip › Extended version Figures EV1-EV5/EMBOJ-2025-121050 Figure EV5/EV5C/Microscopy Rab11 Flg-Rab5.tif]

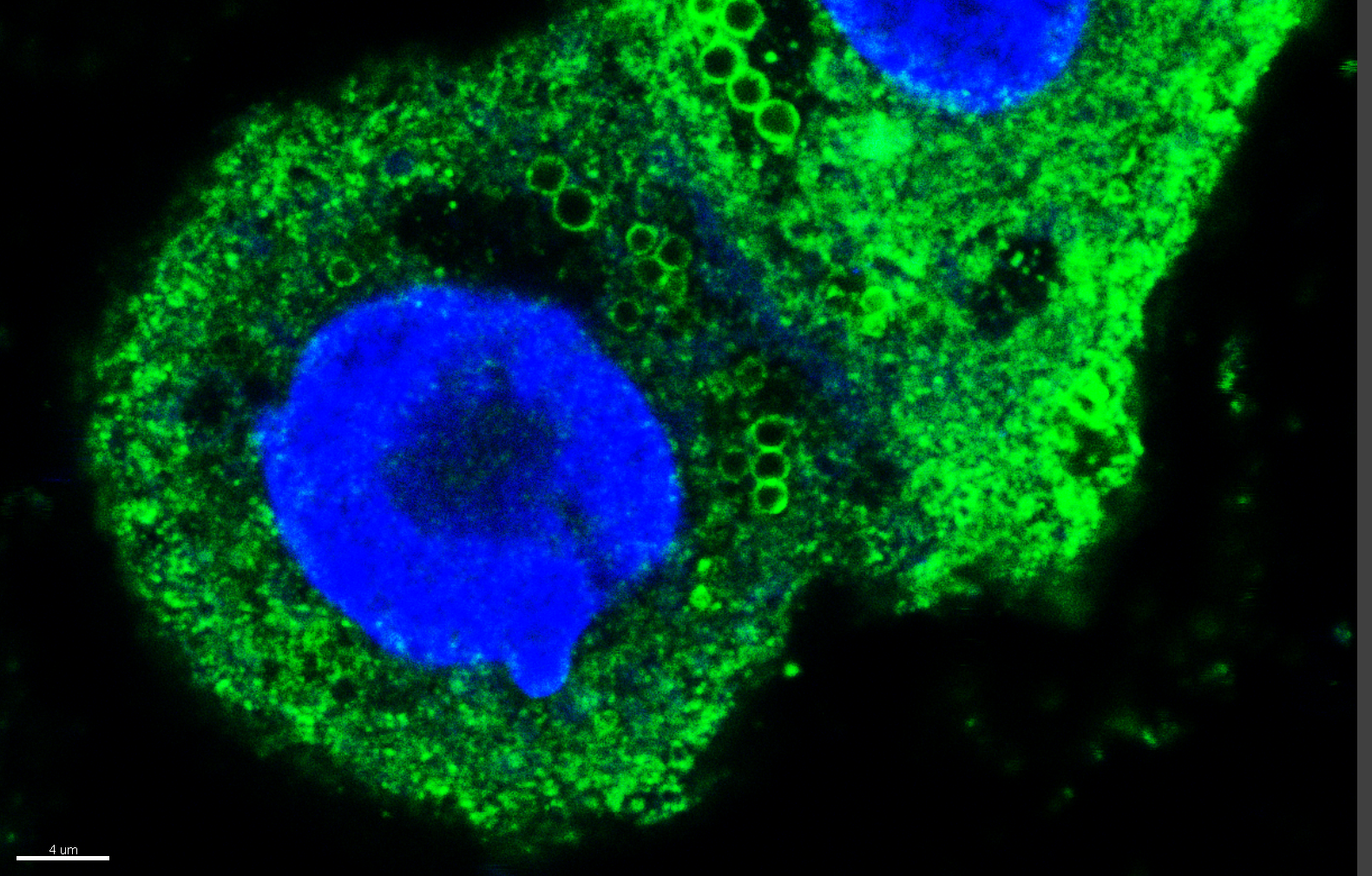

Supplement: Supplementary file 11 — Figure EV1-EV5 Source Data [file 44318_2026_755_MOESM11_ESM.zip › Extended version Figures EV1-EV5/EMBOJ-2025-121050 Figure EV5/EV5C/Microscopy Flag-Rab11.tif]

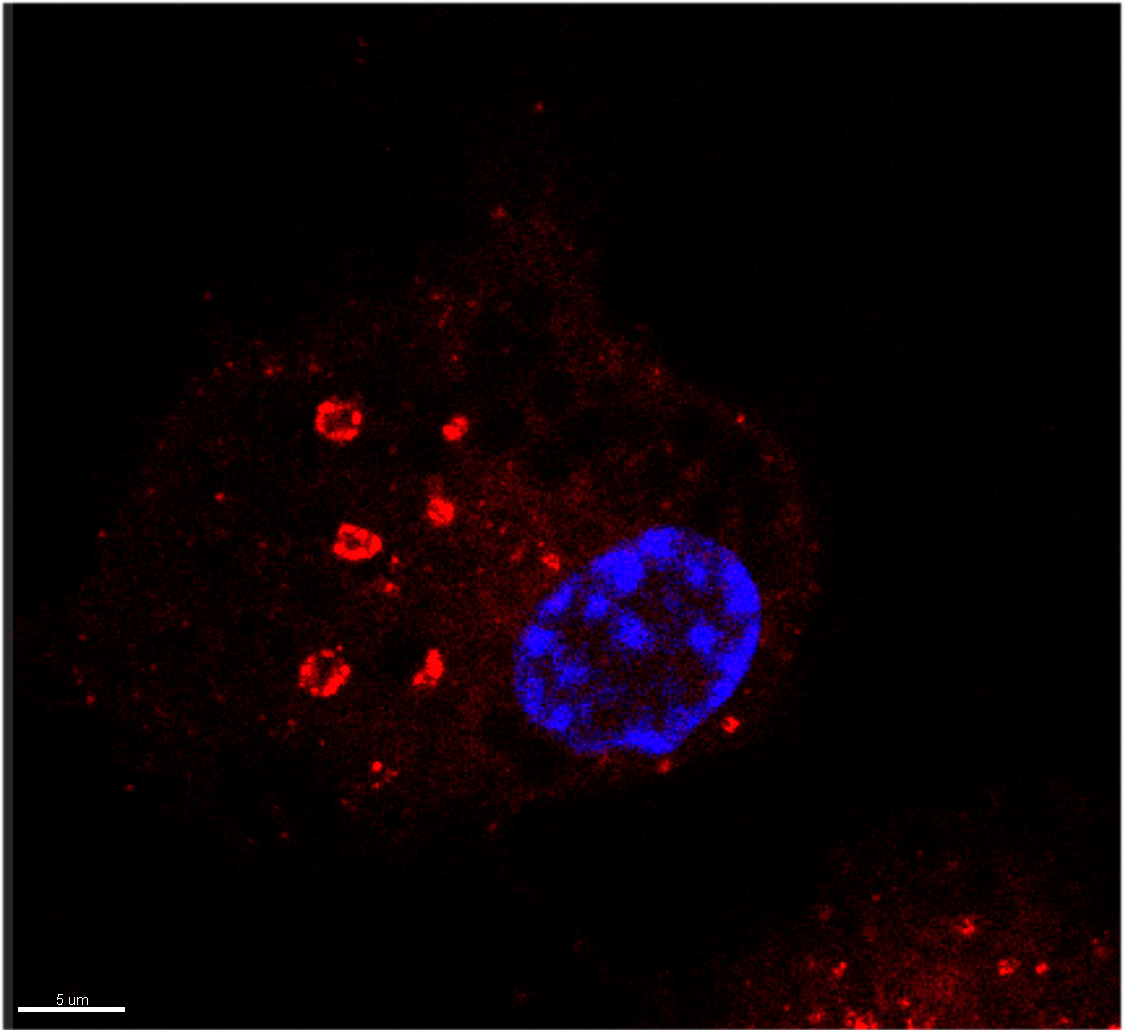

Supplement: Supplementary file 11 — Figure EV1-EV5 Source Data [file 44318_2026_755_MOESM11_ESM.zip › Extended version Figures EV1-EV5/EMBOJ-2025-121050 Figure EV5/EV5A/Microscopy EEA1 .tif]

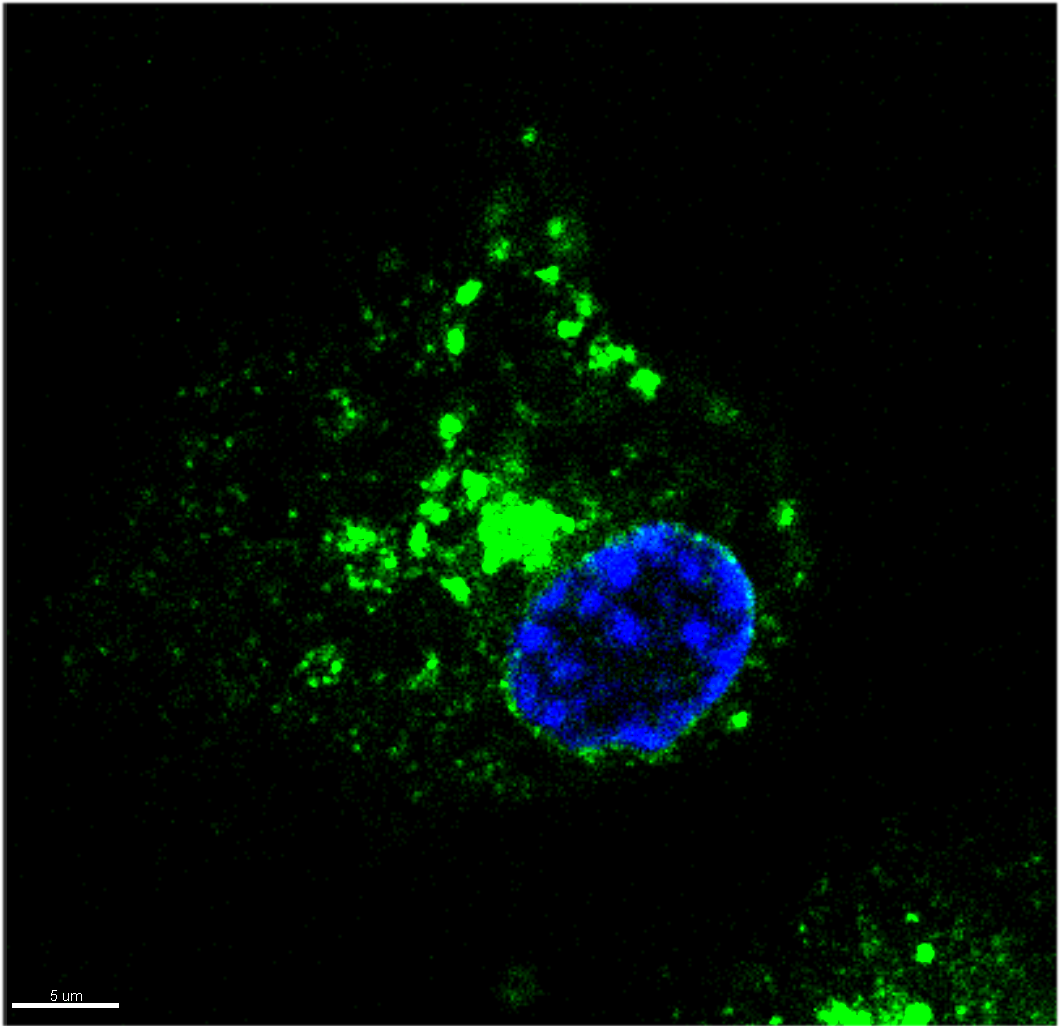

Supplement: Supplementary file 11 — Figure EV1-EV5 Source Data [file 44318_2026_755_MOESM11_ESM.zip › Extended version Figures EV1-EV5/EMBOJ-2025-121050 Figure EV5/EV5A/Microscopy.tif]

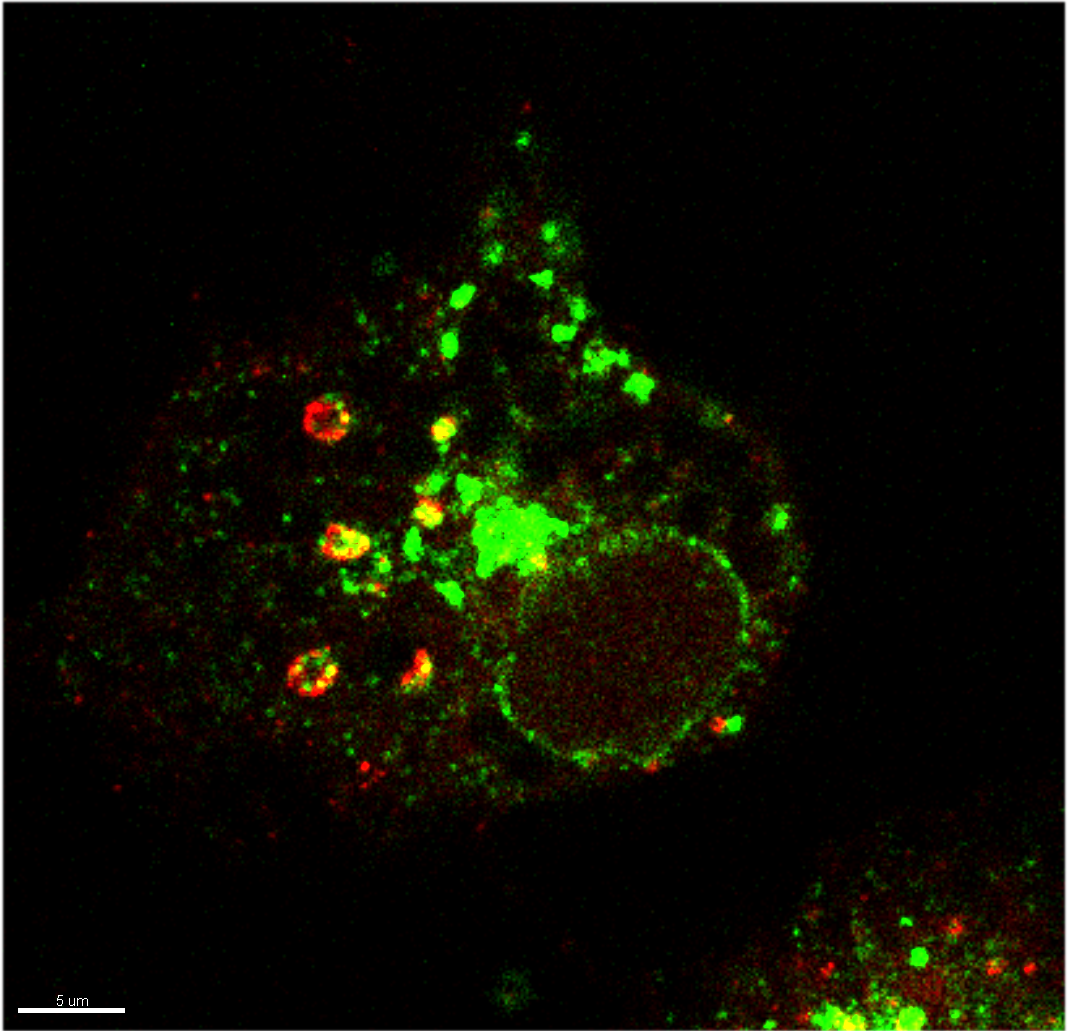

Supplement: Supplementary file 11 — Figure EV1-EV5 Source Data [file 44318_2026_755_MOESM11_ESM.zip › Extended version Figures EV1-EV5/EMBOJ-2025-121050 Figure EV5/EV5A/Microscopy TGN46_EEA1 .tif]

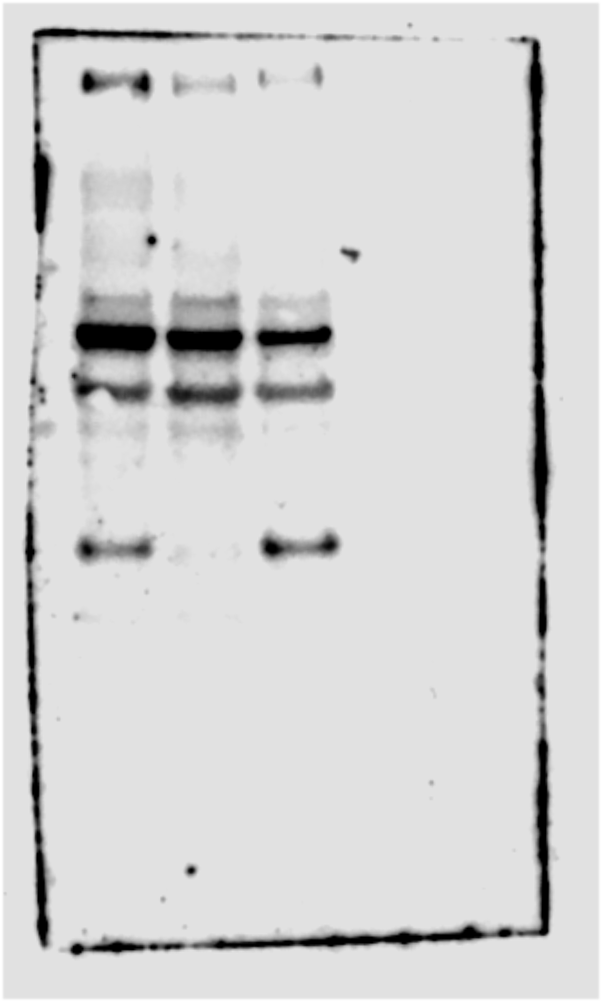

Supplement: Supplementary file 11 — Figure EV1-EV5 Source Data [file 44318_2026_755_MOESM11_ESM.zip › Extended version Figures EV1-EV5/EMBOJ-2025-121050 Figure EV2/Western TIF/western EV2C Rab11a.tif]

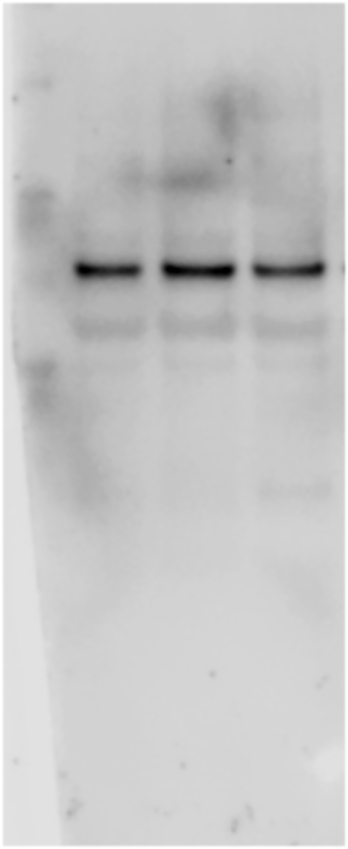

Supplement: Supplementary file 11 — Figure EV1-EV5 Source Data [file 44318_2026_755_MOESM11_ESM.zip › Extended version Figures EV1-EV5/EMBOJ-2025-121050 Figure EV2/Western TIF/western EV2D beta_tubulin.tif]

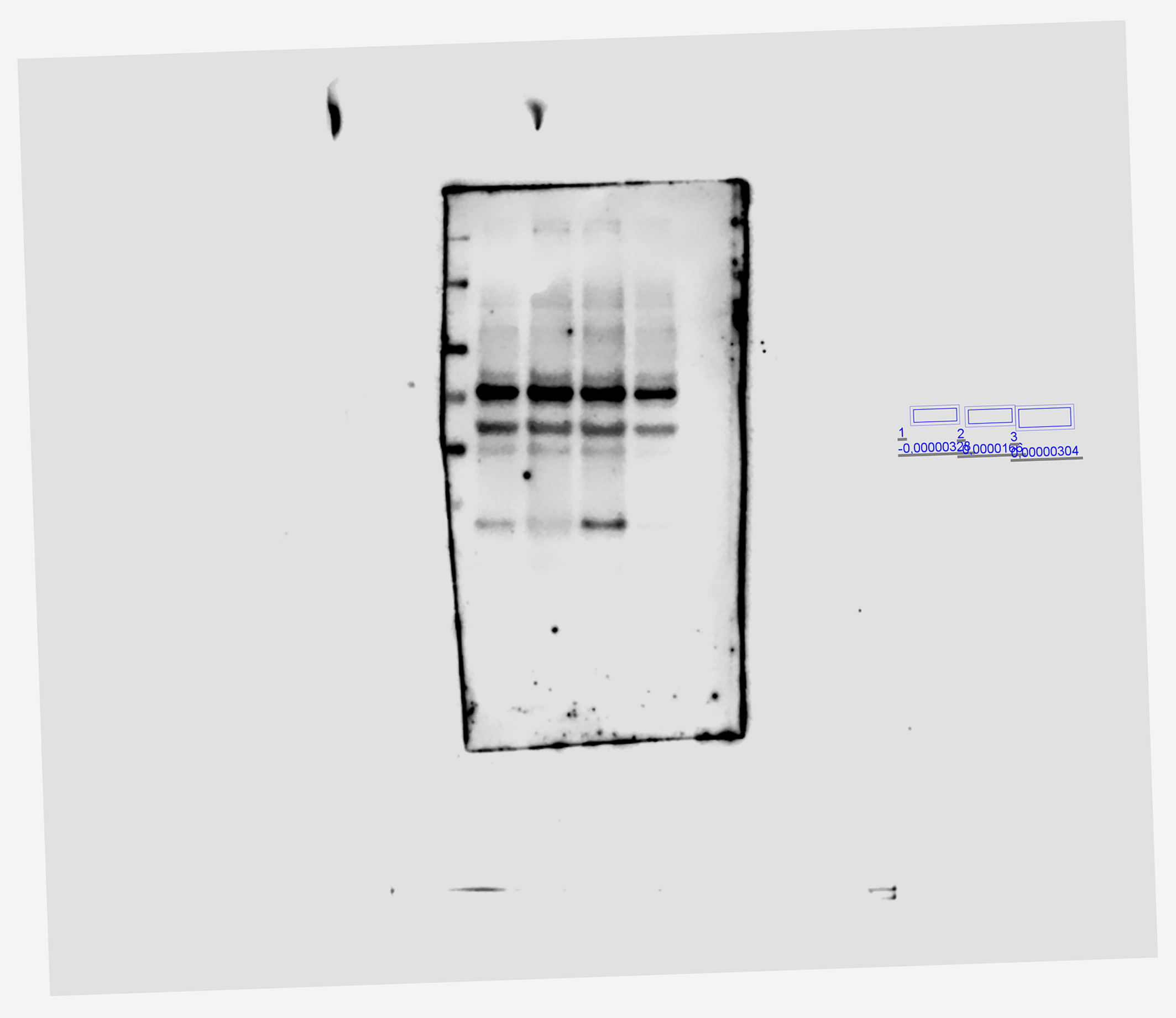

Supplement: Supplementary file 11 — Figure EV1-EV5 Source Data [file 44318_2026_755_MOESM11_ESM.zip › Extended version Figures EV1-EV5/EMBOJ-2025-121050 Figure EV2/Western TIF/western EV2D Rab11a.tif]

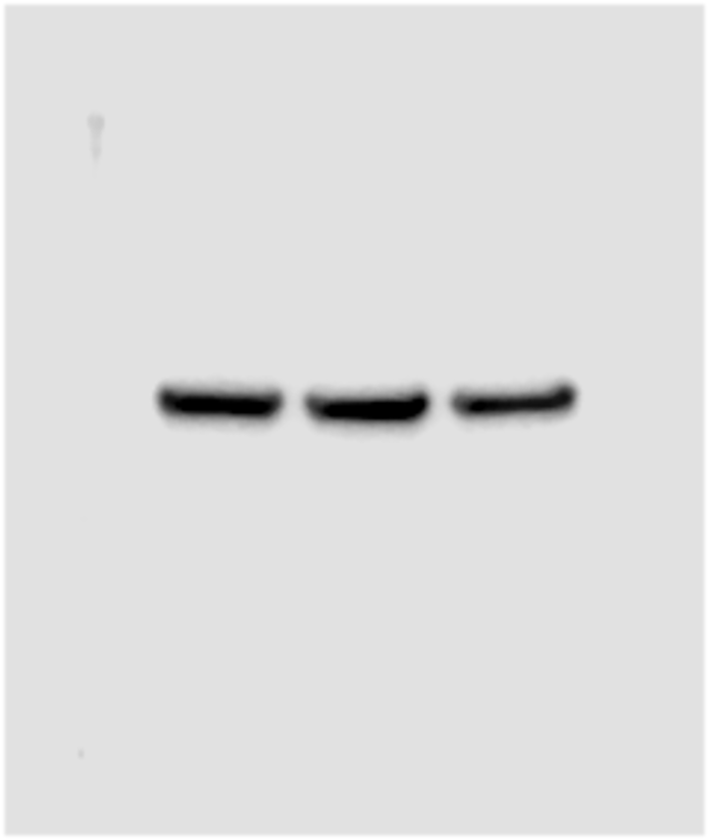

Supplement: Supplementary file 11 — Figure EV1-EV5 Source Data [file 44318_2026_755_MOESM11_ESM.zip › Extended version Figures EV1-EV5/EMBOJ-2025-121050 Figure EV2/Western TIF/western EV2C beta_Tubulin.tif]

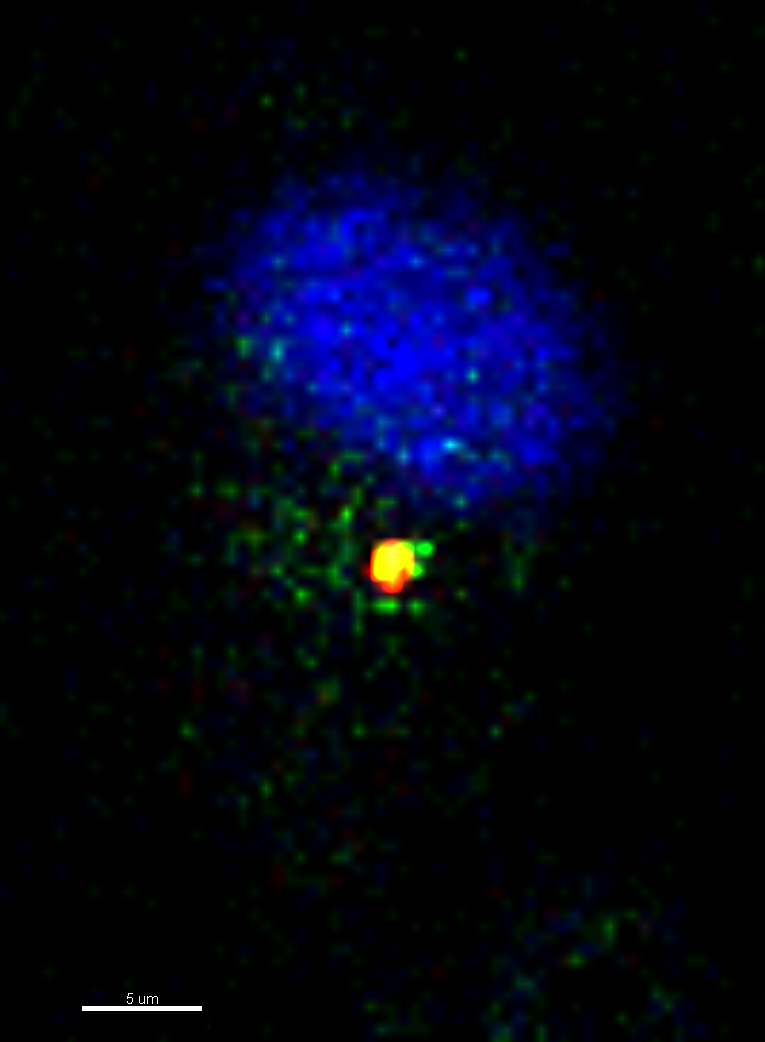

Supplement: Supplementary file 11 — Figure EV1-EV5 Source Data [file 44318_2026_755_MOESM11_ESM.zip › Extended version Figures EV1-EV5/EMBOJ-2025-121050 Figure EV3/EV3A/microscopy EV3A FIP2_ASC.tif]

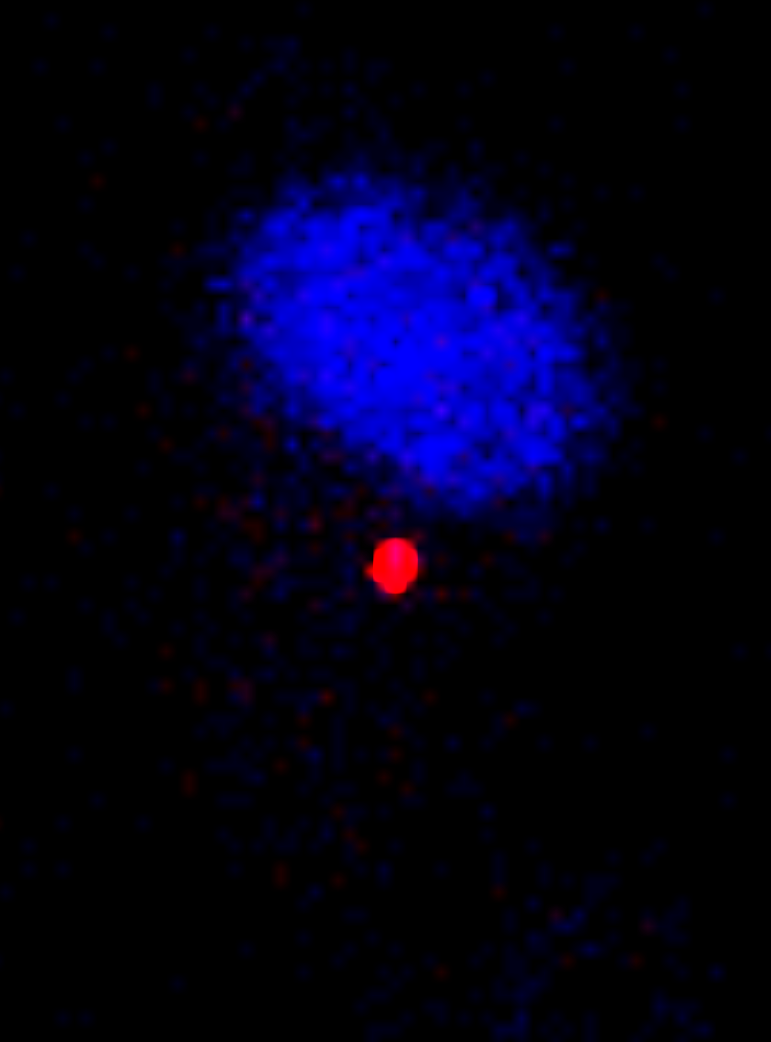

Supplement: Supplementary file 11 — Figure EV1-EV5 Source Data [file 44318_2026_755_MOESM11_ESM.zip › Extended version Figures EV1-EV5/EMBOJ-2025-121050 Figure EV3/EV3A/microscopy EV3A ASC.tif]

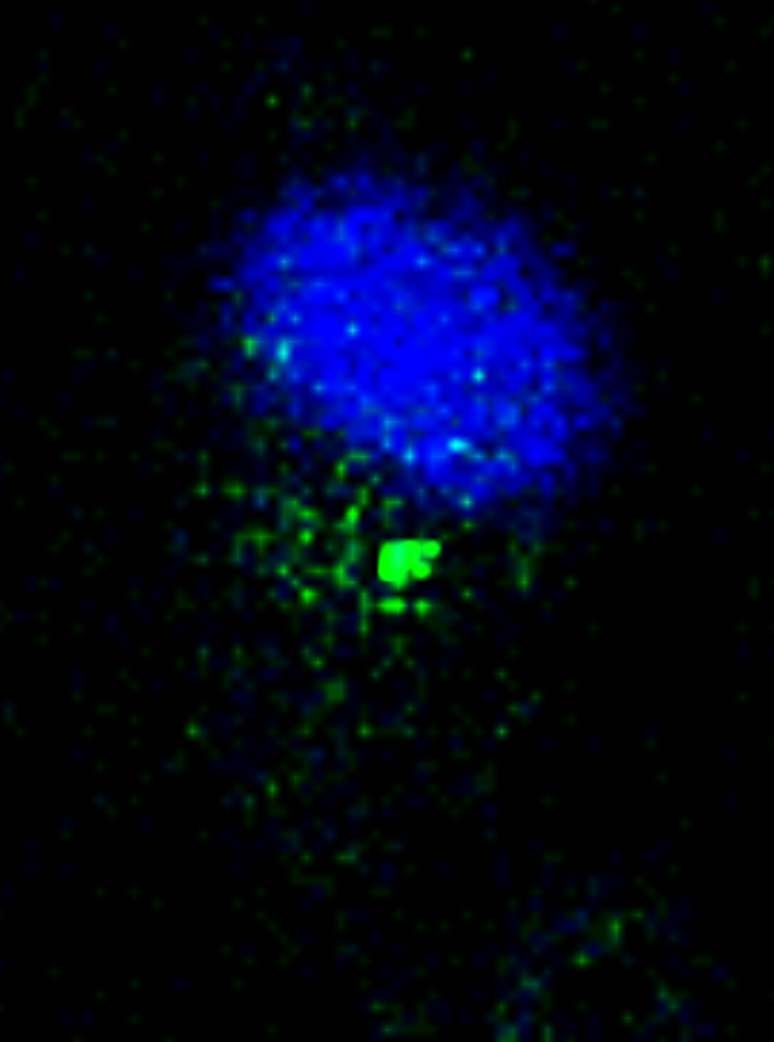

Supplement: Supplementary file 11 — Figure EV1-EV5 Source Data [file 44318_2026_755_MOESM11_ESM.zip › Extended version Figures EV1-EV5/EMBOJ-2025-121050 Figure EV3/EV3A/microscopy EV3A FIP2.tif]

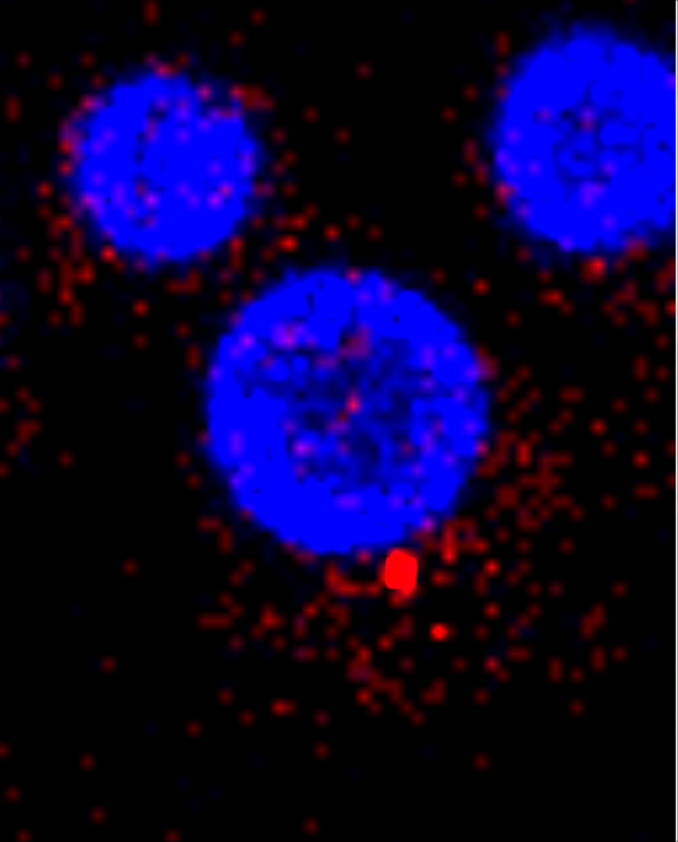

Supplement: Supplementary file 11 — Figure EV1-EV5 Source Data [file 44318_2026_755_MOESM11_ESM.zip › Extended version Figures EV1-EV5/EMBOJ-2025-121050 Figure EV3/EV3B/microscopy EV3B ASC.tif]

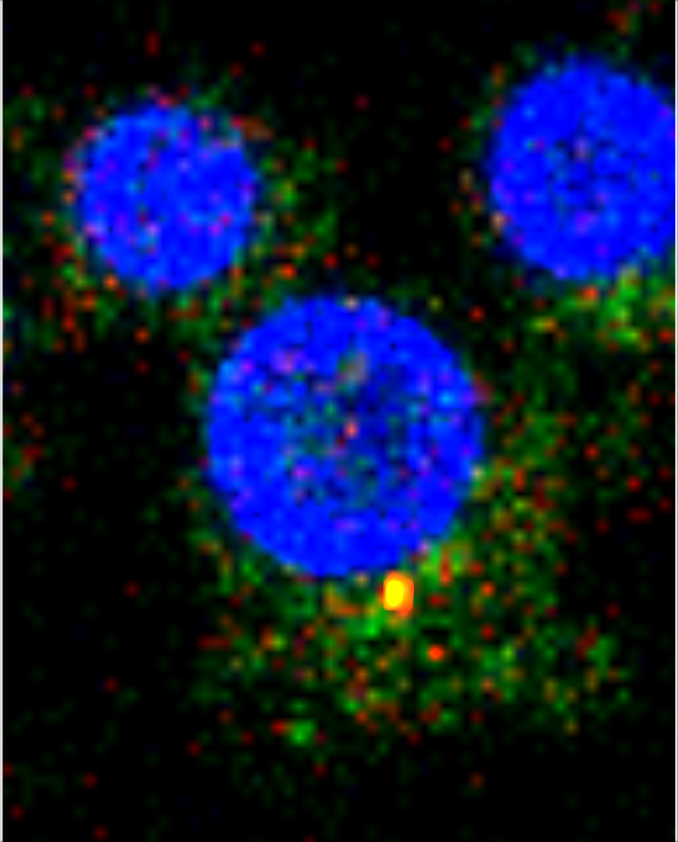

Supplement: Supplementary file 11 — Figure EV1-EV5 Source Data [file 44318_2026_755_MOESM11_ESM.zip › Extended version Figures EV1-EV5/EMBOJ-2025-121050 Figure EV3/EV3B/microscopy EV3B NLRP3_ASC.tif]

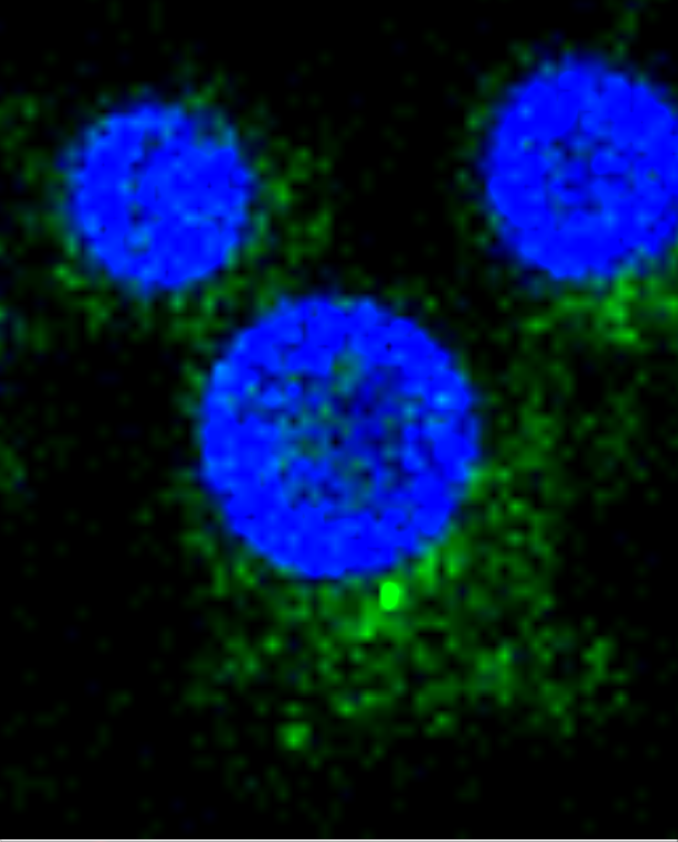

Supplement: Supplementary file 11 — Figure EV1-EV5 Source Data [file 44318_2026_755_MOESM11_ESM.zip › Extended version Figures EV1-EV5/EMBOJ-2025-121050 Figure EV3/EV3B/microscopy EV3B NLRP3.tif]

EV4A

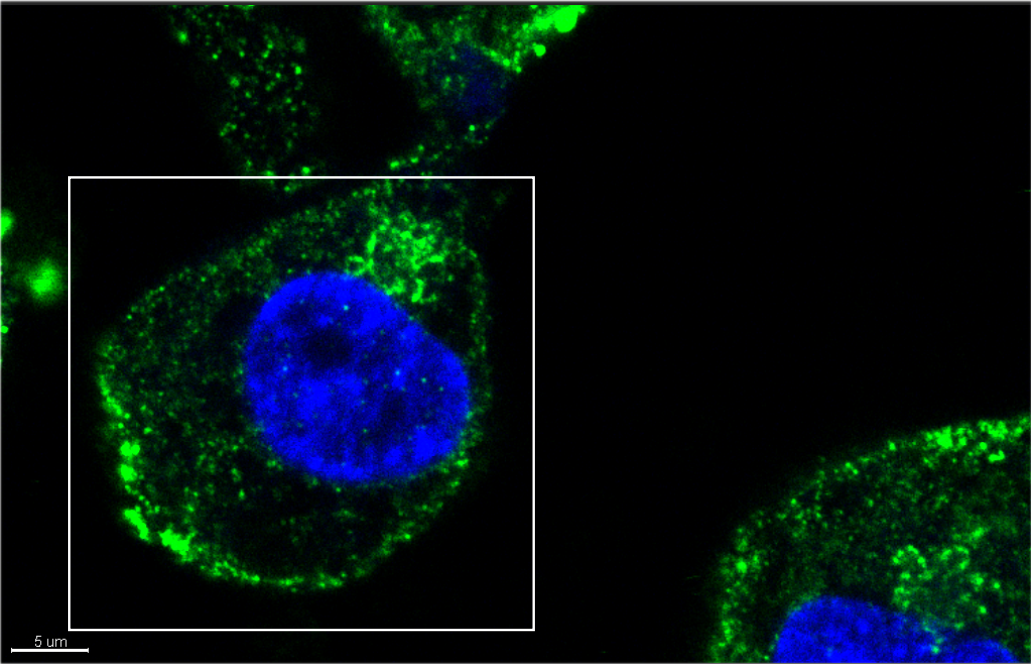

PI4P Crop

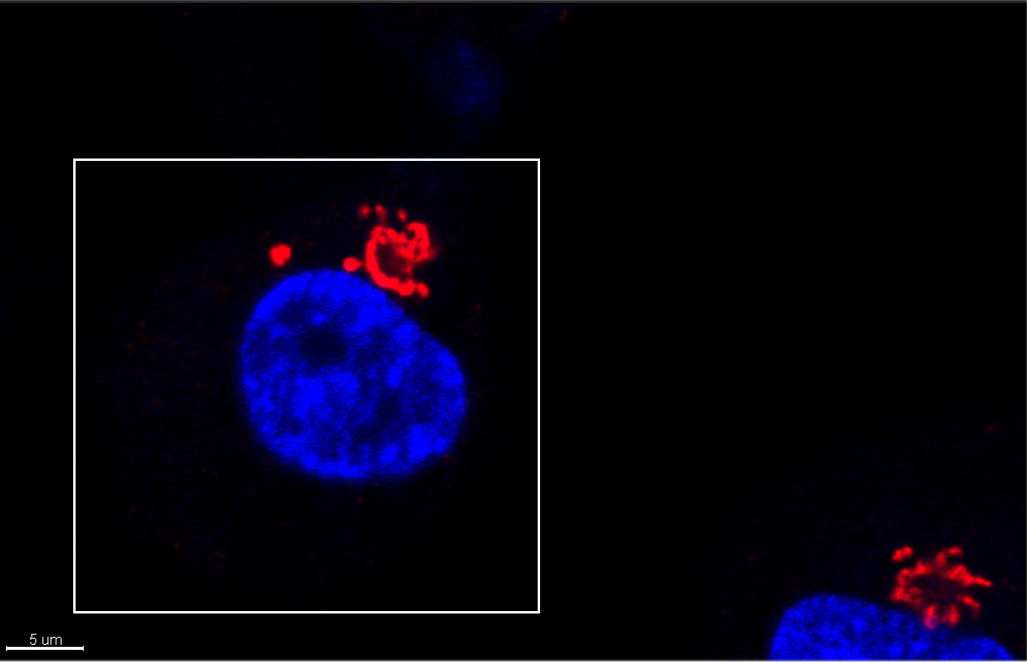

TGN46 Crop

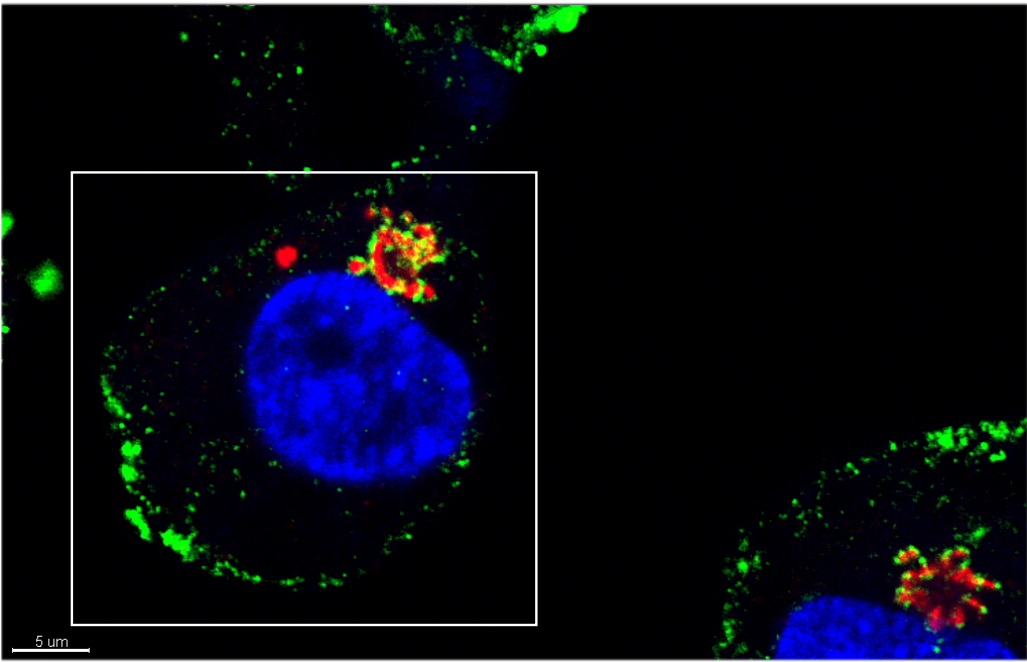

PI4P\_TGN46 Crop

Supplement: Supplementary file 11 — Figure EV1-EV5 Source Data [file 44318_2026_755_MOESM11_ESM.zip › Extended version Figures EV1-EV5/EMBOJ-2025-121050 Figure EV4/EV4A/~ai-efd3df2f-56d4-45c4-a48d-04e2432fc428_.tmp]

EV4B

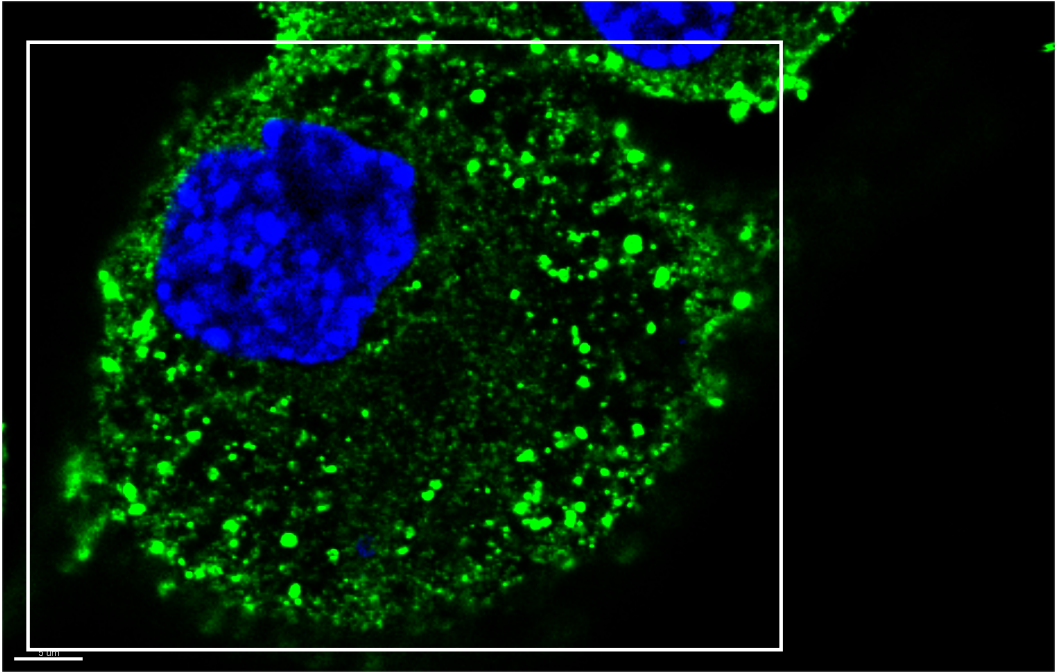

PI4P Crop

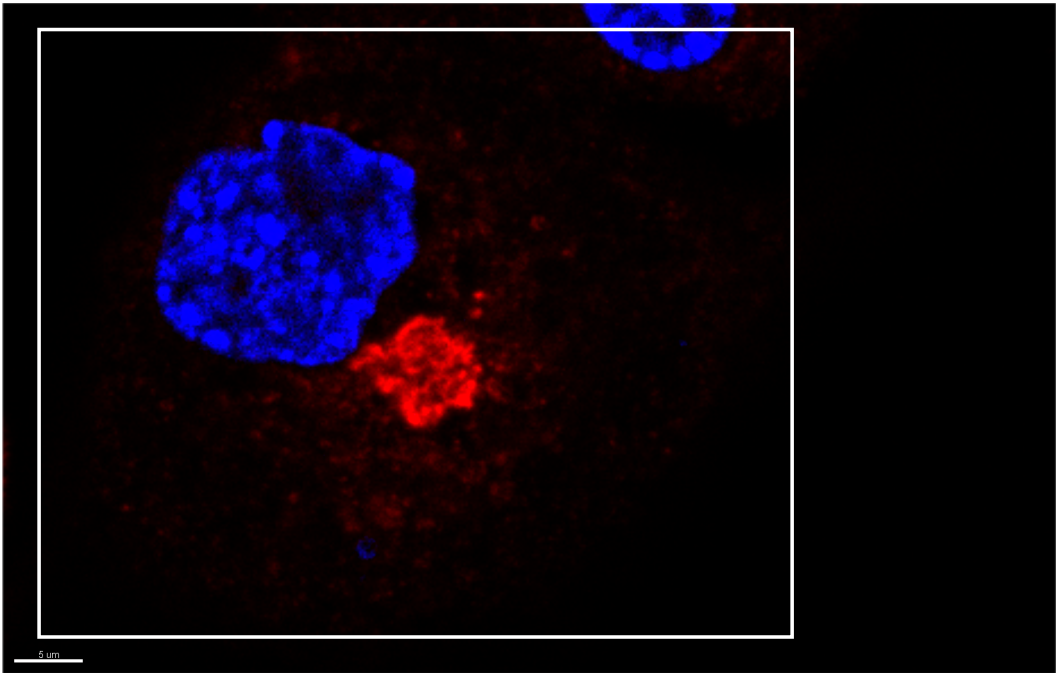

TGN46 Crop

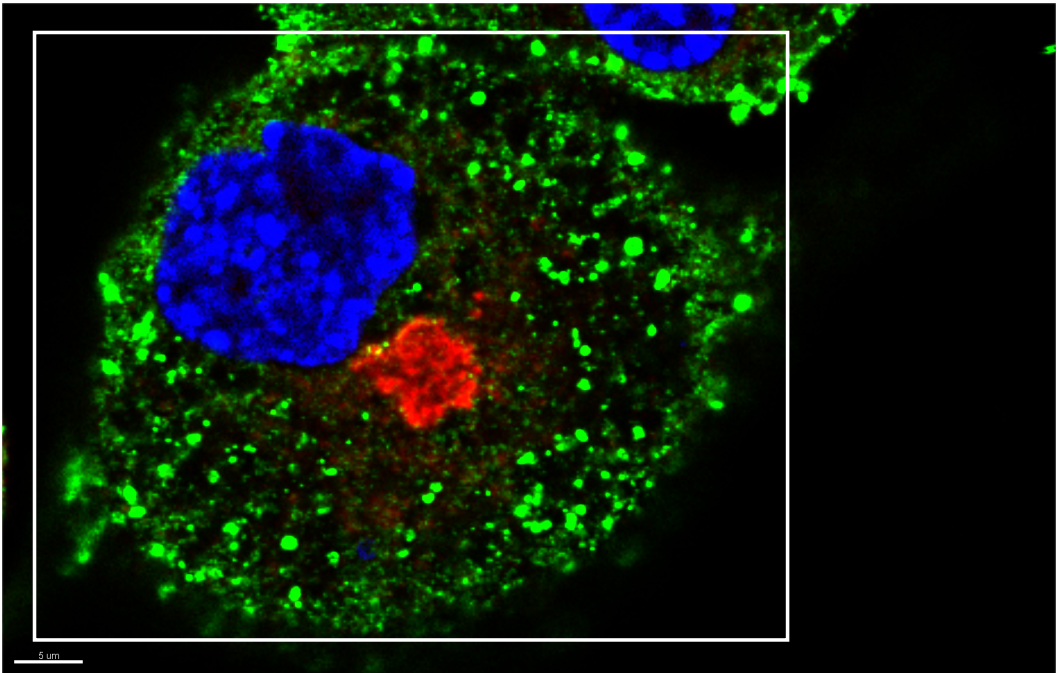

PI4P\_TGN46 Crop

Supplement: Supplementary file 11 — Figure EV1-EV5 Source Data [file 44318_2026_755_MOESM11_ESM.zip › Extended version Figures EV1-EV5/EMBOJ-2025-121050 Figure EV4/EV4B/~ai-ebc66fca-1ebb-4200-8d3c-90310e42600b_.tmp]

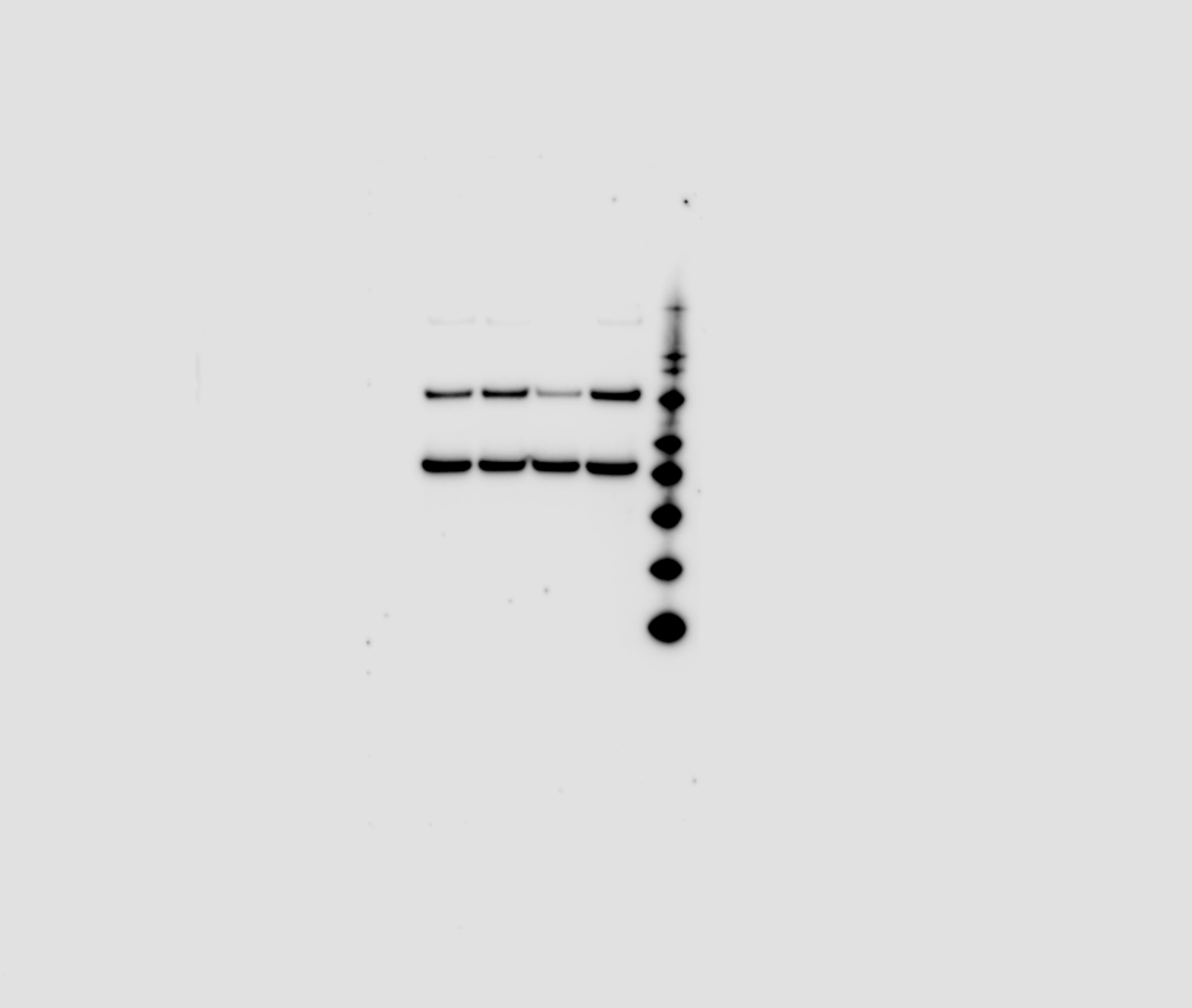

Supplement: Supplementary file 11 — Figure EV1-EV5 Source Data [file 44318_2026_755_MOESM11_ESM.zip › Extended version Figures EV1-EV5/EMBOJ-2025-121050 Figure EV1/Fig. EV1 western TIF/EV1D/EV1D_beta_tubulin (FIP1).tif]

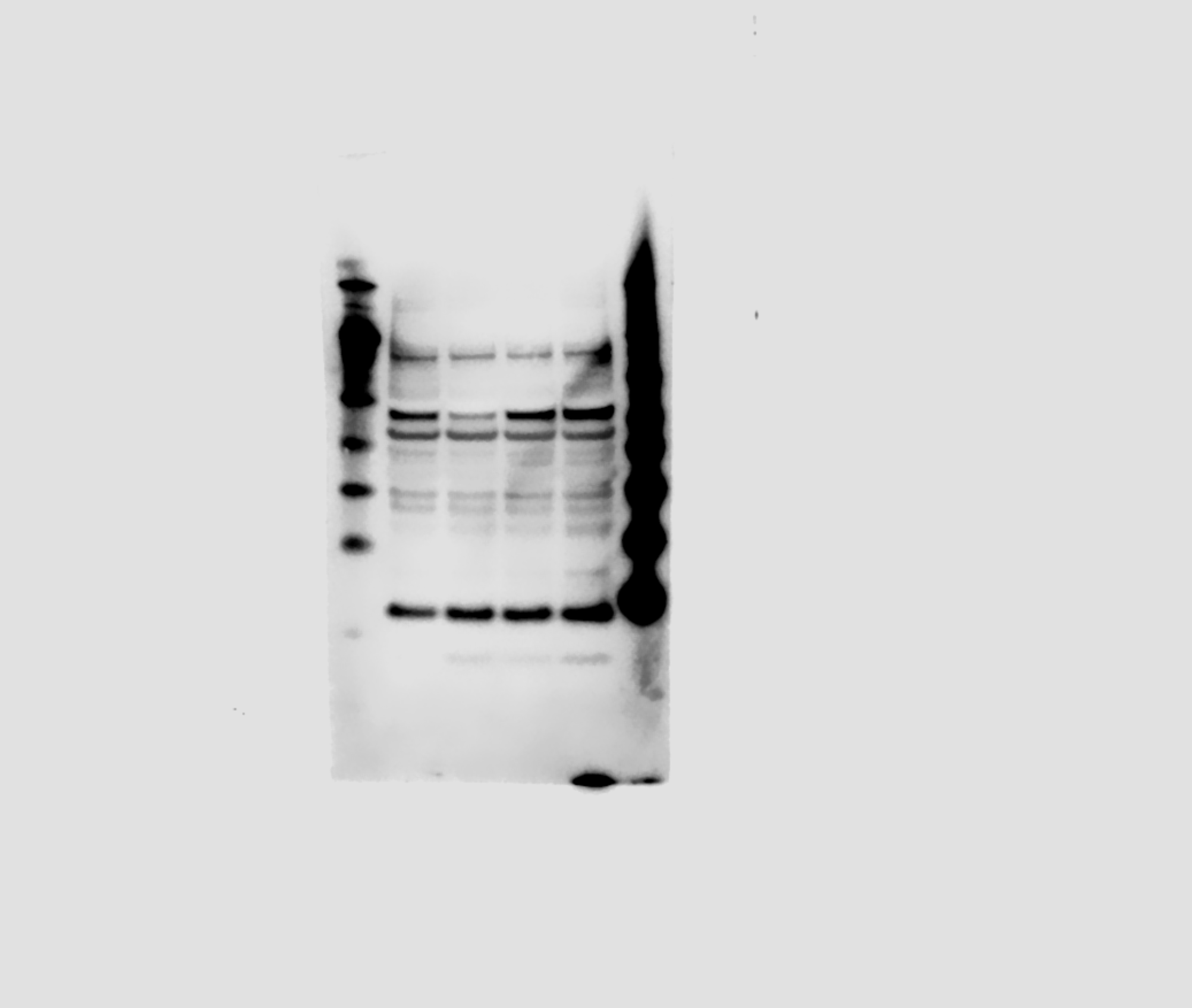

Supplement: Supplementary file 11 — Figure EV1-EV5 Source Data [file 44318_2026_755_MOESM11_ESM.zip › Extended version Figures EV1-EV5/EMBOJ-2025-121050 Figure EV1/Fig. EV1 western TIF/EV1D/EV1D FIP2.tif]

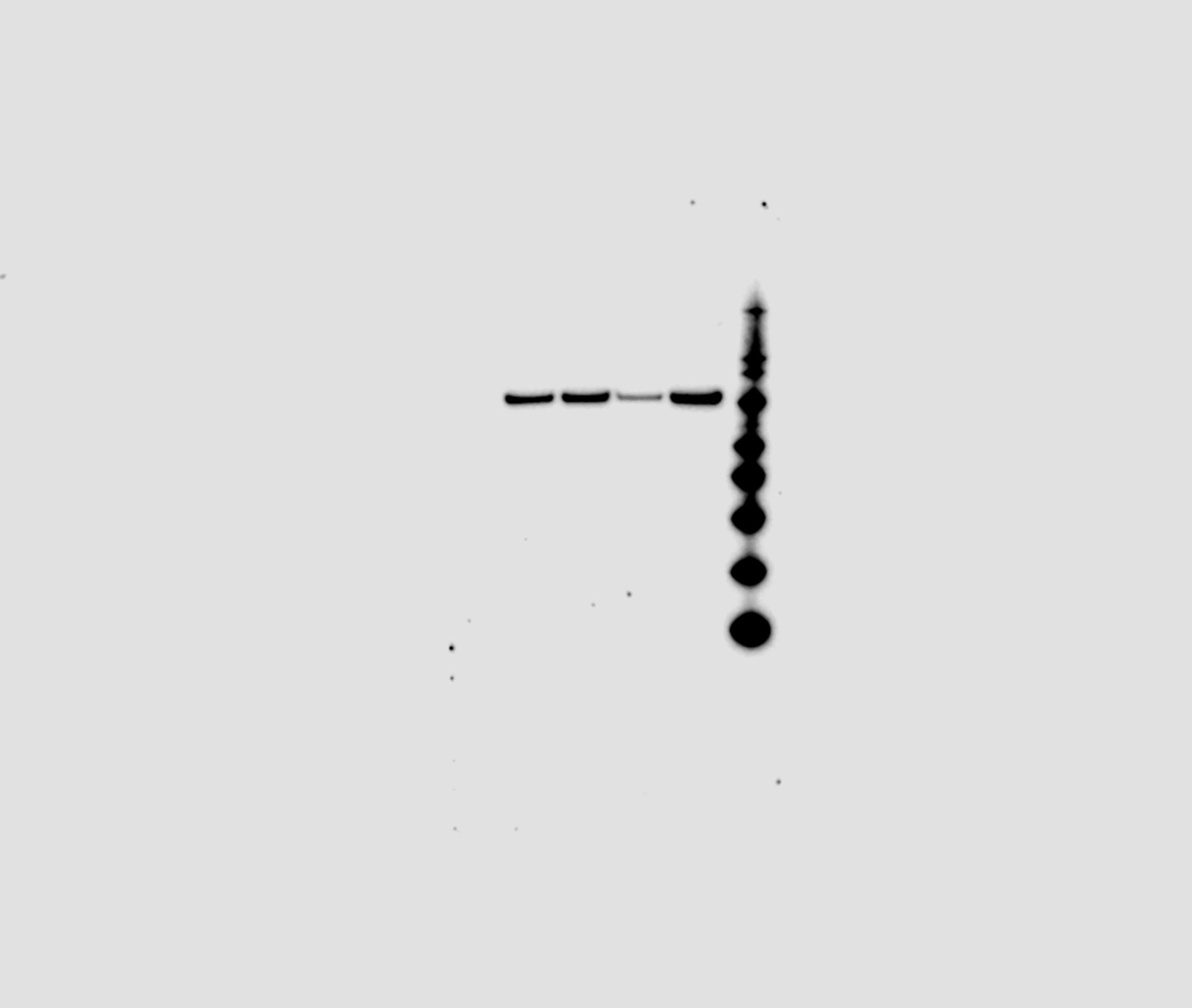

Supplement: Supplementary file 11 — Figure EV1-EV5 Source Data [file 44318_2026_755_MOESM11_ESM.zip › Extended version Figures EV1-EV5/EMBOJ-2025-121050 Figure EV1/Fig. EV1 western TIF/EV1D/EV1D FIP1.tif]

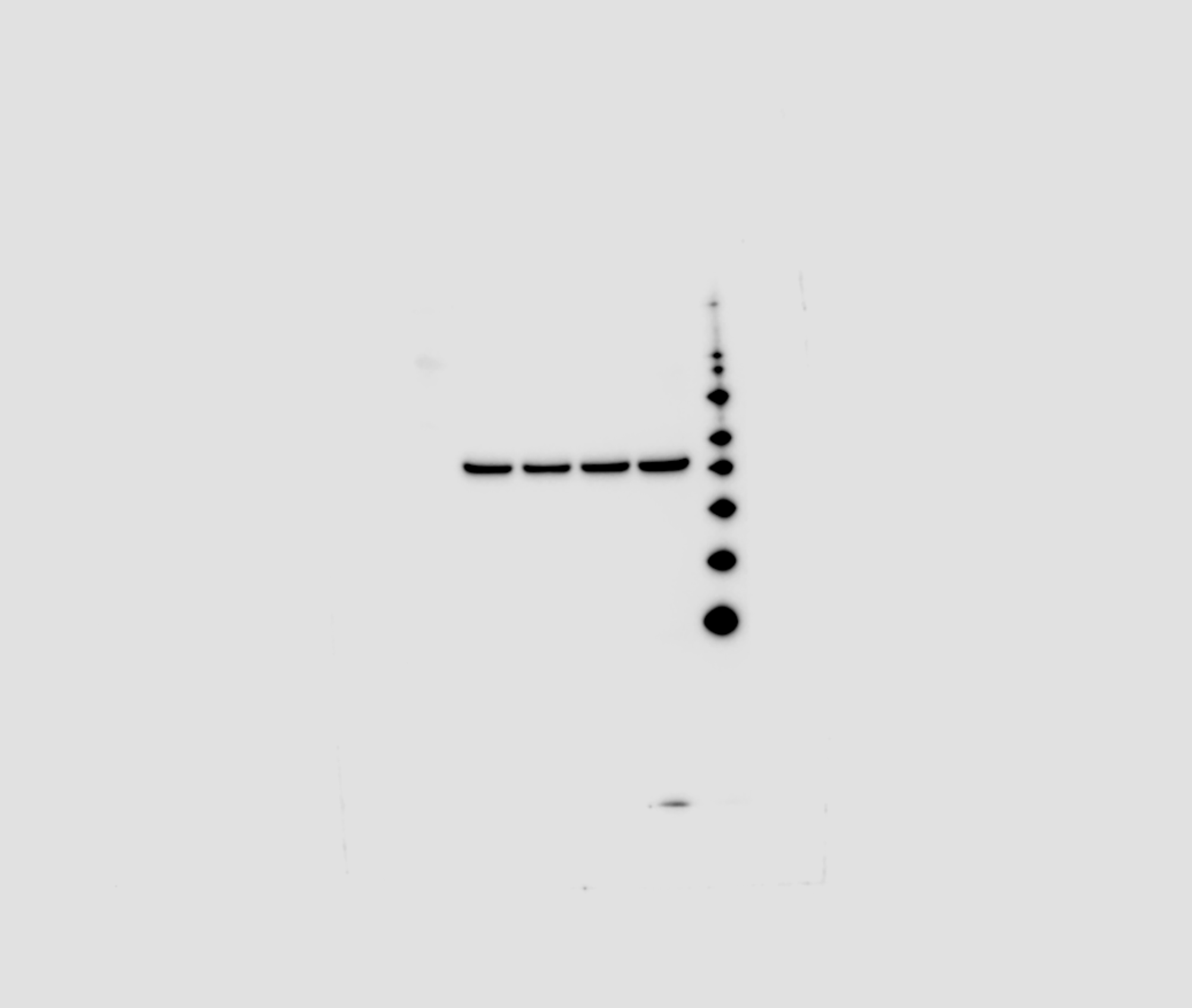

Supplement: Supplementary file 11 — Figure EV1-EV5 Source Data [file 44318_2026_755_MOESM11_ESM.zip › Extended version Figures EV1-EV5/EMBOJ-2025-121050 Figure EV1/Fig. EV1 western TIF/EV1D/EV1D_beta_tubulin (FIP2).tif]

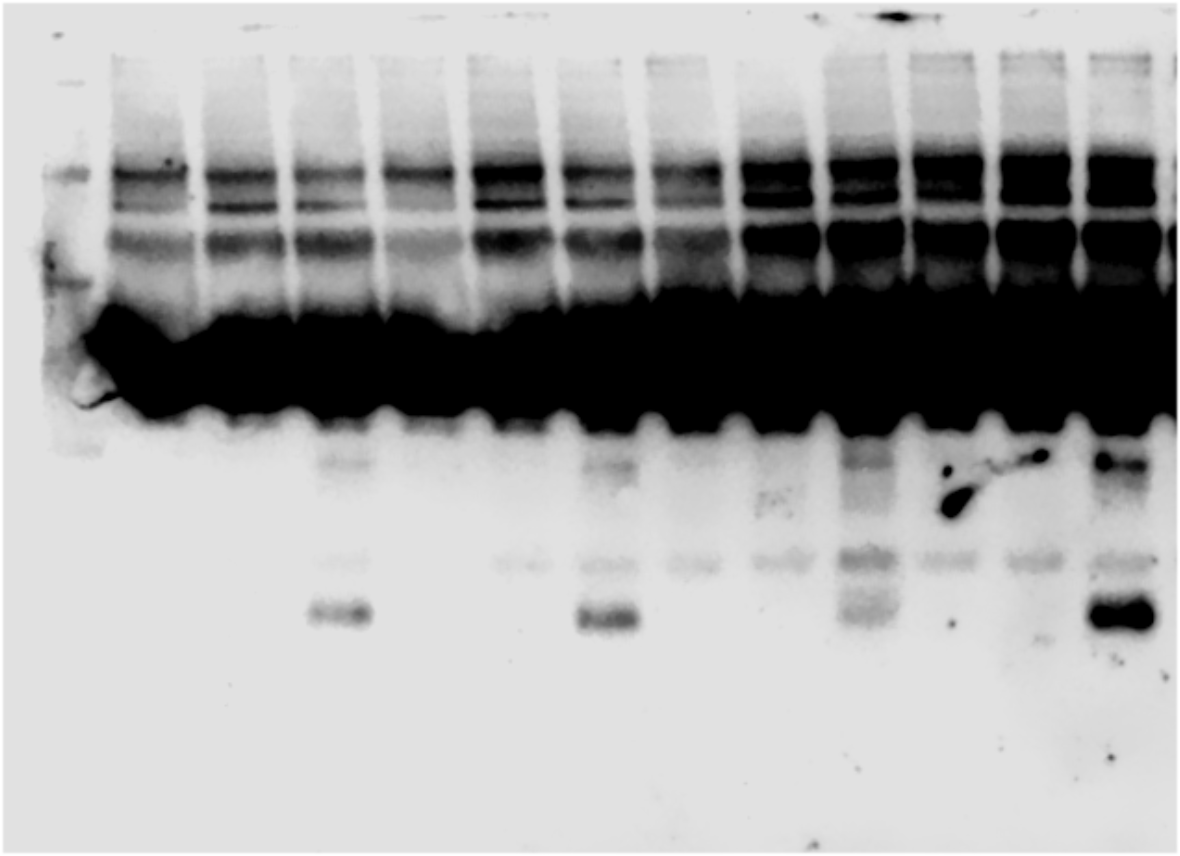

Supplement: Supplementary file 11 — Figure EV1-EV5 Source Data [file 44318_2026_755_MOESM11_ESM.zip › Extended version Figures EV1-EV5/EMBOJ-2025-121050 Figure EV1/Fig. EV1 western TIF/EV1F/EV1F sup_casp1.tif]

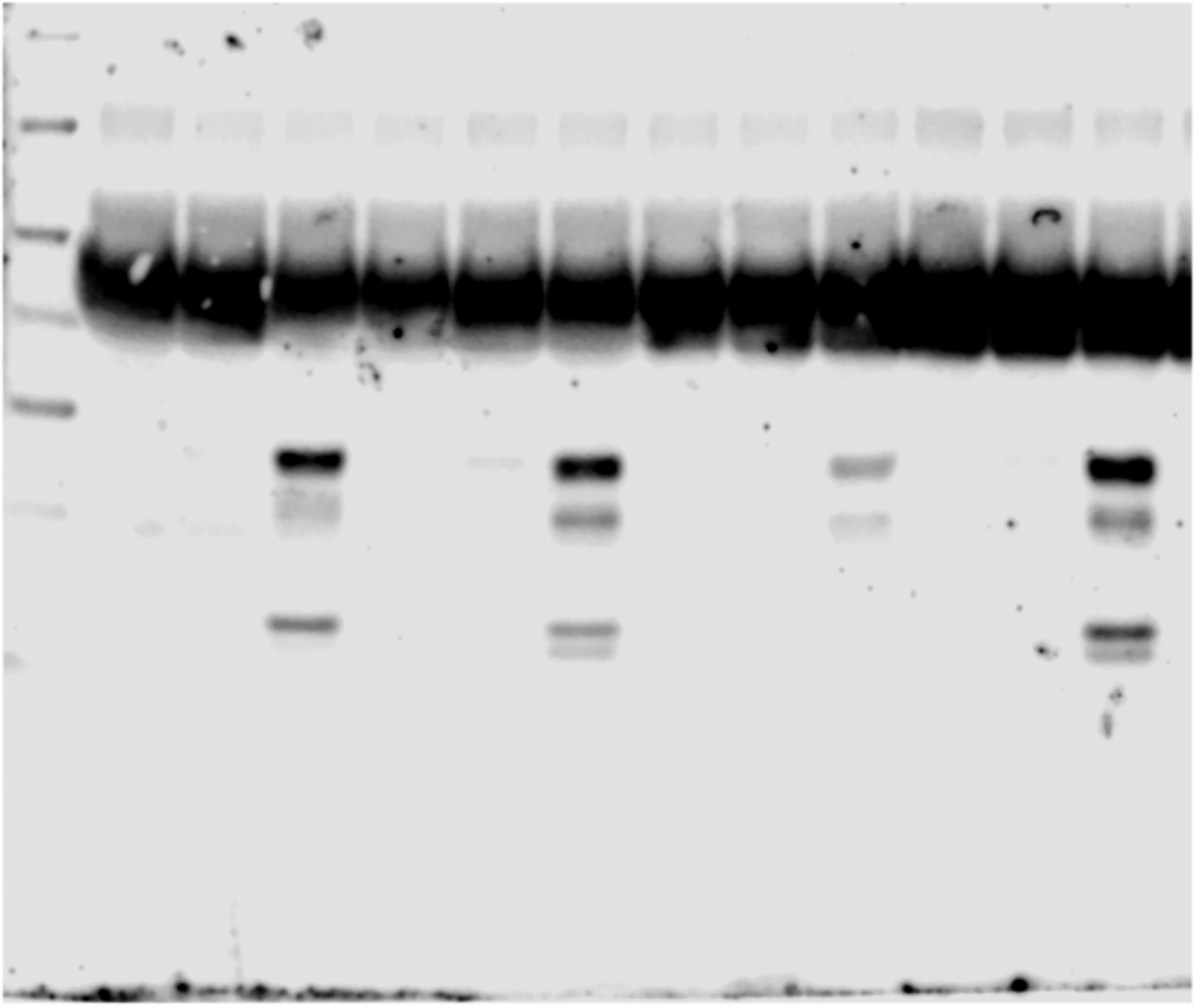

Supplement: Supplementary file 11 — Figure EV1-EV5 Source Data [file 44318_2026_755_MOESM11_ESM.zip › Extended version Figures EV1-EV5/EMBOJ-2025-121050 Figure EV1/Fig. EV1 western TIF/EV1F/EV1F sup_IL1b.tif]

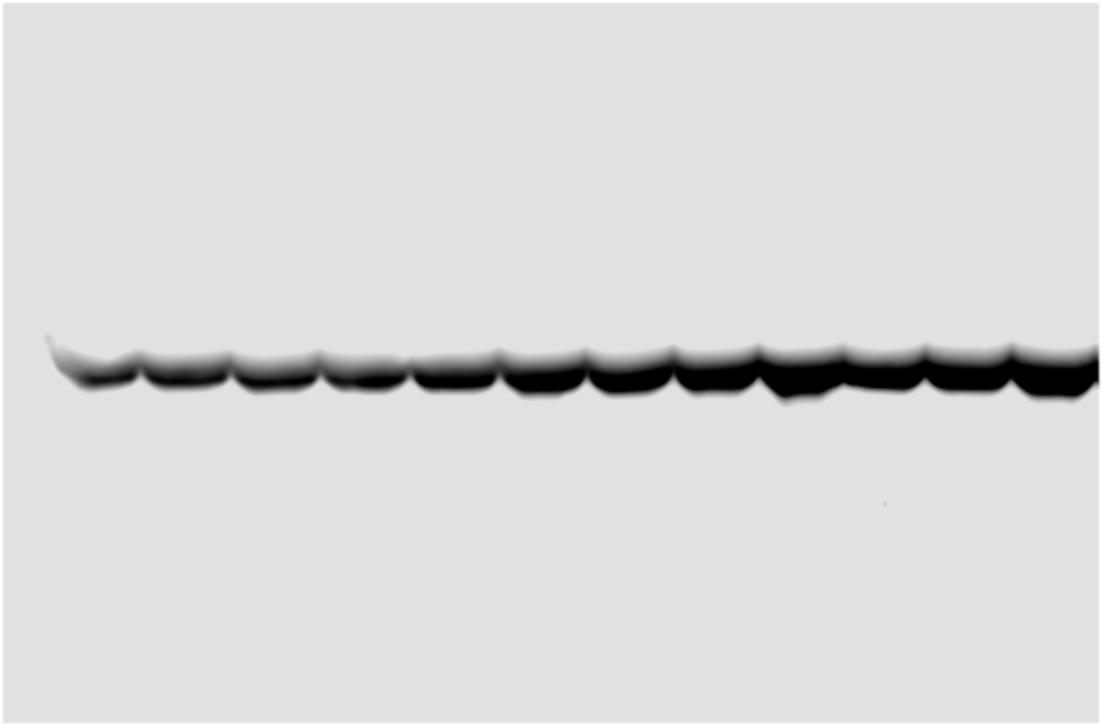

Supplement: Supplementary file 11 — Figure EV1-EV5 Source Data [file 44318_2026_755_MOESM11_ESM.zip › Extended version Figures EV1-EV5/EMBOJ-2025-121050 Figure EV1/Fig. EV1 western TIF/EV1F/EV1F sup_pro-casp1.tif]

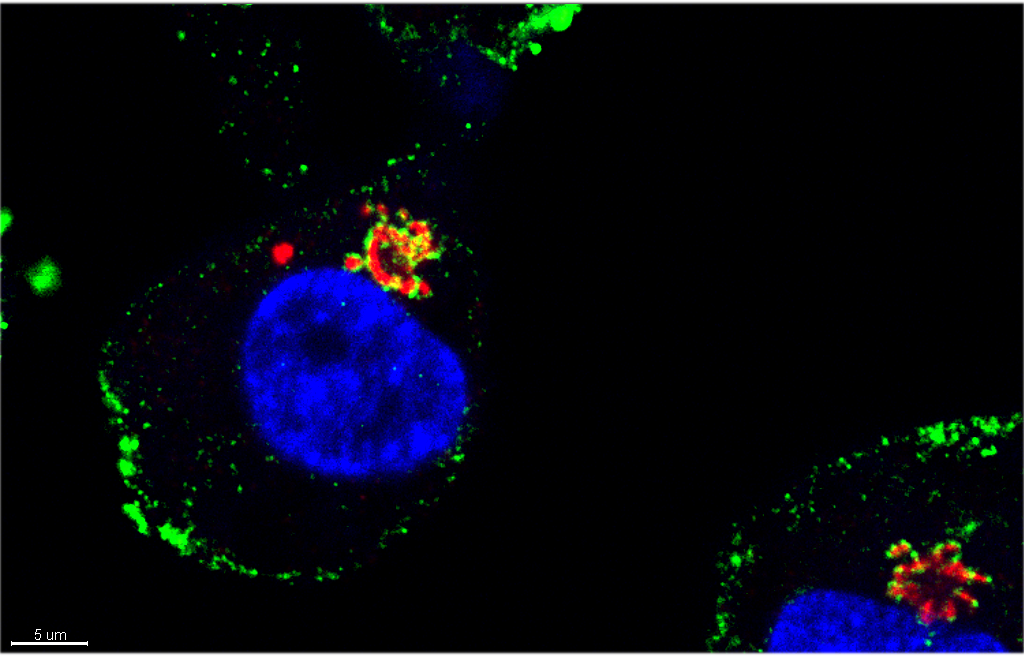

Supplement: Supplementary file 11 — Figure EV1-EV5 Source Data [file 44318_2026_755_MOESM11_ESM.zip › Extended version Figures EV1-EV5/EMBOJ-2025-121050 Figure EV4/Mirocsopy TIF/EV4A/Microscopy PI4P_TGN48.tif]

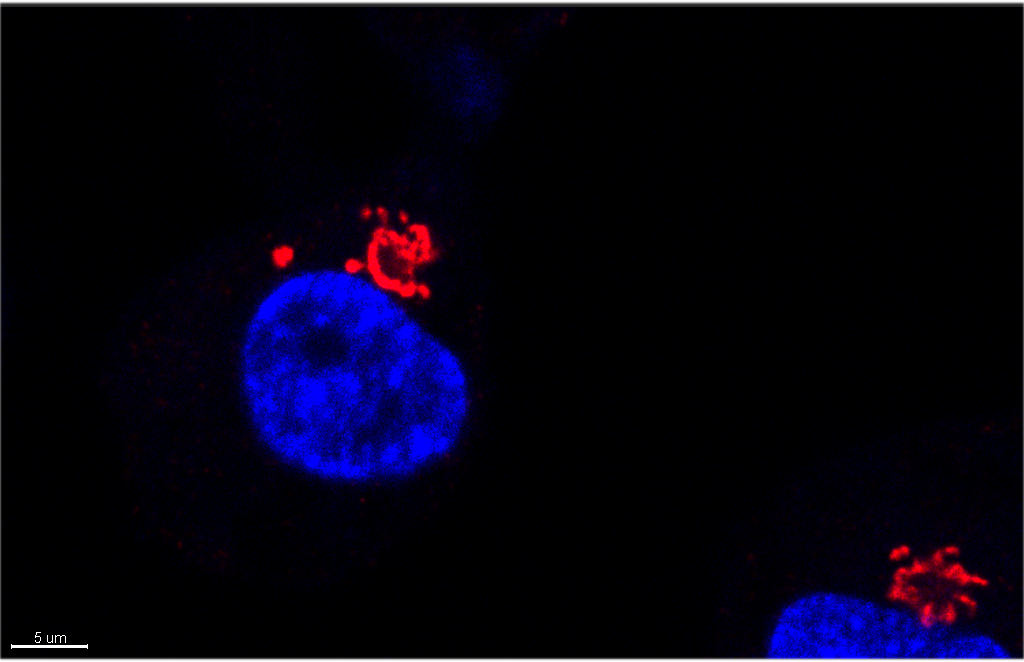

Supplement: Supplementary file 11 — Figure EV1-EV5 Source Data [file 44318_2026_755_MOESM11_ESM.zip › Extended version Figures EV1-EV5/EMBOJ-2025-121050 Figure EV4/Mirocsopy TIF/EV4A/EV4A TGN46 Medium.tif]

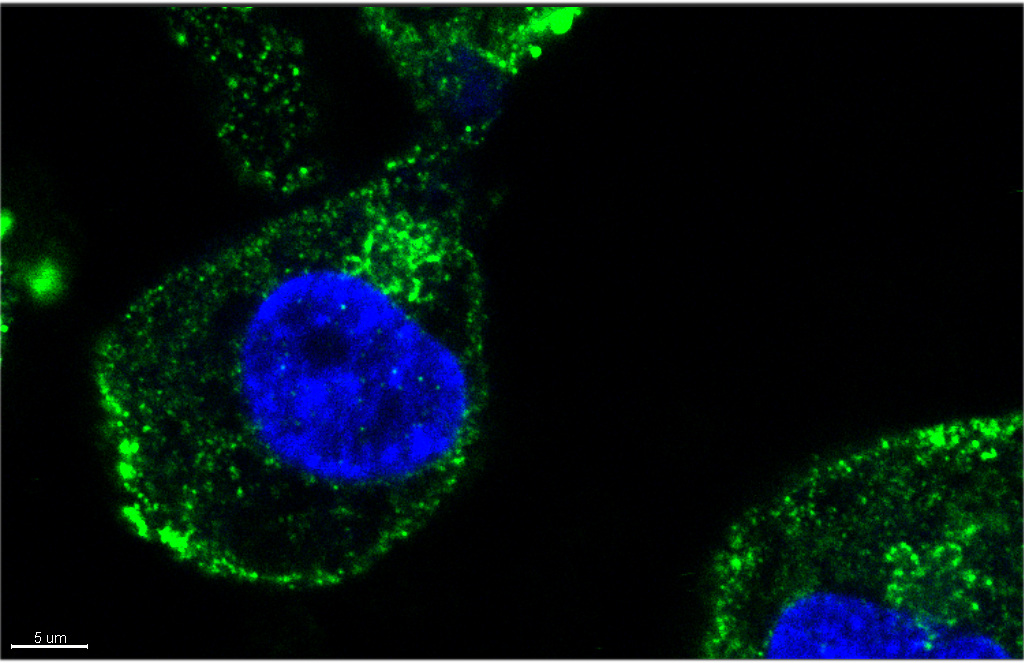

Supplement: Supplementary file 11 — Figure EV1-EV5 Source Data [file 44318_2026_755_MOESM11_ESM.zip › Extended version Figures EV1-EV5/EMBOJ-2025-121050 Figure EV4/Mirocsopy TIF/EV4A/Microscopy PI4P.tif]

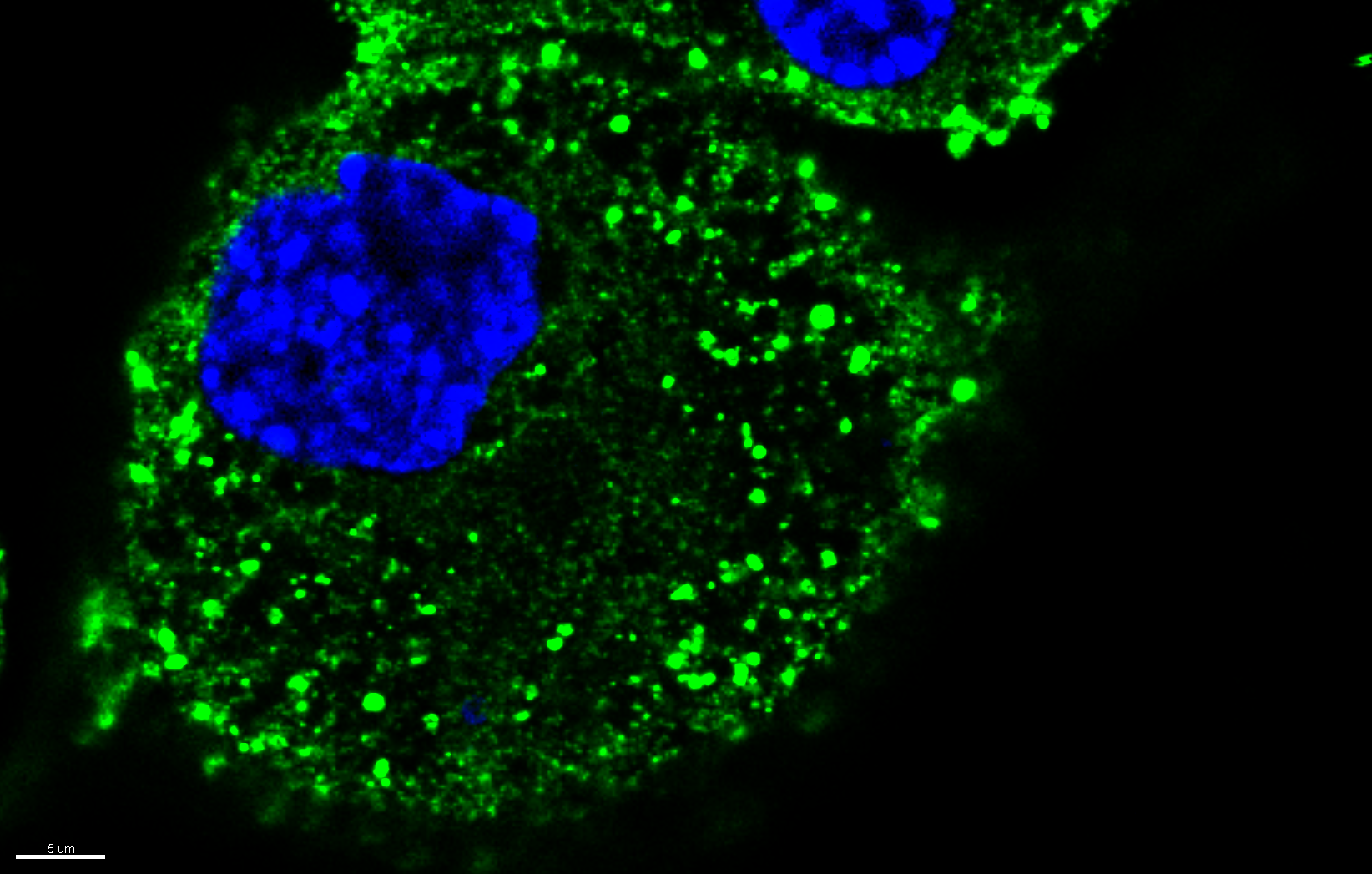

Supplement: Supplementary file 11 — Figure EV1-EV5 Source Data [file 44318_2026_755_MOESM11_ESM.zip › Extended version Figures EV1-EV5/EMBOJ-2025-121050 Figure EV4/Mirocsopy TIF/EV4B/EV4B PI4P LPS.tif]

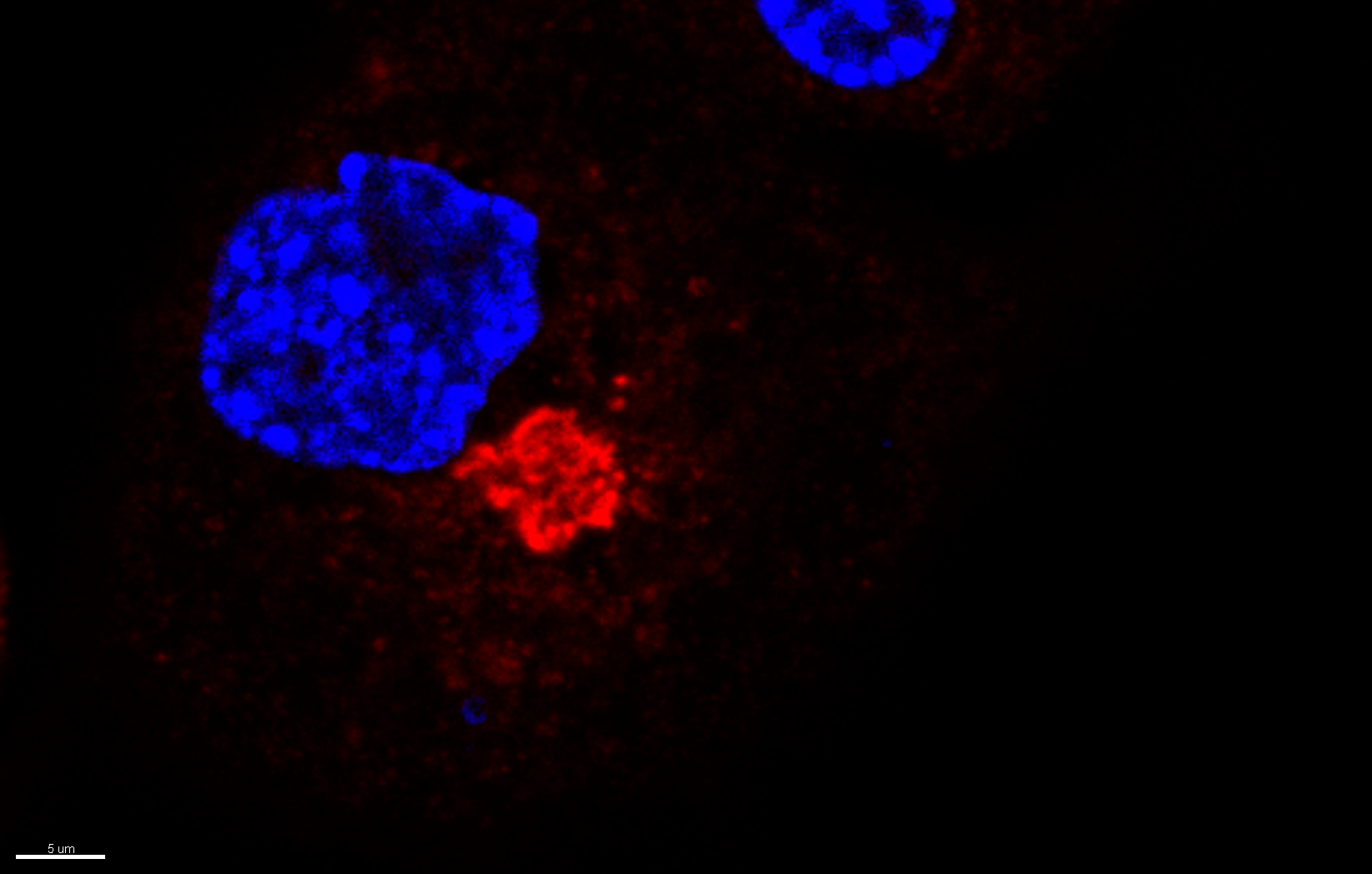

Supplement: Supplementary file 11 — Figure EV1-EV5 Source Data [file 44318_2026_755_MOESM11_ESM.zip › Extended version Figures EV1-EV5/EMBOJ-2025-121050 Figure EV4/Mirocsopy TIF/EV4B/EV4A TGN46 LPS.tif]

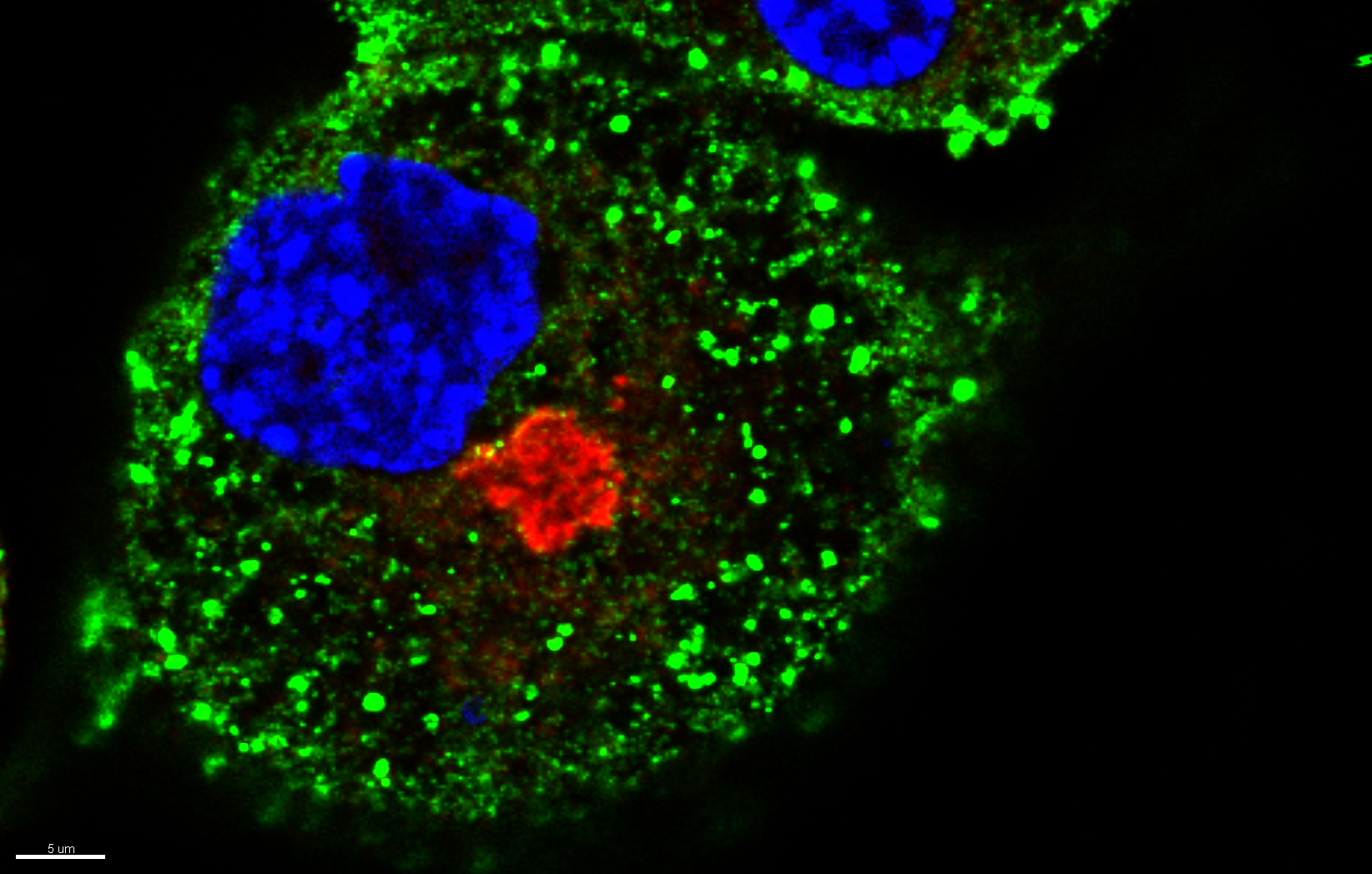

Supplement: Supplementary file 11 — Figure EV1-EV5 Source Data [file 44318_2026_755_MOESM11_ESM.zip › Extended version Figures EV1-EV5/EMBOJ-2025-121050 Figure EV4/Mirocsopy TIF/EV4B/EV4B PI4P_TGN LPS.tif]
