# Supplementary material for: GTPase Rab11b and effector Rab11-FIP2 promote NLRP3 stability during inflammasome priming
Source: EMBO J. 2026 Mar 25;45(9):2991–3021. doi: 10.1038/s44318-026-00755-7 (PMC13144346; doi:10.1038/s44318-026-00755-7)
Supplement: Supplementary file 12 — Expanded View Figures [file 44318_2026_755_MOESM12_ESM.pdf]

Expanded View Figures

Figure EV1. FIP2 is the driver of caspase-1-mediated IL-1β cleavage of the class I FIPs.

(**A**) Quantification of LDH release in THP-1-derived macrophages treated with DMSO or NLRP3 inhibitor MCC950 before LPS priming and nigericin treatment, $n = 2$ independent experiments. (**B**) IL-1β ELISA from the cell supernatants in (**A**). (**C**) Quantification of LDH release in primary human macrophages treated with NS RNA, FIP1 siRNA, FIP2 siRNA or FIP5 siRNA between technical replicates of macrophages from $n = 3$ human donors, $P = 0.0022$ (NS RNA vs FIP2 siRNA) in LPS-primed and nigericin-treated cells. (**D**) Immunoblots of FIP2, FIP1 and β-tubulin in NS RNA-, FIP1 siRNA-, FIP2 siRNA- or FIP5 siRNA-treated human macrophages to verify FIP2 and FIP1 silencing. (**E**) FIP5 mRNA levels measured by RT-qPCR in NS RNA- and FIP5 siRNA-treated human macrophages, between technical replicates from $n = 4$ human donors. $P = 0.0001$ between NS RNA- and FIP2 siRNA-treated macrophages (Welch *t* test). (**F**) Immunoblot of pro-IL-1β, IL-1β p17, pro-caspase-1 (including a light exposure of the blot) and caspase-1 p20 in supernatants from human macrophages treated with NS RNA, FIP1 siRNA, FIP2 siRNA or FIP5 siRNA, one donor of at least $n = 3$ human donors. (**G**) Quantification of IL-1β p17 protein levels in supernatants from NS RNA-, FIP1 siRNA-, FIP2 siRNA- or FIP5 siRNA-treated human macrophages, $n = 4$ human donors. $P = 0.0001$ (NS RNA vs FIP2 siRNA) after LPS and nigericin. (**H**) Quantification of pro-IL-1β protein levels in supernatant of the macrophages from G, $n = 3$ human donors. $P = 0.0159$ (NS RNA vs FIP1 siRNA) and $P = 0.0218$ (NS RNA vs FIP2 siRNA) after LPS and nigericin. (**I**) Quantification of caspase-1 p20 protein levels in the macrophage supernatants, $n = 3$ human donors. (**J**) Quantification of pro-caspase-1 protein levels in the macrophages from (**G**), $n = 3$ donors. The cells were treated with respective siRNAs before primed with 100 ng/mL LPS for 2 h and treated with 5 μM Nigericin for 2 h as indicated. In data (**A**, **B**), data are presented as mean +/− s.d. and in (**C**, **E**, **G**–**J**) as mean +/− s.e.m. and shown as black bars (two-way ANOVA Tukey's multiple comparisons test with adj. *P* values). Source data are available online for this figure.

 © The Author(s)

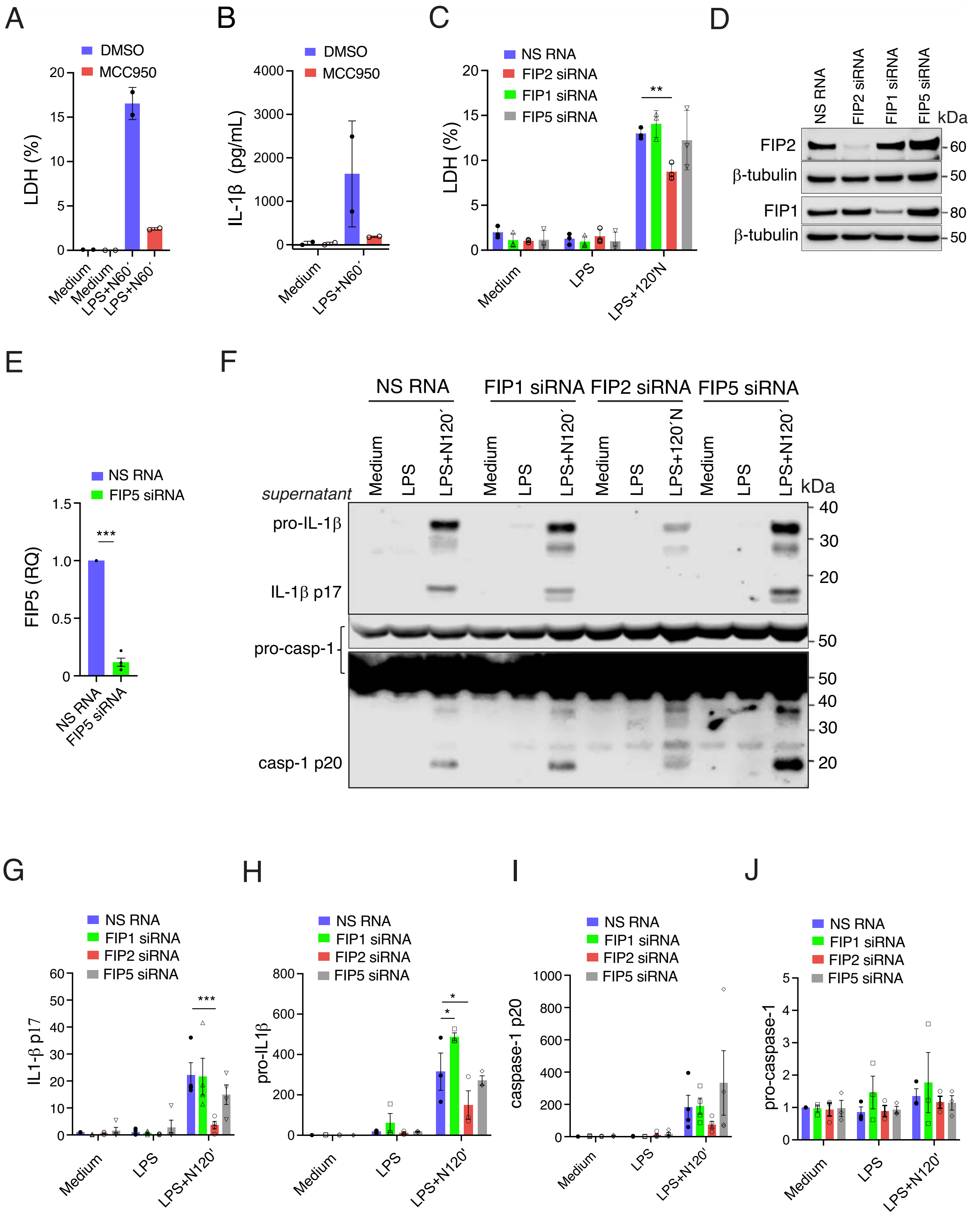
A
B
C
D
E
F
G
H
I
J
DMSO
MCC950
LDH (%)
IL-1β (pg/mL)
Medium
LPS+N60´
NS RNA
FIP2 siRNA
FIP1 siRNA
FIP5 siRNA
LPS
LPS+120´N
**
kDa
FIP2
β-tubulin
FIP1
FIP5 (RQ)
***
supernatant
pro-IL-1β
IL-1β p17
pro-casp-1
casp-1 p20
LPS+N120´
IL1-β p17
pro-IL1β
caspase-1 p20
pro-caspase-1
*

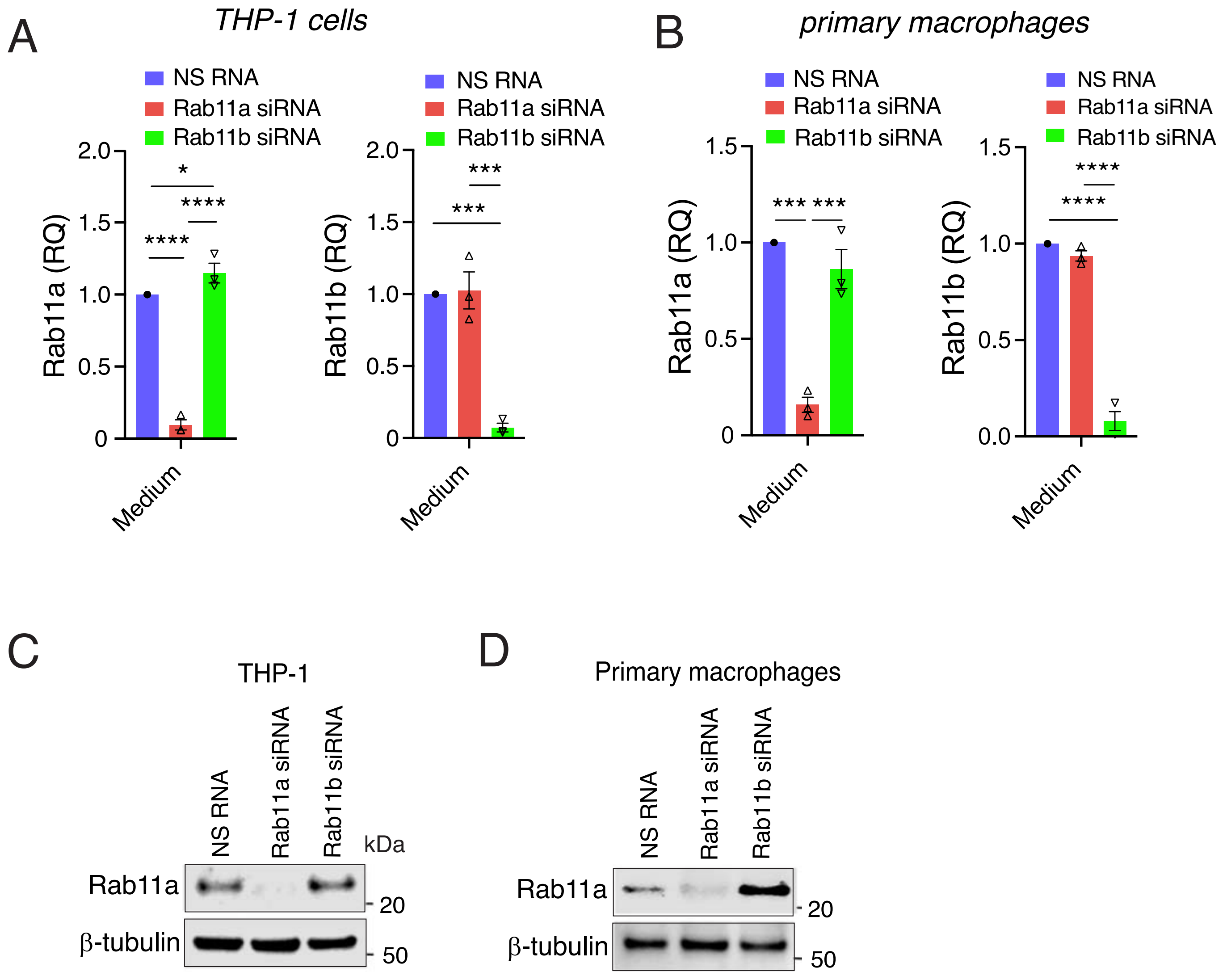

Figure EV2. Rab11a and Rab11b silencing efficiency.

(**A**) Rab11a and Rab11b levels measured by RT-qPCR in THP-1-derived macrophages, $n = 3$ independent experiments. Left panel: $P < 0.0001$ (NS RNA vs Rab11a siRNA), $P = 0.0285$ (NS RNA vs Rab11b siRNA) and $P < 0.0001$ (Rab11a siRNA vs Rab11b siRNA) unstimulated cells. Rigth panel: $P = 0.0004$ (NS RNA vs Rab11b siRNA) and $P = 0.0001$ (Rab11a siRNA vs Rab11b siRNA) unstimulated cells. (**B**) Rab11a and Rab11b levels measured by RT-qPCR in primary human macrophages, $n = 3$ human donors. Left panel: $P = 0.0002$ (NS RNA vs Rab11b siRNA) and $P = 0.0004$ (Rab11a siRNA vs Rab11b siRNA) unstimulated cells. Right panel: $P < 0.0001$ (NS RNA vs Rab11a siRNA) and $P < 0.0001$ (Rab11a siRNA vs Rab11b siRNA) unstimulated cells. (**C**) Rab11a levels measured by Rab11a immunoblotting in THP-1-derived macrophages. (**D**) Rab11a levels measured by Rab11a immunoblotting in primary human macrophages. In data (**A**, **B**), data are presented as mean +/− s.e.m. and shown as black bars (one-way ANOVA Holm-Sidak´s multiple comparisons test with adj. P values). Source data are available online for this figure.

 © The Author(s)

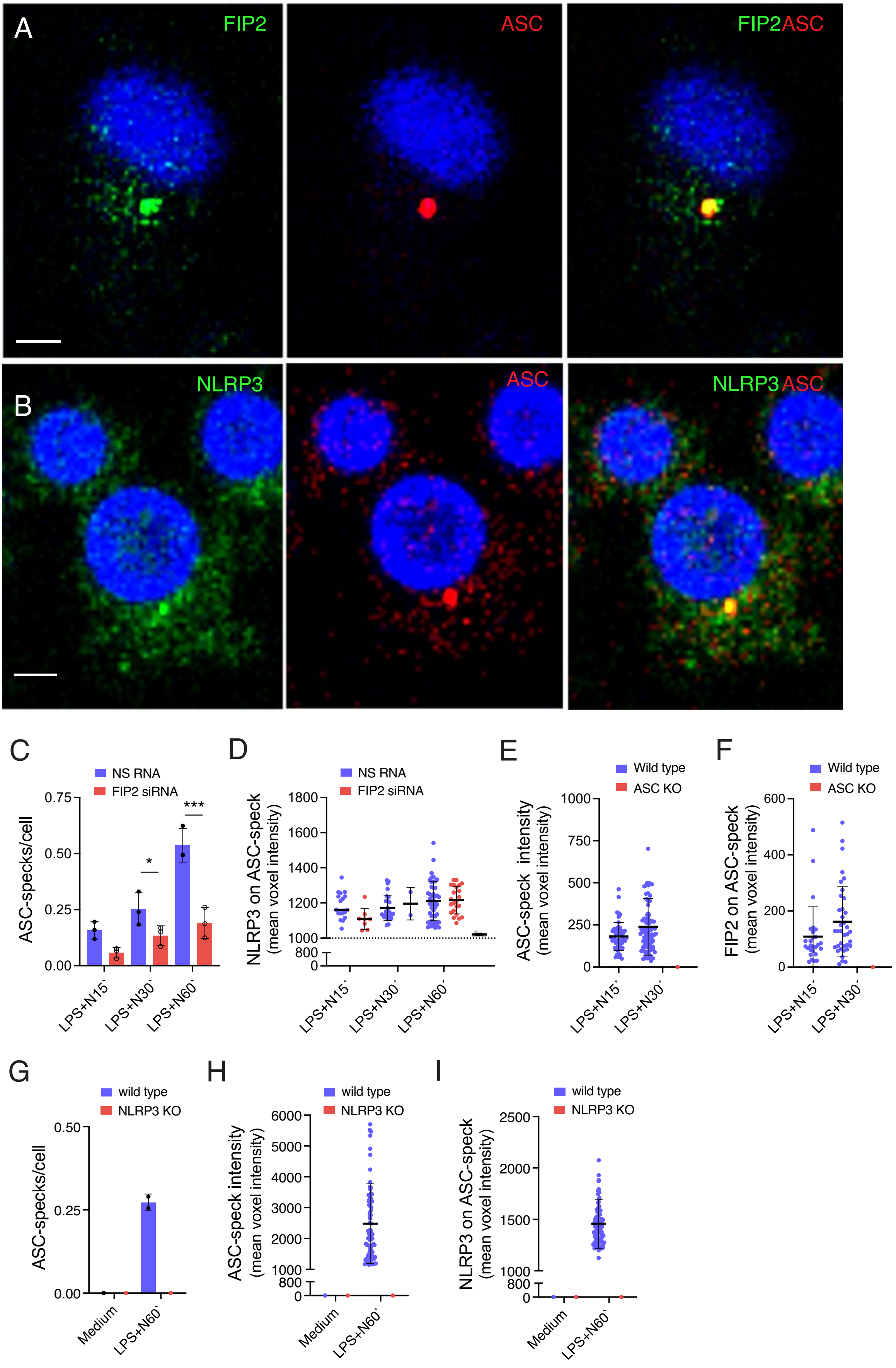

A
FIP2
ASC
FIP2ASC
B
NLRP3
ASC
NLRP3 ASC
C
NS RNA
FIP2 siRNA
ASC-specks/cell
0.75
0.50
0.25
0.00
*
***
LPS+N15`
LPS+N30`
LPS+N60`
D
NS RNA
FIP2 siRNA
NLRP3 on ASC-speck (mean voxel intensity)
1800
1600
1400
1200
1000
800
0
LPS+N15`
LPS+N30`
LPS+N60`
E
Wild type
ASC KO
ASC-speck intensity (mean voxel intensity)
1000
750
500
250
0
LPS+N15`
LPS+N30`
F
Wild type
ASC KO
FIP2 on ASC-speck (mean voxel intensity)
600
400
200
0
LPS+N15`
LPS+N30`
G
wild type
NLRP3 KO
ASC-specks/cell
0.50
0.25
0.00
Medium
LPS+N60`
H
wild type
NLRP3 KO
ASC-speck intensity (mean voxel intensity)
6000
5000
4000
3000
2000
1000
800
0
Medium
LPS+N60`
I
wild type
NLRP3 KO
NLRP3 on ASC-speck (mean voxel intensity)
2500
2000
1500
1000
800
0
Medium
LPS+N60`

Figure EV3. Characterization of ASC speck formation and NLRP3 recruitment.

(**A**) Confocal image showing FIP2 (green) on ASC speck (red). (**B**) Confocal image showing NLRP3 (green) on ASC speck (red). The THP-1-derived macrophages were treated with NS RNA or FIP2 siRNA before they were primed with LPS for 2 h and then treated with nigericin for 30 min (**A**, **B**). (**C**) Quantification of ASC speck formation in LPS-primed cells following 15 min, 30, min and 60 min of nigericin treatment of THP-1-derived macrophages, in $n = 3$ three biological replicates of the same experiment containing 195–239 cells in total. $P = 0.0137$ (NS RNA vs FIP2 siRNA) 30 min nigericin of treatment in LPS-primed cells and $P < 0.0001$ (NS RNA vs FIP2 siRNA) 60 min of nigericin treatment of LPS-primed cells. (**D**) Quantification NLRP3 intensity on ASC specks in LPS primed cells treated with nigericin as indicated, $n = 1$ biological replicate. (**E**) Quantification of ASC speck intensity in wild type and ASC-deficient THP-1-derived macrophages. 266–483 cells were monitored per condition. (**F**) Quantification of FIP2 intensity on ASC specks in the cells of (**E**). (**G**) Quantification of NLRP3 intensity on ASC specks in wild type and NLRP3 KO THP-1-derived macrophages stimulated as indicated, $n = 2$ independent experiments monitoring in total 243–413 cells. (**H**) Quantification of ASC speck intensity in the cells of (**G**). (**I**) Quantification of NLRP3 on ASC specks in the cells of (**G**). ASC specks were identified by the spot detection mode of the IMARIS 8.2 imaging software on 3-D confocal imaging raw data. The cells were treated with respective siRNAs before primed with 100 ng/mL LPS for 2 h and treated with 5 µM Nigericin as indicated. Data in (**D**, **H**, **I**) are presented as 16 bits and therefore have higher values than the data in (**E**, **F**) which is presented as 12 bits. In (**C**), data are presented as mean $+/-$ s.e.m. and shown as black bars (one-way ANOVA Holm–Sidak´s multiple comparisons test with adj. *P* values). N nigericin. Source data are available online for this figure.

© The Author(s)

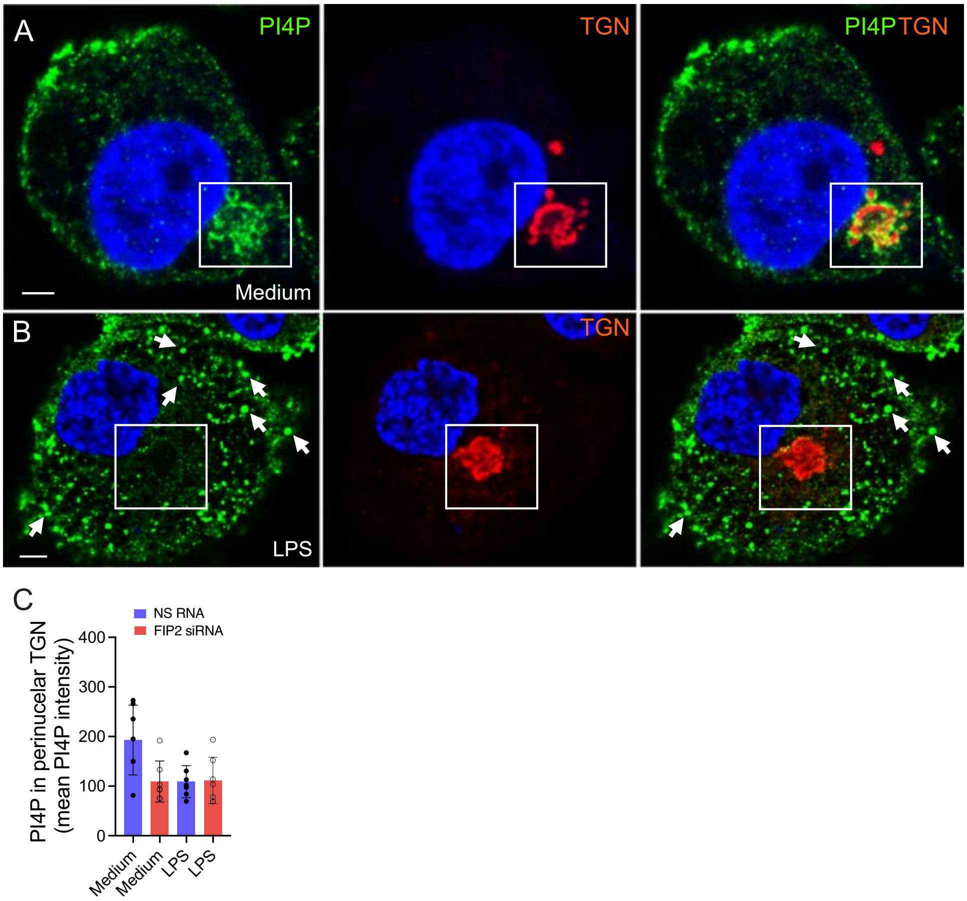

Figure EV4. There is a marked reduction of PI4P in the TGN-ring ring following LPS priming.

(**A**) Confocal images showing PI4P-positive (green) in the perinuclear TGN-ring (red) in unstimulated THP-1-derived macrophages. (**B**) Confocal images showing PI4P (green) TGN46-positive puncta (red) in LPS-primed THP-1-derived macrophages. (**C**) Quantification of PI4P in the perinuclear TGN-ring during. The cells were left unstimulated or primed with 100 ng/mL LPS for 2 h. PI4P was quantified in TGN46-positive ring structures were using the IMARIS 8.2 imaging software on 3-D confocal imaging raw data. In (**C**), data are presented as mean +/− s.d. and shown as black bars. Arrows points at TGN46 puncta positive for PI4P. Scale bar = 5 µm. Source data are available online for this figure.

© The Author(s)

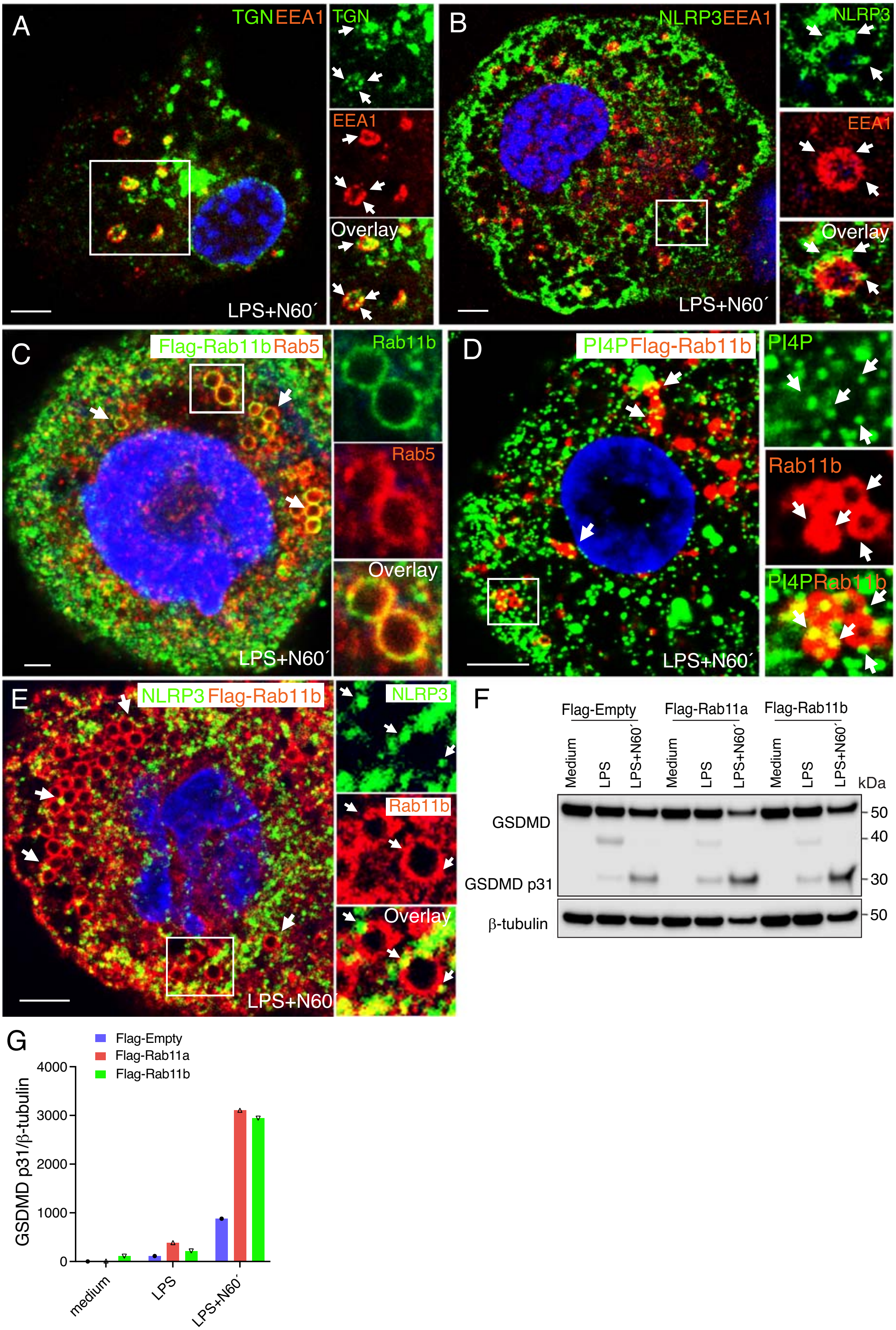
A
TGN EEA1
TGN
EEA1
Overlay
LPS+N60´
B
NLRP3 EEA1
NLRP3
EEA1
Overlay
LPS+N60´
C
Flag-Rab11b Rab5
Rab11b
Rab5
Overlay
LPS+N60´
D
PI4P Flag-Rab11b
PI4P
Rab11b
PI4P Rab11b
LPS+N60´
E
NLRP3 Flag-Rab11b
NLRP3
Rab11b
Overlay
LPS+N60´
F
Flag-Empty
Flag-Rab11a
Flag-Rab11b
Medium
LPS
LPS+N60´
kDa
GSDMD
GSDMD p31
β-tubulin
50
40
30
50
G
Flag-Empty
Flag-Rab11a
Flag-Rab11b
GSDMD p31/β-tubulin
4000
3000
2000
1000
0
medium
LPS
LPS+N60´

 © The Author(s)

Figure EV5. PI4P and NLRP3 are locate to microdomains on Rab11b-positive early endosomes.

(**A**) Confocal image showing TGN46 (green) on EEA1-positive endosomes (red). (**B**) Confocal image showing NLRP3 (green) on a EEA1-positive endosome (red). (**C**) Confocal image showing Rab5 (red) on Rab11b-positive endosomes (green) in Flag-Rab11b expressing THP-1-derived macrophages. (**D**) Confocal image showing PI4P (green) positive microdomains on Flag-Rab11b-positive endosomes. (**E**) Confocal image showing NLRP3 (green) positive microdomains on Flag-Rab11b endosomes (red). (**F**) Immunoblot total GSDMD, caspase-1 cleaved p31 GSDMD fragment and b-tubulin in Flag-Empty, Flag-Rab11a and Flag-Rab11b co-expressing THP-1-derived macrophages. (**G**) Quantification of the GSDMD p31 fragment in the cells from (**F**). The cells were primed with 100 ng/mL LPS for 2 h and treated with 5 µM nigericin for 1 h. Scale bar = 5 µm. Source data are available online for this figure.

© The Author(s)
